# Supplementary material for: Assessing agricultural effects on benthic invertebrate communities in ponds and ditches using δ¹⁵N and δ¹³C isotope niches
Source: PLoS One. 2025 Nov 24;20(11):e0336486. doi: 10.1371/journal.pone.0336486 (PMC12643296; doi:10.1371/journal.pone.0336486)
Supplement: S1 File — (DOCX) [file pone.0336486.s001.docx]

**Supporting information 1: Active substances measured (99) with their limits of quantification and detection, as well as the endpoints and test organism used for the toxicity calculations.**

| **active substance** | **substance class** | **EC50/LC60 invertebrates [mg/L] **** | **test organism invertebrates** | **EC50 macrophytes [mg/L]** | **test organism macrophytes** | **EC50 algae [mg/L]** | **test organism algae** | **LOQ [mg/L]** | **LOD [mg/L]** |
| --- | --- | --- | --- | --- | --- | --- | --- | --- | --- |
| **2,4-D** | herbicide | 12.4 * | Chironomus *sp.* | 2.7 | *Lemna gibba* | 24.2 | *Raphidocelis subcapitata* | 0.001 | 0.001 |
| **acetamiprid** | insecticide | 0.01156 * | Chironomus *sp.* | 1 | *Lemna gibba* | 98.3 | *Scenedesmus subspicatus* | 0.001 | 0.0001 |
| **aclonifen** | herbicide | 1.2 | *Daphnia magna* | 0.006 | *Lemna gibba* | 0.47 | *Navicula pelliculosa* | 0.001 | 0.0005 |
| **amisulbrom** | fungicide | 0.037 | *Daphnia magna* | NA | NA | 0.2 | *Pseudokirchneriella subcapitata* | 0.001 | 0.0005 |
| **azoxystrobin** | fungicide | 0.23 | *Daphnia magna* | 3.2 | *Lemna gibba* | 0.36 | *Pseudokirchneriella subcapitata* | 0.001 | 0.0001 |
| **bentazone** | herbicide | 100 | *Daphnia magna* | 5.4 | *Lemna gibba* | 10.1 | *Anabaena flos-aquae* | 0.001 | 0.0005 |
| **beta-cyfluthrin** | insecticide | 0.00029 | *Daphnia magna* | 0.00084 | *Lemna gibba* | 0.002 | *Scenedesmus subspicatus* | 0.005 | 0.001 |
| **bifenox** | herbicide | 0.66 | *Daphnia magna* | 0.0021 | *Lemna gibba* | 0.00018 | *Scenedesmus subspicatus* | 0.05 | 0.005 |
| **bifenthrin** | insecticide | 0.00011 | *Daphnia magna* | NA | NA | 0.822 | *Scenedesmus subspicatus* | 0.005 | 0.001 |
| **bixafen** | fungicide | 1.2 | *Daphnia magna* | NA | NA | 0.097 | *Pseudokirchneriella subcapitata* | 0.001 | 0.0001 |
| **boscalid** | fungicide | 5.33 | *Daphnia magna* | 3.9 | *Lemna gibba* | 3.75 | *Pseudokirchneriella subcapitata* | 0.001 | 0.0005 |
| **bromoxynil** | herbicide | 12.5 | *Daphnia magna* | 0.033 | *Lemna gibba* | 0.12 | *Navicula pelliculosa* | 0.001 | 0.001 |
| **chlorantraniliprole** | insecticide | 0.0116 | *Daphnia magna* | 2 | *Lemna gibba* | 4 | *Pseudokirchneriella subcapitata* | 0.001 | 0.0005 |
| **chloridazon** | herbicide | 132 | *Daphnia magna* | 3.16 | *Lemna gibba* | 3 | *Pseudokirchneriella subcapitata* | 0.001 | 0.001 |
| **chlorpyrifos** | insecticide | 0.000024 | *Chironomus riparius* | 0.53 | *Lemna minor* | 0.48 | Unknown species | 0.0001 | 0.0001 |
| **chlortoluron** | herbicide | 67 | *Daphnia magna* | 0.038 | *Lemna gibba* | 0.024 | *Pseudokirchneriella subcapitata* | 0.001 | 0.0001 |
| **clothianidin** | insecticide | 0.029 | *Chironomus riparius* | 121 | *Lemna gibba* | 55 | *Pseudokirchneriella subcapitata* | 0.001 | 0.0005 |
| **cyazofamid** | fungicide | 0.19 | *Daphnia magna* | 0.033 | Unknown species | 0.025 | *Raphidocelis subcapitata* | 0.001 | 0.0001 |
| **cypermethrin** | insecticide | 0.00021 | *Daphnia magna* | NA | NA | 0.0667 | *Pseudokirchneriella subcapitata* | 0.005 | 0.001 |
| **cyprodinil** | fungicide | 0.22 | *Daphnia magna* | 7.71 | *Lemna gibba* | 2.6 | *Pseudokirchneriella subcapitata* | 0.005 | 0.005 |
| **dichlorprop** | herbicide | 100 | *Daphnia magna* | NA | NA | 1100 | *Pseudokirchneriella subcapitata* | 0.05 | 0.05 |
| **difenoconazole** | fungicide | 0.77 | *Daphnia magna* | 2.5 | *Lemna gibba* | 0.032 | *Scenedesmus subspicatus* | 0.001 | 0.0005 |
| **diflufenican** | herbicide | 0.24 | *Daphnia magna* | 0.056 | *Lemna gibba* | 0.00025 | *Scenedesmus subspicatus* | 0.001 | 0.0001 |
| **dimethachlor** | herbicide | 24 | *Daphnia magna* | 0.035 | *Lemna gibba* | 0.0065 | *Desmodesmus subspicatus* | 0.001 | 0.0001 |
| **dimethenamid-P** | herbicide | 3.2 | *Daphnia magna* | 0.006 | *Lemna gibba* | 0.019 | *Pseudokirchneriella subcapitata* | 0.001 | 0.0001 |
| **dimethoate** | insecticide | 0.76945 * | Chironomus *sp.* | NA | NA | 90.4 | *Raphidocelis subcapitata* | 0.001 | 0.0005 |
| **dimethomorph** | fungicide | 20.1 | *Daphnia magna* | 1 | *Lemna gibba* | 29.2 | *Scenedesmus subspicatus* | 0.001 | 0.0001 |
| **dimoxystrobin** | fungicide | 0.0394 | *Daphnia magna* | NA | NA | 0.017 | *Pseudokirchneriella subcapitata* | 0.001 | 0.0005 |
| **epoxiconazole** | fungicide | 0.0625 | *Chironomus riparius* | 0.014 | *Lemna gibba* | 10.69 | *Pseudokirchneriella subcapitata* | 0.001 | 0.0001 |
| **esfenvalerate** | insecticide | 0.00013 | *Chironomus riparius* | NA | NA | 0.0065 | *Pseudokirchneriella subcapitata* | 0.005 | 0.001 |
| **famoxadone** | fungicide | 0.033 | *Daphnia magna* | 0.0081 | *Lemna gibba* | 0.00308 | *Pseudokirchneriella subcapitata* | 0.05 | 0.005 |
| **fenpropidin** | fungicide | 0.54 | *Daphnia magna* | NA | NA | 0.0057 | *Scenedesmus subspicatus* | 0.001 | 0.0005 |
| **fenpropimorph** | fungicide | 2.24 | *Daphnia magna* | NA | NA | 0.327 | *Pseudokirchneriella subcapitata* | 0.001 | 0.001 |
| **florasulam** | herbicide | 292 | *Daphnia magna* | 0.001 | *Lemna gibba* | 0.00894 | Unknown species | 0.001 | 0.001 |
| **fludioxonil** | fungicide | 0.4 | *Daphnia magna* | 0.92 | *Lemna gibba* | 0.024 | *Scenedesmus subspicatus* | 0.001 | 0.0001 |
| **flufenacet** | herbicide | 30.9 | *Daphnia magna* | 0.002 | *Lemna gibba* | 0.00204 | *Raphidocelis subcapitata* | 0.001 | 0.0001 |
| **flumioxazin** | herbicide | 5.9 | *Daphnia magna* | 0.00035 | *Lemna gibba* | 0.000852 | *Raphidocelis subcapitata* | 0.001 | 0.0005 |
| **fluopicolide** | fungicide | 1.8 | *Daphnia magna* | 3.2 | *Lemna gibba* | 0.029 | *Navicula pelliculosa* | 0.001 | 0.0001 |
| **flupyrsulfuron-methyl** | herbicide | 721 | *Daphnia magna* | 0.0025 | *Lemna gibba* | 0.0037 | *Raphidocelis subcapitata* | 0.001 | 0.001 |
| **fluquinconazole** | fungicide | 5 | *Daphnia magna* | 1.4 | *Lemna minor* | 0.046 | *Pseudokirchneriella subcapitata* | 0.001 | 0.001 |
| **fluroxypyr** | herbicide | 100 | *Daphnia magna* | 12.3 | *Lemna gibba* | 49.8 | *Pseudokirchneriella subcapitata* | 0.05 | 0.05 |
| **flurtamone** | herbicide | 13 | *Daphnia magna* | 0.0141 | *Lemna gibba* | 0.073 | *Pseudokirchneriella subcapitata* | 0.001 | 0.0001 |
| **fluxapyroxad** | fungicide | 6.78 | *Daphnia magna* | 2.19 | *Lemna gibba* | 0.7 | *Pseudokirchneriella subcapitata* | 0.05 | 0.05 |
| **foramsulfuron** | herbicide | 100 | *Daphnia magna* | 0.001 | *Lemna gibba* | 8.1 | *Anabaena flos-aquae* | 0.001 | 0.001 |
| **imazosulfuron** | herbicide | 91 | *Daphnia magna* | 0.00075 | *Lemna gibba* | 0.69 | *Pseudokirchneriella subcapitata* | 0.001 | 0.001 |
| **imidacloprid** | insecticide | 0.008775 * | Chironomus *sp.* | NA | NA | 10 | *Scenedesmus subspicatus* | 0.001 | 0.001 |
| **iodosulfuron-methyl** | herbicide | 100 | *Daphnia magna* | 0.00083 | *Lemna gibba* | 0.07 | *Pseudokirchneriella subcapitata* | 0.001 | 0.001 |
| **isoproturon** | herbicide | 0.58 | Unknown species | 0.031 | *Lemna minor* | 0.013 | *Navicula pelliculosa* | 0.001 | 0.0005 |
| **isopyrazam** | fungicide | 0.044 | *Daphnia magna* | 0.5 | *Lemna gibba* | 2.2 | *Pseudokirchneriella subcapitata* | 0.001 | 0.0001 |
| **lambda-cyhalothrin** | insecticide | 0.00023 | *Daphnia magna* | NA | NA | 0.005 | *Pseudokirchneriella subcapitata* | 0.05 | 0.001 |
| **lenacil** | herbicide | 8.4 | *Daphnia magna* | 0.019 | *Lemna gibba* | 0.0077 | *Pseudokirchneriella subcapitata* | 0.001 | 0.0005 |
| **MCPA** | herbicide | 190 | *Daphnia magna* | 0.152 | *Lemna gibba* | 79.8 | *Pseudokirchneriella subcapitata* | 0.001 | 0.0005 |
| **mecoprop** | herbicide | 200 | *Daphnia magna* | 40.2 | Unknown species | 237 | Unknown species | 0.001 | 0.0005 |
| **mesosulfuron-methyl** | herbicide | 100 | *Daphnia magna* | 0.00062 | *Lemna gibba* | 0.2 | *Raphidocelis subcapitata* | 0.001 | 0.001 |
| **mesotrione** | herbicide | 622 | *Daphnia magna* | 0.022 | *Lemna minor* | 3.5 | *Raphidocelis subcapitata* | 0.05 | 0.05 |
| **metamitron** | herbicide | 5.7 | *Daphnia magna* | 0.4 | *Lemna minor* | 0.4 | *Pseudokirchneriella subcapitata* | 0.005 | 0.005 |
| **metazachlor** | herbicide | 33 | *Daphnia magna* | 0.0023 | *Lemna gibba* | 0.0162 | *Pseudokirchneriella subcapitata* | 0.001 | 0.0001 |
| **methiocarb** | insecticide | 0.008 | *Daphnia magna* | NA | NA | 2.2 | *Scenedesmus subspicatus* | 0.001 | 0.001 |
| **metosulam** | herbicide | 100 | *Daphnia magna* | 0.0023 | *Lemna gibba* | 0.075 | *Scenedesmus subspicatus* | 0.001 | 0.0005 |
| **metrafenone** | fungicide | 0.92 | *Daphnia magna* | 0.327 | *Lemna gibba* | 0.71 | *Raphidocelis subcapitata* | 0.001 | 0.0001 |
| **metribuzin** | herbicide | 49 | *Daphnia magna* | 0.0161 | *Lemna gibba* | 0.0266 | *Desmodesmus subspicatus* | 0.001 | 0.001 |
| **metsulfuron-methyl** | herbicide | 43.1 | *Daphnia magna* | 0.00036 | *Lemna gibba* | 0.113 | *Anabaena flos-aquae* | 0.001 | 0.001 |
| **napropamide** | herbicide | 14.3 | *Daphnia magna* | 0.24 | *Lemna gibba* | 3.4 | *Pseudokirchneriella subcapitata* | 0.001 | 0.0001 |
| **nicosulfuron** | herbicide | 90 | *Daphnia magna* | 0.002 | *Lemna gibba* | 7.8 | *Pseudokirchneriella subcapitata* | 0.001 | 0.0005 |
| **pendimethalin** | herbicide | 0.147 | *Daphnia magna* | 0.022 | *Lemna gibba* | 0.004 | *Selenastrum capricornutum* | 0.001 | 0.001 |
| **pethoxamid** | herbicide | 23 | *Daphnia magna* | 0.0247 | *Lemna gibba* | 149 | *Pseudokirchneriella subcapitata* | 0.001 | 0.0001 |
| **phenmedipham** | herbicide | 2.033 | *Daphnia magna* | 0.157 | *Lemna minor* | 0.086 | *Raphidocelis subcapitata* | 0.05 | 0.05 |
| **picolinafen** | herbicide | 0.45 | *Daphnia magna* | 0.057 | *Lemna gibba* | 0.00018 | *Pseudokirchneriella subcapitata* | 0.001 | 0.0001 |
| **picoxystrobin** | fungicide | 0.024 | *Daphnia magna* | 0.26 | *Lemna gibba* | 0.056 | *Raphidocelis subcapitata* | 0.001 | 0.001 |
| **pirimicarb** | insecticide | 0.017 | *Daphnia magna* | NA | NA | 140 | *Pseudokirchneriella subcapitata* | 0.001 | 0.0001 |
| **prochloraz** | fungicide | 4.3 | *Daphnia magna* | 0.171 | *Lemna gibba* | 0.0055 | *Scenedesmus subspicatus* | 0.001 | 0.0005 |
| **propamocarb** | fungicide | 106 | *Daphnia magna* | NA | NA | 301 | *Scenedesmus quadricauda* | 0.001 | 0.0001 |
| **propiconazole** | fungicide | 10.2 | *Daphnia magna* | 4.9 | *Lemna gibba* | 0.093 | *Navicula seminulum* | 0.001 | 0.001 |
| **propyzamide** | herbicide | 5.6 | *Daphnia magna* | 1.4 | *Lemna gibba* | 2.8 | *Raphidocelis subcapitata* | 0.001 | 0.0001 |
| **proquinazid** | fungicide | 0.287 | *Daphnia magna* | 0.2 | *Lemna gibba* | 0.684 | *Pseudokirchneriella subcapitata* | 0.001 | 0.001 |
| **prosulfocarb** | herbicide | 0.51 | *Daphnia magna* | 0.69 | *Lemna gibba* | 0.049 | *Pseudokirchneriella subcapitata* | 0.001 | 0.0005 |
| **prosulfuron** | herbicide | 120 | *Daphnia magna* | 0.00126 | *Lemna gibba* | 0.0089 | *Raphidocelis subcapitata* | 0.001 | 0.001 |
| **pymetrozine** | insecticide | 87 | *Daphnia magna* | 106 | *Lemna gibba* | 21.6 | *Raphidocelis subcapitata* | 0.001 | 0.0005 |
| **pyraclostrobin** | fungicide | 0.016 | *Daphnia magna* | 1.72 | *Lemna gibba* | 0.843 | *Pseudokirchneriella subcapitata* | 0.001 | 0.0005 |
| **pyraflufen-ethyl** | herbicide | 0.1 | *Daphnia magna* | 0.0026 | *Lemna gibba* | 0.00023 | *Raphidocelis subcapitata* | 0.001 | 0.001 |
| **pyroxsulam** | herbicide | 100 | *Daphnia magna* | 0.0026 | *Lemna gibba* | 0.924 | *Pseudokirchneriella subcapitata* | 0.001 | 0.0005 |
| **quinmerac** | herbicide | 100 | *Daphnia magna* | 96 | *Lemna gibba* | 48.5 | *Chlorella fusca* | 0.001 | 0.001 |
| **quinoxyfen** | fungicide | 0.08 | *Daphnia magna* | 1.85 | *Lemna gibba* | 0.027 | *Raphidocelis subcapitata* | 0.001 | 0.0001 |
| **quizalofop-P** | herbicide | NA | NA | NA | NA | NA | *NA* | 0.05 | 0.05 |
| **S-metolachlor** | herbicide | 11.2 | *Daphnia magna* | 0.037 | *Lemna gibba* | 0.017 | *Pseudokirchneriella subcapitata* | 0.001 | 0.0005 |
| **spinosyn A** | insecticide | 6.5 | *Daphnia magna* | NA | NA | NA | NA | 0.001 | 0.0001 |
| **spiroxamine** | fungicide | 6.1 | *Daphnia magna* | 2.76 | *Lemna gibba* | 0.003 | *Scenedesmus subspicatus* | 0.001 | 0.0001 |
| **sulcotrione** | herbicide | 848 | *Daphnia magna* | 0.051 | *Lemna gibba* | 1.2 | *Raphidocelis subcapitata* | 0.005 | 0.005 |
| **tau-fluvalinate** | insecticide | 0.0089 | *Daphnia magna* | NA | NA | 42 | *Scenedesmus subspicatus* | 0.005 | 0.001 |
| **tebuconazole** | fungicide | 2.79 | *Daphnia magna* | 0.144 | *Lemna gibba* | 1.96 | *Scenedesmus subspicatus* | 0.001 | 0.0001 |
| **tefluthrin** | insecticide | 0.00007 | *Daphnia magna* | NA | NA | 1.05 | *Pseudokirchneriella subcapitata* | 0.005 | 0.001 |
| **terbuthylazine** | herbicide | 21.2 | *Daphnia magna* | 0.0128 | *Lemna gibba* | 0.012 | *Pseudokirchneriella subcapitata* | 0.001 | 0.0001 |
| **thiacloprid** | insecticide | 0.00106 * | Chironomus *sp.* | 95.4 | *Lemna gibba* | 60.6 | *Selenastrum capricornutum* | 0.001 | 0.0001 |
| **thiamethoxam** | insecticide | 0.078 * | Chironomus *sp.* | 90 | *Lemna gibba* | 100 | *Pseudokirchneriella subcapitata* | 0.001 | 0.0001 |
| **thifensulfuron-methyl** | herbicide | 60.7 | *Daphnia magna* | 0.0013 | *Lemna gibba* | 0.8 | *Pseudokirchneriella subcapitata* | 0.001 | 0.0005 |
| **triasulfuron** | herbicide | 100 | *Daphnia magna* | 0.000068 | *Lemna gibba* | 0.035 | *Raphidocelis subcapitata* | 0.001 | 0.0005 |
| **trifloxystrobin** | fungicide | 0.011 | *Daphnia magna* | 1.93 | *Lemna gibba* | 0.0053 | *Scenedesmus subspicatus* | 0.001 | 0.0001 |
| **tritosulfuron** | herbicide | 100 | *Daphnia magna* | 0.026 | *Lemna gibba* | 0.23 | *Pseudokirchneriella subcapitata* | 0.001 | 0.001 |
| **zoxamide** | fungicide | 0.78 | *Daphnia magna* | 0.017 | *Lemna gibba* | 0.011 | *Scenedesmus subspicatus* | 0.001 | 0.0001 |

* Source Ecotox (35), all other values are taken from the pesticides properties database (PPDB) from (34)

*** Daphnia magna* EC50 values, Chironomus sp. and *Chironomus riparius* LC50 values
