# Supplementary material for: Assessing agricultural effects on benthic invertebrate communities in ponds and ditches using δ¹⁵N and δ¹³C isotope niches
Source: PLoS One. 2025 Nov 24;20(11):e0336486. doi: 10.1371/journal.pone.0336486 (PMC12643296; doi:10.1371/journal.pone.0336486)
Supplement: S2 File — Ellipses incorporate 40% of the data. (DOCX) [file pone.0336486.s002.docx]

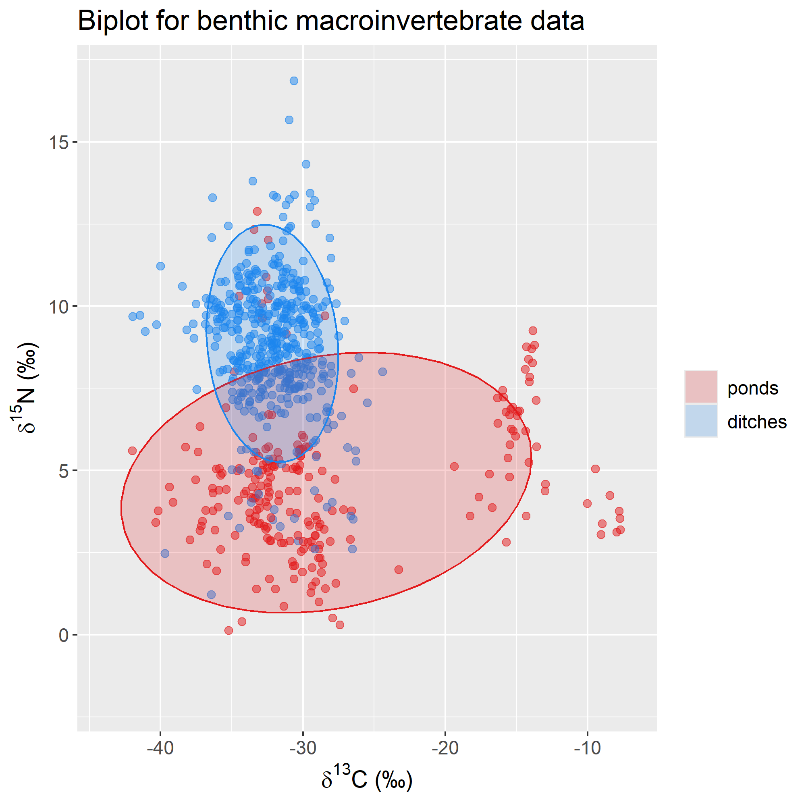


**Supporting information 2: Biplot for benthic macroinvertebrate before correction for δ^15^N values from and the influence of organic fertilization. Ellipses incorporate 40% of the data.**
