# Supplementary material for: Assessing agricultural effects on benthic invertebrate communities in ponds and ditches using δ¹⁵N and δ¹³C isotope niches
Source: PLoS One. 2025 Nov 24;20(11):e0336486. doi: 10.1371/journal.pone.0336486 (PMC12643296; doi:10.1371/journal.pone.0336486)
Supplement: S3 File — Black dots are the modes. Shaded boxes represent the 50%, 75% and 95% confidence intervals, from dark to light red/blue. Grouping is based on the FFG (six groups), the communities are assigned for the water body types (pond, ditch). (DOCX) [file pone.0336486.s003.docx]

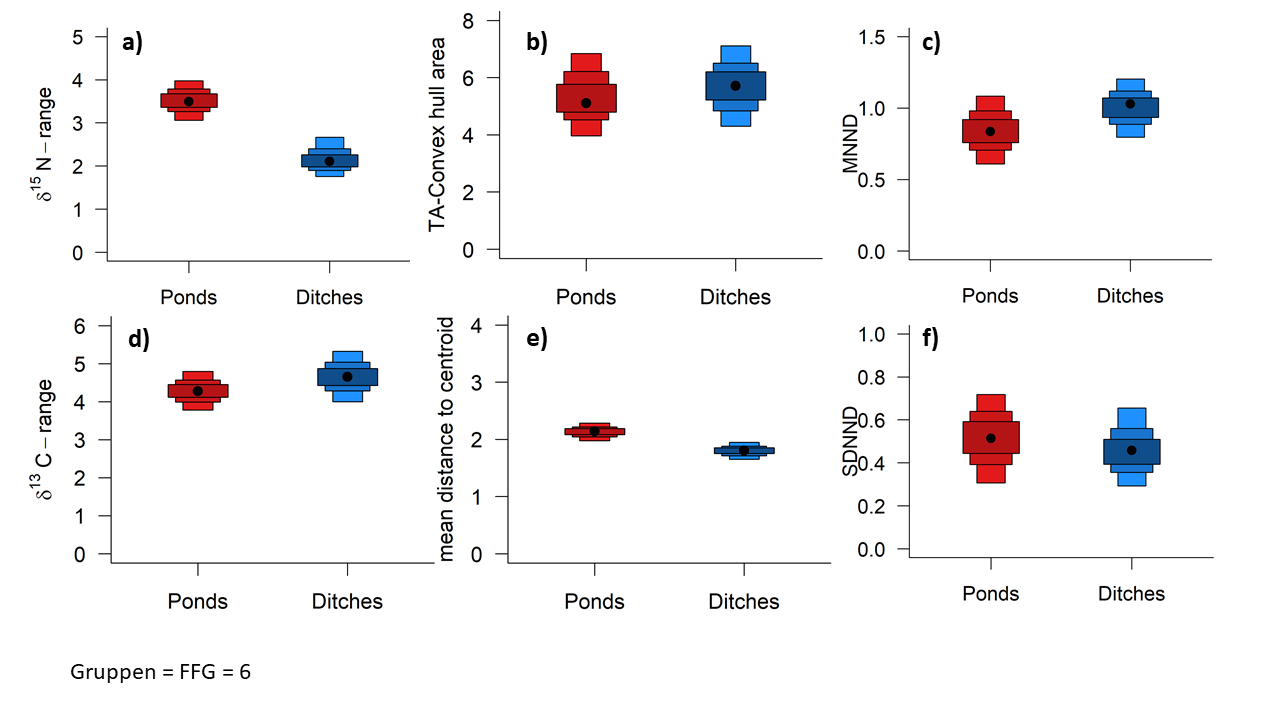


**Supporting information 3: SIBER density plots for the Layman metrics, computed via Bayesian analysis, corrected for δ^15^N and δ^13^C values. Black dots are the modes. Shaded boxes represent the 50 %, 75 % and 95 % confidence intervals, from dark to light red/blue. Grouping is based on the FFG (six groups), the communities are assigned for the water body types (pond, ditch).**
