# Supplementary material for: Assessing agricultural effects on benthic invertebrate communities in ponds and ditches using δ¹⁵N and δ¹³C isotope niches
Source: PLoS One. 2025 Nov 24;20(11):e0336486. doi: 10.1371/journal.pone.0336486 (PMC12643296; doi:10.1371/journal.pone.0336486)
Supplement: S4 File — (DOCX) [file pone.0336486.s004.docx]

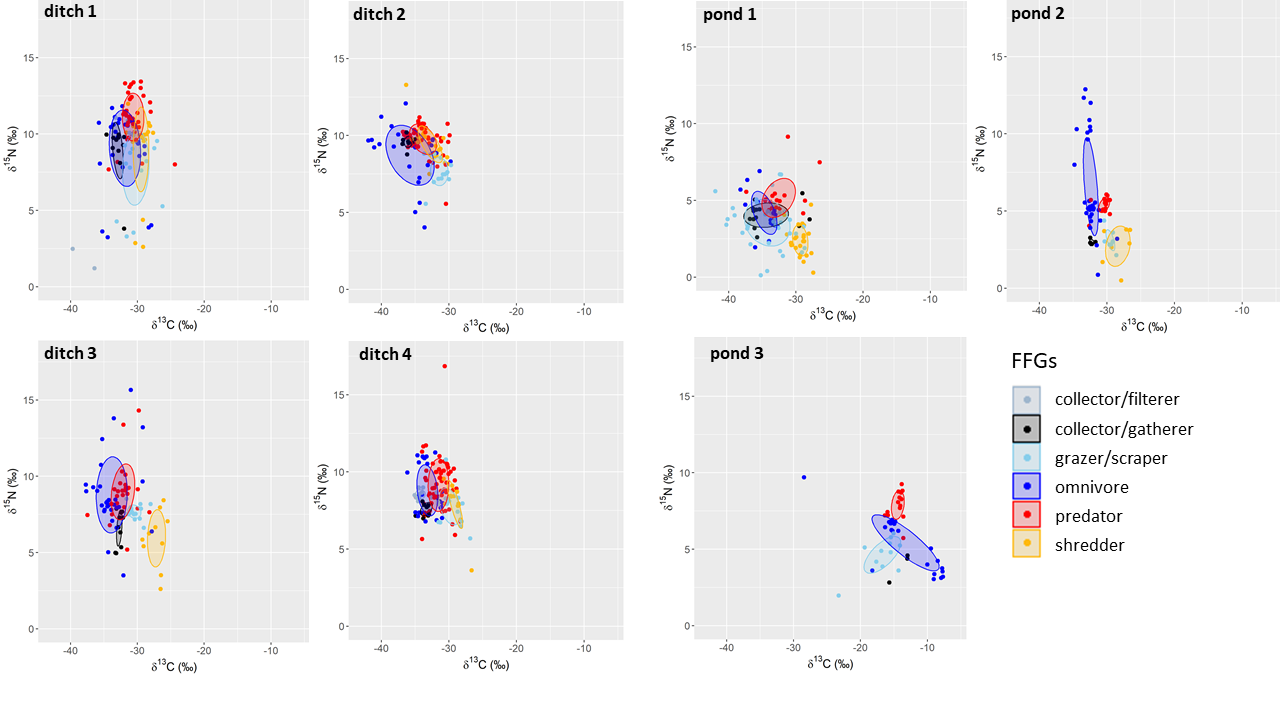


**Supporting information 4: Representation of the FFGs as coloured dots and ellipses, 40% of the data points are represented by the ellipses.**
