# Supplementary material for: Assessing agricultural effects on benthic invertebrate communities in ponds and ditches using δ¹⁵N and δ¹³C isotope niches
Source: PLoS One. 2025 Nov 24;20(11):e0336486. doi: 10.1371/journal.pone.0336486 (PMC12643296; doi:10.1371/journal.pone.0336486)
Supplement: S5 File — (DOCX) [file pone.0336486.s005.docx]

**Supporting information 5: Overview of the sampled FFGs and determination for the ponds and ditches.**

| **FFG** | **ponds** | | | | **ditches** | | | |
| --- | --- | --- | --- | --- | --- | --- | --- | --- |
|  | **taxagroup** | **family_subfamily** | **genus** | **N** | **taxagroup** | **family_subfamily** | **genus** | **N** |
| collector_filterer |  |  |  |  | Bivalvia | Sphaeriidae | *Sphaerium* sp. | 9 |
| collector_filterer |  |  |  |  | Bivalvia | NA | NA | 1 |
| collector_filterer |  |  |  |  | Diptera | Culicidae | *Culex* sp. | 2 |
| collector_gatherer | Diptera | Eristalinae | NA | 1 |  |  |  |  |
| collector_gatherer | Diptera | Stratiomyidae | NA | 2 |  |  |  |  |
| collector_gatherer | Ephemeroptera | Baetidae | *Cloeon* sp. | 13 | Ephemeroptera | Baetidae | *Cloeon* sp. | 37 |
| collector_gatherer | Ephemeroptera | NA | NA | 5 |  |  |  |  |
| collector_gatherer |  |  |  |  | Ephemeroptera | Caenidae | *Caenis* sp. | 3 |
| collector_gatherer |  |  |  |  | Oligoacheta | NA | NA | 1 |
| grazer_scraper | Diptera | Culicidae | *Anopheles* sp. | 8 |  |  |  |  |
| grazer_scraper | Gastropoda | Acroloxoidea | NA | 1 |  |  |  |  |
| grazer_scraper | Gastropoda | Lymnaeidae | *Radix* sp. | 6 | Gastropoda | Lymnaeidae | *Radix* sp. | 7 |
| grazer_scraper | Gastropoda | Lymnaeidae | *Stagnicola* sp. | 15 |  |  |  |  |
| grazer_scraper |  |  |  |  | Gastropoda | Lymnaeidae | *Lymnaea* sp. | 22 |
| grazer_scraper | Gastropoda | Lymnaeidae | NA | 10 | Gastropoda | Lymnaeidae | NA | 9 |
| grazer_scraper | Gastropoda | Planorbidae | NA | 14 | Gastropoda | Planorbidae | NA | 12 |
| grazer_scraper | Gastropoda | Viviparidae | NA | 2 | Gastropoda | Bithyniidae | *Bithynia* sp. | 4 |
| grazer_scraper |  |  |  |  | Gastropoda | Planorbidae | *Anisus* sp. | 1 |
| grazer_scraper |  |  |  |  | Gastropoda | Planorbidae | *Planorbarius* sp. | 3 |
| grazer_scraper |  |  |  |  | Gastropoda | Planorbidae | *Planorbis* sp. | 1 |
| grazer_scraper |  |  |  |  | Gastropoda | NA | NA | 5 |
| omnivore | Coleoptera | Haliplidae | *Haliplus* sp. | 4 | Coleoptera | Haliplidae | *Haliplus* sp. | 34 |
| omnivore | Diptera | Chironomidae | NA | 33 | Diptera | Chironomidae | NA | 36 |
| omnivore |  |  |  |  | Diptera | Stratiomyidae | NA | 2 |
| omnivore | Heteroptera | Corixidae | *Sigara* sp. | 9 | Heteroptera | Corixidae | NA | 34 |
| omnivore | Heteroptera | Corixidae | NA | 22 |  |  |  |  |
| predator | Coleoptera | Dytiscidae | *Colymbetes* sp. | 4 | Coleoptera | Dytiscidae | *Colymbetes* sp. | 1 |
| predator |  |  |  |  | Coleoptera | Dytiscidae | *Hyphydrus* sp. | 2 |
| predator |  |  |  |  | Coleoptera | Dytiscidae | *Laccophilus* sp. | 1 |
| predator | Coleoptera | Dytiscidae | NA | 9 | Coleoptera | Dytiscidae | NA | 12 |
| predator | Diptera | Ceratopogonidae | *Phaenobezzia* sp. | 5 | Diptera | Ceratopogonidae | *Phaenobezzia* sp. | 3 |
| predator |  |  |  |  | Diptera | Ceratopogonidae | *Culicoides* sp. | 3 |
| predator | Diptera | Ceratopogonidae | NA | 3 | Diptera | Ceratopogonidae | NA | 5 |
| predator |  |  |  |  | Diptera | Chaoboridae | NA | 1 |
| predator | Diptera | Muscidae | *Lispe* sp. | 1 |  |  |  |  |
| predator | Heteroptera | Notonecidae | *Notonecta* sp. | 2 | Heteroptera | Notonecidae | *Notonecta* sp. | 49 |
| predator |  |  |  |  | Heteroptera | Pleidae | *Plea* sp. | 13 |
| predator |  |  |  |  | Heteroptera | Naucoridae | NA | 12 |
| predator |  |  |  |  | Hirudinea | Erpobdellidae | *Erpobdella* sp. | 11 |
| predator |  |  |  |  | Hirudinea | Glossiphoniidae | *Hemiclepsis* sp. | 1 |
| predator |  |  |  |  | Megaloptera | Sialidae | *Sialis* sp. | 9 |
| predator |  |  |  |  | Odonata | Aeshnidae | *Anaciaeschna* sp. | 2 |
| predator | Odonata | Aeshnidae | *Anax* sp. | 3 | Odonata | Aeshnidae | *Anax* sp. | 1 |
| predator | Odonata | Aeshnidae | *Brachytron* sp. | 1 | Odonata | Aeshnidae | *Brachytron* sp. | 3 |
| predator | Odonata | Coenagrionidae | *Coenagrion* sp. | 11 | Odonata | Coenagrionidae | *Coenagrion* sp. | 2 |
| predator | Odonata | Coenagrionidae | *Enallagma* sp. | 1 |  |  |  |  |
| predator |  |  |  |  | Odonata | Calopterygidae | *Calopteryx* sp. | 1 |
| predator | Odonata | Coenagrionidae | *Ischnura* sp. | 1 | Odonata | Coenagrionidae | *Ischnura* sp. | 3 |
| predator |  |  |  |  | Odonata | Coenagrionidae | *Erytromma* sp. | 1 |
| predator | Odonata | Coenagrionidae | *Phyrrosoma* sp. | 1 | Odonata | Coenagrionidae | NA | 26 |
| predator | Odonata | Coenagrionidae | NA | 8 | Odonata | Corduliidae/  Libelluidae | NA | 1 |
| predator | Odonata | Corduliidae/  Libelluidae | NA | 1 |  |  |  |  |
| predator |  |  |  |  | Odonata | Platycnemididae | *Platycnemis* sp. | 1 |
| predator |  |  |  |  | Odonata | Zygoptera | NA | 5 |
| predator | Trichoptera | Polycentropidae | NA | 1 |  |  |  |  |
| shredder | Crustacea | Asellidae | *Asellus* sp. | 13 | Crustacea | Asellidae | *Asellus* sp. | 30 |
| shredder |  |  |  |  | Crustacea | Gammaridae | NA | 26 |
| shredder | Diptera | Tipulidae | NA | 17 | Diptera | Tipulidae | NA | 3 |
| shredder |  |  |  |  | Lepidoptera | NA | NA | 2 |
| shredder | Trichoptera | Sericostomatidae | *Sericostoma* sp. | 1 |  |  |  |  |
