# Supplementary material for: Assessing agricultural effects on benthic invertebrate communities in ponds and ditches using δ¹⁵N and δ¹³C isotope niches
Source: PLoS One. 2025 Nov 24;20(11):e0336486. doi: 10.1371/journal.pone.0336486 (PMC12643296; doi:10.1371/journal.pone.0336486)
Supplement: S6 File — (DOCX) [file pone.0336486.s006.docx]

**Supporting information 6: Layman metrics for ponds and ditches, calculated for the FFGs and the different water bodies as grouping.**

|  | pond | ditch | pond | ditch | pond | ditch | pond | ditch | pond | ditch | pond | ditch |
| --- | --- | --- | --- | --- | --- | --- | --- | --- | --- | --- | --- | --- |
|  | **dY range** | | **dX range** | | **TA** | | **CD** | | **MNND** | | **SDNND** | |
| collector/filterer | NA | 3.61 | NA | 2.22 | NA | 0.00 | NA | 2.12 | NA | 4.24 | NA | 0.00 |
| collector/gatherer | 1.05 | 2.98 | 20.54 | 3.32 | 10.66 | 3.46 | 8.66 | 1.59 | 7.74 | 1.72 | 9.22 | 0.95 |
| grazer/scraper | 1.52 | 0.13 | 17.53 | 1.57 | 6.39 | 0.08 | 6.77 | 0.47 | 7.48 | 0.46 | 4.65 | 0.35 |
| omnivore | 2.39 | 0.36 | 21.44 | 3.89 | 24.24 | 0.28 | 9.09 | 1.14 | 8.61 | 1.14 | 9.14 | 0.62 |
| predator | 2.73 | 2.13 | 18.14 | 3.20 | 0.88 | 2.89 | 7.67 | 1.32 | 6.84 | 1.32 | 8.09 | 0.81 |
| shredder | 0.33 | 3.50 | 0.96 | 5.62 | 0.00 | 4.51 | 0.51 | 2.04 | 1.01 | 2.10 | 0.00 | 1.11 |
