# Supplementary material for: Assessing agricultural effects on benthic invertebrate communities in ponds and ditches using δ¹⁵N and δ¹³C isotope niches
Source: PLoS One. 2025 Nov 24;20(11):e0336486. doi: 10.1371/journal.pone.0336486 (PMC12643296; doi:10.1371/journal.pone.0336486)
Supplement: S7 File — Raw data was corrected with δ15N means of resources. (DOCX) [file pone.0336486.s007.docx]

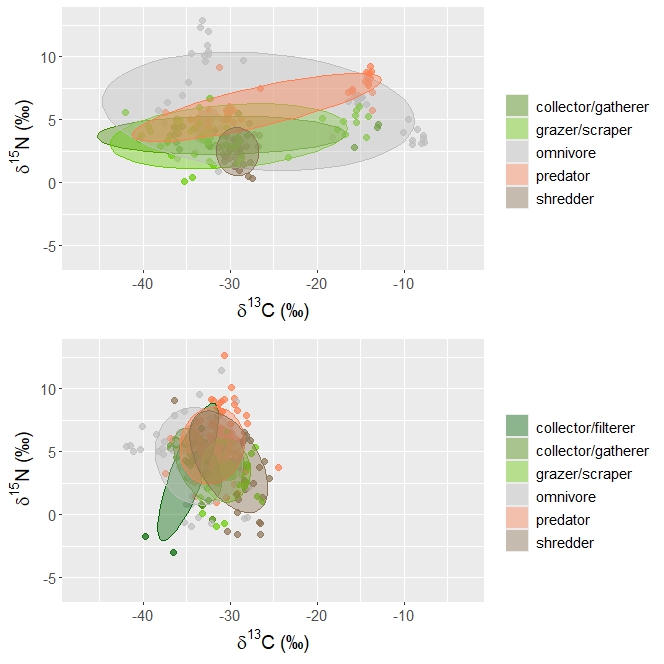


**Supporting information 7: Biplots for benthic macroinvertebrates in ponds (top) and ditches (bottom). Raw data was corrected with δ15N means of resources.**
