# Supplementary material for: Assessing agricultural effects on benthic invertebrate communities in ponds and ditches using δ¹⁵N and δ¹³C isotope niches
Source: PLoS One. 2025 Nov 24;20(11):e0336486. doi: 10.1371/journal.pone.0336486 (PMC12643296; doi:10.1371/journal.pone.0336486)
Supplement: S8 File — Raw data was corrected with δ15N means of resources and δ13C values were corrected for the influence of C4-plant maize. (DOCX) [file pone.0336486.s008.docx]

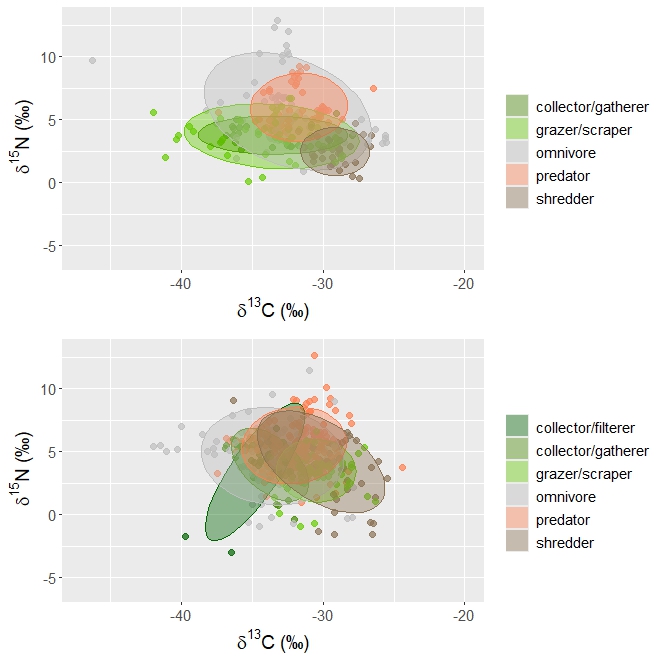


**Supporting information 8: Biplots for benthic macroinvertebrates in ponds (top) and ditches (bottom). Raw data was corrected with δ15N means of resources and δ13C values were corrected for the influence of C4-plant maize.**
