# Supplementary material for: Assessing agricultural effects on benthic invertebrate communities in ponds and ditches using δ¹⁵N and δ¹³C isotope niches
Source: PLoS One. 2025 Nov 24;20(11):e0336486. doi: 10.1371/journal.pone.0336486 (PMC12643296; doi:10.1371/journal.pone.0336486)
Supplement: Attachment — Submitted filename: PONE-D-25-03786_reviewer_13.3.2025.pdf [file pone.0336486.s009.pdf]

PLOS ONE

Assessing agricultural effects on benthic invertebrate communities in ponds and ditches using δ15N and δ13C isotope niches

--Manuscript Draft--

**Manuscript Number:**	PONE-D-25-03786
**Article Type:**	Research Article
**Full Title:**	Assessing agricultural effects on benthic invertebrate communities in ponds and ditches using δ15N and δ13C isotope niches
**Short Title:**	Assessing agricultural effects on benthic invertebrate isotope niches
**Corresponding Author:**	Stefan Lorenz<br>Julius Kühn Institute Berlin: Julius Kuhn-Institut Berlin<br>Berlin, GERMANY
**Keywords:**	agricultural landscape; benthic invertebrates; food web; isotope niche; small water bodies
**Abstract:**	Agriculture is one of the dominant types of land use worldwide. The conversion of land and intensification of agriculture have severe impacts on biodiversity and the associated ecosystem functions in the production areas and in natural or semi-natural habitats embedded in the agricultural landscape. Ponds and ditches, while vulnerable to the negative impacts of agriculture, are also valuable habitats for benthic invertebrates and harbor a diverse community as well as endangered or scarce taxa. It is challenging to detect community disturbance in these habitats due to stressors from agriculture, as shown by the failure of classic structural biodiversity metrics to perform well in previous studies. Therefore, we used a functional approach to understand the influence of agricultural stressors on benthic invertebrates. Benthic invertebrates and water samples were collected from three ponds and four ditches in an agricultural landscape in Brandenburg, Germany. The invertebrates were analysed for their stable isotope compositions of carbon and nitrogen, while the water samples were analysed for pesticide and nutrient residues. Standard ellipse areas and Layman.B community metrics were derived using a Bayesian approach for the two water body types and the six functional feeding groups (FFGs) present. A distance-based Redundancy Analysis was carried out to detect the environmental variables that show the maximum correlation with the derived metrics. Benthic invertebrates in ponds occupied a larger total area and showed greater spacing of individuals in the isotope space than communities in ditches, indicating that more resources and/or habitats were available and utilized in ponds. No clear pattern emerged when comparing the FFGs in the two water body types, but some unexpected differences were found, for example in FFG predator. Overall, the communities seem to be influenced on a case-by-case basis by various factors, either at the production or the consumption level. In the end, our study found that benthic invertebrate communities respond in complex ways to stressors in small unstable systems, which are also subject to strong fluctuations in biotic and abiotic parameters. However, larger datasets may yield in more pronounced patterns on effects of agriculture on stable isotope composition in ponds and ditches.
**Order of Authors:**	Fee Nanett Trau
	Kathrin Fisch
	Stefan Lorenz
**Additional Information:**	
**Question**	**Response**
**Financial Disclosure**<br><br>Enter a financial disclosure statement that describes the sources of funding for the work included in this submission. Review the submission guidelines for detailed	Yes

Powered by Editorial Manager® and ProduXion Manager® from Aries Systems Corporation

requirements. View published research articles from *PLOS ONE* for specific examples.<br><br>This statement is required for submission and **will appear in the published article** if the submission is accepted. Please make sure it is accurate.<br><br>**Funded studies**<br>Enter a statement with the following details:<br>• Initials of the authors who received each award<br>• Grant numbers awarded to each author<br>• The full name of each funder<br>• URL of each funder website<br>• Did the sponsors or funders play any role in the study design, data collection and analysis, decision to publish, or preparation of the manuscript?<br><br>Did you receive funding for this work?	
Please add funding details.<br>as follow-up to "**Financial Disclosure**<br><br>Enter a financial disclosure statement that describes the sources of funding for the work included in this submission. Review the submission guidelines for detailed requirements. View published research articles from *PLOS ONE* for specific examples.<br><br>This statement is required for submission and **will appear in the published article** if the submission is accepted. Please make sure it is accurate.	This work was funded by the German Federal Ministryof Food and Agriculture (BMEL) via the Agency for Renewable Resources (FNR; project number22012018) based on a decision of the Parliament of the Federal Republic of Germany.

**Funded studies**<br>Enter a statement with the following details:<br>• Initials of the authors who received each award<br>• Grant numbers awarded to each author<br>• The full name of each funder<br>• URL of each funder website<br>• Did the sponsors or funders play any role in the study design, data collection and analysis, decision to publish, or preparation of the manuscript?<br><br>Did you receive funding for this work?"	
Please select the country of your main research funder (please select carefully as in some cases this is used in fee calculation).<br>as follow-up to "**Financial Disclosure**<br><br>Enter a financial disclosure statement that describes the sources of funding for the work included in this submission. Review the submission guidelines for detailed requirements. View published research articles from *PLOS ONE* for specific examples.<br><br>This statement is required for submission and **will appear in the published article** if the submission is accepted. Please make sure it is accurate.<br><br>**Funded studies**<br>Enter a statement with the following details:<br>• Initials of the authors who received each award<br>• Grant numbers awarded to each author<br>• The full name of each funder<br>• URL of each funder website<br>• Did the sponsors or funders play any role in the study design, data collection and analysis, decision to publish, or preparation of the manuscript?<br><br>Did you receive funding for this work?"	GERMANY - DE
**Competing Interests**	The authors have declared that no competing interests exist.

Use the instructions below to enter a competing interest statement for this submission. On behalf of all authors, disclose any competing interests that could be perceived to bias this work—acknowledging all financial support and any other relevant financial or non-financial competing interests.<br><br>This statement is required for submission and **will appear in the published article** if the submission is accepted. Please make sure it is accurate and that any funding sources listed in your Funding Information later in the submission form are also declared in your Financial Disclosure statement.<br><br>View published research articles from *PLOS ONE* for specific examples.<br><br>**NO authors have competing interests**<br><br>Enter: *The authors have declared that no competing interests exist.*<br><br>**Authors with competing interests**<br><br>Enter competing interest details beginning with this statement:<br><br>*I have read the journal's policy and the authors of this manuscript have the following competing interests: [insert competing interests here]*<br><br>* typeset	
**Ethics Statement**<br><br>Enter an ethics statement for this submission. This statement is required if the study involved:<br><br>• Human participants<br>• Human specimens or tissue<br>• Vertebrate animals or cephalopods<br>• Vertebrate embryos or tissues	Invertebrate tissue was collected under field permissions granted by the FInAl project (fundeb by the Agency for Renewable Resources (FNR; project number22012018)).

Powered by Editorial Manager® and ProduXion Manager® from Aries Systems Corporation

• Field research<br><br>Write "N/A" if the submission does not require an ethics statement.<br><br>General guidance is provided below. Consult the submission guidelines for detailed instructions. **Make sure that all information entered here is included in the Methods section of the manuscript.**	

Powered by Editorial Manager® and ProduXion Manager® from Aries Systems Corporation

**Format for specific study types**<br><br>**Human Subject Research (involving human participants and/or tissue)**<br>• Give the name of the institutional review board or ethics committee that approved the study<br>• Include the approval number and/or a statement indicating approval of this research<br>• Indicate the form of consent obtained (written/oral) or the reason that consent was not obtained (e.g. the data were analyzed anonymously)<br><br>**Animal Research (involving vertebrate animals, embryos or tissues)**<br>• Provide the name of the Institutional Animal Care and Use Committee (IACUC) or other relevant ethics board that reviewed the study protocol, and indicate whether they approved this research or granted a formal waiver of ethical approval<br>• Include an approval number if one was obtained<br>• If the study involved *non-human primates*, add *additional details* about animal welfare and steps taken to ameliorate suffering<br>• If anesthesia, euthanasia, or any kind of animal sacrifice is part of the study, include briefly which substances and/or methods were applied<br><br>**Field Research**<br><br>Include the following details if this study involves the collection of plant, animal, or other materials from a natural setting:<br>• Field permit number<br>• Name of the institution or relevant body that granted permission	
**Data Availability**<br><br>Authors are required to make all data underlying the findings described fully available, without restriction, and from the time of publication. PLOS allows rare exceptions to address legal and ethical concerns. See the PLOS Data Policy and FAQ for detailed information.	Yes - all data are fully available without restriction

A Data Availability Statement describing where the data can be found is required at submission. Your answers to this question constitute the Data Availability Statement and **will be published in the article**, if accepted.<br><br>**Important:** Stating 'data available on request from the author' is not sufficient. If your data are only available upon request, select 'No' for the first question and explain your exceptional situation in the text box.<br><br>Do the authors confirm that all data underlying the findings described in their manuscript are fully available without restriction?	
**Describe where the data may be found in full sentences. If you are copying our sample text, replace any instances of XXX with the appropriate details.**<br><br>• If the data are **held or will be held in a public repository**, include URLs, accession numbers or DOIs. If this information will only be available after acceptance, indicate this by ticking the box below. For example: *All XXX files are available from the XXX database (accession number(s) XXX, XXX.*).<br>• If the data are all contained **within the manuscript and/or Supporting Information files**, enter the following: *All relevant data are within the manuscript and its Supporting Information files.*<br>• If neither of these applies but you are able to provide **details of access elsewhere**, with or without limitations, please do so. For example:<br><br>*Data cannot be shared publicly because of [XXX]. Data are available from the XXX Institutional Data Access / Ethics Committee (contact via XXX) for researchers who meet the criteria for access to confidential data.*<br><br>*The data underlying the results presented in the study are available from (include the name of the third party*	The raw data on the stable isotope composition (δ13C and δ15N) of the consumers will be made available at the IsoBank repository. The data have already been submitted and are still under processing by IsoBank. Final DOI and reference to the data set will be incorporated in the manuscript during review process if the manuscript is seen egligible for PLOS ONE.

and contact information or URL).<br>• This text is appropriate if the data are owned by a third party and authors do not have permission to share the data.<br><br>* typeset	
Additional data availability information:	Tick here if the URLs/accession numbers/DOIs will be available only after acceptance of the manuscript for publication so that we can ensure their inclusion before publication.

Powered by Editorial Manager® and ProduXion Manager® from Aries Systems Corporation

Click here to access/download;Manuscript;Manuscript_Trau_2025_isotope_

Titel: Assessing agricultural effects on benthic invertebrate communities in ponds and ditches using $\delta^{15}N$ and $\delta^{13}C$ isotope niches

Authors: Fee Nanett Trau [1*], Kathrin Fisch[1], Stefan Lorenz [1]

[1] Julius Kühn-Institut, Institute for ecological chemistry, plant analysis and stored product protection, Königin-Luise-Straße 19, 14195 Berlin, Germany

* Corresponding author:

E-Mail: fee-nanett.trau@julius-kuehn.de (FNT)

Abstract

Agriculture is one of the dominant types of land use worldwide. The conversion of land and intensification of agriculture have severe impacts on biodiversity and the associated ecosystem functions in the production areas and in natural or semi-natural habitats embedded in the agricultural landscape. Ponds and ditches, while vulnerable to the negative impacts of agriculture, are also valuable habitats for benthic invertebrates and harbor a diverse community as well as endangered or scarce taxa. It is challenging to detect community disturbance in these habitats due to stressors from agriculture, as shown by the failure of classic structural biodiversity metrics to perform well in previous studies. Therefore, we used a functional approach to understand the influence of agricultural stressors on benthic invertebrates. Benthic invertebrates and water samples were collected from three ponds and four ditches in an agricultural landscape in Brandenburg, Germany. The invertebrates were analysed for their stable isotope compositions of carbon and nitrogen, while the water samples were analysed for pesticide and nutrient residues. Standard ellipse areas and Layman.B community metrics were derived using a Bayesian approach for the two water body types and the six functional feeding groups (FFGs) present. A distance-based Redundancy Analysis was carried out to detect the environmental variables that show the maximum correlation with the derived metrics. Benthic invertebrates in ponds occupied a larger total area and showed greater spacing of individuals in the

isotope space than communities in ditches, indicating that more resources and/or habitats were available and utilized in ponds. No clear pattern emerged when comparing the FFGs in the two water body types, but some unexpected differences were found, for example in FFG *predator*. Overall, the communities seem to be influenced on a case-by-case basis by various factors, either at the production or the consumption level. In the end, our study found that benthic invertebrate communities respond in complex ways to stressors in small unstable systems, which are also subject to strong fluctuations in biotic and abiotic parameters. However, larger datasets may yield in more pronounced patterns on effects of agriculture on stable isotope composition in ponds and ditches.

Keywords: agricultural landscape, benthic invertebrates, food web, isotope niche, small water bodies

1. Introduction

Agriculture covers about 38 % of the world's terrestrial area, which makes it the predominant land use type (1). The conversion of forests, wetlands, and natural grasslands to agricultural land has had severe impacts on the provision of habitats, biodiversity, and associated ecosystem functions (1,2). Likewise, the increasing specialization of farmers and intensification of agriculture (e.g. by fertilization and pesticide application) further increased homogenization of the landscape, resulting in accelerated habitat fragmentation and biodiversity losses (3). Those developments continue to exert ongoing pressure on the remaining semi-natural or natural habitats within the agricultural landscape and on the surrounding aquatic and terrestrial environments (4).

Examples of remaining non-productive areas in the context of agriculture are water bodies such as ponds and ditches, which provide valuable habitats for aquatic and semi-aquatic species. The importance of particularly ponds, but also ditches, for benthic macroinvertebrate biodiversity and ecosystem services is increasingly gaining recognition in research activities (5–11). Ditches have the potential to host diverse communities as well as scarce or endangered aquatic species (9) and show a high richness of Coleoptera, Gastropoda, Trichoptera and Heteroptera species. Furthermore, ponds and ditches are biodiversity hotspots and form aquatic corridors in a landscape dominated by agriculture in need of conservation action (9,12). Unfortunately, these small water bodies have not received as much attention in ecological research as they deserve (9,13) and are usually not included in monitoring programs (9,14).

To conserve and promote these aquatic habitats and their biodiversity, knowledge on the status and indicators for measuring biodiversity are indispensable. However, the assessment of benthic invertebrate biodiversity is challenging, and results from previous studies on ponds have shown that conventional structural biodiversity metrics (e.g. taxonomic diversity, Shannon index, and the EPT index, to a certain degree) do not perform well in detecting stressed communities in agricultural areas due to long-term and ubiquitous persistence of stressors and short stressor gradients (8). Therefore, functional indicators and approaches could be promising tools to detect community disturbance and

to examine possible direct as well as indirect effects of stressors on benthic invertebrate communities and their functions (8,15,16). Common traits used to assess the functional diversity of benthic invertebrates are feeding mode, locomotion, maximum body size, and respiration (17). As benthic invertebrates play an important role in the cycling of nutrients and decomposition of organic matter, the analysis of trophic relationships and functional feeding groups (FFGs) are central to understanding ecosystem processes (18,19) and to visualizing effects of agricultural activity on communities (15,20–22).

Stable isotope studies are a powerful tool for understanding trophic relationships and community use of resources and habitats. The basic principle of "you are what you eat (plus a few ‰)" (23) makes it possible to use stable isotopes to analyse isotopic niches, food web structures, and interactions between species in ecosystems (24). Two elements commonly used for this purpose are nitrogen and carbon. The ratio of ^{15}N to ^{14}N enriches stepwise along the trophic levels whereas the ratio of ^{13}C to ^{12}C remains almost unchanged when passing through the food web, but discriminates food sources due to different photosynthetic pathways (23). Accordingly, $\delta^{15}N$ values are used to determine the trophic position of an organism and $\delta^{13}C$ values provide information on the sources utilized by the organism.

Based on this information, the isotopic niche of a species or taxa group can be quantified and community metrics derived, which are statistically comparable over a broad spatial scale and between communities composed of different species (16,25).

The niche concept in community ecology is fundamental to understanding how communities are organized and how ecological interactions shape their strategies and ecological shifts (26). The niche of a species describes the range of conditions necessary for the species to exist and reproduce, including its abiotic and biotic interactions with the environment (27). Niches can overlap when species are co-occurring, which leads to interspecific competition. Higher species richness can lead to greater niche overlaps, as for example in carabid communities (28). For a detailed review of niche concepts and developments during the 20th century see (26) and for examples on the further development of the `old` niche concepts (in the early 2000s) see (29) and (27).

In this study we will use niche in the sense of the isotopic niche, which can serve as a proxy for the realized ecological niche in relation to the use of resources and habitat (29). We will compare niche widths of groups and communities based on a geometric approach originally developed by (22) to examine the isotopic niches of benthic macroinvertebrates in ponds and ditches, as well as to assess the influence of agricultural stressors (e.g. pesticide residues) on the isotopic niche metrics.

We ask the following questions, using ponds and ditches as an example: Can the isotopic niches depict the effects of agriculture on benthic invertebrate communities? Are the observed patterns comparable in ponds and ditches for the FFGs?

In pursuit of answers to these questions, we hypothesize that agricultural activity and low habitat variability in ditches have led to simplified trophic structures, which will be shown by wider isotopic niches, reduced niche differentiation, and lower niche overlap due to reduced specialisation of the FFGs.

2. Methods and data

2.1 Study area

Sampling was carried out in Brandenburg in the “Havelländisches Luch” region (Fig 1). During the time of the German Democratic Republic until the 1980s, the wet lowlands of this area were drained and an intensively maintained ditch system was created for industrial agricultural production (30). Traditional grasslands were converted to seeded grasslands, often with only one type of grass, and in some cases also plowed into arable land (30). Additionally, the landscape is characterized by a large number of small standing water bodies (kettle holes, consistently named “ponds” within the context of this paper), which were formed during the last glacial period. Ponds are defined as water bodies with a maximum extent of one hectare and cover about 5 % of the arable land over a total area of 38.000 km^2 in northeast Germany (31).

Fig 1: Map of sampling locations. Map data: Google satellite, QGIS Version 3.34.3

Samples were taken in four ditches (Fig 2) and three ponds (Fig 3) in October and November 2020. Three of the four ditches were dominated by grasses in the riparian zone. Ditch 1 was dominated by

reeds. Ponds 2 and 3 are characterized by a mix of trees, shrubs, grasses, and reeds in the riparian zone, while pond 1 is entirely encompassed with trees, shrubs, and reeds. See also Table 1 for descriptions of the water bodies and their surroundings.

Table 1: Description of the sampling sites. CPOM = coarse particulate organic matter; FPOM = fine particulate organic matter

			Riparian zone [in %]				
**Site**	**Habitats**	**Agricultural cultivation**	**Grasses**	**Herbaceous perennials**	**Trees**	**Shrubs**	**Reeds**
**Pond 1**	Reeds, CPOM, macrophytes	Grassland	0	0	25	25	50
**Pond 2**	Reeds, CPOM, macrophytes	Grassland	30	0	5	5	60
**Pond 3**	Reeds, CPOM, FPOM	Maize, cereal	0	20	10	10	60
**Ditch 1**	Reeds, FPOM	Grassland	20	0	0	0	80
**Ditch 2**	CPOM, macrophytes	Grassland	100	0	0	0	0
**Ditch 3**	Reeds, CPOM, FPOM	Grassland	80	0	0	0	20
**Ditch 4**	Reeds, FPOM, macrophytes	Grassland	100	0	0	0	0

Fig 2: Sampled ditches, pictures: Fee Nanett Trau, JKI

Fig 3: Sampled ponds, pictures: Fee Nanett Trau, JKI

2.2 Sampling and sample processing - invertebrates

Benthic invertebrates from the different FFGs present in the selected water bodies were sampled via hand netting (mesh size 500 µm) from present habitats, such as FPOM, CPOM, submerse macrophytes, and reeds. Organisms were sorted on-site by taxonomy (to the level of order or family) and feeding groups. Sampling was carried out until 10 specimen of the present orders were collected as recommended by (25), whenever possible. All animals were kept alive for about 24 hours and then humanely dispatched with boiling water. In the laboratory, the benthic macroinvertebrates were taxonomically determined to family or genus level when possible. Gastropods, Bivalves, and

Trichoptera were removed from their shell or caddis and parasites were removed from afflicted Heteropterans before further sample processing. The samples were dried (48 hours at 60 °C, Memmert UFE 600). The individual organisms were stored in Eppendorf tubes (1.5 ml). If the animal weight was >2 mg (Sartorius LA 230 P), the individual was ground into a powder. For lower sample weights, individuals were pooled to reach the optimum sample weight of 1 mg and then ground. Material was homogenized using a mixer mill (Retsch MM400) and stored in Eppendorf tubes.

The encapsulation as well as the isotope analysis was carried out by the stable isotope laboratory at Cornell University (COIL), Department of Ecology and Evolutionary Biology, USA. Stable isotope analysis of ^{15}N and ^{13}C was performed using a Thermo Delta V isotope mass spectrometer (IRMS) interfaced to a NC2500 elemental analyser. Quality control values for data can be obtained here (insert reference to IsoBank repository, status in submission). Isotope corrections were performed using linear regression of all δ values using two additional in-house standards (32). Results were reported as δ values as ratios of ^{13}C:^{12}C relative Vienna PeeDee Belemnite and ^{15}N:^{14}N relative to atmospheric air (Eq. 1). Measurements were carried out from April 2021 to July 2022 in four batches.

$$\delta X = \left[\left(R_{sample}/R_{standard}\right) - 1\right] * 10^3 \quad (1)$$

Where X is ^{13}C or ^{15}N, R is the corresponding ratio ^{13}C /^{12}C or ^{15}N /^{14}N. Increased δ values show an increase of the heavy isotope in the sample. ~~Taken from~~ (33).

2.3 Sampling and sample processing - water

Physical and chemical parameters were measured to identify potential variables that could impact food web structures or isotopic niches. Water samples were taken via grab sampling. A mixed water sample of approximately 5 L was taken from four to six locations around the shoreline of the ponds or along a stretch of 50 m from the ditches.

Physicochemical water parameters [oxygen content, electrical conductivity (EC), pH, and temperature] were measured in-situ using a portable meter (WTW Multi 3430 IDS with OxiCal-SL FDO 925, TetraCon 925 and SenTix 940 probes, Germany). Samples for pesticide analysis were partitioned into two 500 mL

glass bottles, with one bottle holding 50 mL of dichloromethane for pyrethroid analysis. The water samples were analysed using multi-methods validated for analysis of 99 active substances (S1 Table).

Except for the pyrethroids, the selected active substances in the water samples were quantified by liquid chromatography mass spectrometry (LC-ESI-MS/MS) using the internal standard method after enrichment and clean-up via solid phase extraction (Chromabond HR-P, Macherey-Nagel, Germany, 50-100 µm, 3 mL, 200 mg). For LC-MS/MS analysis a 1290 Infinity II LC system (Agilent, USA) coupled to a QTRAP 6500+ mass spectrometer (SCIEX, USA) was used.

After concentration by liquid–liquid extraction of the sample, pyrethroids were analysed by gas chromatography-mass spectrometry (GC-MS) using a Trace GC Ultra coupled to a TSQ Quantum GC XLS- mass spectrometer (Thermo Electron Corporation, USA). All pesticide analysis were done accordingly to (8).

Samples for nutrient analysis were filtered through a 0.45 µm cellulose acetate filter before analysis. The concentrations of NH_4^+ + and PO_4^{3-}were measured photometrically using a spectrophotometer (DR/2000 Hach) according to DIN 38 406–5 and EN ISO 6878:2004, respectively. NO_3^- and NO_2^- concentrations were analysed by high-performance liquid chromatography (HPLC, Agilent 1100 Series HPLC System DAD, USA).

2.3.1 Toxicity calculations

The toxic pressure for each sample is calculated as the sum of toxic units; TU_{sum} (Eq. 2). Toxic units were calculated for the pesticide concentrations based on half-maximal effective concentration (EC50) or 50% lethal concentration (LC50) values for invertebrates, macrophytes, and algae. Analyzed pesticide concentrations were divided by the concentration for the most sensitive test organism and based on 48 h EC50 values for *Daphnia magna* or 96 h LC50 values for *Chironomus sp.* for the invertebrate toxicity. Toxicity calculations for macrophytes are based on 7-day EC50 values for *Lemna gibba* and *Lemna minor* and for algae on 72 h EC50 values for mostly*, Raphidocelis subcapitata (*syn: *Pseudokirchneriella subcapitata*) and *Scenedesmus subspicatus*. Data was obtained from the Pesticides

Properties Database (34) or Ecotox database (35). See also S1 table for details on substances measured and the test organisms.

$$TU_{sum} = \log \sum_{i=1}^{n} \left(\frac{C_i}{EC_{50_i}/LC_{50_i}}\right) \quad (2)$$

C_i is the concentration of pesticide *i* in µg/L. Concentrations below the LOQ but above 0 were replaced with half of the LOQ.

2.4 Description of the dataset

The dataset contained $\delta^{13}C$ and $\delta^{15}N$ data from 13 orders covering six FFGs (Table 2). FFGs were assigned to the individuals according to the family determination, if available, otherwise by order (36–39). The dataset contained values for 22 environmental parameters measured (Table 3).

Table 2: Sample sizes.

FFG	Pond 1	Pond 2	Pond 3	Ditch 1	Ditch 2	Ditch 3	Ditch 4	Sum FFG pond/ditch
**Collector/ filterer**	0	0	0	3	0	0	9	**0/12**
**Collector/ gatherer**	13	5	3	11	10	10	10	**21/41**
**Grazer/ scraper**	37	8	11	16	14	18	16	**56/64**
**Indifferent**	15	31	22	28	23	30	25	**68/106**
**Predator**	19	19	14	38	45	26	60	**52/169**
**Shredder**	24	7	0	18	21	10	12	**31/61**
**Sum type**	**Ponds = 228**				**Ditches = 453**			

Table 3: Overview of the measured water quality and environmental parameters for the seven water bodies. EC = electric conductivity, CPOM = coarse particulate organic matter, FPOM = fine particulate organic matter

Sample	C °	EC [µS/cm]	pH	O_2 [mg/L]	NH_4^+ [mg/L]	NO_2^- [mg/L]	NO_3^- [mg/L]	PO_4^{3-} [mg/L]	NH_4^+ NO_2^- $+NO_3^-$ [mg/L]	TUsum algae	TUsum plants	TUsum inverte-brates	Algae total [µg/l]	Cyano-bacteria [µg/l]	Green algae [µg/l]	Diatoms [µg/l]	Crypto-phyceae [µg/l]	Photo-synthetic activity [%]	Reeds [%]
Pond 1	7.4	1570	6.2	5.2	0.01	0	0.2	0.25	0.21	-2.686	-4.602	-5.221	28.85	0	17.07	4.39	7.39	49.34	50
Pond 2	10.5	1112	6.5	10.6	0.09	0	0.14	0.05	0.23	-4.057	-5.205	-5.074	112.3	0	63.68	48.31	0.35	52.03	60
Pond 3	11.2	881	7	13.8	0.08	0	0.23	0.11	0.31	-2.650	-4.216	-4.632	468.8	241.55	0	0	227.27	0.56	60
Ditch 1	9.7	588	7	11.5	0.86	0	1.1	0.44	1.96	-2.685	-4.849	-5.428	41.25	0.74	7.35	31.73	1.43	62.39	80
Ditch 2	8.3	815	7.6	6.1	0.08	0	0.72	0.06	0.8	-3.253	-3.512	-5.116	40.98	0.79	6.9	29.99	3.3	60.25	0
Ditch 3	11	928	7	8.8	0.08	0	0.36	0.06	0.44	-2.614	-3.930	-4.817	78.27	2.05	25.75	42.66	7.81	58.63	20
Ditch 4	12.7	900	8	8.8	0.08	0	1.32	0.03	1.4	-3.456	-3.641	-4.925	5.7	0	1.22	0.88	3.6	52.15	0

2.5 Statistical methods

All data processing and statistical analyses were performed with R Studio version 4.3.1 (40) and JAGS version 4.3.1.

2.5.1 $\delta^{15}N$ correction

The influence of organic fertilizer was visible in ditches by examining the $\delta^{15}N$ values of benthic macroinvertebrates (consumers). The $\delta^{15}N$ values for ditches are higher than in ponds due to higher $\delta^{15}N$ values in resources present in these water bodies (S2 Fig.). To compare the FFGs in the two water body types this was corrected as follows: For both water body types the mean $\delta^{15}N$ value for the consumers was calculated and its difference was subtracted from the $\delta^{15}N$ values of the consumers in ditches only.

2.5.2 $\delta^{13}C$ correction

The $\delta^{13}C$ values for one pond are higher due to the influence of maize cultivation (C4-plant) on the consumers (Fig. 4). For ponds 1 and 2 (no maize influence) and pond 3 (maize influence) the mean values for $\delta^{13}C$ consumers were calculated and its difference was subtracted from $\delta^{13}C$ values from consumers in pond 3 only.

The dataset with corrected $\delta^{15}N$ and $\delta^{13}C$ values was used for the comparison of FFGs using the corrected Standard Ellipse Area (SEA.c). The calculation of the Bayesian Layman metrics (Layman.B) and the distance-based Redundancy Analysis (dbRDA) were performed for the data set with corrected $\delta^{15}N$ values. Both versions were calculated to visualize the benthic invertebrate data in a biplot and to compare niche overlaps for the FFGs.

2.5.3 SIBER (Stable Isotope Bayesian Ellipses in R)

The R package SIBER (41) was used for the analysis of isotopic niche width of the FFGs and the comparison of food web structures between different water body types. The analysis follows a Bayesian approach to overcome uncertainties when calculating and comparing metrics (41). The estimation by Bayesian inference incorporates error from the sampling process and allows robust comparisons between groups with different and small sample sizes (25).

First the raw data was plotted for the six FFGs (*collector/filterer, collector/gatherer, grazer/scraper, indifferent, shredder,* and *predator*) and the respective water body types (S4 Fig.). Then the Layman metrics and the Bayesian inference-derived Layman.B metrics were calculated for the combination of water body and FFGs as group (37 groups) and the water body types pond and ditch as community as well as for the FFG as group (six groups) and the water body types pond and ditch as community. For the dbRDA (see below), the Layman metrics for the seven water bodies as community and FFG as group (six) were calculated.

The described metrics are based on (22) and summarize information on the distribution of data points in the isotope space. The six metrics are the $\delta^{13}C$ range, the $\delta^{15}N$ range, the total area of the convex hull (TA), the mean distance to centroid (CD), the mean nearest neighbor distance (MNND) and the standard deviation of the nearest neighbor distance (SDNND) (25). The TA describes the niche width, CD is giving information on niche width and species spacing, the MNND is a description of density and clustering of species, and the SDNND gives information on the evenness of the species density (25).

Additionally, the Bayesian standard ellipse areas (SEA.B), corrected standard ellipse areas (SEAc), and convex hull area (TA) based on Bayesian interference were calculated for the FFGs in ponds and ditches. The $\delta^{15}N$ and the $\delta^{13}C$ values were corrected for the influence of organic fertilization and the influence of the C4-plant maize in order to focus on the influence of the FFGs on the ellipse area. The data was plotted using the function *siberDensityPlot* (42).

The probability that the posterior distribution is smaller or larger for a group can be estimated by calculation of the SEA.B niche overlap. SEA.B overlaps between ponds and ditches were calculated for the FFGs based on the maximum likelihood estimates of the means and the covariance matrices of each group (function: *maxLikOverlap* calculated for the proportion of both overlaps, p-interval of 0.95).

Bayesian models were run with JAGS and model parameters were defined according (25) to fit the ellipses.

2.5.4 Multivariate statistics

Environmental variables were standardized using the *scale* function of the *base* package (43), whereby the mean value was set to zero and scaled to unit variance. Variable selection for the dbRDA was carried out by the *bioenv* function from the *vegan* package (44). This function detects the best environmental variable subset in a way such that the environmental variables show the maximum rank correlation (method: Spearman) with the Layman metrics matrix. The dissimilarity index for the Layman metrics matrix used was "mahalanobis", a measure that accounts for correlations across multiple variables. Selected environmental variables, excluding the variables EC and O_2 due to small sample size and variability of measures during the course of the day, were used for the dbRDA, which can show if an explanatory variable has significant impact on the response variables and how much of the variation is explained by each axis. (function *dbrda*, package *vegan*, 44). Selected variables are: nutrient concentration sum, number of pesticide detections, TU_{sum} for algae, and TU_{sum} for invertebrates. To test for the significance of the model, a permutation test on terms and axes was carried out ($p \leq 0.05$; function *anova.cca*, package *vegan*, 44).

3. Results

The raw data on the stable isotope composition ($\delta^{13}C$ and $\delta^{15}N$) of the consumers and sampled resources are available at IsoBank repository (in submission).

3.1 Isotopic niches of communities in ponds and ditches

After correction for the influence of organic fertilizers and of maize, the $\delta^{13}C$ and $\delta^{15}N$ values of consumers show a similar range in ditches and in ponds (Fig. 4 and S2 Fig.). The biplot shows that ponds have a slightly larger ellipse area but that the water body types are comparable in general (Fig. 4).

Fig 4: Biplots for the macroinvertebrate data. The left plot is corrected for $\delta^{15}N$ influence of organic fertilizers on ditches. The right plot is corrected for $\delta^{15}N$ as well as $\delta^{13}C$ values for the influence of C4-plant maize on one pond. Ellipses contain 40% of the data.

The probable mode of the $\delta^{15}N$ range is comparable between the two water body types (after correction for organic fertilization) (Fig. 5 a). The probable mode of the range of $\delta^{13}C$ is clearly larger in ponds (Fig. 5 b). The probable distribution of TA and thus total niche width and the spacing of individuals - CD is larger in pond communities compared to communities in ditches (Fig. 5 b, e). The

probable values for the MNND and thus clustering of individuals within the community is larger in ponds, where the evenness of density is similar in ponds and ditches (Fig. 5 e, f).

Fig 5: SIBER density plots for the Layman metrics based on the dataset corrected for δ^{15}N. Black dots show the modes. Shaded boxes represent the 50 %, 75 %, and 95 % confidence intervals, from dark to light red/blue. Grouping is based on individual small water body (SWB) and FFG (in total 37 groups), the communities are assigned to the SWB types (pond, ditch). δ^{15}N values of the ditches are corrected for the influence of organic fertilizers.

When the maize influence is corrected, the δ^{13}C values are comparable between water body types (Fig. 4), but the δ^{15}N values are slightly larger in ponds. For an illustration of the Layman metrics calculated for both stable isotopes corrected, see also table S3.

3.2 Comparison of functional feeding groups

There are clear differences for the SEA.B, but there is no clear pattern visible when comparing FFGs in ponds and ditches. SEA.B and SEAc modes for *shredders* are higher in ditches than in ponds (Fig. 6). Values for *predators* and *collector/gatherers* are similar in the two water body types (Fig. 6). The credible intervals for *predators* are rather small, which shows that the uncertainty is low and that the dataset provides a precise representation of the population mean. The FFG *predator* was in general more abundant and diverse in ditches, where they form six taxa groups and 13 families and were recorded with a mean abundance of 42.25 individuals, whereas in ponds they form five taxa groups and eight families and were recorded with a mean abundance of 17.3 individuals (S5 Table). The groups *indifferent* and *grazer/scraper* have larger SEA.B/SEAc modes in ponds (Fig. 6).

Fig 6: SIBER density plots computed via Bayesian analysis using corrected δ^{15}N and δ^{13}C values. Plots are shown for the FFGs in ponds (red) and ditches (blue). Black dots are the modes of SEA.B. The grey X indicates the size-corrected standard ellipse area (SEAc) mode. Shaded boxes represent the 50 %, 75 %, and 95 % confidence intervals.

SEA.B niche overlaps for the FFGs between ponds and ditches vary between 15 and 22.9 % (corrected only for δ^{15}N) and between 22.9 to 44.7 % when the data is corrected for δ^{15}N and δ^{13}C (Table 4). For *shredders* the overlap is the same for both correction approaches, as there were no *shredders* found in the maize pond and thus no δ^{13}C correction was needed. For the corrected for δ^{15}N and δ^{13}C values, niche overlaps for *predators* (44.7 %) are largest in ponds and ditches. *Shredders* show least similarity in niches between ponds and ditches (22.9 %).

Table 4: Niche overlaps of ponds and ditches for the FFGs calculated for two datasets. The overlap for collector/filterer *cannot be calculated due to their absence in pond samples.*

Functional Feeding Group	Niche overlaps of ponds and ditches in %, corrected for N values	Niche overlaps of ponds and ditches in %, corrected for C and N values
Collector/gatherer	15	28.4
Grazer/scraper	16.9	29.8
Indifferent	18.6	38.6
Predator	20	44.7
Shredder	22.9	22.9

3.3 Effects of agricultural stressors on the observed results

Water quality parameters measured in the water samples indicate low numbers of pesticides in ponds and high numbers of pesticides in ditches. Mean value equals 2.7 ±1.5 µg/L pesticides detected in ponds and 6 ± 1.4 µg/L pesticides detected in ditches. 81 % of the pesticides detected overall (ponds and ditches) are herbicides and 19 % are fungicides. Furthermore, nutrient concentration sum is larger in ditches (mean value of 1.15 ±0.7 mg/L) compared to ponds (mean value of 0.25 ±0.05 mg/L). Dissolved oxygen concentrations vary from 5.2 mg/L to 13.8 mg/L in ponds and from 6.1 mg/L to 11.5 mg/L in ditches. EC varies from 881 to 1570 µS/cm in ponds and from 588 to 928 µS/cm in ditches.

The dbRDA plot does not show a clear pattern for ponds and ditches (Fig. 7); the water bodies are influenced individually by different parameters. However, isotopic composition of benthic invertebrates and the resulting Layman metrics seem to be either influenced by the toxicity of water samples for invertebrates (TUsum_invert) or by a combination of the toxicity of water samples for algae (TUsum_algae), the concentration of nutrients (nutrients_sum), and the number of pesticides found (N_pesticides). Constrained variables at the first axis explain 75 % of the variance. However, the permutation test resulted in no statistical difference ($p \leq 0.05$) for the tested model, model parameters, or axes.

Fig 7: dbRDA plot, distance measure = Euclidean. Water bodies are plotted according to the Layman metrics calculated for the stable isotope results of the communities.

4. Discussion

The two water body types were in general comparable after the correction of $\delta^{15}N$ for the influence of organic fertilizer, which was clearly visible in consumers in ditches. Such relative enrichment was also

found by (45) in urban and agricultural streams compared to woodland streams. However, the effects of agriculture on benthic invertebrate communities could not be depicted by the isotopic niches. Also, the comparison of the isotope niches of the two water body types, pond and ditch, did not reveal any clear patterns when analysing the FFGs.

The hypothesis that we expect simplified trophic structures in ditches due to agricultural activity and low habitat variability can only be partially confirmed. The finding that the likely distribution of total niche width and distances between the individuals is greater in pond communities than in ditch communities indicates that more resources/habitats are available in ponds, which lead to more extreme isotope ratios. Furthermore, consumers are more divergent in ponds in terms of the isotopic niche MNND, while the evenness of density SDNND is similar in ponds and ditches. This indicates less clustering in pond communities at the same evenness level, which in turn indicates more available resources and/or habitats in ponds that were evenly utilized and colonised.

Even though there were no clear patterns visible when comparing FFGs, some differences were pronounced. Contrary to our expectations, the FFG *predator* was more abundant and diverse in ditches. Therefore, functional redundancy for this feeding type can be assumed. The high abundance and diversity of *predators* in ditches could be explained by the availability of a high number of primary producers facilitated by high nutrients inputs and thus high primary production, which leads to more available prey. The influence of changes on the base of the food web propagated to higher trophic levels was also observed by (46),where nutrient pollution promoted biofilm production and lead to an increase in herbivory and carnivory by omnivores. However in contrast to results from (46), the nutrient pollution in ditches did not lead to more homogenous communities for the FFG *predator* in ditches compared to ponds, instead the opposite is the case. In ditches, the number of recorded families belonging to the group of predators is nearly twice as high compared to ponds. The high secondary production is probably stimulated by bottom-up effects of nutrient enrichment (45,47). Although we can assume that the ditches have been enriched for several years, the effects of long-term enrichment predicted by (47), namely the reduction in secondary production and the shift in

community structure, were not observed in the ditch system studied when we compare it to the ponds. This positive influence of fertilization might be explained by intermediate but not high nutrient enrichment, as in the case of (48), which found highest densities of invertebrates at intermediate levels of nutrient enrichment (primarily of phosphate).

The *indifferent* FFG expressed a variety of feeding strategies. In pond 2 they were clearly mostly predatory with some individuals forming a cluster with the highest $\delta^{15}N$ values in that water body (S 4 Table). In the ditches 2, 3 and 4 they were predatory as well but $\delta^{15}N$ values were on the same level as the values of the predators. Some individuals classified as indifferent showed very low $\delta^{15}N$ values which indicates an herbivorous lifestyle. Clusters on the upper and lower level of $\delta^{15}N$ values are visible in four water bodies (two ditches and two ponds) which shows the shift of omnivores towards either herbivorous or predatory lifestyles as stated by (46). However, according to (46), this shift occurs in polluted water bodies, thus also the ponds probably show signs of communities under the influence of agricultural stressors.

The FFG *collector/gatherer* was dominated by ephemeropterans (Cloeon *sp.*) in both water body types which explains the similarity of SEA.c modes in the water body types and shows that this FFG is similar in its resource use in both water body types.

The larger SEA.c mode for FFG *grazer/scraper* in ponds could be explained by a wider habitat and/or resource base available in ponds, which had a more diverse habitat in and around the water body compared to the ditches, which were dominated by grasses. The lower diversity of littoral habitats was also found to be associated with lower numbers of trophic links and less resource and consumer diversity by (49) for lake ecosystems. This would mean that the nutrient-rich and thus primary producer-rich ditches are not diverse in resources but that some resources are very abundant. Additionally, the wider habitat and/or resource use can also be explained by higher taxa richness and the associated possible different preferences in ponds. Two *grazer/scraper* taxa groups and five families were found in ponds, but only one *grazer/scraper* taxa group and three families in ditches.

The absence of a clear pattern is also underlined by the dbRDA. The configuration of the water bodies shows that they are influenced on a case-by-case basis by the measured parameters, and other factors were not detected. In general, the composition of benthic invertebrates and the resulting Layman metrics appear to be influenced either at the production level (indicated by the variables nutrients sum, toxicity for algae, and number of pesticides detected (mainly herbicides)) or at the consumer level (indicated by the toxicity for invertebrates). Ponds 2 and 3 are similar to each other and are largely influenced on the consumer level. The four ditches and pond 1 are more strongly influenced at the primary producer level, so bottom-up effects are also probably stronger here.

Small and uneven sample sizes are principally of disadvantage, but the Bayesian statistic approach with the SEA.c should enable an unbiased, robust analysis (25). Nonetheless, the FFGs *indifferent* in ponds and *collector/filterer* in ditches show wide credible intervals that indicate a high degree of opportunism and variability in their feeding strategies and further sampling would be needed to predict modes with less uncertainty.

Additionally, the time of sampling (autumn) has to be taken into account and has most probably influenced the detection of taxa and pesticides. Information on community composition may have been skewed toward a less diverse composition, with lower numbers of especially Ephemeroptera, Trichoptera, and Dipterans. Although higher numbers of pesticides were found in ditches compared to ponds, the lack of a strong toxicity gradient for algae, invertebrates, and water plants made the analysis challenging. This should be adjusted in further studies, and sampling between the months of April and June is recommended.

Constrained variables at the first axis of the dbRDA explained 75 % of the variance. However, the permutation test resulted in no statistical difference ($p > 0.05$) for the tested model, model parameters, or axes. The high explanatory power of the constrained variables on the first axis most likely resulted from running the model on a small dataset and with a high number of explanatory variables.

5. Conclusion

With the obtained dataset, we were unable to recognize clear patterns of the effects of agriculture on isotopic niches of the benthic invertebrates. Agricultural activity and a homogenous environment did not lead to simplified structures in the ditches compared to ponds, but it must be assumed that the pond communities are also already influenced by agricultural activity and lack the image of a "realistic" control community. Ecological systems respond in complex ways to the presence of abiotic and biotic stressors. Particularly small systems are unstable and exposures are often unclear, leading to ambiguous responses and uncertain patterns. The individual water bodies are influenced by different parameters, and the impacts of individual structures within each water body are more pronounced than influences based on the type of water body, at least for the dataset present. Larger dataset are promising options to overcome this issues, which to our knowledge are not available for small water bodies in the agricultural landscapes in Europe. Additionally, selected water bodies should be monitored more closely and analysed on a broader temporal scale to detect potential effects of agriculture on benthic invertebrate communities over time.

Acknowledgments

The authors thank Marlen Heinz, Manuel König, Karin Meinikmann, Maren Stockmann, and Elke Zeidler for their assistance during the field sampling campaign. Furthermore, we thank Dominique Conrad, Michael Glitschka, Christine Reichmann, Ina Stachewicz, and Gabrielle Smykalla for the sample preparation and pesticide and nutrient analysis. In addition, we thank Dominique Conrad and Jörg Stauch for their help in preparing the biological samples. Thanks also to Matthias Stähler for discussions on analytical methods and Mario Brauns, Kristin Scharnweber, René Sahm, and Matthias Pilecky for fruitful discussions on data analysis. Furthermore, we thank Holly Blevins for proofreading.

References

1. Foley JA, Ramankutty N, Brauman KA, Cassidy ES, Gerber JS, Johnston M, et al. Solutions for a cultivated planet. Nature 2011;478(7369):337–42. PubMed PMID: 21993620.
2. Tscharntke T, Klein AM, Kruess A, Steffan-Dewenter I, Thies C. Landscape perspectives on agricultural intensification and biodiversity – ecosystem service management. Ecol Lett 2005;8(8):857–74.
3. Matson PA, Parton WJ, Power AG, Swift MJ. Agricultural intensification and ecosystem properties. Science 1997;277(5325):504–9. PubMed PMID: 20662149.
4. Stoate C, Báldi A, Beja P, Boatman ND, Herzon I, van Doorn A, et al. Ecological impacts of early 21st century agricultural change in Europe--a review. J Environ Manage 2009;91(1):22–46. PubMed PMID: 19717221.
5. Williams P, Whitfield M, Biggs J, Bray S, Fox G, Nicolet P, et al. Comparative biodiversity of rivers, streams, ditches and ponds in an agricultural landscape in Southern England. Biological Conservation 2004;115(2):329–41.
6. Davies BR, Biggs J, Williams PJ, Lee JT, Thompson S. A comparison of the catchment sizes of rivers, streams, ponds, ditches and lakes: implications for protecting aquatic biodiversity in an agricultural landscape. Hydrobiologia 2008;597(1):7–17.
7. Darwall W, Bremerich V, Wever A de, Dell AI, Freyhof J, Gessner MO, et al. The Alliance for Freshwater Life : A global call to unite efforts for freshwater biodiversity science and conservation. Aquatic Conserv: Mar Freshw Ecosyst 2018;28(4):1015–22.
8. Trau FN, Fisch K, Lorenz S. Habitat type strongly influences the structural benthic invertebrate community composition in a landscape characterized by ubiquitous, long-term agricultural stress. Inland Waters 2023:1–13.
9. Hill MJ, Chadd RP, Morris N, Swaine JD, Wood PJ. Aquatic macroinvertebrate biodiversity associated with artificial agricultural drainage ditches. Hydrobiologia 2016;776(1):249–60.
10. Lynch AJ, Cooke SJ, Arthington AH, Baigun C, Bossenbroek L, Dickens C, et al. People need freshwater biodiversity. WIREs Water 2023.
11. Fehlinger L, Misteli B, Morant D, Juvigny-Khenafou N, Cunillera-Montcusí D, Chaguaceda F, et al. The ecological role of permanent ponds in Europe: a review of dietary linkages to terrestrial ecosystems via emerging insects. Inland Waters 2023;13(1):30–46.
12. Tews J, Brose U, Grimm V, Tielbörger K, Wichmann MC, Schwager M, et al. Animal species diversity driven by habitat heterogeneity/diversity: the importance of keystone structures. Journal of Biogeography 2004;31(1):79–92.
13. Rolke D, Jaenicke B, Pfaender J, Rothe U. Drainage ditches as important habitat for species diversity and rare species of aquatic beetles in agricultural landscapes (Insecta: Coleoptera). J Limnol 2018;77(3).
14. Biggs J, Fumetti S von, Kelly-Quinn M. The importance of small waterbodies for biodiversity and ecosystem services: implications for policy makers. Hydrobiologia 2017;793(1):3–39.
15. Kemp VA, Grey J, Hemprich-Bennett D, Rossiter SJ, Lewis OT, Wilkinson CL, et al. Changes in trophic ecology of mobile predators in response to rainforest degradation. Journal of Applied Ecology 2023;60(6):1139–48.
16. Múrria C, Iturrarte G, Gutiérrez-Cánovas C. A trait space at an overarching scale yields more conclusive macroecological patterns of functional diversity. Global Ecol. Biogeogr. 2020;29(10):1729–42.
17. Ao S, Chiu M-C, Lin X, Cai Q. Trait selection strategy for functional diversity in freshwater systems: A case framework of macroinvertebrates. Ecological Indicators 2023;153:110450.

18. Zinkann A-C, Wooller MJ, O'Brien D, Iken K. Does feeding type matter? Contribution of organic matter sources to benthic invertebrates on the Arctic Chukchi Sea shelf. Food Webs 2021;29:e00205.
19. Bearhop S, Adams CE, Waldron S, Fuller RA, Macleod H. Determining trophic niche width a novel approach using stable isotope analysis. Journal of Animal Ecology 2004(73):1007–12.
20. Bonnaffé W, Danet A, Leclerc C, Frossard V, Edeline E, Sentis A. The interaction between warming and enrichment accelerates food-web simplification in freshwater systems. Ecol Lett 2024;27(8):e14480. PubMed PMID: 39096032.
21. Guzman I de, Elosegi A, Schiller D von, González JM, Paz LE, Gauzens B, et al. Treated and highly diluted, but wastewater still impacts diversity and energy fluxes of freshwater food webs. J Environ Manage 2023;345:118510. PubMed PMID: 37390732.
22. Layman CA, Quattrochi JP, Peyer CM, Allgeier JE. Niche width collapse in a resilient top predator following ecosystem fragmentation. Ecol Lett 2007;10(10):937–44. PubMed PMID: 17845294; PubMed Central PMCID: PMC2040226.
23. DeNiro MJ, Epstein S, editors. You are what you eat (plus a few‰): the carbon isotope cycle in food chains. Geological Society of America - Abstracts with Programs; 1976.
24. Post DM. Using stable isotopes to estimate trophic position: Models, methods and Assumptions. Ecology 2002;83(3):703–18.
25. Jackson AL, Inger R, Parnell AC, Bearhop S. Comparing isotopic niche widths among and within communities: SIBER - Stable Isotope Bayesian Ellipses in R. J Anim Ecol 2011;80(3):595–602. PubMed PMID: 21401589.
26. Leibold MA. The Niche Concept Revisited: Mechanistic Models and Community Context. Ecology 1995(76(5)):1371–82.
27. Polechová J, Storch D. Ecological Niche. In: Encyclopedia of Ecology: Elsevier; 2019. p. 72–80.
28. Giménez Gómez VC, Verdú JR, Gómez-Cifuentes A, Vaz-de-Mello FZ, Zurita GA. Influence of land use on the trophic niche overlap of dung beetles in the semideciduous Atlantic forest of Argentina. Insect Conserv Diversity 2018;11(6):554–64.
29. Newsome SD, Del Martinez Rio C, Bearhop S, Phillips DL. A niche for isotopic ecology. Frontiers in Ecology and the Environment 2007;5(8):429–36.
30. Hartong H, Schmid C, Kornack I, Wolter A. Landschaftsrahmenplan Landkreis Havelland: Band 2 Bestand und Bewertug. Landkreis Havelland; 2014.
31. Kalettka T, Rudat C. Hydrogeomorphic types of glacially created kettle holes in North-East Germany. Limnologica 2006;36(1):54–64.
32. COIL. Data Interpretation Guide for Isotopic Analysis; 2021. Available from: www.cobsil.com.
33. Peterson BJ, Fry B. Stable Isotopes in Ecosystem Studies. Annual Review of Ecology and Systematics 1987:293–320.
34. Lewis KA, Tzilivakis J, Warner DJ, Green A. An international database for pesticide risk assessments and management. Human and Ecological Risk Assessment: An International Journal 2016;22(4):1050–64.
35. Olker JH, Elonen CM, Pilli A, Anderson A, Kinziger B, Erickson S, et al. The ECOTOXicology Knowledgebase: A Curated Database of Ecologically Relevant Toxicity Tests to Support Environmental Research and Risk Assessment. Environ Toxicol Chem 2022;41(6):1520–39. PubMed PMID: 35262228; PubMed Central PMCID: PMC9408435.
36. Gholizadeh M, Heydarzadeh m. Functional feeding groups of macroinvertebrates and their relationship with environmental parameters, case study: in Zarin-Gol River. Iranian Journal of Fisheries Sciences 2017:2532–43.
37. Parreira de Castro DM, Reis de Carvalho D, Pompeu PdS, Moreira MZ, Nardoto GB, Callisto M. Land Use Influences Niche Size and the Assimilation of Resources by Benthic

Macroinvertebrates in Tropical Headwater Streams. PLoS One 2016;11(3):e0150527. PubMed PMID: 26934113; PubMed Central PMCID: PMC4774910.
38. Ramírez A, Gutiérrez-Fonseca PE. Functional feeding groups of aquatic insect families in Latin America: a critical analysis and review of existing literature. Rev Biol Trop 2014;62 Suppl 2:155–67. PubMed PMID: 25189076.
39. Schmidt-Kloiber A, Hering D. www.freshwaterecology.info – An online tool that unifies, standardises and codifies more than 20,000 European freshwater organisms and their ecological preferences. Ecological Indicators 2015;53:271–82.
40. R core Team. R: A language and environment for statistical computing.; 2023.
41. Jackson A, Parnell A. SIBER: Stable Isotope Bayesian Ellipses in R 2023.
42. Jackson A, Parnell A. _SIBER: Stable Isotope Bayesian Ellipses in R_; 2023.
43. R core Team. R: A Language and Environment for Statistical Computing; 2024.
44. Oksanen J, Simpson GL, Blanchet GF, Kindt R, Legendre P, Minchin PR, et al. vegan: Community Ecology Package; 2022.
45. Price EL, Sertić Perić M, Romero GQ, Kratina P. Land use alters trophic redundancy and resource flow through stream food webs. J Anim Ecol 2019;88(5):677–89. PubMed PMID: 30712255.
46. Guzman I de, Montoya JM, Elosegi A, Pérez-Calpe AV, Schiller D von, González JM, et al. Food-web energy fluxes, energy transfer efficiency, and diversity respond distinctively to pollution and water diversion in rivers. Freshwater Biology 2024.
47. Cross WF, Wallace JB, Rosemond AD, Eggert SL. Whole-system nutrient enrichment increases secondary production in a detritus based ecosystem. Ecology 2006;87(6):1556–65.
48. García L, Pardo I, Cross WF, Richardson JS. Moderate nutrient enrichment affects algal and detritus pathways differently in a temperate rainforest stream. Aquat Sci 2017;79(4):941–52.
49. Brauns M, Gücker B, Wagner C, Garcia X-F, Walz N, Pusch MT. Human lakeshore development alters the structure and trophic basis of littoral food webs. Journal of Applied Ecology 2011;48(4):916–25.

Supporting information

Supporting information 1: Active substances measured (99) with their limits of quantification and detection, as well as the endpoints and test organism used for the toxicity calculations.

Supporting information 2: Biplot for benthic macroinvertebrate before correction for δ15N values from and the influence of organic fertilization. Ellipses incorporate 40% of the data.

Supporting information 3: SIBER density plots for the Layman metrics, computed via Bayesian analysis. Black dots are the modes. Shaded boxes represent the 50 %, 75 % and 95 % confidence intervals, from dark to light red/blue. Grouping is based on the FFG (six groups), the communities are assigned for the water body types (pond, ditch).

Supporting information 4: Representation of the FFGs as coloured dots and ellipses, 40% of the data points are represented by the ellipses.

Supporting information 5: Overview of the sampled FFGs and determination for the ponds and ditches.

Supporting information 6: Layman metrics for ponds and ditches, calculated for the FFGs and the different water bodies as grouping.

Supporting information 7: Biplots for benthic macroinvertebrates in ponds (top) and ditches (bottom). Raw data was corrected with δ15N means of resources.

Supporting information 8: Biplots for benthic macroinvertebrates in ponds (top) and ditches (bottom). Raw data was corrected with δ15N means of resources and δ13C values were corrected for the influence of C4-plant maize.

Figure 1

Click here to access/download;Figure;Fig1.tif

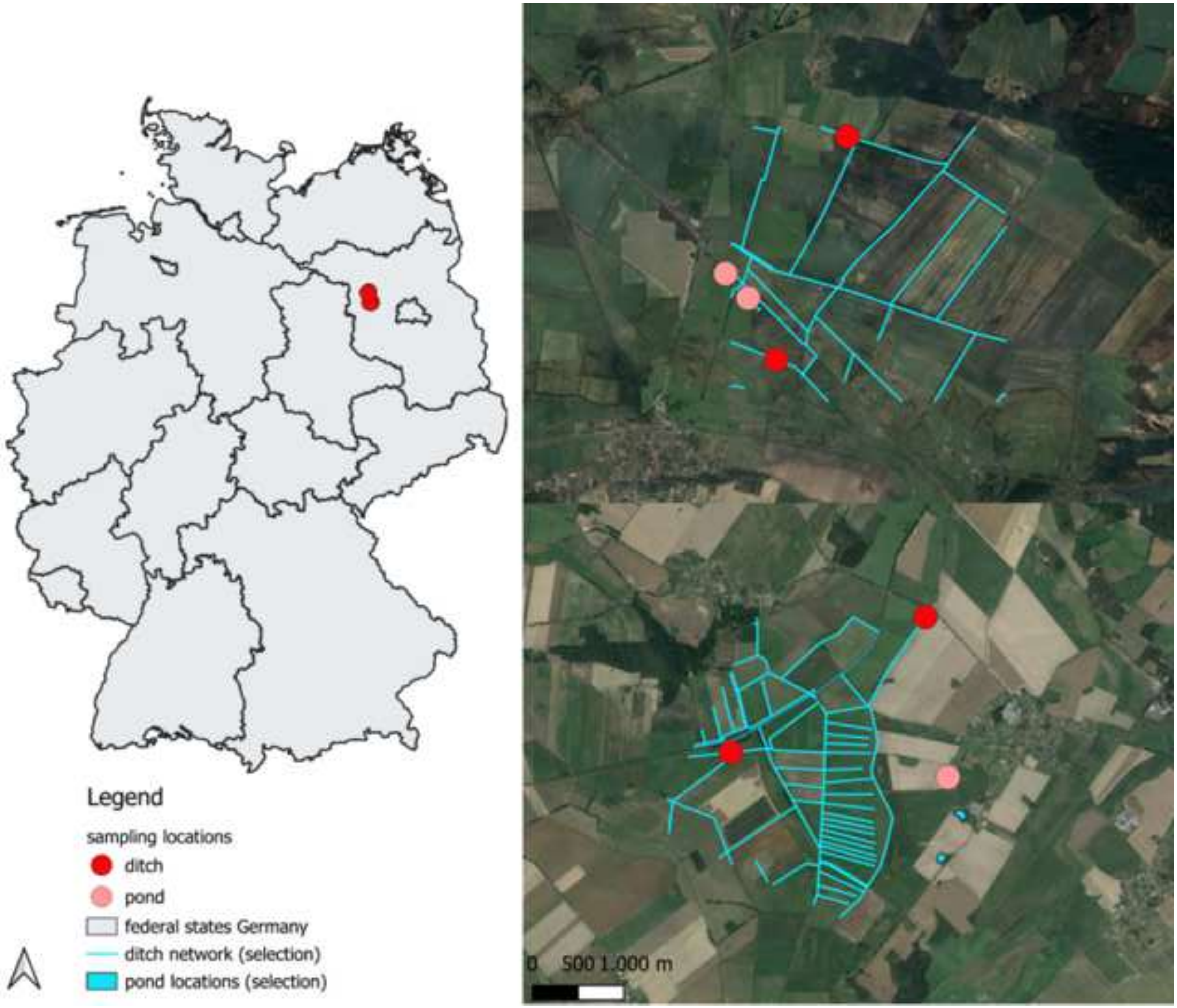

Figure 2

Click here to access/download;Figure;Fig2.tif

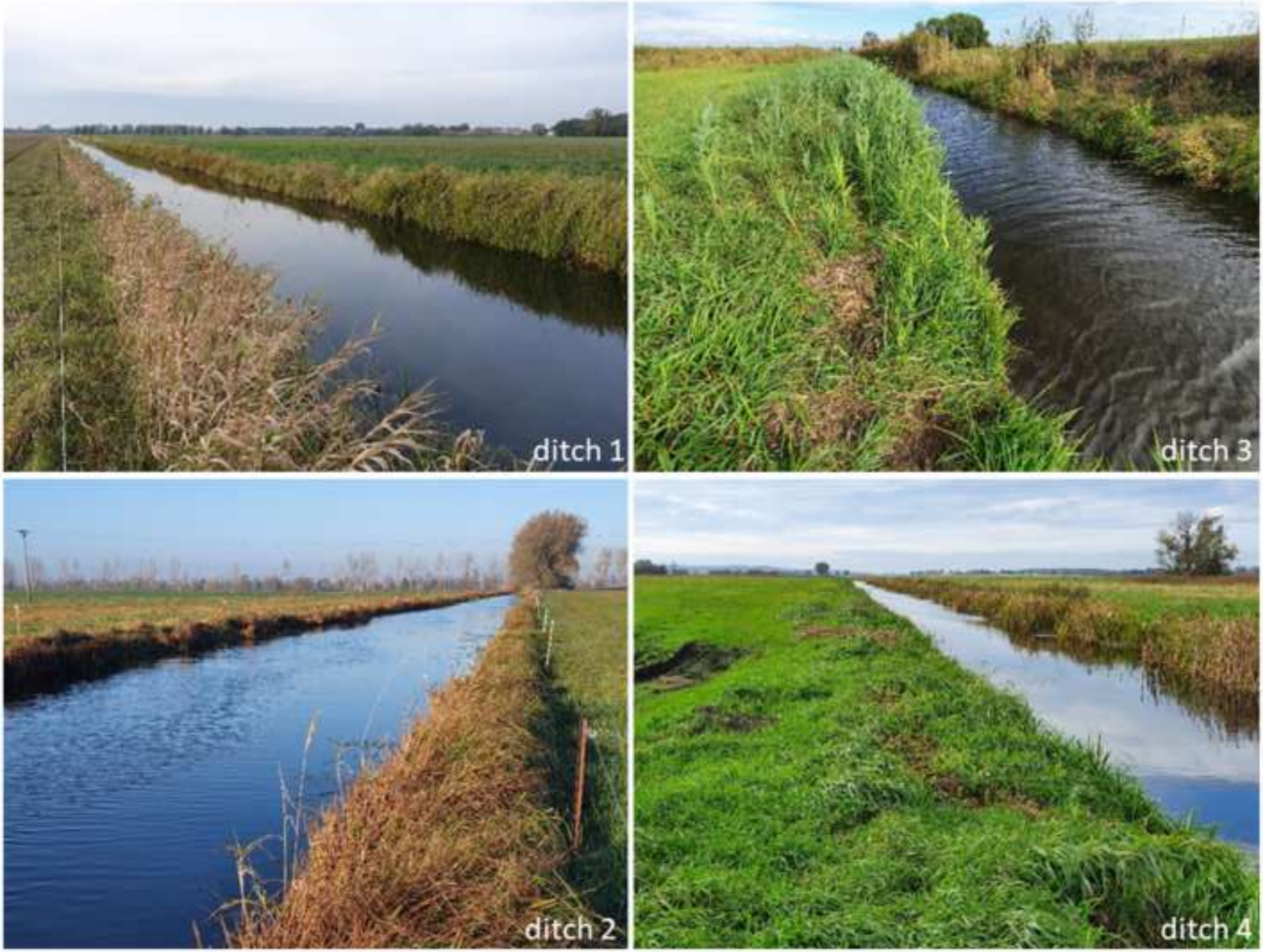

Figure 3

Click here to access/download;Figure;Fig3.tif

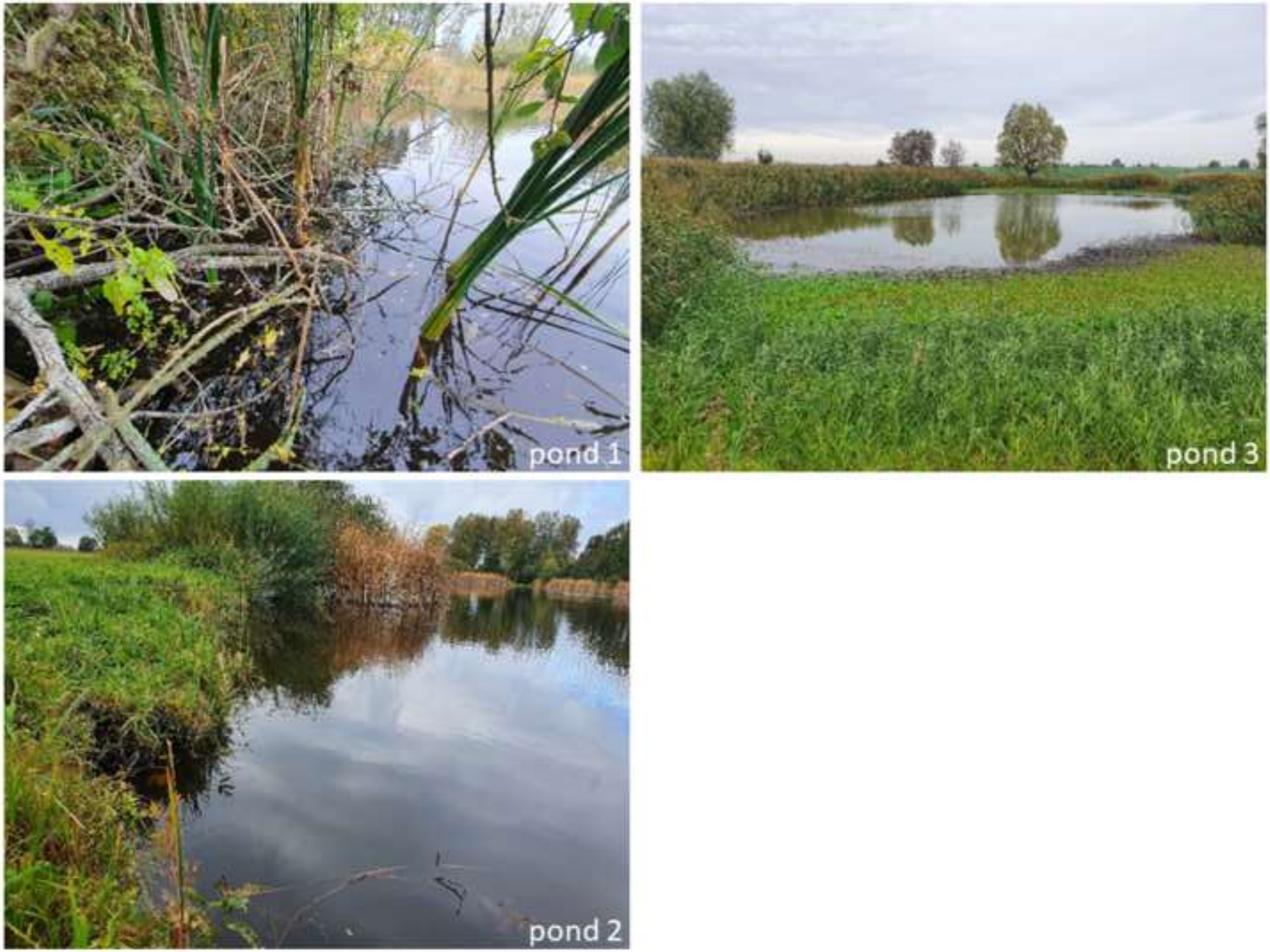

Figure 4

Click here to access/download;Figure;Fig4.tif

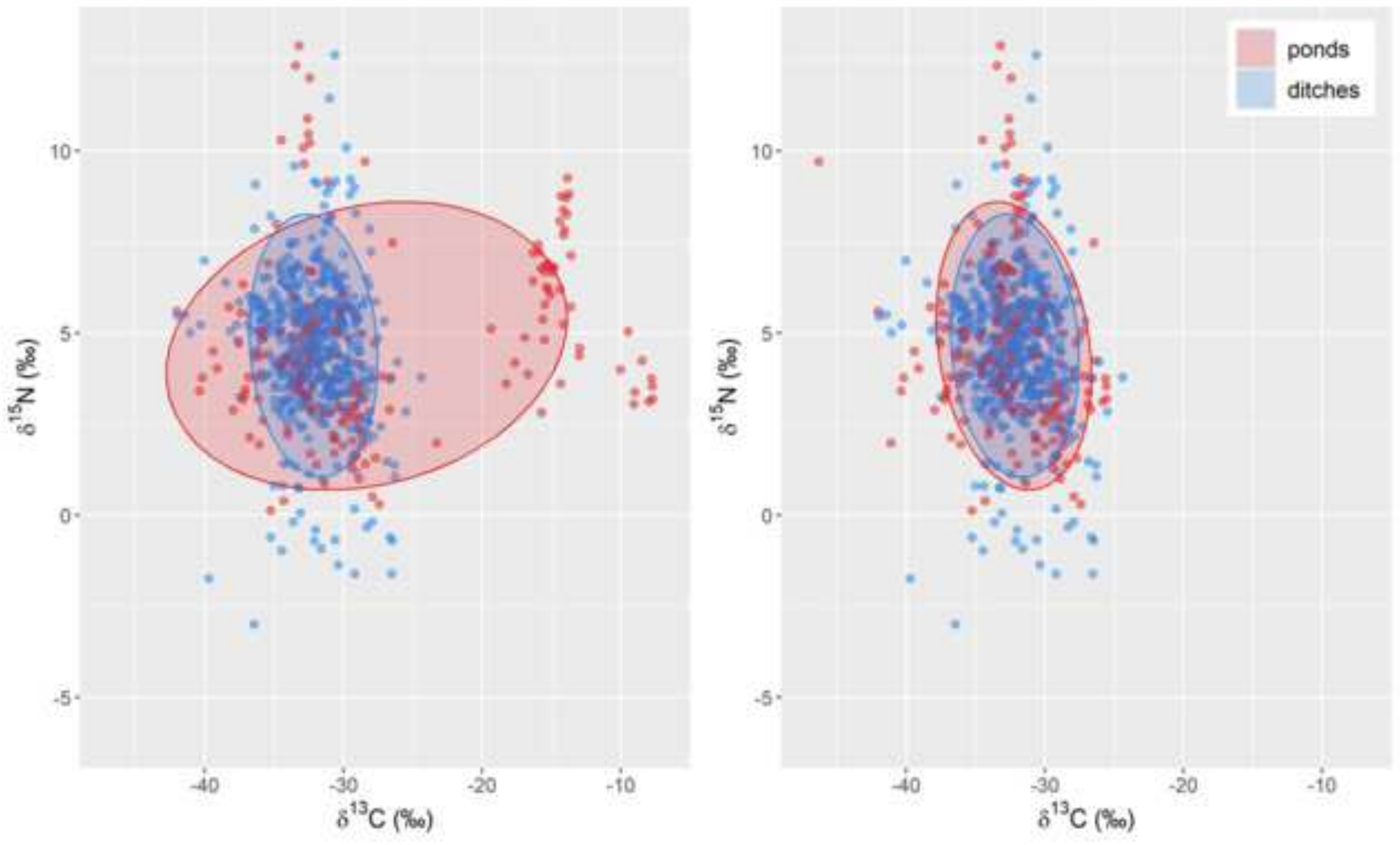

Figure 5 Click here to access/download;Figure;Fig5.tif

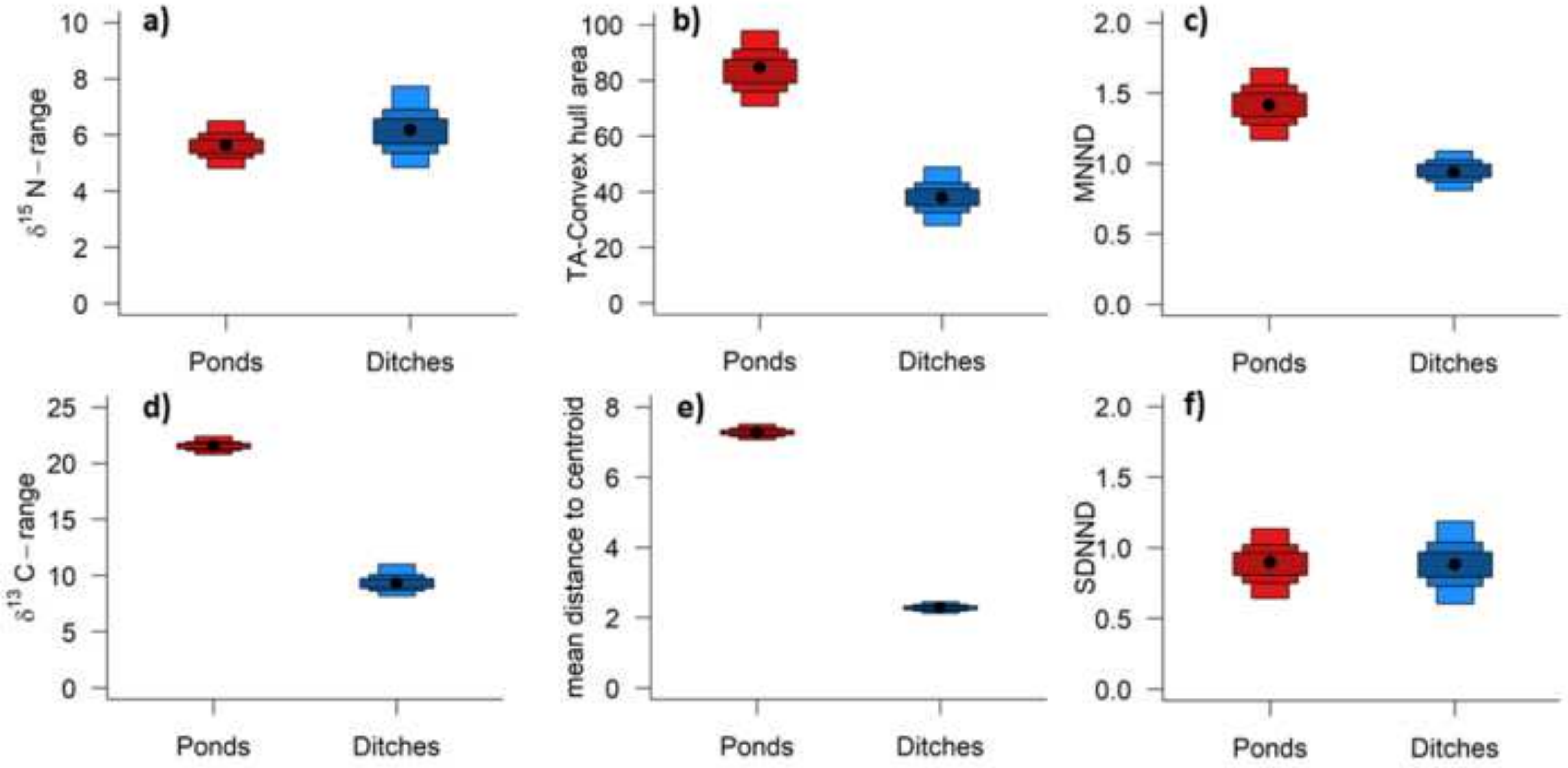

Figure 6

Click here to access/download;Figure;Fig6.tif

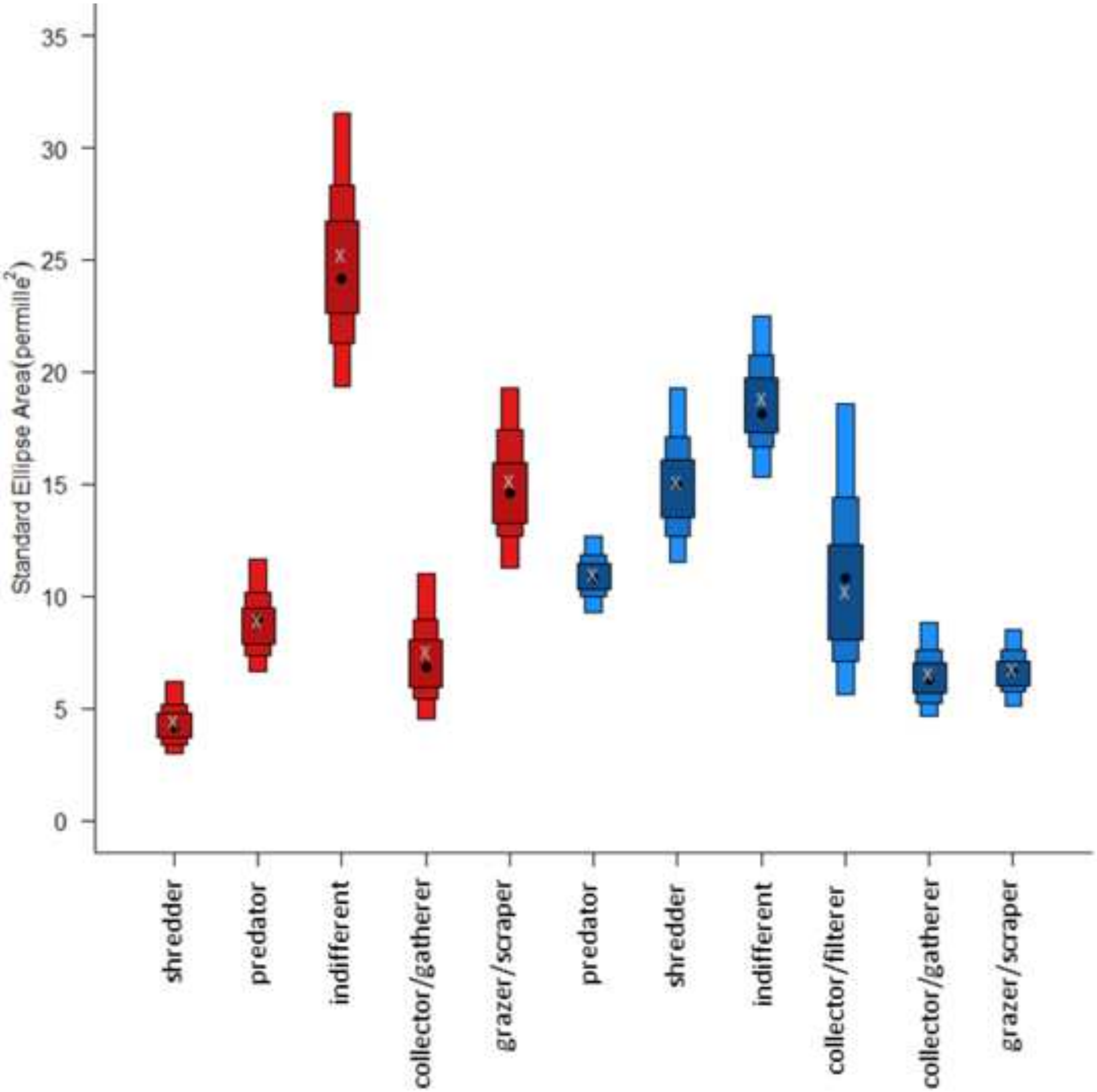

Figure 7

Click here to access/download;Figure;Fig7.tif

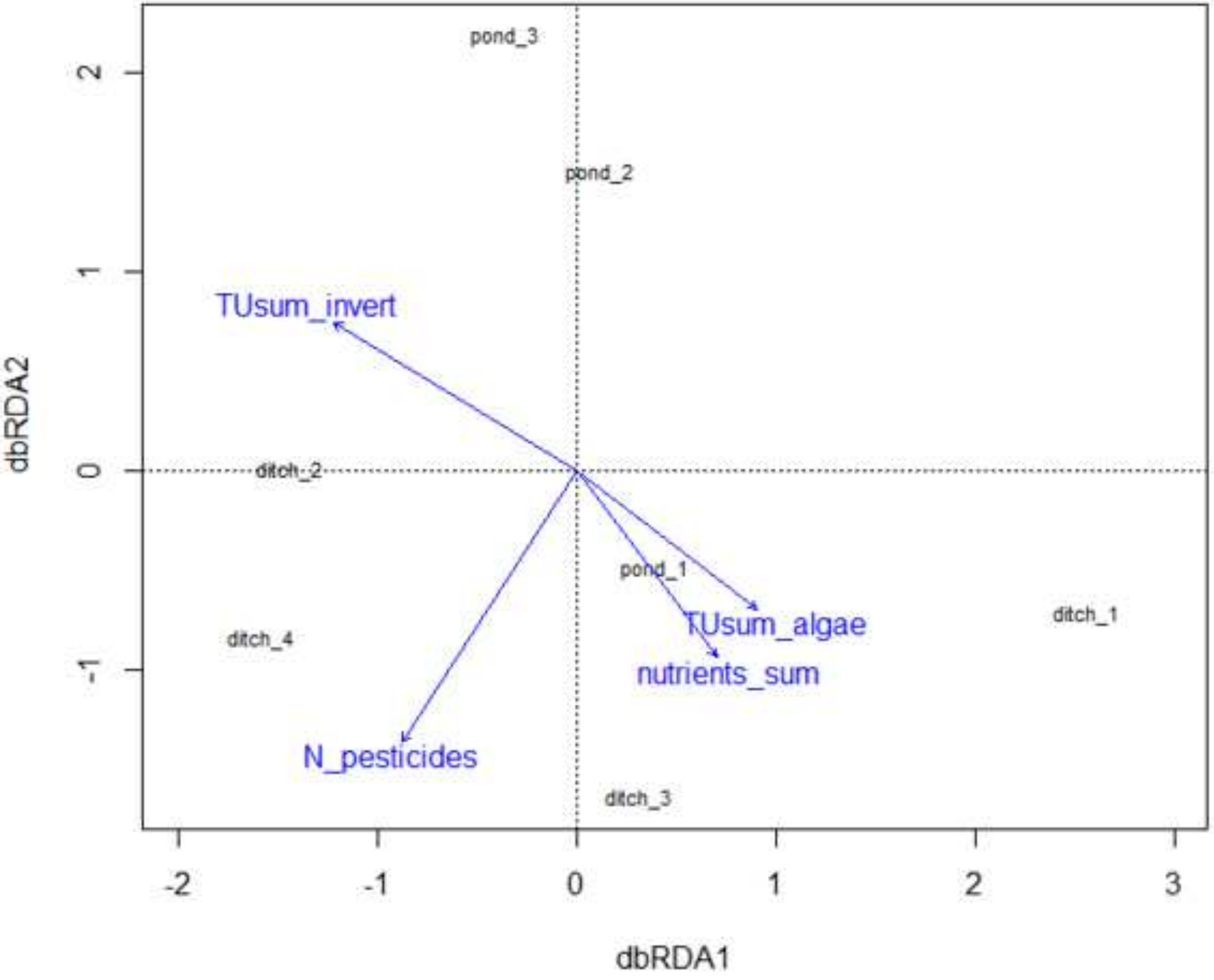

Click here to access/download
Supporting Information
Supporting Information_Trau_2025.docx
