# Supplementary material for: Assessing agricultural effects on benthic invertebrate communities in ponds and ditches using δ¹⁵N and δ¹³C isotope niches
Source: PLoS One. 2025 Nov 24;20(11):e0336486. doi: 10.1371/journal.pone.0336486 (PMC12643296; doi:10.1371/journal.pone.0336486)
Supplement: Attachment — Submitted filename: PONE-D-25-03786_R1_reviewer.pdf [file pone.0336486.s011.pdf]

PLOS ONE

Assessing agricultural effects on benthic invertebrate communities in ponds and ditches using δ15N and δ13C isotope niches

--Manuscript Draft--

**Manuscript Number:**	PONE-D-25-03786R1
**Article Type:**	Research Article
**Full Title:**	Assessing agricultural effects on benthic invertebrate communities in ponds and ditches using δ15N and δ13C isotope niches
**Short Title:**	Assessing agricultural effects on benthic invertebrate isotope niches
**Corresponding Author:**	Fee Nanett Trau<br>Julius Kühn-Institut Berlin: Julius Kuhn-Institut Berlin<br>Berlin, GERMANY
**Keywords:**	agricultural landscape; benthic invertebrates; food web; isotope niche; small water bodies
**Abstract:**	Agriculture is one of the dominant types of land use worldwide. The conversion of land and intensification of agriculture have severe impacts on biodiversity and the associated ecosystem functions in the production areas and in natural or semi-natural habitats embedded in the agricultural landscape. Ponds and ditches, while vulnerable to the negative impacts of agriculture, are valuable habitats for benthic invertebrates due to their heterogeneity and location in a rather homogeneous landscape and harbor a diverse community as well as endangered or rare taxa. It is challenging to detect community disturbance in these habitats due to indirect or multiple stressors from agriculture, as shown by the failure of classic structural biodiversity metrics, such as overall taxa number, Shannon or evenness index, to perform well in previous studies. Therefore, we used a functional approach to understand the influence of agricultural stressors on isotopic compositions of benthic invertebrate in ditch and pond communities. Benthic invertebrates, from six different functional feeding groups (FFGs), and water samples were collected from three ponds and four ditches in an agricultural landscape in Brandenburg, Germany. We analyzed the carbon and nitrogen stable isotope ratios of the invertebrates and the pesticides and nutrient residues of water samples. Estimates of community metrics were derived using a Bayesian approach for the two water body types and the FFGs present. A distance-based Redundancy Analysis was carried out to detect the environmental variables that show the maximum correlation with the derived metrics. Benthic invertebrates in ponds occupied a larger total area and showed greater spacing of individuals in the isotope space than communities in ditches, indicating that more resources and/or habitats were available and utilized in ponds. No clear pattern emerged when comparing the FFGs in the two water body types, but some unexpected differences were found, for example in FFG predator, which was more abundant and diverse in ditches. Our study found that benthic invertebrate communities respond in complex ways to stressors in small unstable systems, which are also subject to strong fluctuations in biotic and abiotic parameters. The impacts of individual structures within each water body are more pronounced than influences based on the type of water body. However, larger datasets may yield in more pronounced patterns on effects of agriculture on stable isotope composition in ponds and ditches.
**Order of Authors:**	Fee Nanett Trau
	Kathrin Fisch
	Stefan Lorenz
**Response to Reviewers:**	Author’s response:<br>We thank the Academic Editor for handling our manuscript and for the careful selection of qualified Reviewers. We are glad to receive such a constructive, detailed, and fair review report. We revised our manuscript according to the comments of the Reviewers.<br>Comments to the Author<br>1. Is the manuscript technically sound, and do the data support the conclusions?<br>The manuscript must describe a technically sound piece of scientific research with data

that supports the conclusions. Experiments must have been conducted rigorously, with appropriate controls, replication, and sample sizes. The conclusions must be drawn appropriately based on the data presented.
Reviewer #1: Partly
Reviewer #2: Partly

____

2. Has the statistical analysis been performed appropriately and rigorously?
Reviewer #1: I Don't Know
Reviewer #2: Yes

____

3. Have the authors made all data underlying the findings in their manuscript fully available?
The PLOS Data policy requires authors to make all data underlying the findings described in their manuscript fully available without restriction, with rare exception (please refer to the Data Availability Statement in the manuscript PDF file). The data should be provided as part of the manuscript or its supporting information, or deposited to a public repository. For example, in addition to summary statistics, the data points behind means, medians and variance measures should be available. If there are restrictions on publicly sharing data—e.g. participant privacy or use of data from a third party—those must be specified.
Reviewer #1: Yes
Reviewer #2: Yes

____

4. Is the manuscript presented in an intelligible fashion and written in standard English?
PLOS ONE does not copyedit accepted manuscripts, so the language in submitted articles must be clear, correct, and unambiguous. Any typographical or grammatical errors should be corrected at revision, so please note any specific errors here.
Reviewer #1: Yes
Reviewer #2: Yes

____

5. Review Comments to the Author
Please use the space provided to explain your answers to the questions above. You may also include additional comments for the author, including concerns about dual publication, research ethics, or publication ethics. (Please upload your review as an attachment if it exceeds 20,000 characters)

____

6. PLOS authors have the option to publish the peer review history of their article (what does this mean?). If published, this will include your full peer review and any attached files.
If you choose "no", your identity will remain anonymous but your review may still be made public.
Do you want your identity to be public for this peer review? For information about this choice, including consent withdrawal, please see our Privacy Policy.
Reviewer #1: Yes: Daniela Cortes Guzman
Reviewer #2: No

____

Reviewer #1:
I finalized revising the manuscript "Assessing agricultural effects on benthic invertebrate communities in ponds and ditches using $\delta^{15}N$ and $\delta^{13}C$ isotope niches".
The topic is interesting and attractive to a broad audience. However, major improvements need to be done in the manuscript. The questions and hypotheses, and how the methods were applied to solve these questions, were rather cryptic for me. It was generally hard to understand from the introduction which is the knowledge gap the authors are trying to fulfill. Moreover, without clear questions, assessing the science behind the methods and results was difficult. My major misunderstanding is why the authors expect differences between the two types of ecosystems assessed, and therefore, why this is an interesting question in the framework of agricultural stressors.
I think the authors have an interesting and valuable dataset with the potential of answering interesting ecological questions. However, significant work needs to be done. I left detailed comments for each of the sections.
Author's response:
We thank the reviewer for the time that she invested in evaluating our manuscript and we hope that we have addressed all the concerns raised by the reviewer accordingly.

Powered by Editorial Manager® and ProduXion Manager® from Aries Systems Corporation

Comments reviewer 1 with authors responses:
Abstract
14: Why do ponds and ditches harbor particularly endangered or scarce taxa? Moreover, why do you mean by scarce, is it low abundant taxa or taxa with low occupancy? I'd recommend removing this last part of the sentence.
Explained in more detail why these water bodies are valuable habitats and replaced scarce with rare.
16 I disagree. Numerous studies have shown the adverse effects of agricultural land use in biodiversity. Perhaps what you mean is that these habitats are likely to face multiple stressors, all derived from agriculture, which results in a problematic complex to address. More importantly, the reader does not know so far which are these previous approaches and why they were not successful. For example, what do you mean by "classic structural biodiversity metrics"?
Added the word "indirect or multiple" to stressors and examples for the classical metrics mentioned. Problems with previous studies will be elaborated on in the introduction section. The sentence in the abstract should only function as a teaser for the reader.
19: We analyzed the carbon and nitrogen stable isotope composition of the invertebrates and the pesticides and nutrient residues of water.
Addressed.
21 readers do not know what are Standard ellipse and Layman B community metrics. I recommend leaving technical terms out of the abstract.
Addressed. Terms left out and circumscribed as "Estimates of community metrics…"
22 FFG should be introduced earlier.
FFGs are introduced earlier in the abstract when the general approach is explained. Line 18 ff. "Benthic invertebrates, from six different functional feeding groups (FFGs), and water samples were collected from three ponds and four ditches in an agricultural landscape in Brandenburg, Germany."
28 Which differences? You're leaving the reader hanging on the unexpected differences but don't tell them (us) which.
Explanation for predator example added. "…, which was more abundant and diverse in ditches."

28-32 See my previous comment. These sentences are uninformative for readers. We don't know why case-by-case patterns emerged, or where the differences lay. What do you mean by the production or consumption level? How was production measured or estimated? We also don't know which strong fluctuations on parameters did you observed; therefore, we don't know how did you reach this conclusion.
Addressed. Deleted the sentence from the abstract as it is confusing here and will be elaborated in the results and discussion.
30 Remove In the end
Addressed.
Introduction
40 is it specialization, and use of fertilizer and pesticides homogenizing the landscape? I'd say that the expansion of agricultural land is what homogenizes the landscape. Ref 3
I would still argue that it is specialisation and the increased use of inputs in intensive or industrial agriculture that makes a landscape more homogenous as intensive agriculture usually goes hand in hand with larger plots being used for one crop, which can be sprayed and harvested by large machines. The expansion of agriculture is another point that can also lead to homogenization when land is converted to intensive agricultural land. Thus, I kept the sentence but added expansion to the explanations in the sentence.
43 What do you mean by semi-natural habitats? Under less intensive agriculture?
I mean habitats such as ponds, ditches, field margins or hedges. In general, habitats that were altered by humans but still have a high biodiversity value. Examples added in text.
45 Starting a paragraph with an example is quite counterintuitive. You haven't introduced yet non-productive areas, so why are you giving us examples?
I connected the paragraph with the paragraph before, as this is the explanation with examples for the semi-natural areas. See also comment above.
49 Same as in the abstract: would you define scarce more precisely.
Replaced scarce with rare.
50 a high richness compared to what? Also, these groups are highly abundant in many

aquatic ecosystems; therefore, why are they particularly important in ditches?
I changed the sentence to make my intention with that sentence clear. "Also ditches, which are in close proximity to agricultural practices, have the potential to host diverse communities as well as rare or endangered aquatic species (9) and show a high richness for the groups Coleoptera, Gastropoda, Trichoptera and Heteroptera (9).
51 I think this is your topic sentence for this paragraph; therefore, it should be the first one -or maybe the second one, but at the beginning!
Paragraph reordered:
"Ponds and ditches are biodiversity hotspots and form aquatic corridors in a landscape dominated by agriculture in need of conservation action (5,6). Unfortunately, these small water are usually not included in monitoring programs (5,7), but the importance of particularly ponds, but also ditches, for benthic macroinvertebrate biodiversity and ecosystem services is increasingly gaining recognition in research activities (5,9–14). Ponds are known to harbour high species numbers (9), however also ditches, which are usually in close proximity to agricultural practices, have the potential to host diverse communities as well as rare or endangered aquatic species (5) and show a high richness for the groups Coleoptera, Gastropoda, Trichoptera and Heteroptera (5)."
53 but this contradicts your previous sentence in line 48
Yes true, deleted the contradicting part. The main point here was that those small water bodies are not included in monitoring programs.
57 in which sense is challenging? Actually, invertebrates are one of the most used groups for biodiversity assessment because sampling is comparatively easy and cheap to other taxa, and they are very good indicators of ecosystem status and functions.
Added explanation in the sentence. "…the assessment of water or habitat quality by benthic invertebrate biodiversity is challenging…"
59 But there is extensive literature showing the effects of agriculture on biodiversity. I think you need to be more specific as to why functional approaches outperform, or complement, taxonomic approaches. For example, why would functional approaches be more sensible to persistent stressors or short gradients? Ref 8
Added: "Additionally species occurrences may be subject to some degree of stochasticity (15) and functional responses can reflect the ecological conditions and habitat change better than taxonomic approaches (16,17)."
64 why were these other traits not included in your analysis? One important effect of agricultural land use is the change in inputs of organic matter to the water bodies; this justifies the use of feeding groups. However, other effects are channelization or modification of the physical structure, which might produce a response in locomotion; changes in productivity (eutrophication), with effects on body size and respiration; changes in temperature, with effects on thermal tolerance and respiration. In other words, you need either to include more traits, or to justify why feeding groups are the best approach to address the effects of agriculture, including persistent stressors and short gradients.
Channelization and physical modification are important factors for (natural) streams (or lakes for physical modification) suffering from anthropogenic pressure. Ditches are per se longitudinal channels, all of which are constantly modified in their physical structure by bank cleaning and mowing. Therefore, we assume communities to be adapted to this environment.
Added in the manuscript: "We selected FFG to address the effects of agriculture as further stressors such as channelization and modification of physical structure do not vary in their intensity in the selected water bodies"
80 this paragraph is disconnected with the rest of the introduction. While important concepts need to be defined, the reader wants to know how the niche concept(s) is related to your own work and very importantly, to your question and knowledge gap. It is your task, as writers, to tell the reader how this concept can help understanding effects of stressors in biodiversity -not give the reader homework . For example, I'd recommend combining this paragraph with the trait paragraph, since the niche is going to be defined based on those traits. This will provide more coherence between your paragraphs and shorten your introduction.
Addressed. Combined with the trait paragraph as suggested. This also makes clear that the adaptation of the niche concept in stable isotope studies is done to create isotopic niches, which are a proxy for the habitat and resource use of a community. Habitat and resources are linked to the land use and agricultural stressors. This link was stressed in the text.
88 we used. You haven't defined isotopic niche nor realized niche. Feeding resources? And which habitats? Do you mean between ponds and ditches? If so, why feeding

Powered by Editorial Manager® and ProduXion Manager® from Aries Systems Corporation

resources and, therefore, isotopic niches would differ between them?
Described in more detail above in the manuscript.
89 we compared
Addressed
90 which groups?
Addressed. "…compared niche widths of FFGs and the pond and ditch communities based on…"
92 are you assessing more than one stressor? Or is it pesticides the only metric you are using?
No, pesticides and nutrients are examined. Changed in the text as follows: "…influence of agricultural stressors (toxicity of water samples due to pesticide residues and eutrophication due to nutrient inputs)…"
93 we asked. Can
Adressed
94 I don't fully understand your questions. Are you assessing isotopic niches in a gradient of agricultural stress, as measured by pesticides? Why do you expect differences, or not, between ponds and ditches?
Added in the text: "We assume that ponds and ditches are influenced differently by agricultural stressors. Ditches are expected to be more strongly influenced by agricultural stressors than ponds due to their characteristics, which are a rather homogeneous habitat in and around the water body, a directly farmed environment with the location in the field (grassland) without a riparian buffer. "
Questions adapted: "We asked the following questions, using ponds and ditches as an example.: Can the isotopic niches depict the effects of agriculture on benthic invertebrate communities in the selected water bodies in the study region? Are the observed patterns of isotopic niches of FFGs comparable in ponds and ditches for the FFGs?"
96 to answer these questions…
Adressed
98 what is the difference between niche differentiation and niche specialization? And why reduced niche specialization would result in less overlap? I'd expect the opposite. How about the expectations for ponds? Do ponds have different effects of agriculture?
The term differentiation and specialisation mean the same. Niche differentiation = competing species use the niche in different ways to coexist, different use of resources, portioning of space and time. The word specialisation was replaced with differentiation in the second part of the sentence.
Yes, it should be "larger overlaps". Changed in the text.
It is not clear to me which questions are being answered and how. Is it the question related to differences in niche width due to different levels of agricultural stress? Then, would these effects differ between ponds and ditches? Why? What do you mean by habitat variability? Is this variability inside each water body, or among types?
Added in the text, see above.
Added text at the beginning of the paragraph: "….affecting habitat availability and quality and thus influencing benthic invertebrate food webs and niche size (33,34)."
Added text: "Ditches, for example, are probably more contaminated, but as they are part of a flow system, pollutants can also be transported downstream. Ponds, on the other hand, are better protected from pesticide inputs, but can act as a source and sink for pesticide residues in the sediment (35)."

Methods
103 I think that saying During the 1980s, the wet lowlands… is enough. Moreover, I'd turn the sentence around: The wet lowlands in the Havelländisches region were drained … during the 1980s.
Addressed
105: What do you mean by traditional grasslands? As in, they were already agricultural lands but transformed into monoculture crops? Or were they natural grasslands converted to agriculture?
I mean grasslands but extensively used with a number of herbs and grasses growing together, extensively managed. This was changed to grassland, which was seeded with only one type of grass. Changed in the text to: "Traditional, extensively managed and diverse grasslands were converted to seeded grasslands…"
107 What about ditches?
What exactly do you mean? I talk about the ditches in the previous sentences, when talking about the draining of land and the established ditch system in the region.

Powered by Editorial Manager® and ProduXion Manager® from Aries Systems Corporation

	113 describe the site's characteristics in order, from Pond 1 forward, and ditch 1 on.<br>Addressed<br>Table 1: How were these habitats characterized? Do they correspond to the littoral zone, the whole water body? Which area of the water body was estimated or quantified? Same for the riparian zone: which distance from the shore was considered riparian, and how these percentages were estimated/quantified?<br>Adressed. Text added in the caption. "Habitats were described for the water bodies, when present in the wadeable area of the shore length with more than 5 %. The riparian zone is defined as the area from the edge of the field including the edge strip to the waterline, on this area the growth of grasses etc. was estimated as a percentage."<br>You could highlight the dominant type of riparian vegetation to help readers focus.<br>Adressed. Written in bold in table 1<br>124 Benthic invertebrates were sampled using hand netting…<br>Addressed. Text reordered. "Benthic invertebrates were sampled using hand netting (mesh size 500 µm) from the habitats (FPOM, CPOM, submerse macrophytes, reed) present in the selected water bodies. Individuals from all FFGs present were sampled and organisms were sorted on-site by taxonomy (to the level of order or family)."<br>126 meaning that samples were composed by combining all habitats?<br>Yes. The sampling strategy of every habitat and description of habitats was used to get representative samples and to describe the water bodies, but data was not analysed for the single habitats.<br>126 how were feeding groups distinguished in the field? Based on morphology?<br>Yes. Taxonomy changed to morphology in the text.<br>129 how is dispatch with boiling water humanely? To what end were they kept alive for 24 hours? And why did you use this boiling water method, instead of preservatives like alcohol or formol. This is related to the isotopic signal, but the reader does not know this.<br>It is humane in the sense that it happens very quickly and the animals hopefully notice as little as possible. They are kept alive for 24 hours so that they can empty their gastrointestinal tract. This material should not be analysed. Information added in the text.<br>131 Explain why their shells were remove.<br>Addressed. Explanation added in the text. "…to only analyse the isotopic signal of the body tissue of selected animals"<br>149 I don't see how conductivity and pH can affect the food web structure or isotopic niche. Oxygen and temperature, indirectly, if they reflect productivity?<br>True. I wanted to say parameters that could function to explain the community's food web structures or isotopic niches of groups. Changed in the text.<br>175 Why was toxicity for macrophytes and algae estimate?<br>As we know from parallel studies currently under review there might be indirect effects from pesticides affecting the invertebrate communities. These effects, e.g. herbicide effects on macrophytes or algae can be measured by the toxicity of the samples towards macrophytes and algae. Therefore, it was estimated here and used as variables in the dbRDA. However, in the end only the value for algae was used in the analysis thus I deleted the information on toxicity on macrophytes in the manuscript.<br>187 So far, FFGs have not being defined or described. What are they, what do they represent, how do you assign an organism to a FFG, or which categories exist. This table should be probably moved to the Results.<br>Paragraph added to describe the FFGs and what they represent: "Classified as collector/filterer are Bivalvia and the family Culicidae Gen. sp., which filter suspended FPOM from the water column (32). The families of Ephemeroptera and some Dipterans belong to the group collector/gatherers, which feed on sedimented FPOM. Grazers/scrapers graze on surfaces where they feed on algae tissue or biofilm, also they feed on algae or plant tissue (32). In the present dataset, those are mostly Gastropods and families belonging to the group are for example Lymnaeidae or Planorbidae. Within the group indifferent families are gathered that could not be assigned to one distinct group. In the present dataset Chironomidae, Corixidae, Haliplidae and Stratiomyidae were assigned to the group. Predators hunt on prey and examples are Dytiscidae, Ceratopogonidae or Coenagrionidae. Typical shredders are Gammaridae or Asselidae, which feed on plant tissue, fallen leaves or CPOM (32). A detailed list of the families belonging to the FFGs can be found in the table supporting Information 5."<br>The authors decided to not move the table to the results section as it is describing the

database for the following methods and thus should be kept in the material and methods section.
188 family determination based on which literature? FFGs can vary in different regions because organisms are flexible in their diets, even given certain morphology. Moreover, they can usually be assigned to more than one category. Even in the book of Merrit, Cummins, and Berg (the “inventors” of FFGs), taxa are usually categorized in more than one group.
Table 3. CPOM and FPOM are in the legend but not the table. Columns after the TU are not explained. How were all these metrics quantified and to what aim? Move to Results.
The table was very wide and some columns were probably not visible for you. However, I can delete them here as the information on habitats and riparian zone is already given in table 1. Column reed in % deleted, as the information is already given in table 1. Columns after TU with information on algae concentrations deleted, as they are not used in the further analysis of the data.
Additionally a sentence before table three was added as explanation and the table was moved to results as suggested.
200 was observed
Addressed
201 of all invertebrates or for primary consumers? This does not seem to be an appropriate justification, from my point of view, precisely because you are looking for potential differences between water body types. To my knowledge, there are two ways of correcting dN: first, by using a basal resource dN value as a baseline; this might have the problem of having different values among sites, if they differ in the amount of fertilizer. Second, using the dN value of the primary consumer with the lowest dN to obtain comparable trophic positions across sites.
The difference of d15N values was subtracted from all consumers in the ditches. We followed the presented approach as the differences between all FFGs between ponds and ditches in their d15N values were visible and most likely originating from fertilization in the ditches (farmers data not shown). See also supporting information 2. Thus, we only correct for the effect of fertilization to compare FFGs between ditches and ponds to avoid biases by the fertilization regime. Therefore, we calculated the correction value for d15N from all invertebrates in ponds and ditches and used it to correct all invertebrates in ditches. As you already stated the commonly used correction with basal resources does not correct the fertilizer effect. The second method you propose, using the d15N value of the lowest primary consumer to correct will also not solve the strong effect of fertilization that might override possible patterns. Therefore, we used the most appropriate method for this special issue in our data.
204 difference between what and what?
The difference between the d15N mean in ditches and d15N mean in ponds. More explanation added in the text.
209 what difference?
The difference between the mean d13C values from pond 1/ 2 and the mean d13C values from pond 3. More explanation added in the text.
210 an alternative is centering or scaling the deltas of all sites to obtain comparable values, but I don’t understand why to alter the variability of the sites showing effects of agriculture, if that’s your question? Moreover, it seems odd and a bit arbitrary to transform values of one site.
Yes, we do observe the effect of agriculture, in this case maize cultivation. This is a special effect as maize is a C4 plant and has different isotope composition than other plants such as grasses, wheat and so on. Our intention was not to measure the effect of a single crop (which in case of C4 plant cropping is just a random effect) when comparing general responses of ponds and ditches. If we would keep original C values the C4 maize effect will override everything else we might be able to observe in the data. Thus we only corrected the d13C values in pond 3 which is the only pond influenced by C4 cropping..
212 Explain what these analyses are and why were they performed
Sentence deleted here and moved to the chapters 2.5.3 and 2.5.4 where the statistical analysis are explained.
233 what information
CD provides information on niche width and species spacing
235 to what end? What is the information provided by these metrics?
Added in the manuscript: “The Bayesian estimates provide robust measures of probability and can reflect model uncertainty by averaging many possible models into

one single model (45). The SEA.c is based on SEA.B, which describes isotopic niche with, and corrected for small sample sizes for unbiased analysis (31). “

237 I don’t understand why do you want to correct for the effects of agriculture? Also, is this another correction or the same one mentioned in the previous subsections?
No, it is the correction from chapters 2.5.1 and 2.5.2. As explained in more detail above we are not correcting for the effect of agriculture itself but for the random effect of C4 cropping at this specific field compared to the rest of the field where C3 plants were cultivated. We are correcting for these effects, as they would override further patterns behind the C3/C4 crop difference due to the dimension of d13C scaling between C3 and C4for further analysis I used the corrected data to compare the FFGs in the two water body types.
Results
265 it hasn’t been explained or justified why these two types of habitats would differ in the isotopic composition
Text was changed and further explanation on this issue has been added in detail at the end of the introduction as follows:
“ We compared niche widths of FFGs and the pond and ditch communities based on a geometric approach originally developed by (25) to examine the isotopic niches of benthic invertebrates in ponds and ditches. The aim is to assess the influence of agricultural stressors (toxicity of water samples due to pesticide residues and eutrophication due to nutrient inputs) on the isotopic niche metrics.
We assume that ponds and ditches are affected differently by agricultural stressors, affecting habitat availability and quality and thus influencing benthic invertebrate food webs and niche size (33,34). Ditches are expected to be more strongly influenced by agricultural stressors than ponds due to their characteristics, which are a rather homogeneous habitat in and around the water body, a directly farmed environment with the location in the field (grassland) without a riparian buffer. Ditches, for example, are probably more contaminated, but as they are part of a flow system, pollutants can also be transported downstream. Ponds, on the other hand, are better protected from pesticide inputs, but can act as a source and sink for pesticide residues in the sediment (35). The water body type’s ponds and ditches are used as gradients of agricultural effects, including nutrient enrichment, pesticides, and the proportion of surrounding crops to test our hypotheses. We asked the following questions, using ponds and ditches. Can the isotopic niches depict the effects of agriculture on benthic invertebrate communities in the selected water bodies in the study region? Are the observed patterns of isotopic niches of FFGs comparable in ponds and ditches for the FFGs? To answers these questions, we hypothesize that agricultural activity and low habitat variability in ditches have led to simplified trophic structures, which will be shown by wider isotopic niches, reduced niche differentiation, and larger niche overlap due to reduced differentiation of the FFGs and more generalists present.”
Figure 4 the resolution of the figure is very poor, please improve it.
I don’t understand why resolution was poor for you. Maybe the journal send lower resolutions to the reviewers. Quality is at 300 dpi.
271 replace clearly by a more appropriate term, like statistically
Addressed. The typical range of δ13C values is distinct in the two water body types and larger in ponds (Fig. 5 b).
274 … for the MNND (Fig 5c)…
Addressed
275 evenness is Fig 5f, not e
Addressed
284 Which clear differences, between what and what? Please, try to avoid using words like “clear” or “clearly” since patterns might not be as clear or obvious for the readers.
Addressed.
Figure 6: the boxes correspond to SEA.B or SEAc?
To both. It is explained in the description for the figure as follows: “Black dots are the modes of SEA.B. The grey X indicates the size-corrected standard ellipse area (SEAc) mode.”
284 this paragraph needs to be more organized. It is mixing the isotope values with abundances and diversity for one FFG.
Addressed. Paragraph reordered and simplified.
306-309 these sentences are quite hard to read; please, revise the grammar.
Addressed. Text revised
321 were the explanatory variables scaled before running the analysis?

Powered by Editorial Manager® and ProduXion Manager® from Aries Systems Corporation

	Yes, they were scaled with scale function of the base package. This was explained in chapter 2.5.4 multivariate statistics in the methods section.<br>Discussion<br>327 but how was this tested?<br>Addressed. Changed in the text as follows: " However, within the agricultural affected sites studied here, isotopic niches could not depict benthic invertebrate community changes except for general impacts on basal C and N resources (e.g. by fertilization or C4 cropping)."<br>339 why did you expect more abundance and diversity in ponds?<br>Ponds in general are more diverse as stated by various authors (Biggs J, Fumetti S von, Kelly-Quinn M. The importance of small waterbodies for biodiversity and ecosystem services: implications for policy makers. Hydrobiologia 2017;793(1):3–39 and Williams P, Whitfield M, Biggs J, Bray S, Fox G, Nicolet P, et al. Comparative biodiversity of rivers, streams, ditches and ponds in an agricultural landscape in Southern England. Biological Conservation 2004;115(2):329–41 and Davies BR, Biggs J, Williams PJ, Lee JT, Thompson S. A comparison of the catchment sizes of rivers, streams, ponds, ditches and lakes: implications for protecting aquatic biodiversity in an agricultural landscape. Hydrobiologia 2008;597(1):7–17. This is also stated in the introduction from line 51 ff.<br>341 was any of this measured? Is the relative abundance of herbivores significatively higher in ditches? This all seems very speculative<br>Term abundance deleted in the sentences.<br>Reference added: Hobbie, Sarah E.; Villóeger, Sóebastien (2015): Interactive effects of plants, decomposers, herbivores, and predators on nutrient cycling. In: Torrance C. Hanley und Kimberly J. La Pierre (Hg.): Trophic Ecology: Cambridge University Press, S. 233–259.<br>346 homogenous in which sense? How was homogeneity measured?<br>Homogenous is meant here as low in number of families. However as stated in the results (section: 3.2) the FFG predator is a diverse/heterogenous group in ditches, compared to ponds.<br>347 then, why citing 46?<br>The reference is cited as it explains my statement before.<br>349 I don't think secondary production is an appropriate term here, since this was not measured. Secondary production is a complex metric requiring, at the very least, biomass estimation.<br>Yes, true. Secondary production was substituted by species number in the text.<br>351 again, secondary production was not measured.<br>Yes, see comment above.<br>355 this paragraph is generally confusing. I'd recommend you explain your results at the beginning, as a topic sentence, and then develop the ideas that support your findings. In the current version, sentences supporting and contradicting your findings are mixed making it hard for readers to get the main message you want to transmit.<br>Agreed. The paragraph was slightly changed and rearranged as follows:<br>"Even though there were no clear patterns visible when comparing FFGs, some differences were pronounced. Striking was the finding that contrary to our expectations, the FFG predator was more diverse in ditches. The high diversity of predators in ditches could be explained by the availability of a high number of primary producers facilitated by high nutrients inputs and thus high primary production, which leads to more available prey (52,53). The influence of changes on the base of the food web propagated to higher trophic levels was also observed by (53), where nutrient pollution promoted biofilm production and lead to an increase in herbivory and carnivory by omnivores. This positive influence of fertilization might be explained by intermediate but not high nutrient enrichment, as in the case of (55), which found highest densities of invertebrates at intermediate levels of nutrient enrichment (primarily of phosphate). The high species number is probably stimulated by bottom-up effects of nutrient enrichment (51,54). Although we can assume that the ditches have been enriched for several years, the effects of long-term enrichment predicted by (54), namely the reduction in species numbers and the shift in community structure, were not observed in the ditch system studied when we compare it to the ponds.<br>However in contrast to results from (53), the nutrient pollution in ditches did not lead to more homogenous communities for the FFG predator in ditches compared to ponds, instead the opposite is the case. In ditches, the number of recorded families belonging to the group of predators is nearly twice as high compared to ponds. Therefore, functional redundancy for this feeding type can be assumed."

	356 is there a better term than indifferent? I think generalist could be more appropriate<br>I changed indifferent to the term omnivore.<br>357 meaning enriched values either due to high levels of omnivory or secondary predators.<br>The omnivore-feeding group acts mostly as predators in the pond 2. They formed two clusters. One was on the same level as the predators and the second cluster even higher. I changed the text to make that clear. "In pond 2 they were clearly mostly predatory with two clusters formed. One cluster formed by individuals with the highest δ15N values in that water body and a second cluster with the δ15N values on the same level as the FFG predators in that pond (S 4 Table)."<br>360 herbivorous feeding preference<br>Addressed<br>364 but you measured pollution. This is something you should be able to test.<br>True. I deleted the word probably.<br>368 why is this related to wider habitats?<br>Because a more diverse habitat leads to more diverse resources which leads to a more diverse diet and a more diverse isotopic signal, resulting in wider niches = larger SEA.c mode.<br>There was a sentence added in the methods section to make this connection clear for the reader.<br>"A larger SEA corresponds to a more diverse isotopic signal and thus to wider niches."<br>370 lower diversity of littoral microhabitats in ditches? You started your paragraph talking about ponds.<br>I made clear in the text that I mean more diverse habitat around the ponds than the ditches in this study. I changed the order of the next sentence to make clear that I am talking about lake ecosystems. This was the closest I could get to ponds in the body of scientific literature on that issue.<br>373 a well-documented effect of eutrophication is the development of cyanobacteria, that outcompete other primary producers, but are commonly toxic for animals, explaining why eutrophication could actually reduce grazers diversity.<br>Added in the text as follows: ". In eutrophic waters cyanobacteria blooms can occur which are toxic for other primary producers and can lower the number of grazers, but the concentration of cyanobacteria was high for one pond only (data not shown). This could be a measurement of a peak value that had already been exceeded in the other waters, as cyanobacterial blooms can occur very quickly."<br>374 it is not clear what do you mean by wider habitat? Is the width of the water body, or the number of microhabitats available?<br>Yes, the number of microhabitats is meant. Changed in the text.<br>374 higher taxa richness where? These results were not presented before<br>The sentence was changed to: "Additionally, the larger number of microhabitats and/or more resources used can explain commonly observed patterns of higher taxa richness and the associated possible different preferences in ponds (8-10)"<br>375 what is a taxa group?<br>The larger groups, such as Coleoptera, Diptera, Ephemeroptera etc. Taxa groups named in the text for clarity.<br>381 could pesticides have important direct effects on the invertebrates, leading to higher mortality rates, in addition to the indirect effects through primary production?<br>They could have direct effects and these direct effects can be measured with the TUsum for invertebrates. This value indicates the toxicity of the sample towards invertebrates, see also chapter 2.3.1. However the TU values for invertebrates were not above the critical threshold value of 3.27 (see Liess et al. 2021) in our data thus we do not expect high mortality rates from direct pesticide exposure. Nevertheless, the data is based on one measurement and peak concentrations can be missed.<br>386 it makes sense for me to have higher variability in these groups related to their diets; they are after all generalists<br>Yes, true for the omnivores. Text was changed as follows: "Nonetheless, the FFGs indifferent omnivore in ponds and collector/filterer in ditches show wide credible intervals that indicate a high degree of opportunism and variability in their feeding strategies which is consistent for the FFG omnivore. For the FFG collector/filterer and further sampling would be needed to predict modes with less uncertainty.<br>393 Why?<br>We reformulated this sentence as follows: "Although higher numbers of pesticides were found in ditches compared to ponds, this impact does not transport into the expected toxicity gradient for algae, invertebrates, and aquatic plants, presumably due to the

application of low-toxicity pesticide products by the farmers. Additionally, (54) recently found patterns of synchronous toxicity levels towards algae and aquatic plants between water bodies with a comparable distance to each other like in our study. This should be accounted for in further studies, and future sampling between the months of April and June is generally recommended (54,55).
405 I am surprised to read here that ponds were supposed to be a reference community. This kind of statement should be mentioned very early in the Methods or Introduction. Moreover, I don't think ponds embedded in an agricultural landscape could be used as a reference, less impacted community by agriculture. I'd suggest using the water bodies as a gradient of agricultural effects, including nutrient enrichment, pesticides, and the proportion of surrounding crops to test your hypotheses.
Yes, the ponds are no control community in the real sense of a control. The part of the sentence was deleted. In the manuscript we are concluding that: "Agricultural activity and a homogenous environment did not lead to simplified structures in the ditches compared to ponds."
Your suggestion was added at the end of the introduction leading to the research questions.
Reviewer #2:
Manuscript:
Assessing agricultural effects on benthic invertebrate communities in ponds and ditches
using $\delta^{15}N$ and $\delta^{13}C$ isotope niches
Authors: Fee Nanett Trau, Kathrin Fisch, Stefan Lorenz
Overall, the manuscript presents interesting and valuable research, but several key areas require further clarification to improve its structure, transparency and readability. The study's aim is currently too vague and should be made more specific, particularly in regard to which aspects of benthic invertebrates are being investigated. The introduction would benefit from a clearer distinction between ponds and ditches, specifying their purpose, shape, and typical formation or usage.
In the methods section, there is a lack of clarity surrounding the toxicity calculations mentioned. It is important to explicitly connect these methods to the study's objectives so the reader can follow the logic of your approach. At the moment, the description of toxicity tests, seem disconnected from the rest of the paper and need to be more clearly integrated into the study's goals. If toxicity testing was conducted, please describe the specific methods used, including whether test organisms or particular pesticides were involved. Further, the process for sorting organisms into taxonomic and functional feeding groups (FFGs) should be described more clearly. The use of boiling water as a method needs more explanation- if this method is employed instead of freeze-drying, please justify why it was chosen and explain how it might affect the isotopic analysis.
It would be valuable to emphasize whether the observed differences between ponds and ditches are statistically significant. Discussing the statistical significance of these differences would provide a clearer understanding of their reliability and help contextualize the results within the broader field. Additionally, providing information about the statistical tests used, the p-values, and any relevant confidence intervals could strengthen the interpretation of the findings and give readers more confidence in the conclusions drawn.
Some aspects of the figures and tables require additional detail. For example, Figure 1 should have a more informative caption, specifying what the red labels represent and distinguishing between the different symbols. Some Tables also need clearer explanations.
In addition, there are a number of minor issues that need addressing. It would be useful to include more details on the taxonomic composition of riparian vegetation, ideally at the family level. The citation format should be checked according to the authors' guidelines to ensure consistency. Also, please specify the number of replicates used at each location and for each taxonomic or functional feeding group, as well as the amount of material weighed for each replicate sample.
More specific comments and suggestions are provided in the attached PDF.
Addressing these points will strengthen the manuscript, making it clearer, more precise, and easier for readers to follow.
Author's response:
We thank the Reviewer for the time that he/she invested in evaluating our manuscript and we hope that we have addressed all the concerns raised by the reviewer

Powered by Editorial Manager® and ProduXion Manager® from Aries Systems Corporation

accordingly.

Comments reviewer 2 with authors responses:
Abstract
17/18 The aim should be more detailed and clear. It is not clear which aspect of benthic invertebrates are you investigating?
Aspect added in the text. "… isotopic compositions of benthic invertebrates in ditch and pond communities.
20 carbon and nitrogen stable isotope ratios
Addressed, changed to ratios.
28 what exactly regarding predators?
The large diversity in ditches compared to ponds, added in text.
30-32 this is very general conclusion - try to be more specific following your findings
More specific information added in the text as follows: "The impacts of individual structures within each water body are more pronounced than influences based on the type of water body. "
Introduction
46 I would recommend adding a sentence or two that highlights the key differences between a pond and a ditch, specifically regarding their purpose, shape, and typical formation or usage. This would help clarify the distinction between the two and provide a more comprehensive understanding for the reader..
The differences of ponds and ditches were highlighted with regards to the influence of agriculture they are prone to and the importance for benthic invertebrates in the introduction. Information on the formation and usage can be found in chapter 2.1.
64 Maybe it would be more precise to say "respiration mode" here.
Addressed
69-72 I suggest revising the sentence for clarity. The current wording implies that the principle of "you are what you eat (plus a few ‰)" makes stable isotope analysis possible, but it's actually the use of stable isotopes that has led to the conclusion that this principle applies.
A more accurate phrasing could be: "The principle of "you are what you eat (plus a few ‰)" (23) has led to the use of stable isotopes to analyze isotopic niches, food web structures, and species interactions in ecosystems (24)."
This revision would better reflect the relationship between stable isotopes and ecological analysis.
Addressed. Changed as suggested.
73 Consider to shape this part as follows: "...progressively increases at each trophic level."
Addressed. Changed as suggested.
76 , while
Addressed. Changed as suggested.
77 which exactly? (information on the carbon and nitrogen stable isotope ratios?)
Yes, info added for clarity.
78 Which community metrics? It would be helpful to clarify what metrics are being referred to (such as species diversity, trophic structure, or niche overlap?)
Metrics explained in the text as follows " …can be quantified and metrics that for example provide insights on the isotopic niche width or proxies for resources use can be derived,…"
85 can you maybe provide an example more relevant for your (freshwater) study ?
We searched relevant literature to the topic of ecological disturbance and changes of niche overlaps and to the best of our knowledge, no such literature exists. We also made it more clear in the sentence that this is an example from a disturbed ecosystem. If you have literature suggestions we are very open to those.
85-87 Since this is not a review paper, I recommend removing this sentence. If relevant, you could summarize key points from these sources, but as it stands, it doesn't contribute significantly to the understanding of your study topic.
Removed as suggested.
88 I recommend avoiding the use of "we will" when stating the study aim. Instead, consider rephrasing it as "The aim of this study is/was to..." which maintains a more formal and objective tone
Addressed, as follows: "In this study the word niche will be used in the sense of the isotopic niche,…"
89 The goal stated like this lacks clarity regarding which specific ponds and ditches are being studied, as well as the geographical regions involved. It would be beneficial to

Powered by Editorial Manager® and ProduXion Manager® from Aries Systems Corporation

	specify the study locations or the regions of interest to provide context. Consider revising the sentence to include this information, such as: "...compare the isotopic niche widths of benthic macroinvertebrate groups and communities in ponds and ditches from [specific regions or habitats], using a geometric approach (22), to assess the impact of agricultural stressors, such as pesticide residues, on these niche metrics. Throughout the review process, it was stated more clearly in the introduction what are the differences between ponds and ditches and why we are investigating, those water body types. For example the paragraph was changed in the text as follows: "We compared niche widths of FFGs and the pond and ditch communities based on a geometric approach originally developed by (25) to examine the isotopic niches of benthic invertebrates in ponds and ditches. The aim is to assess the influence of agricultural stressors (toxicity of water samples due to pesticide residues and eutrophication due to nutrient inputs) on the isotopic niche metrics.<br>We assume that ponds and ditches are affected differently by agricultural stressors, affecting habitat availability and quality and thus influencing benthic invertebrate food webs and niche size (33,34). Ditches are expected to be more strongly influenced by agricultural stressors than ponds due to their characteristics, which are a rather homogeneous habitat in and around the water body, a directly farmed environment with the location in the field (grassland) without a riparian buffer. Ditches, for example, are probably more contaminated, but as they are part of a flow system, pollutants can also be transported downstream. Ponds, on the other hand, are better protected from pesticide inputs, but can act as a source and sink for pesticide residues in the sediment (35). The water body type's ponds and ditches are used as gradients of agricultural effects, including nutrient enrichment, pesticides, and the propo...
**Additional Information:**	
**Question**	**Response**
**Financial Disclosure**<br><br>Enter a financial disclosure statement that describes the sources of funding for the work included in this submission. Review the submission guidelines for detailed requirements. View published research articles from *PLOS ONE* for specific examples.<br><br>This statement is required for submission and **will appear in the published article** if the submission is accepted. Please make sure it is accurate.<br><br>**Funded studies**<br>Enter a statement with the following details:<br>• Initials of the authors who received each award<br>• Grant numbers awarded to each author<br>• The full name of each funder<br>• URL of each funder website<br>• Did the sponsors or funders play any role in the study design, data collection and analysis, decision to publish, or preparation of the manuscript?	Yes

Did you receive funding for this work?	
Please add funding details.<br>as follow-up to "**Financial Disclosure**<br><br>Enter a financial disclosure statement that describes the sources of funding for the work included in this submission. Review the submission guidelines for detailed requirements. View published research articles from *PLOS ONE* for specific examples.<br><br>This statement is required for submission and **will appear in the published article** if the submission is accepted. Please make sure it is accurate.<br><br>**Funded studies**<br>Enter a statement with the following details:<br>• Initials of the authors who received each award<br>• Grant numbers awarded to each author<br>• The full name of each funder<br>• URL of each funder website<br>• Did the sponsors or funders play any role in the study design, data collection and analysis, decision to publish, or preparation of the manuscript?<br><br>Did you receive funding for this work?"	This work was funded by the German Federal Ministryof Food and Agriculture (BMEL) via the Agency for Renewable Resources (FNR; project number22012018) based on a decision of the Parliament of the Federal Republic of Germany.
Please select the country of your main research funder (please select carefully as in some cases this is used in fee calculation).<br>as follow-up to "**Financial Disclosure**<br><br>Enter a financial disclosure statement that describes the sources of funding for the work included in this submission. Review the submission guidelines for detailed requirements. View published research articles from *PLOS ONE* for specific examples.<br><br>This statement is required for submission and **will appear in the published article** if the submission is accepted. Please make	GERMANY - DE

sure it is accurate.<br><br>**Funded studies**<br>Enter a statement with the following details:<br>• Initials of the authors who received each award<br>• Grant numbers awarded to each author<br>• The full name of each funder<br>• URL of each funder website<br>• Did the sponsors or funders play any role in the study design, data collection and analysis, decision to publish, or preparation of the manuscript?<br><br>Did you receive funding for this work?"	
**Competing Interests**<br><br>Use the instructions below to enter a competing interest statement for this submission. On behalf of all authors, disclose any competing interests that could be perceived to bias this work—acknowledging all financial support and any other relevant financial or non-financial competing interests.<br><br>This statement is required for submission and **will appear in the published article** if the submission is accepted. Please make sure it is accurate and that any funding sources listed in your Funding Information later in the submission form are also declared in your Financial Disclosure statement.<br><br>View published research articles from *PLOS ONE* for specific examples.	The authors have declared that no competing interests exist.

**NO authors have competing interests**<br><br>Enter: *The authors have declared that no competing interests exist.*<br><br>**Authors with competing interests**<br><br>Enter competing interest details beginning with this statement:<br><br>*I have read the journal's policy and the authors of this manuscript have the following competing interests: [insert competing interests here]*<br><br>* typeset	
**Ethics Statement**<br><br>Enter an ethics statement for this submission. This statement is required if the study involved:<br><br>• Human participants<br>• Human specimens or tissue<br>• Vertebrate animals or cephalopods<br>• Vertebrate embryos or tissues<br>• Field research<br><br>Write "N/A" if the submission does not require an ethics statement.<br><br>General guidance is provided below. Consult the submission guidelines for detailed instructions. **Make sure that all information entered here is included in the Methods section of the manuscript.**	Invertebrate tissue was collected under field permissions granted by the FInAl project (fundeb by the Agency for Renewable Resources (FNR; project number22012018)).<br><br>A species protection exemption permit in accordance with Section 45 (7) No. 3 of the Federal Nature Conservation Act (BNatSchG) for the capture and killing of invertebrates with non-specific traps for agro ecological studies in the Havelland district was issued by the Brandenburg State Office for the Environment (LfU).

Powered by Editorial Manager® and ProduXion Manager® from Aries Systems Corporation

**Format for specific study types**<br><br>**Human Subject Research (involving human participants and/or tissue)**<br>• Give the name of the institutional review board or ethics committee that approved the study<br>• Include the approval number and/or a statement indicating approval of this research<br>• Indicate the form of consent obtained (written/oral) or the reason that consent was not obtained (e.g. the data were analyzed anonymously)<br><br>**Animal Research (involving vertebrate animals, embryos or tissues)**<br>• Provide the name of the Institutional Animal Care and Use Committee (IACUC) or other relevant ethics board that reviewed the study protocol, and indicate whether they approved this research or granted a formal waiver of ethical approval<br>• Include an approval number if one was obtained<br>• If the study involved *non-human primates*, add *additional details* about animal welfare and steps taken to ameliorate suffering<br>• If anesthesia, euthanasia, or any kind of animal sacrifice is part of the study, include briefly which substances and/or methods were applied<br><br>**Field Research**<br><br>Include the following details if this study involves the collection of plant, animal, or other materials from a natural setting:<br>• Field permit number<br>• Name of the institution or relevant body that granted permission	
**Data Availability**<br><br>Authors are required to make all data underlying the findings described fully available, without restriction, and from the time of publication. PLOS allows rare exceptions to address legal and ethical concerns. See the PLOS Data Policy and FAQ for detailed information.	Yes - all data are fully available without restriction

A Data Availability Statement describing where the data can be found is required at submission. Your answers to this question constitute the Data Availability Statement and **will be published in the article**, if accepted.<br><br>**Important:** Stating 'data available on request from the author' is not sufficient. If your data are only available upon request, select 'No' for the first question and explain your exceptional situation in the text box.<br><br>Do the authors confirm that all data underlying the findings described in their manuscript are fully available without restriction?	
**Describe where the data may be found in full sentences. If you are copying our sample text, replace any instances of XXX with the appropriate details.**<br><br>• If the data are **held or will be held in a public repository**, include URLs, accession numbers or DOIs. If this information will only be available after acceptance, indicate this by ticking the box below. For example: *All XXX files are available from the XXX database (accession number(s) XXX, XXX.).*<br>• If the data are all contained **within the manuscript and/or Supporting Information files**, enter the following: *All relevant data are within the manuscript and its Supporting Information files.*<br>• If neither of these applies but you are able to provide **details of access elsewhere**, with or without limitations, please do so. For example:<br><br>*Data cannot be shared publicly because of [XXX]. Data are available from the XXX Institutional Data Access / Ethics Committee (contact via XXX) for researchers who meet the criteria for access to confidential data.*<br><br>*The data underlying the results presented in the study are available from (include the name of the third party*	The raw data on the stable isotope composition (δ13C and δ15N) of the consumers will be made available at the IsoBank repository. The data have already been submitted and are still under processing by IsoBank. Final reference to the data set will be incorporated in the manuscript during review process if the manuscript is seen egligible for PLOS ONE.

and contact information or URL).<br>• This text is appropriate if the data are owned by a third party and authors do not have permission to share the data.<br><br>* typeset	
Additional data availability information:	Tick here if the URLs/accession numbers/DOIs will be available only after acceptance of the manuscript for publication so that we can ensure their inclusion before publication.

Powered by Editorial Manager® and ProduXion Manager® from Aries Systems Corporation

Assessing agricultural effects on benthic invertebrate communities in ponds and ditches using $\delta^{15}N$ and $\delta^{13}C$ isotope niches

Authors: Fee Nanett Trau [1*], Kathrin Fisch[1], Stefan Lorenz [1]

[1] Institute for ecological chemistry, plant analysis and stored product protection, Julius Kühn-Institute, Berlin, Germany

* Corresponding author:

E-Mail: fee-nanett.trau@julius-kuehn.de (FNT)

Abstract

Agriculture is one of the dominant types of land use worldwide. The conversion of land and intensification of agriculture have severe impacts on biodiversity and the associated ecosystem functions in the production areas and in natural or semi-natural habitats embedded in the agricultural landscape. Ponds and ditches, while vulnerable to the negative impacts of agriculture, are valuable habitats for benthic invertebrates due to their heterogeneity and location in a rather homogeneous landscape and harbor a diverse community as well as endangered or rare taxa. It is challenging to detect community disturbance in these habitats due to indirect or multiple stressors from agriculture, as shown by the failure of classic structural biodiversity metrics, such as overall taxa number, Shannon or evenness index, to perform well in previous studies. Therefore, we used a functional approach to understand the influence of agricultural stressors on isotopic compositions of benthic invertebrate in ditch and pond communities. Benthic invertebrates, from six different functional feeding groups (FFGs), and water samples were collected from three ponds and four ditches in an agricultural landscape in Brandenburg, Germany. We analyzed the carbon and nitrogen stable isotope ratios of the invertebrates and the pesticides and nutrient residues of water samples. Estimates of community metrics were derived using a Bayesian approach for the two water body types and the FFGs present. A distance-based Redundancy Analysis was carried out to detect the environmental variables that show the maximum correlation with the derived metrics. Benthic invertebrates in ponds occupied a

larger total area and showed greater spacing of individuals in the isotope space than communities in ditches, indicating that more resources and/or habitats were available and utilized in ponds. No clear pattern emerged when comparing the FFGs in the two water body types, but some unexpected differences were found, for example in FFG *predator*, which was more abundant and diverse in ditches. Our study found that benthic invertebrate communities respond in complex ways to stressors in small unstable systems, which are also subject to strong fluctuations in biotic and abiotic parameters. The impacts of individual structures within each water body are more pronounced than influences based on the type of water body. However, larger datasets may yield in more pronounced patterns on effects of agriculture on stable isotope composition in ponds and ditches.

Keywords: agricultural landscape, benthic invertebrates, food web, isotope niche, small water bodies

1. Introduction

Agriculture covers about 38 % of the world's terrestrial area, which makes it the predominant land use type (1). The conversion of forests, wetlands, and natural grasslands to agricultural land has had severe impacts on the provision of habitats, biodiversity, and associated ecosystem functions (1,2). Likewise, the increasing specialization of farmers, the expansion and intensification of agriculture (e.g. by fertilization and pesticide application) further increased homogenization of the landscape, resulting in accelerated habitat fragmentation and biodiversity losses (3). Those developments continue to exert ongoing pressure on the remaining semi-natural (e.g. hedges, ponds, field margins, ditches) or natural habitats within the agricultural landscape and on the surrounding aquatic and terrestrial environments (4). Examples for those remaining semi-natural habitats and non-productive areas in the context of agriculture are water bodies such as ponds and ditches, which provide valuable habitats for aquatic and semi-aquatic species. Ponds and ditches are biodiversity hotspots and form aquatic corridors in a landscape dominated by agriculture in need of conservation action (5,6). Unfortunately, these small water are usually not included in monitoring programs (5,7), but the importance of particularly ponds, but also ditches, for benthic macroinvertebrate biodiversity and ecosystem services is increasingly gaining recognition in research activities (5,8–13). Ponds are known to harbour high species numbers (8). However, also ditches, which are usually in close proximity to agricultural activities, have the potential to host diverse communities as well as rare or endangered aquatic species (5) and show a high richness for the groups Coleoptera, Gastropoda, Trichoptera and Heteroptera (5).

To conserve and promote these aquatic habitats and their biodiversity, knowledge on the status and indicators for measuring biodiversity are indispensable. However, the assessment of water or habitat quality by benthic invertebrate biodiversity is challenging, and results from a previous study on ponds have shown that conventional structural biodiversity metrics (e.g. taxonomic diversity, Shannon index, and the EPT index, to a certain degree) do not perform well in detecting stressed communities in agricultural areas due to long-term and ubiquitous persistence of stressors and short stressor gradients (11). Additionally species occurrences may be subject to some degree of stochasticity (14) and

functional responses can reflect the ecological conditions and habitat change better than taxonomic approaches (15,16). Therefore, functional indicators and approaches could be promising tools to detect community disturbance and to examine possible direct as well as indirect effects of stressors on benthic invertebrate communities and their functions (11,17,18). Common traits used to assess the functional diversity of benthic invertebrates are feeding mode, locomotion, maximum body size, and respiration mode (19). We selected FFG to address the effects of agriculture as further stressors such as channelization and modification of physical structure do not vary in their intensity in the selected water bodies. As benthic invertebrates play an important role in the cycling of nutrients and decomposition of organic matter, the analysis of trophic relationships and functional feeding groups (FFGs) are central to understand ecosystem processes (20,21) and to visualize effects of agricultural activity on communities (17,22–24). The niche concept is fundamental to understand how communities are organized and how ecological interactions shape their strategies and ecological shifts (25). The niche of a species describes the range of conditions necessary for the species to exist and reproduce, including its abiotic and biotic interactions within the environment (26). Niches can overlap when species are co-occurring, which leads to interspecific competition. Higher species richness can lead to greater niche overlaps under environmental disturbance, as for example found in carabid communities (27). The basic principle of "you are what you eat (plus a few ‰)" (28) has led to the use of stable isotopes to analyse isotopic niches, food web structures, and species interactions in ecosystems (29). Two elements commonly used for this purpose are nitrogen and carbon. The ratio of ^{15}N to ^{14}N progressively increases at each trophic level whereas the ratio of ^{13}C to ^{12}C remains almost unchanged when passing through the food web, but discriminates food sources due to different photosynthetic pathways (28). Accordingly, $\delta^{15}N$ values are used to determine the trophic position of an organism while $\delta^{13}C$ values provide information on the sources utilized by the organism. Based on the information on carbon and nitrogen stable isotope ratios, the isotopic niche of a species or taxa group (e.g. Coleoptera, Odonata etc.) can be quantified and metrics that for example provide insights on the isotopic niche width or proxies for resource use can be derived, which are statistically

comparable over a broad spatial scale and between communities composed of different species (18,30). The isotopic niche can thus serve as a proxy for the realized ecological niche in relation to the use of resources and habitat (31). The realised niches are linked to land use and agricultural stress factors, as these change the availability of resources and habitat quality. In this study, the term niche will be used in the sense of the isotopic niche as described above.

We compared niche widths of FFGs and the pond and ditch communities based on a geometric approach originally developed by (24) to examine the isotopic niches of benthic invertebrates in ponds and ditches. The aim is to assess the influence of agricultural stressors (toxicity of water samples due to pesticide residues and eutrophication due to nutrient inputs) on the isotopic niche metrics.

We assume that ponds and ditches are affected differently by agricultural stressors, affecting habitat availability and quality and thus influencing benthic invertebrate food webs and niche size (32,33). Ditches are expected to be more strongly influenced by agricultural stressors than ponds due to their characteristics, which are a rather homogeneous habitat in and around the water body, a directly farmed environment with the location in the field (grassland) without a riparian buffer. Ditches, for example, are probably more contaminated, but as they are part of a flow system, pollutants can also be transported downstream. Ponds, on the other hand, are better protected from pesticide inputs, but can act as a source and sink for pesticide residues in the sediment (34). The water body type’s ponds and ditches are used as gradients of agricultural effects, including nutrient enrichment, pesticides, and the proportion of surrounding crops to test our hypotheses. We asked the following questions, using ponds and ditches. Can the isotopic niches depict the effects of agriculture on benthic invertebrate communities in the selected water bodies in the study region? Are the observed patterns of isotopic niches of FFGs comparable in ponds and ditches for the FFGs? ~~To answers these questions,~~ we hypothesize that agricultural activity and low habitat variability in ditches have led to simplified trophic structures, which will be shown by wider isotopic niches, reduced niche differentiation, and larger niche overlap due to reduced differentiation of the FFGs and more generalists present.

2. Methods and data

2.1 Study area

Sampling was carried out in Brandenburg in the "Havelländisches Luch" region (Fig 1). The wet lowlands in the region were drained and an intensively maintained ditch system was created for industrial agricultural production during the 1980s (35). Traditional extensively managed and diverse grasslands were converted to seeded grasslands, often with only one type of grass, and in some cases also plowed into arable land (35). Additionally, the landscape is characterized by a large number of small standing water bodies (kettle holes, consistently named "ponds" within the context of this paper), which were formed during the last glacial period. Ponds are defined as water bodies with a maximum extent of one hectare and cover about 5 % of the arable land area over a total area of 38.000 km^2 in northeast Germany (36).

Fig 1: Map of sampling locations, ditches in blue and ponds in red. Map data: Open street map, QGIS Version 3.34.3

Samples were taken in three ponds (Fig 2) and four ditches (Fig 3) in October and November 2020. Pond 1 is entirely encompassed with trees, shrubs, and reed stands (Phragmites sp.). Ponds 2 and 3 are characterized by a mix of trees, shrubs, grasses (Poaceae), and reed (Phragmites sp.) in the riparian zone. Ditch 1 was dominated by reed (Phragmites sp.), while ditches 2 to 3 were dominated by grasses (Poaceae) in the riparian zone. See also Table 1 for descriptions of the water bodies and their surroundings.

Table 1: Description of the sampling sites. Habitats were described for the water bodies when present in the wadeable area of the shore length with a share < 5 %. The riparian zone is defined as the area from the edge of the field to the waterline where vegetation was estimated as a percentage. CPOM = coarse particulate organic matter; FPOM = fine particulate organic matter. Dominant types of riparian vegetation are written in bold.

			Riparian zone [in %]				
**Site**	**Habitats**	**Agricultural cultivation**	**Grasses**	**Herbaceous perennials**	**Trees**	**Shrubs**	**Reeds**
**Pond 1**	Reeds, CPOM, macrophytes	Grassland	0	0	25	25	**50**
**Pond 2**	Reeds, CPOM, macrophytes	Grassland	30	0	5	5	**60**
**Pond 3**	Reeds, CPOM, FPOM	Maize, cereal	0	20	10	10	**60**

**Ditch 1**	Reeds, FPOM	Grassland	20	0	0	0	**80**
**Ditch 2**	CPOM, macrophytes	Grassland	**100**	0	0	0	0
**Ditch 3**	Reeds, CPOM, FPOM	Grassland	**80**	0	0	0	20
**Ditch 4**	Reeds, FPOM, macrophytes	Grassland	**100**	0	0	0	0

Fig 2: Sampled ponds, pictures: Fee Nanett Trau, JKI

Fig 3: Sampled ditches, pictures: Fee Nanett Trau, JKI

2.2 Sampling and sample processing – invertebrates

A species protection exemption permit in accordance with Section 45 (7) No. 3 of the Federal Nature Conservation Act (BNatSchG) for the capture and killing of invertebrates with non-specific traps for agro ecological studies in the Havelland district was issued by the Brandenburg State Office for the Environment (LfU).

Benthic invertebrates were sampled using hand netting (mesh size 500 µm) from the habitats (FPOM, CPOM, submerse macrophytes, reed) present in the selected water bodies. Individuals from all FFGs present were sampled and organisms were sorted on-site by morphology (to the level of order or family). Sampling was carried out until 10 specimen of the present orders/families were collected as recommended by (30), whenever possible. All animals were kept alive for about 24 hours so that they can empty their gastrointestinal tract before analysis and then killed as quickly and humanely as possible with boiling water to avoid altering the isotopic signals through the use of ethanol. Afterwards the samples were frozen before further processing. In the laboratory, the benthic macroinvertebrates were further taxonomically determined to family (where the order was assigned on site) or genus level when possible and sorted into the FFGs based on the freshwater ecology database (37). Gastropods, Bivalves, and Trichoptera were removed from their shell or caddis and parasites were removed from afflicted Heteropterans before further sample processing to only analyse the isotopic signal of the body tissue of selected animals. In the next step, the samples were dried (48 hours at 60 °C, Memmert UFE

600). If the animal weight was >2 mg (Sartorius LA 230 P), one individual organism was ground into a powder. For lower sample weights, several individuals from one family were pooled into one sample to reach the optimum sample weight of 2 mg and then ground. Material was homogenized using a mixer mill (Retsch MM400) and stored in Eppendorf tubes (1.5 ml). For each family present, all samples were analysed with a maximum of 10 to 15 samples.

The encapsulation as well as the isotope analysis was carried out by the stable isotope laboratory at Cornell University (COIL), Department of Ecology and Evolutionary Biology, USA. Stable isotope analysis of ^{15}N and ^{13}C was performed using a Thermo Delta V isotope mass spectrometer (IRMS) interfaced to a NC2500 elemental analyser. Raw data, sample weights and quality control values for presented data can be obtained here (insert reference to IsoBank repository, status in submission). Isotope corrections were performed using linear regression of all δ values using two additional in-house standards (38). Results were reported as δ values as ratios of ^{13}C:^{12}C relative to Vienna PeeDee Belemnite and ^{15}N:^{14}N relative to atmospheric air (Eq. 1). Measurements were carried out from April 2021 to July 2022 in four batches.

$$\delta X = \left[\left(R_{sample}/R_{standard}\right) - 1\right] * 10^3 \quad (1)$$

Where X is ^{13}C or ^{15}N, R is the corresponding ratio ^{13}C /^{12}C or ^{15}N /^{14}N. Increased δ values show an increase of the heavy isotope in the sample (39).

2.3 Sampling and sample processing - water

Environmental chemical parameters were measured to identify potential variables that could function to explain the community's food web structures or isotopic niches of groups. Water samples were taken via grab sampling. A mixed water sample of approximately 5 L was taken from four to six locations around the shoreline of the ponds or along a stretch of 50 m from the ditches.

Environmental water parameters [oxygen content, electrical conductivity (EC), pH, and temperature] were measured in-situ using a portable meter (WTW Multi 3430 IDS with OxiCal-SL FDO 925, TetraCon 925 and SenTix 940 probes, Germany). Samples for pesticide analysis were partitioned into two 500 mL

glass bottles, with one bottle holding 50 mL of dichloromethane for pyrethroid analysis. The water samples were analysed using multi-methods validated for analysis of 99 active substances (S1 Table).

Except for the pyrethroids, the selected active substances in the water samples were quantified by liquid chromatography mass spectrometry (LC-ESI-MS/MS) using the internal standard method after enrichment and clean-up via solid phase extraction (Chromabond HR-P, Macherey-Nagel, Germany, 50-100 µm, 3 mL, 200 mg). For LC-MS/MS analysis a 1290 Infinity II LC system (Agilent, USA) coupled to a QTRAP 6500+ mass spectrometer (SCIEX, USA) was used.

After concentration by liquid–liquid extraction of the sample, pyrethroids were analysed by gas chromatography-mass spectrometry (GC-MS) using a Trace GC Ultra coupled to a TSQ Quantum GC XLS- mass spectrometer (Thermo Electron Corporation, USA). All pesticide analysis were done accordingly to (11).

Samples for nutrient analysis were filtered through a 0.45 µm cellulose acetate filter before analysis. The concentrations of NH_4^+ + and PO_4^{3-}were measured photometrically using a spectrophotometer (DR/2000 Hach) according to DIN 38 406–5 and EN ISO 6878:2004, respectively. NO_3^- and NO_2^- concentrations were analysed by high-performance liquid chromatography (HPLC, Agilent 1100 Series HPLC System DAD, USA).

2.3.1 Toxicity calculations

To calculate the toxicity of the analysed water samples for invertebrates and algae based on measured pesticide residue concentrations, the toxic pressure for each sample is calculated as the sum of toxic units; TU_{sum} (Eq. 2). Toxic units were calculated for the pesticide concentrations based on half-maximal effective concentration (EC50) or 50% lethal concentration (LC50) values for invertebrates and algae. Analyzed pesticide concentrations were divided by the concentration for the most sensitive test organism and based on 48 h EC50 values for *Daphnia magna* or 96 h LC50 values for *Chironomus* sp. for the invertebrate toxicity. Toxicity calculations for algae are based on 72 h EC50 values for mostly, *Raphidocelis subcapitata (*syn: *Pseudokirchneriella subcapitata*) and *Scenedesmus subspicatus*. Data

on the EC50 and LC50 values was obtained from the Pesticides Properties Database (40) or Ecotox database (41). See also S1 table for details on substances measured and the test organisms.

$$TU_{sum} = \log \sum_{i=1}^{n} \left(\frac{C_i}{EC_{50_i}/LC_{50_i}}\right) \quad (2)$$

C_i is the concentration of pesticide *i* in µg/L. Concentrations below the LOQ but above 0 were replaced with half of the LOQ.

2.4 Description of the dataset

The dataset contained $\delta^{13}C$ and $\delta^{15}N$ data covering six FFGs (Table 2) from 13 orders (see S5 for details). FFGs were assigned to the individuals according to the family determination, if available, otherwise by order (32,37,42,43). Classified *as collector/filterer* are Bivalvia and the family Culicidae, which filter suspended FPOM from the water column (37). The families of Ephemeroptera and some dipterans belong to the group *collector/gatherers*, which feed on sedimented FPOM. *Grazers/scrapers* feed on algae tissue or biofilm growing on surfaces, also they feed on algae or plant tissue (37). In the present dataset *grazers/scrapers* are mostly gastropods e.g. of the families Lymnaeidae or Planorbidae. Within the group *omnivore*, families are gathered that could not be assigned to one distinct group. In the present dataset Chironomidae, Corixidae, Haliplidae and Stratiomyidae were assigned as *omnivore*. *Predators* hunt on prey and examples are Dytiscidae, Ceratopogonidae or Coenagrionidae. Typical *shredders* are crustaceans like Gammaridae or Asselidae, which feed on plant tissue, fallen leaves or CPOM (37). A detailed list of the families belonging to the FFGs can be found in the table supporting information 5.

Table 2: Number of samples available and used for analysis for each water body and the FFGs.

FFG	Pond 1	Pond 2	Pond 3	Ditch 1	Ditch 2	Ditch 3	Ditch 4	Sum FFG pond/ditch
**Collector/ filterer**	0	0	0	3	0	0	9	**0/12**
**Collector/ gatherer**	13	5	3	11	10	10	10	**21/41**
**Grazer/ scraper**	37	8	11	16	14	18	16	**56/64**
**Omnivore**	15	31	22	28	23	30	25	**68/106**
**Predator**	19	19	14	38	45	26	60	**52/169**

**Shredder**	24	7	0	18	21	10	12	**31/61**
**Sum type**	**Ponds = 228**			**Ditches = 453**				

2.5 Statistical methods

All data processing and statistical analyses were performed with R Studio version 4.3.1 (44) and JAGS version 4.3.1.

2.5.1 δ^{15}N correction

The influence of organic fertilizer was observed in ditches by examining the δ^{15}N values of benthic macroinvertebrates (consumers). The δ^{15}N values for ditches are higher than in ponds due to higher δ^{15}N values in resources present in these water bodies (S2 Fig.). To compare the FFGs in the two water body types this was corrected as follows: For both water body types the mean δ^{15}N value for the consumers was calculated. The means were subtracted from each other and the difference was subtracted from the δ^{15}N values of the consumers in ditches only.

2.5.2 δ^{13}C correction

The δ^{13}C values for one pond are higher due to the influence of maize cultivation (C4-plant) on the consumers (Fig. 4). For ponds 1 and 2 (no maize influence) and pond 3 (maize influence) the mean values for δ^{13}C consumers were calculated. The means were subtracted from each other and the difference was subtracted from δ^{13}C values from consumers in pond 3 only.

2.5.3 SIBER (Stable Isotope Bayesian Ellipses in R)

The R package SIBER (45) was used for the analysis of isotopic niche width of the FFGs and the comparison of food web structures between different water body types. The analysis follows a Bayesian approach to overcome uncertainties when calculating and comparing metrics (45). The estimation by Bayesian inference incorporates error from the sampling process and allows robust comparisons between groups with different and small sample sizes (30).

First the raw data was plotted for the six FFGs (*collector/filterer, collector/gatherer, grazer/scraper, omnivore, shredder,* and *predator*) and the respective water body types (S4 Fig.). Then the Layman metrics and the Bayesian inference-derived Layman.B metrics were calculated for the combination of water body and FFGs as group (37 groups) and the water body types pond and ditch as community as

well as for the FFG as group (six groups) and the water body types pond and ditch as community. To compare the groups the Welsch's t-test was used (confidence interval 95 %). For the dbRDA (see below), the Layman metrics for the seven water bodies as community and FFG as group (six) were calculated.

The described metrics are based on (24) and summarize information on the distribution of data points in the isotope space. The six metrics are the $\delta^{13}C$ range, the $\delta^{15}N$ range, the total area of the convex hull (TA), the mean distance to centroid (CD), the mean nearest neighbor distance (MNND) and the standard deviation of the nearest neighbor distance (SDNND) (30). The TA describes the niche width, CD provides information on niche width and species spacing, the MNND is a description of density and clustering of species, and the SDNND gives information on the evenness of the species density (30).

Additionally, the Bayesian standard ellipse areas (SEA.B), corrected standard ellipse areas (SEAc), and convex hull area (TA) based on Bayesian interference were calculated for the FFGs in ponds and ditches. The Bayesian estimates provide robust measures of probability and can reflect model uncertainty by averaging many possible models into one single model (46). The SEA.c is based on SEA.B, which describes isotopic niche width, and corrected for small sample sizes for unbiased analysis (30).A larger SEA corresponds to a more diverse isotopic signal and thus to wider niches. The dataset with corrected $\delta^{15}N$ and $\delta^{13}C$ values was used for the comparison of FFGs using the corrected Standard Ellipse Area (SEA.c). The data was plotted using the function *siberDensityPlot* (47).

The probability that the posterior distribution is smaller or larger for a group can be estimated by calculation of the SEA.B niche overlap. SEA.B overlaps between ponds and ditches were calculated for the FFGs based on the maximum likelihood estimates of the means and the covariance matrices of each group (function: *maxLikOverlap* calculated for the proportion of both overlaps, p-interval of 0.95). The calculation of the Bayesian Layman metrics (Layman.B) was performed for the data set with corrected $\delta^{15}N$ values.

Bayesian models were run with JAGS and model parameters were defined according (30) to fit the ellipses.

2.5.4 Multivariate statistics

Environmental variables were standardized using the *scale* function of the *base* package (48), whereby the mean value was set to zero and scaled to unit variance. Variable selection for the dbRDA was carried out by the *bioenv* function from the *vegan* package (49). This function detects the best environmental variable subset in a way such that the environmental variables show the maximum rank correlation (method: Spearman) with the Layman metrics matrix. The dissimilarity index for the Layman metrics matrix used was "mahalanobis", a measure that accounts for correlations across multiple variables. Selected environmental variables, excluding the variables EC and O_2 due to small sample size and variability of measures during the course of the day, were used for the dbRDA, which can show if an explanatory variable has significant impact on the response variables and how much of the variation is explained by each axis. (function *dbrda*, package *vegan*, 49). Selected variables are: nutrient concentration sum, number of pesticide detections, TU_{sum} for algae, and TU_{sum} for invertebrates. To test for the significance of the model, a permutation test on terms and axes was carried out ($p \leq 0.05$; function *anova.cca*, package *vegan*, 49). The calculation of the distance-based Redundancy Analysis (dbRDA) was performed for the data set with corrected $\delta^{15}N$ values.

3. Results

Results for the environmental parameters, nutrient concentration and toxicities of the water samples measured to describe the quality of the water bodies (Table 3).

Table 3: Overview of the measured water quality and environmental parameters for the seven water bodies. EC = electric conductivity

Sample	*C °*	*EC [µS/cm]*	*pH*	*O_2 [mg/L]*	*NH_4^+ [mg/L]*	*NO_2^- [mg/L]*	*NO_3^- [mg/L]*	*PO_4^{3-} [mg/L]*	*NH_4^+ NO_2^- +NO_3^- [mg/L]*	*TUsum algae*	*TUsum inverte-brates*	*No. of habi-tats*	*Pesticide detec-tions*
Pond 1	7.4	1570	6.2	5.2	0.01	0	0.2	0.25	0.21	-2.686	-5.221	3	3
Pond 2	10.5	1112	6.5	10.6	0.09	0	0.14	0.05	0.23	-4.057	-5.074	2	1
Pond 3	11.2	881	7	13.8	0.08	0	0.23	0.11	0.31	-2.650	-4.632	3	4

Ditch 1	9.7	588	7	11.5	0.86	0	1.1	0.44	1.96	-2.685	-5.428	2	4
Ditch 2	8.3	815	7.6	6.1	0.08	0	0.72	0.06	0.8	-3.253	-5.116	1	6
Ditch 3	11	928	7	8.8	0.08	0	0.36	0.06	0.44	-2.614	-4.817	3	7
Ditch 4	12.7	900	8	8.8	0.08	0	1.32	0.03	1.4	-3.456	-4.925	2	7

The raw data on the stable isotope composition ($\delta^{13}C$ and $\delta^{15}N$) of the consumers and sampled resources are available at IsoBank repository (in submission).

3.1 Isotopic niches of communities in ponds and ditches

After correction for the influence of organic fertilizers and of maize, the $\delta^{13}C$ and $\delta^{15}N$ values of consumers show a similar range in ditches and in ponds (Fig. 4 and S2 Fig.). The biplot shows that ponds have a slightly larger ellipse area but that the water body types are comparable in general (Fig. 4).

Fig 4: Biplots for the macroinvertebrate data. The left plot is corrected for $\delta^{15}N$ influence of organic fertilizers on ditches. The right plot is corrected for $\delta^{15}N$ as well as $\delta^{13}C$ values for the influence of C4-plant maize on pond 3. Ellipses contain 40% of the data.

The posterior mode of the $\delta^{15}N$ range is comparable between the two water body types (after correction for organic fertilization) (Fig. 5 a). The typical range of $\delta^{13}C$ values is statistically significant distinct in the two water body types and larger in ponds (Fig. 5 b). The posterior distribution of TA and thus total niche width and the spacing of individuals - CD is statistically significantly larger in pond communities compared to communities in ditches (Fig. 5 b, e). The posterior values for the MNND (Fig. 5 c) and thus clustering of individuals within the community is larger in ponds, where the evenness of density is similar in ponds and ditches (Fig. 5 f).

Fig 5: SIBER density plots for the Layman metrics computed via Bayesian statitics, based on the dataset corrected for $\delta^{15}N$. Black dots show the modes. Shaded boxes represent the 50 %, 75 %, and 95 % confidence intervals, from dark to light red/blue. Grouping is based on a combination of the individual small water body (7 SWB) and the six FFGs (in total 37 groups), the communities are assigned to the SWB types (pond, ditch). $\delta^{15}N$ values of the ditches are corrected for the influence of organic fertilizers.

For an illustration of the Layman.B metrics calculated for both stable isotopes corrected, see also table S3.

3.2 Comparison of functional feeding groups

There are observable differences for the SEA.B modes and confidence intervals between FFGs, but there is no pattern visible when comparing FFGs in ponds and ditches. SEA.B and SEAc modes for

shredders are higher in ditches than in ponds (Fig. 6). The groups *omnivore* and *grazer/scraper* have larger SEA.B/SEAc modes in ponds (Fig. 6). Values for *predators* and *collector/gatherers* are similar in the two water body types (Fig. 6). The credible intervals for *predators* in ponds and ditches are rather small, which shows that the uncertainty is low and that the dataset provides a precise representation of the population mean for this group. P*redators* were in general more abundant and diverse in ditches. Six taxa groups and 13 families and were recorded there, while five taxa groups and eight families were recorded in ponds (S5 Table).

Fig 6: SIBER density plots computed via Bayesian analysis using corrected $\delta^{15}N$ and $\delta^{13}C$ values. Plots are shown for the FFGs in ponds (red) and ditches (blue). Black dots are the modes of SEA.B. The grey X indicates the size-corrected standard ellipse area (SEAc) mode. Shaded boxes represent the 50 %, 75 %, and 95 % confidence intervals.

SEA.B niche overlaps for the FFGs between ponds and ditches vary between 15 and 22.9 % (corrected only for $\delta^{15}N$) and between 22.9 to 44.7 % when the data is corrected for $\delta^{15}N$ and $\delta^{13}C$ (Table 4). For *shredders* the overlap is the same for both correction approaches, as there were no *shredders* found in the maize pond and thus no $\delta^{13}C$ correction was needed. For the corrected for $\delta^{15}N$ and $\delta^{13}C$ values, niche overlaps for *predators* (44.7 %) are largest in ponds and ditches. *Shredders* show least similarity in niches between ponds and ditches (22.9 %).

Table 4: Niche overlaps of ponds and ditches for the FFGs calculated for two datasets. The overlap for* collector/filterer *cannot be calculated due to their absence in pond samples.

Functional Feeding Group	Niche overlaps of ponds and ditches in %, corrected for N values	Niche overlaps of ponds and ditches in %, corrected for C and N values
Collector/gatherer	15	28.4
Grazer/scraper	16.9	29.8
Omnivore	18.6	38.6
Predator	20	44.7
Shredder	22.9	22.9

3.3 Effects of agricultural stressors on the observed results

Water quality parameters measured in the water samples indicate a low number of pesticides in ponds (M = 2.7 µg/L, SD = 1.5 µg/L) and high numbers of pesticides in ditches (M = 6 µg/L, SD = 1.4 µg/L). 81 % of the total pesticides detected (ponds and ditches) are herbicides and 19 % are fungicides. Furthermore, nutrient concentration sum is larger in ditches (M = 1.15, SD = 0.7 mg/L) compared to ponds (M = 0.25, SD = 0.05 mg/L). Dissolved oxygen concentrations vary from 5.2 mg/L to 13.8 mg/L

in ponds and from 6.1 mg/L to 11.5 mg/L in ditches. EC varies from 881 to 1570 µS/cm in ponds and from 588 to 928 µS/cm in ditches.

The dbRDA plot does not show a clear pattern for ponds and ditches (Fig. 7). The isotopic composition of benthic invertebrates and the resulting Layman metrics of pond 3 and 2 are influenced by the toxicity of water samples for invertebrates (TUsum_invert). Pond 1 and the ditches, except ditch 2, are influenced by the toxicity of water samples for algae (TUsum_algae), the concentration of nutrients (nutrients_sum), and the number of pesticides found (N_pesticides). Constrained variables at the first axis explain 75 % of the variance. However, the permutation test resulted in no statistical difference ($p \leq 0.05$) for the tested model, model parameters, or axes.

Fig 7: dbRDA plot, distance measure = Euclidean. Water bodies are plotted according to the Layman metrics calculated for the stable isotope results of the communities. Environmental parameters with significant correlations are plotted in blue. The length of the arrows shows how much the variable correlates with the water bodies.

4. Discussion

The two water body types were in general comparable after the correction of $\delta^{15}N$ for the influence of organic fertilizer, which was clearly visible in consumers in ditches. Such relative enrichment was also found by (50) in urban and agricultural streams compared to woodland streams. However, within the agricultural affected sites studied here, isotopic niches could not depict benthic invertebrate community changes except for general impacts on basal C and N resources (e.g. by fertilization or maize/C4 cropping). Also, the comparison of the isotope niches of the two water body types, pond and ditch, did not reveal any clear patterns when analysing the FFGs.

The hypothesis that we expect simplified trophic structures in ditches due to agricultural activity and low habitat variability can only be partially confirmed. The finding that the likely distribution of total niche width and distances between the individuals is greater in pond communities than in ditch communities indicates that more resources/habitats are available in ponds, which lead to more extreme isotope ratios (31). Furthermore, consumers are more divergent in ponds in terms of the isotopic niche MNND, while the evenness of density SDNND is similar in ponds and ditches. This

indicates less clustering in pond communities at the same evenness level, which in turn indicates more available resources and/or habitats in ponds that were evenly utilized and colonised (51).

Even though there were no clear patterns visible when comparing FFGs, some differences were pronounced. Striking was the finding that contrary to our expectations, the FFG *predator* was more diverse in ditches. The high diversity of *predators* in ditches could be explained by the availability of a high number of primary producers facilitated by high nutrients inputs and thus high primary production, which leads to more available prey (52,53). The influence of changes on the base of the food web propagated to higher trophic levels was also observed by (52), where nutrient pollution promoted biofilm production and lead to an increase in herbivory and carnivory by omnivores. This positive influence of fertilization might be explained by intermediate but not high nutrient enrichment, as in the case of (54), which found highest densities of invertebrates at intermediate levels of nutrient enrichment (primarily of phosphate). The high species number is probably stimulated by bottom-up effects of nutrient enrichment (50,55). Although we can assume that the ditches have been enriched for several years, the effects of long-term enrichment predicted by (55), namely the reduction in species numbers and the shift in community structure, were not observed in the ditch system studied when we compare it to the ponds.

However in contrast to results from (52), the nutrient pollution in ditches did not lead to more homogenous communities for the FFG *predator* in ditches compared to ponds, instead the opposite is the case. In ditches, the number of recorded families belonging to the group of predators is nearly twice as high compared to ponds. Therefore, functional redundancy for this feeding type can be assumed (56) which is necessary to ensure resilient systems (57).

The *omnivore* FFG expressed a variety of feeding strategies. In pond 2 they were clearly mostly predatory with two clusters formed. One cluster formed by individuals with the highest $\delta^{15}N$ values in that water body and a second cluster with the $\delta^{15}N$ values on the same level as the FFG *predators* in that pond (S 4 Table). In the ditches 2, 3 and 4 they were predatory as well but $\delta^{15}N$ values were on the same level as the values of the predators. Some individuals classified as *omnivore* showed very low

$\delta^{15}N$ values which indicates an herbivorous feeding preference. Clusters on the upper and lower level of $\delta^{15}N$ values are visible in four water bodies (two ditches and two ponds) which shows the shift of omnivores towards either herbivorous or predatory lifestyles as stated by (52). However, according to (52), this shift occurs in polluted water bodies, thus also the ponds show signs of communities under the influence of agricultural stressors.

The FFG *collector/gatherer* was dominated by ephemeropterans (*Cloeon* sp.) in both water body types which explains the similarity of SEA.c modes in the water body types and shows that this FFG is similar in its resource use in both water body types.

The larger SEA.c mode for FFG *grazer/scraper* in ponds could be explained by a larger number of microhabitats and/or more resources available in ponds, which had a more diverse habitat in and around the water body compared to the ditches in this study. For lake ecosystems the lower diversity of littoral habitats was also found to be associated with lower numbers of trophic links and less resource and consumer diversity (58). This would mean that the nutrient-rich and thus primary producer-rich ditches are not diverse in resources but that some resources are very abundant. Additionally, the larger number of microhabitats and/or more resources used can also explain commonly observed patterns of higher taxa richness and the associated possible different preferences in ponds (7–9). Two *grazer/scraper* taxa groups (Diptera, Gastropoda) and five families were found in ponds, but only one *grazer/scraper* taxa group (Gastropoda) and three families in ditches. In eutrophic waters cyanobacteria blooms can occur which are toxic for other primary producers and can lower the number of *grazers*, but the concentration of cyanobacteria was high for one pond only (data not shown). This could be a measurement of a peak value that had already been exceeded in the other waters, as cyanobacterial blooms can occur very quickly.

The absence of a clear pattern is also underlined by the dbRDA. The configuration of the water bodies shows that they are influenced individually by different parameters, and other factors which were probably not detected. In general, the composition of benthic invertebrates and the resulting Layman metrics appear to be influenced either at the production level (indicated by the variables nutrients

sum, toxicity for algae, and number of pesticides detected (mainly herbicides)) or at the consumer level (indicated by the toxicity for invertebrates). Ponds 2 and 3 are similar to each other and are largely influenced on the consumer level. The four ditches and pond 1 are more strongly influenced at the primary producer level, so bottom-up effects are also probably stronger here.

Small and uneven sample sizes are principally of disadvantage, but the Bayesian statistic approach with the SEA.c should enable an unbiased, robust analysis (30). Nonetheless, the FFGs *omnivore* in ponds and *collector/filterer* in ditches show wide credible intervals that indicate a high degree of opportunism and variability in their feeding strategies which is consistent for the FFG *omnivore*. For the FFG *collector/filterer* further sampling would be needed to predict modes with less uncertainty.

The FFG *collector/filterer* was sampled with below three individuals in ponds thus the data was not used in the analysis. Also in ditches their occurrence was low (10 Bivalvia, 2 Diptera, see S5 Table). It is only possible to speculate about the reasons, but potential factors could be: i) time of sampling, low occurrence of collector/filterer in November as their food base is low, ii) hydrological conditions and sedimentation in ponds which are not favourable for e.g. Bivalvia. As *collector/filterer* were present in low numbers in both ponds and ditches (only in the ponds excluded from the analysis), we did not discuss this further.

Additionally, the time of sampling (autumn) has to be taken into account and has most probably influenced the detection of taxa and pesticides. Information on community composition may have been skewed toward a less diverse composition, with lower numbers of especially Ephemeroptera, Trichoptera, and Diptera.

Although higher numbers of pesticides were found in ditches compared to ponds, this impact does not transport into the expected toxicity gradient for algae and invertebrates, presumably due to the application of low-toxicity pesticide products by the farmers. Additionally, (59) recently found patterns of synchronous toxicity levels towards algae and aquatic plants between water bodies with a comparable distance to each other like in our study. This should be accounted for in further studies,

and future sampling between the months of April and June is generally recommended (59,60). Furthermore, the high explanatory power of 75 % of the constrained variables on the first axis most likely resulted from running the model on a small dataset and with a high number of explanatory variables.

5. Conclusion

With the obtained dataset, we were unable to recognize clear patterns of the effects of agriculture on isotopic niches of the benthic invertebrates. Agricultural activity and a homogenous environment did not lead to simplified structures in the ditches compared to ponds. Ecological systems respond in complex ways to the presence of abiotic and biotic stressors. Particularly small systems are unstable and exposures are often unclear, leading to ambiguous responses and uncertain patterns. The individual water bodies are influenced by different parameters, and the impacts of individual structures within each water body are more pronounced than influences based on the type of water body, at least for the dataset present. Larger dataset are promising options to overcome this issues, which to our knowledge are not available for small water bodies in the agricultural landscapes in Europe. Additionally, selected water bodies should be monitored more closely and analysed on a broader temporal scale to detect potential effects of agriculture on benthic invertebrate communities over time.

Acknowledgments

The authors thank Marlen Heinz, Manuel König, Karin Meinikmann, Maren Stockmann, and Elke Zeidler for their assistance during the field sampling campaign. Furthermore, we thank Dominique Conrad, Michael Glitschka, Christine Reichmann, Ina Stachewicz, and Gabriele Smykalla for the sample preparation and pesticide and nutrient analysis. In addition, we thank Dominique Conrad and Jörg Stauch for their help in preparing the biological samples. Thanks also to Matthias Stähler for discussions on analytical methods and Mario Brauns, Kristin Scharnweber, René Sahm, and Matthias Pilecky for fruitful discussions on data analysis. Furthermore, we thank Holly Blevins for proofreading.

References

1. Foley JA, Ramankutty N, Brauman KA, Cassidy ES, Gerber JS, Johnston M, et al. Solutions for a cultivated planet. Nature 2011;478(7369):337–42. PubMed PMID: 21993620.
2. Tscharntke T, Klein AM, Kruess A, Steffan-Dewenter I, Thies C. Landscape perspectives on agricultural intensification and biodiversity – ecosystem service management. Ecol Lett 2005;8(8):857–74.
3. Matson PA, Parton WJ, Power AG, Swift MJ. Agricultural intensification and ecosystem properties. Science 1997;277(5325):504–9. PubMed PMID: 20662149.
4. Stoate C, Báldi A, Beja P, Boatman ND, Herzon I, van Doorn A, et al. Ecological impacts of early 21st century agricultural change in Europe--a review. J Environ Manage 2009;91(1):22–46. PubMed PMID: 19717221.
5. Hill MJ, Chadd RP, Morris N, Swaine JD, Wood PJ. Aquatic macroinvertebrate biodiversity associated with artificial agricultural drainage ditches. Hydrobiologia 2016;776(1):249–60.
6. Tews J, Brose U, Grimm V, Tielbörger K, Wichmann MC, Schwager M, et al. Animal species diversity driven by habitat heterogeneity/diversity: the importance of keystone structures. Journal of Biogeography 2004;31(1):79–92.
7. Biggs J, Fumetti S von, Kelly-Quinn M. The importance of small waterbodies for biodiversity and ecosystem services: implications for policy makers. Hydrobiologia 2017;793(1):3–39.
8. Williams P, Whitfield M, Biggs J, Bray S, Fox G, Nicolet P, et al. Comparative biodiversity of rivers, streams, ditches and ponds in an agricultural landscape in Southern England. Biological Conservation 2004;115(2):329–41.
9. Davies BR, Biggs J, Williams PJ, Lee JT, Thompson S. A comparison of the catchment sizes of rivers, streams, ponds, ditches and lakes: implications for protecting aquatic biodiversity in an agricultural landscape. Hydrobiologia 2008;597(1):7–17.
10. Darwall W, Bremerich V, Wever A de, Dell AI, Freyhof J, Gessner MO, et al. The Alliance for Freshwater Life : A global call to unite efforts for freshwater biodiversity science and conservation. Aquatic Conserv: Mar Freshw Ecosyst 2018;28(4):1015–22.
11. Trau FN, Fisch K, Lorenz S. Habitat type strongly influences the structural benthic invertebrate community composition in a landscape characterized by ubiquitous, long-term agricultural stress. Inland Waters 2023:1–13.
12. Lynch AJ, Cooke SJ, Arthington AH, Baigun C, Bossenbroek L, Dickens C, et al. People need freshwater biodiversity. WIREs Water 2023.
13. Fehlinger L, Misteli B, Morant D, Juvigny-Khenafou N, Cunillera-Montcusí D, Chaguaceda F, et al. The ecological role of permanent ponds in Europe: a review of dietary linkages to terrestrial ecosystems via emerging insects. Inland Waters 2023;13(1):30–46.
14. Krynak EM, Yates AG. Benthic invertebrate taxonomic and trait associations with land use in an intensively managed watershed: Implications for indicator identification. Ecological Indicators 2018;93:1050–9.
15. Dolédec S, Phillips N, Scarsbrook M, Riley RH, Townsend CR. Comparison of structural and functional approaches to determining landuse effects on grassland stream invertebrate communities. Journal of the North American Benthological Society 2006(25 (1)):44–60.
16. Villéger S, Ramos Miranda J, Flores Hernández D, Mouillot D. Contrasting changes in taxonomic vs. functional diversity of tropical fish communities after habitat degradation. Ecol Appl 2010;20(6):1512–22. PubMed PMID: 20945756.
17. Kemp VA, Grey J, Hemprich-Bennett D, Rossiter SJ, Lewis OT, Wilkinson CL, et al. Changes in trophic ecology of mobile predators in response to rainforest degradation. Journal of Applied Ecology 2023;60(6):1139–48.

18. Múrria C, Iturrarte G, Gutiérrez-Cánovas C. A trait space at an overarching scale yields more conclusive macroecological patterns of functional diversity. Global Ecol. Biogeogr. 2020;29(10):1729–42.
19. Ao S, Chiu M-C, Lin X, Cai Q. Trait selection strategy for functional diversity in freshwater systems: A case framework of macroinvertebrates. Ecological Indicators 2023;153:110450.
20. Zinkann A-C, Wooller MJ, O'Brien D, Iken K. Does feeding type matter? Contribution of organic matter sources to benthic invertebrates on the Arctic Chukchi Sea shelf. Food Webs 2021;29:e00205.
21. Bearhop S, Adams CE, Waldron S, Fuller RA, Macleod H. Determining trophic niche width a novel approach using stable isotope analysis. Journal of Animal Ecology 2004(73):1007–12.
22. Bonnaffé W, Danet A, Leclerc C, Frossard V, Edeline E, Sentis A. The interaction between warming and enrichment accelerates food-web simplification in freshwater systems. Ecol Lett 2024;27(8):e14480. PubMed PMID: 39096032.
23. Guzman I de, Elosegi A, Schiller D von, González JM, Paz LE, Gauzens B, et al. Treated and highly diluted, but wastewater still impacts diversity and energy fluxes of freshwater food webs. J Environ Manage 2023;345:118510. PubMed PMID: 37390732.
24. Layman CA, Quattrochi JP, Peyer CM, Allgeier JE. Niche width collapse in a resilient top predator following ecosystem fragmentation. Ecol Lett 2007;10(10):937–44. PubMed PMID: 17845294; PubMed Central PMCID: PMC2040226.
25. Leibold MA. The Niche Concept Revisited: Mechanistic Models and Community Context. Ecology 1995(76(5)):1371–82.
26. Polechová J, Storch D. Ecological Niche. In: Encyclopedia of Ecology: Elsevier; 2019. p. 72–80.
27. Giménez Gómez VC, Verdú JR, Gómez-Cifuentes A, Vaz-de-Mello FZ, Zurita GA. Influence of land use on the trophic niche overlap of dung beetles in the semideciduous Atlantic forest of Argentina. Insect Conserv Diversity 2018;11(6):554–64.
28. DeNiro MJ, Epstein S, editors. You are what you eat (plus a few‰): the carbon isotope cycle in food chains. Geological Society of America - Abstracts with Programs; 1976.
29. Post DM. Using stable isotopes to estimate trophic position: Models, methods and Assumptions. Ecology 2002;83(3):703–18.
30. Jackson AL, Inger R, Parnell AC, Bearhop S. Comparing isotopic niche widths among and within communities: SIBER - Stable Isotope Bayesian Ellipses in R. J Anim Ecol 2011;80(3):595–602. PubMed PMID: 21401589.
31. Newsome SD, Del Martinez Rio C, Bearhop S, Phillips DL. A niche for isotopic ecology. Frontiers in Ecology and the Environment 2007;5(8):429–36.
32. Parreira de Castro DM, Reis de Carvalho D, Pompeu PdS, Moreira MZ, Nardoto GB, Callisto M. Land Use Influences Niche Size and the Assimilation of Resources by Benthic Macroinvertebrates in Tropical Headwater Streams. PLoS One 2016;11(3):e0150527. PubMed PMID: 26934113; PubMed Central PMCID: PMC4774910.
33. Brauns M, Kneis D, Brabender M, Weitere M. Habitat availability determines food chain length and interaction strength in food webs of a large lowland river. River Research & Apps 2022;38(2):323–33.
34. Chaumet B, Probst J-L, Eon P, Camboulive T, Riboul D, Payré-Suc V, et al. Role of Pond Sediments for Trapping Pesticides in an Agricultural Catchment (Auradé, SW France): Distribution and Controlling Factors. Water 2021;13(13):1734.
35. Hartong H, Schmid C, Kornack I, Wolter A. Landschaftsrahmenplan Landkreis Havelland: Band 2 Bestand und Bewertug. Landkreis Havelland; 2014.
36. Kalettka T, Rudat C. Hydrogeomorphic types of glacially created kettle holes in North-East Germany. Limnologica 2006;36(1):54–64.

37. Schmidt-Kloiber A, Hering D. www.freshwaterecology.info – An online tool that unifies, standardises and codifies more than 20,000 European freshwater organisms and their ecological preferences. Ecological Indicators 2015;53:271–82.
38. COIL. Data Interpretation Guide for Isotopic Analysis; 2021. Available from: www.cobsil.com.
39. Peterson BJ, Fry B. Stable Isotopes in Ecosystem Studies. Annual Review of Ecology and Systematics 1987:293–320.
40. Lewis KA, Tzilivakis J, Warner DJ, Green A. An international database for pesticide risk assessments and management. Human and Ecological Risk Assessment: An International Journal 2016;22(4):1050–64.
41. Olker JH, Elonen CM, Pilli A, Anderson A, Kinziger B, Erickson S, et al. The ECOTOXicology Knowledgebase: A Curated Database of Ecologically Relevant Toxicity Tests to Support Environmental Research and Risk Assessment. Environ Toxicol Chem 2022;41(6):1520–39. PubMed PMID: 35262228; PubMed Central PMCID: PMC9408435.
42. Gholizadeh M, Heydarzadeh m. Functional feeding groups of macroinvertebrates and their relationship with environmental parameters, case study: in Zarin-Gol River. Iranian Journal of Fisheries Sciences 2017:2532–43.
43. Ramírez A, Gutiérrez-Fonseca PE. Functional feeding groups of aquatic insect families in Latin America: a critical analysis and review of existing literature. Rev Biol Trop 2014;62 Suppl 2:155–67. PubMed PMID: 25189076.
44. R core Team. R: A language and environment for statistical computing.; 2023.
45. Jackson A, Parnell A. SIBER: Stable Isotope Bayesian Ellipses in R 2023.
46. Ellison AM. Bayesian inference in ecology. Ecol Letters 2004;7(6):509–20.
47. Jackson A, Parnell A. _SIBER: Stable Isotope Bayesian Ellipses in R_; 2023.
48. R core Team. R: A Language and Environment for Statistical Computing; 2024.
49. Oksanen J, Simpson GL, Blanchet GF, Kindt R, Legendre P, Minchin PR, et al. vegan: Community Ecology Package; 2022.
50. Price EL, Sertić Perić M, Romero GQ, Kratina P. Land use alters trophic redundancy and resource flow through stream food webs. J Anim Ecol 2019;88(5):677–89. PubMed PMID: 30712255.
51. Tatenda Dalu, Ryan J. Wasserman, P. William Froneman, Olaf L. F. Weyl. Trophic isotopic carbon variation increases with pond's hydroperiod: Evidence from an Austral ephemeral ecosystem. Sci Rep 2017(7, 7572).
52. Guzman I de, Montoya JM, Elosegi A, Pérez-Calpe AV, Schiller D von, González JM, et al. Food-web energy fluxes, energy transfer efficiency, and diversity respond distinctively to pollution and water diversion in rivers. Freshwater Biology 2024.
53. Hobbie SE, Villóeger S. Interactive effects of plants, decomposers, herbivores, and predators on nutrient cycling. In: Hanley TC, La Pierre KJ, editors. Trophic Ecology: Cambridge University Press; 2015. p. 233–59.
54. García L, Pardo I, Cross WF, Richardson JS. Moderate nutrient enrichment affects algal and detritus pathways differently in a temperate rainforest stream. Aquat Sci 2017;79(4):941–52.
55. Cross WF, Wallace JB, Rosemond AD, Eggert SL. Whole-system nutrient enrichment increases secondary production in a detritus based ecosystem. Ecology 2006;87(6):1556–65.
56. Petchey OL, Gaston KJ. Functional diversity (FD), species richness and community composition. Ecol Lett 2002;5(3):402–11.
57. Rosenfeld JS. Functional redundancy in ecology and conservation. Oikos 2002(98, 1):156–62.
58. Brauns M, Gücker B, Wagner C, Garcia X-F, Walz N, Pusch MT. Human lakeshore development alters the structure and trophic basis of littoral food webs. Journal of Applied Ecology 2011;48(4):916–25.

59. Lorenz S, Trau FN, Ruf LC, Meinikmann K, Fisch K, Stähler M, et al. Pesticide contamination of small standing water bodies in the agricultural landscape of northeast Germany. Sci Total Environ 2025;975:179250. PubMed PMID: 40184998.
60. Liess M, Liebmann L, Vormeier P, Weisner O, Altenburger R, Borchardt D, et al. Pesticides are the dominant stressors for vulnerable insects in lowland streams. Water Res 2021;201:117262. PubMed PMID: 34118650.

Supporting information

Supporting information 1: Active substances (99),with their limits of quantification and detection, measured, as well as the endpoints and test organism used for the toxicity calculations.

Supporting information 2: Biplot for benthic macroinvertebrate before correction for δ15N values from and the influence of organic fertilization. Ellipses incorporate 40% of the data.

Supporting information 3: SIBER density plots for the Layman metrics, computed via Bayesian analysis. Black dots are the modes. Shaded boxes represent the 50 %, 75 % and 95 % confidence intervals, from dark to light red/blue. Grouping is based on the FFG (six groups), the communities are assigned for the water body types (pond, ditch).

Supporting information 4: Representation of the FFGs as coloured dots and ellipses, 40% of the data points are represented by the ellipses.

Supporting information 5: Overview of the sampled FFGs and determination for the ponds and ditches.

Supporting information 6: Layman metrics for ponds and ditches, calculated for the FFGs and the different water bodies as grouping.

Supporting information 7: Biplots for benthic macroinvertebrates in ponds (top) and ditches (bottom). Raw data was corrected with δ15N means of resources.

Supporting information 8: Biplots for benthic macroinvertebrates in ponds (top) and ditches (bottom). Raw data was corrected with δ15N means of resources and δ13C values were corrected for the influence of C4-plant maize.

Figure

Click here to access/download;Figure;Fig1_corrected_OSM.tif

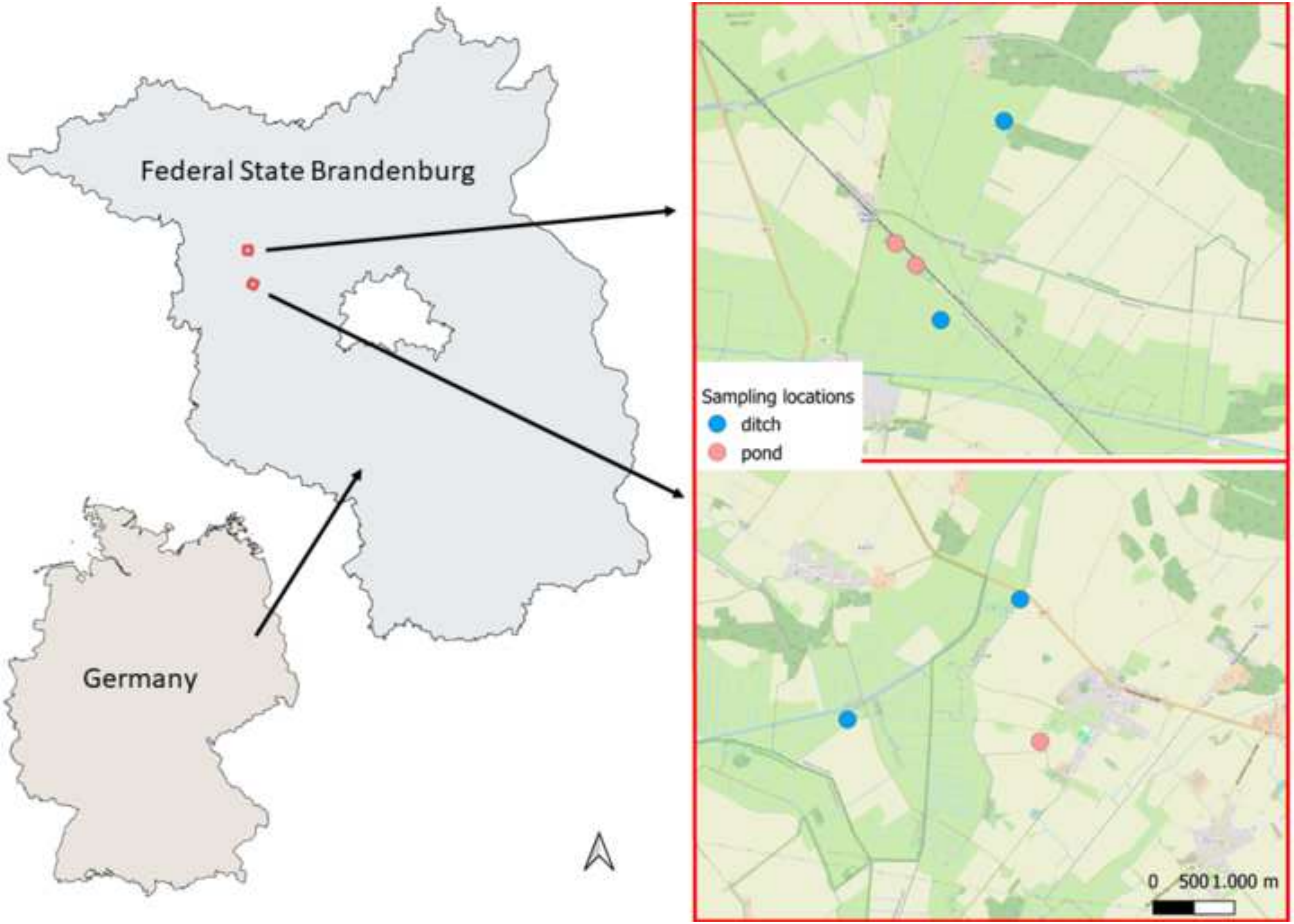

Figure

Click here to access/download;Figure;Fig2.tif

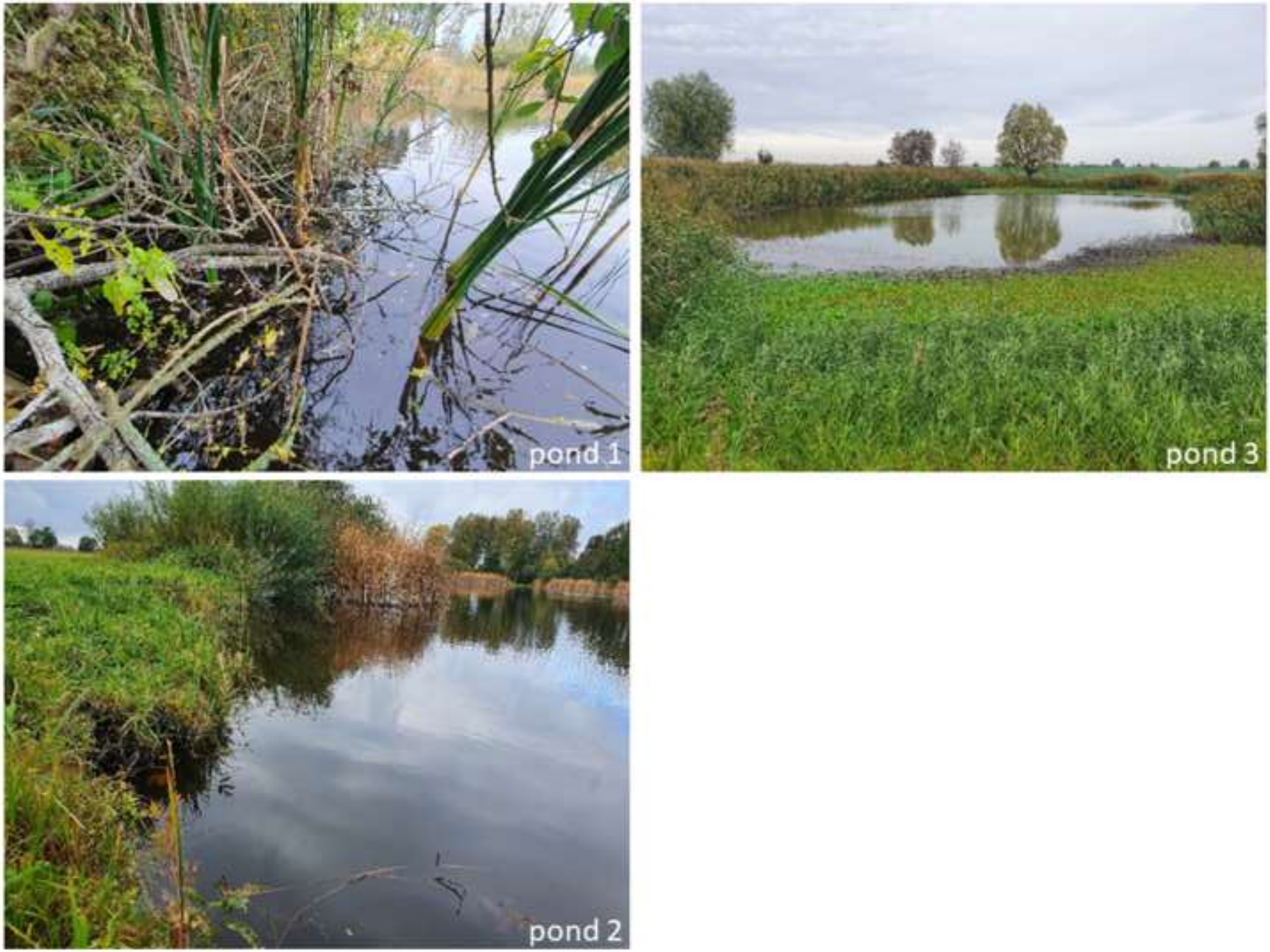

Figure

Click here to access/download;Figure;Fig3.tif

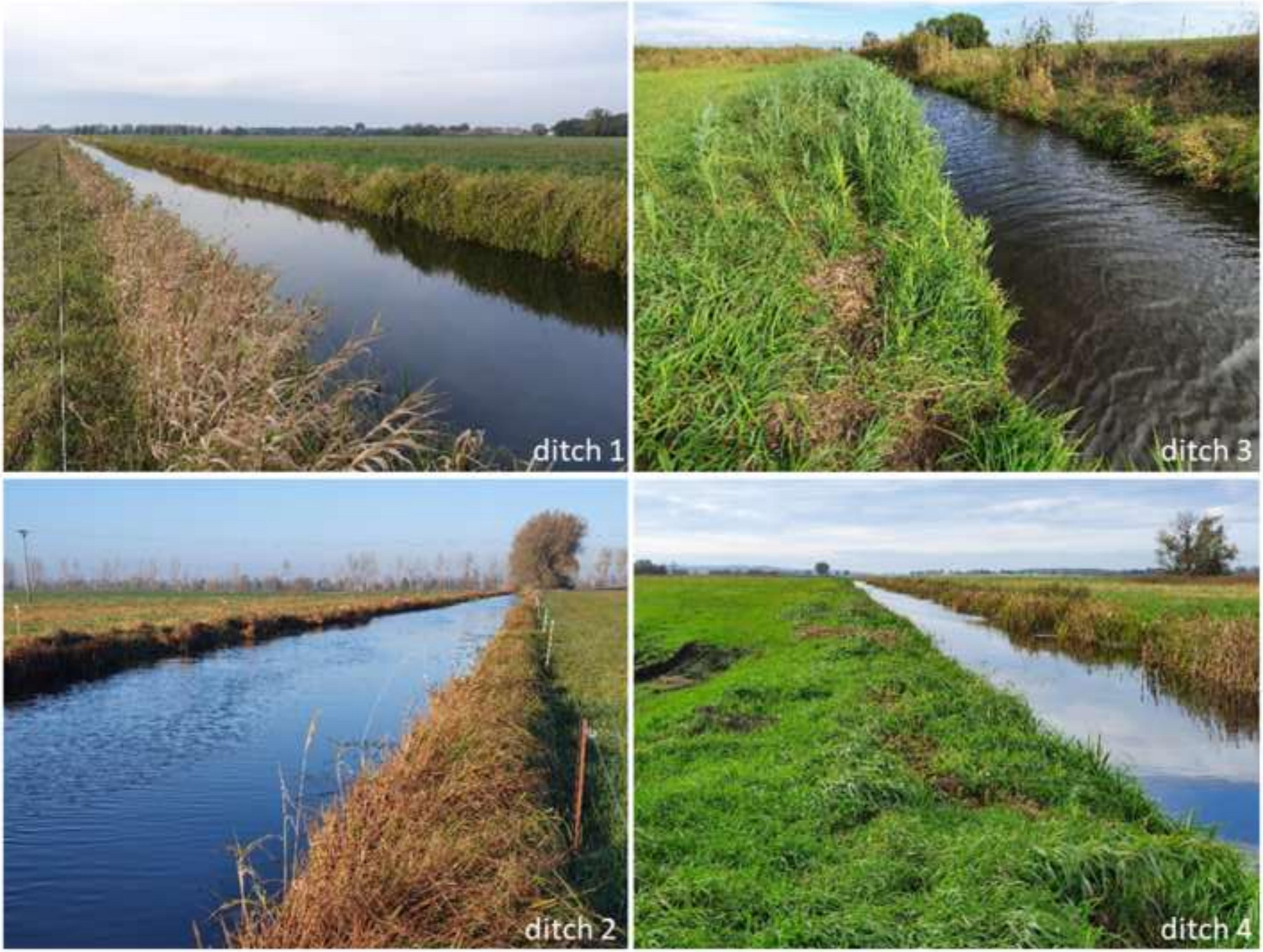

Figure

Click here to access/download;Figure;Fig5_corrected.tif

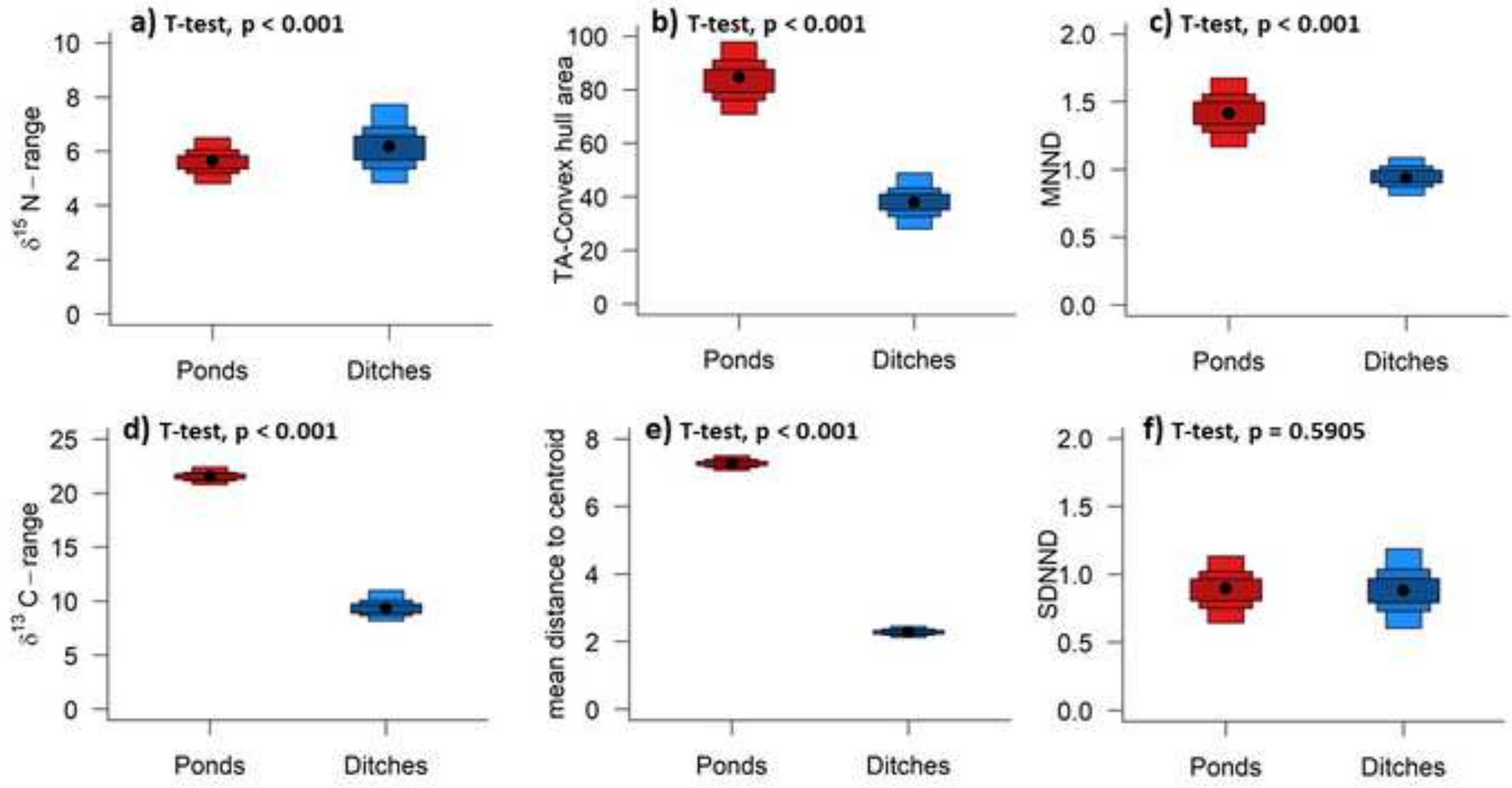

Figure

Click here to access/download;Figure;Fig6_corrected.tif

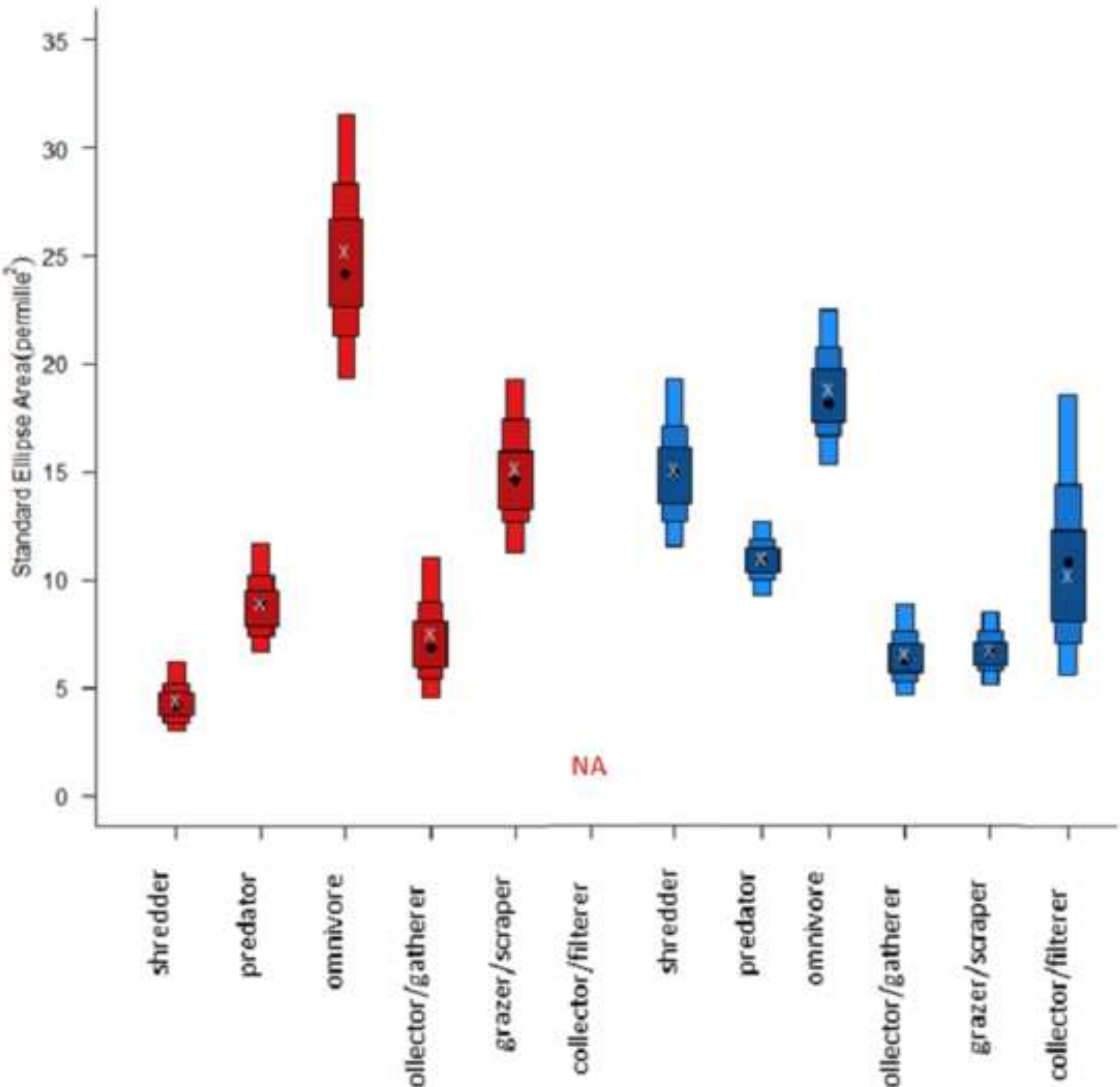

Figure
Click here to access/download;Figure;Fig7.tiff

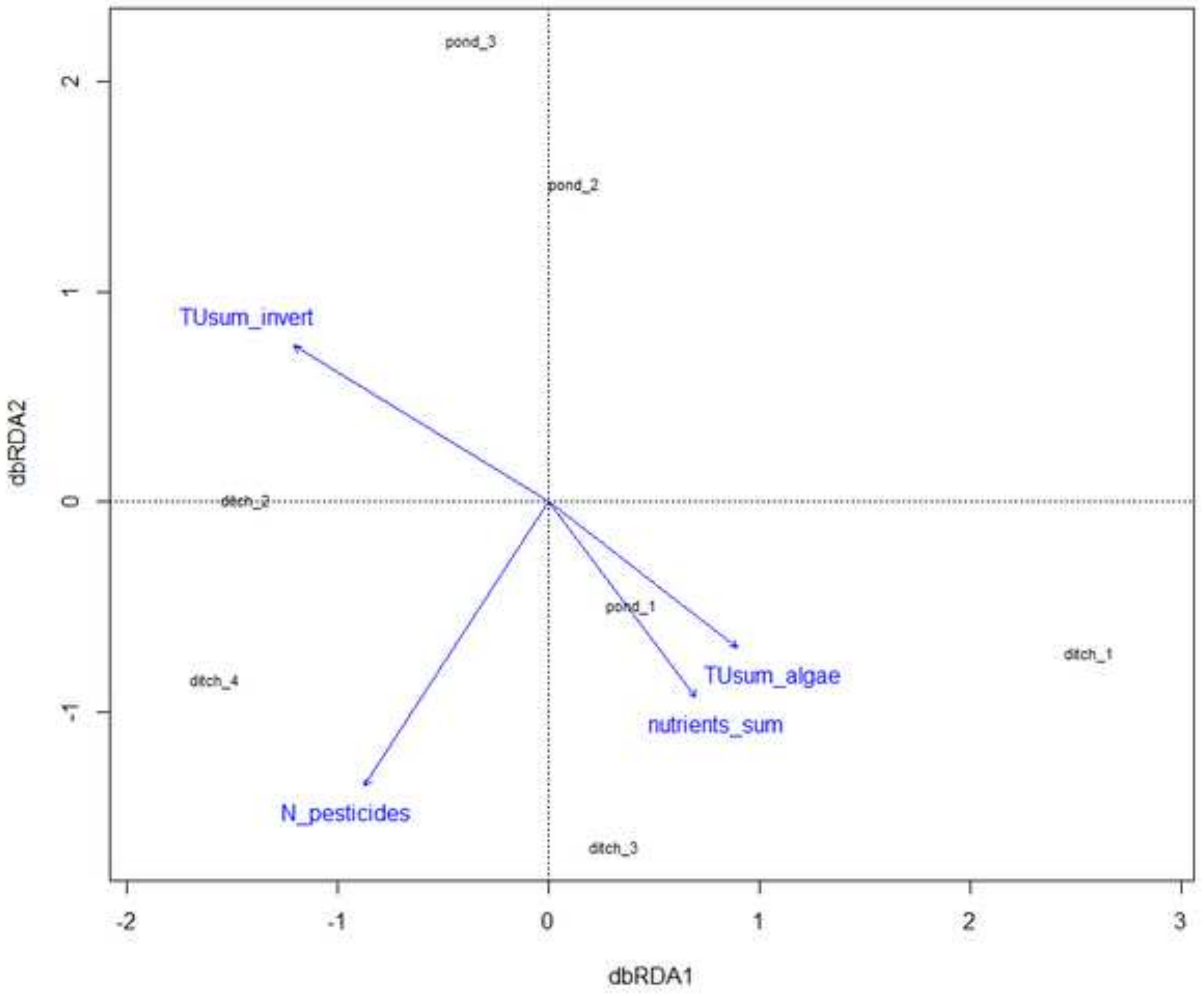

Click here to access/download
Supporting Information
Supporting
Information_Trau_2025_with_track_changes.docx

Click here to access/download
Supporting Information
Supporting Information_Trau_2025_without_track_changes.docx

~~Titel:~~ Assessing agricultural effects on benthic invertebrate communities in ponds and ditches using δ^{15}N and δ^{13}C isotope niches

Formatted: Centered

Authors: Fee Nanett Trau [1*], Kathrin Fisch[1], Stefan Lorenz [1]

[1] ~~Julius Kühn-Institut,~~ Institute for ecological chemistry, plant analysis and stored product protection, Julius Kühn-Institute, Berlin, Germany ~~Königin-Luise-Straße 19, 14195 Berlin, Germany~~

* Corresponding author:

E-Mail: fee-nanett.trau@julius-kuehn.de (FNT)

Abstract

Formatted: Font: Bold

Agriculture is one of the dominant types of land use worldwide. The conversion of land and intensification of agriculture have severe impacts on biodiversity and the associated ecosystem functions in the production areas and in natural or semi-natural habitats embedded in the agricultural landscape. Ponds and ditches, while vulnerable to the negative impacts of agriculture, are valuable habitats for benthic invertebrates due to their heterogeneity and location in a rather homogeneous landscape ~~are also make them valuable habitats for benthic invertebrates~~ and harbor a diverse community as well as endangered or ~~scarce~~ rare taxa. It is challenging to detect community disturbance in these habitats due to indirect or multiple stressors from agriculture, as shown by the failure of classic structural biodiversity metrics, such as overall taxa number, Shannon or evenness index, to perform well in previous studies. Therefore, we used a functional approach to understand the influence of agricultural stressors on isotopic compositions of benthic invertebrate~~s~~ in ditch and pond communities. Benthic invertebrates, from six different functional feeding groups (FFGs), and water samples were collected from three ponds and four ditches in an agricultural landscape in Brandenburg, Germany. We analyzed the carbon and nitrogen stable isotope ratios of the invertebrates and the pesticides and nutrient residues of water samples. ~~The invertebrates were analysed for their stable isotope compositions of carbon and nitrogen, while the water samples were~~

~~analysed for pesticide and nutrient residues. Standard ellipse areas and Layman.B~~Estimates of community metrics were derived using a Bayesian approach for the two water body types and the ~~six functional feeding groups (~~FFGs~~)~~ present. A distance-based Redundancy Analysis was carried out to detect the environmental variables that show the maximum correlation with the derived metrics. Benthic invertebrates in ponds occupied a larger total area and showed greater spacing of individuals in the isotope space than communities in ditches, indicating that more resources and/or habitats were available and utilized in ponds. No clear pattern emerged when comparing the FFGs in the two water body types, but some unexpected differences were found, for example in FFG *predator*, which was more abundant and diverse in ditches. ~~Overall, the communities seem to be influenced on a case-by-case basis by various factors, either at the production or the consumption level. In the end, o~~Our study found that benthic invertebrate communities respond in complex ways to stressors in small unstable systems, which are also subject to strong fluctuations in biotic and abiotic parameters. The impacts of individual structures within each water body are more pronounced than influences based on the type of water body. However, larger datasets may yield in more pronounced patterns on effects of agriculture on stable isotope composition in ponds and ditches.

Keywords: agricultural landscape, benthic invertebrates, food web, isotope niche, small water bodies

Formatted: Font: Not Italic

1. Introduction

Agriculture covers about 38 % of the world's terrestrial area, which makes it the predominant land use type (1). The conversion of forests, wetlands, and natural grasslands to agricultural land has had severe impacts on the provision of habitats, biodiversity, and associated ecosystem functions (1,2). Likewise, the increasing specialization of farmers, the expansion and intensification of agriculture (e.g. by fertilization and pesticide application) further increased homogenization of the landscape, resulting in accelerated habitat fragmentation and biodiversity losses (3). Those developments continue to exert ongoing pressure on the remaining semi-natural (e.g. hedges, ponds, field margins, ditches) or natural habitats within the agricultural landscape and on the surrounding aquatic and terrestrial environments (4).

Examples for those ~~of~~ remaining semi-natural habitats and non-productive areas in the context of agriculture are water bodies such as ponds and ditches, which provide valuable habitats for aquatic and semi-aquatic species. ~~Furthermore, ponds~~Ponds and ditches are biodiversity hotspots and form aquatic corridors in a landscape dominated by agriculture in need of conservation action (5,6). Unfortunately, these small water ~~bodies have not received as much attention in ecological research as they deserve~~ (5,7) ~~and~~ are usually not included in monitoring programs (5,8), but ~~.~~ t~~T~~he importance of particularly ponds, but also ditches, for benthic macroinvertebrate biodiversity and ecosystem services is increasingly gaining recognition in research activities (5,9–14). Ponds are known to harbour high species numbers (9). However, also d~~D~~itches, which are usually in close proximity to agricultural ~~practices~~activities, have the potential to host diverse communities as well as ~~scarce~~ rare or endangered aquatic species (5) and show a high richness ~~of~~ for the groups Coleoptera, Gastropoda, Trichoptera and Heteroptera ~~species~~(5). ~~Furthermore, ponds and ditches are biodiversity hotspots and form aquatic corridors in a landscape dominated by agriculture in need of conservation action~~ (5,6)~~.~~ ~~Unfortunately, these small water bodies have not received as much attention in ecological research as they deserve~~ (5,7) ~~and are usually not included in monitoring programs~~ (5,8)~~.~~

Formatted: Font: Bold

Field Code Changed (×15)

To conserve and promote these aquatic habitats and their biodiversity, knowledge on the status and indicators for measuring biodiversity are indispensable. However, the assessment of water or habitat quality by benthic invertebrate biodiversity is challenging, and results from a previous~~s~~ study~~ies~~ on ponds have shown that conventional structural biodiversity metrics (e.g. taxonomic diversity, Shannon index, and the EPT index, to a certain degree) do not perform well in detecting stressed communities in agricultural areas due to long-term and ubiquitous persistence of stressors and short stressor gradients (12). Additionally species occurrences may be subject to some degree of stochasticity (15) and functional responses can reflect the ecological conditions and habitat change better than taxonomic approaches (16,17). Therefore, functional indicators and approaches could be promising tools to detect community disturbance and to examine possible direct as well as indirect effects of stressors on benthic invertebrate communities and their functions (12,18,19). Common traits used to assess the functional diversity of benthic invertebrates are feeding mode, locomotion, maximum body size, and respiration mode (20). We selected FFG to address the effects of agriculture as further stressors such as channelization and modification of physical structure do not vary in their intensity in the selected water bodies. As benthic invertebrates play an important role in the cycling of nutrients and decomposition of organic matter, the analysis of trophic relationships and functional feeding groups (FFGs) are central to understand~~ing~~ ecosystem processes (21,22) and to visualiz~~ing~~e effects of agricultural activity on communities (18,23–25). The niche concept is ~~F~~fundamental to understand how communities are organized and how ecological interactions shape their strategies and ecological shifts ~~is the niche concept~~ (26). The niche of a species describes the range of conditions necessary for the species to exist and reproduce, including its abiotic and biotic interactions within the environment (27). Niches can overlap when species are co-occurring, which leads to interspecific competition. Higher species richness can lead to greater niche overlaps under environmental disturbance, as for example found in carabid communities (28).

~~Stable isotope studies are a powerful tool for understanding trophic relationships and community use of resources and habitats.~~ The basic principle of “you are what you eat (plus a few ‰)” (29) ~~makes~~

~~it~~has led ~~possible~~ to the use of stable isotopes to analyse isotopic niches, food web structures, and ~~interactions between~~ species interactions in ecosystems (30). Two elements commonly used for this purpose are nitrogen and carbon. The ratio of ^{15}N to ^{14}N ~~enriches stepwise along the~~progressively increases at each trophic level~~s~~ whereas the ratio of ^{13}C to ^{12}C remains almost unchanged when passing through the food web, but discriminates food sources due to different photosynthetic pathways (29). Accordingly, $\delta^{15}N$ values are used to determine the trophic position of an organism ~~and~~ while $\delta^{13}C$ values provide information on the sources utilized by the organism.

Based on ~~this~~ the information on carbon and nitrogen stable isotope ratios, the isotopic niche of a species or taxa group (e.g. Coleoptera, Odonata etc.) can be quantified and ~~community~~ metrics that for example provide insights on the isotopic niche width or proxies for resource~~s~~ use can be derived, which are statistically comparable over a broad spatial scale and between communities composed of different species (19,31). The isotopic niche can thus serve as a proxy for the realized ecological niche in relation to the use of resources and habitat (32). The realised niches are linked to land use and agricultural stress factors, as these change the availability of resources and habitat quality. In this study, the ~~word~~term niche will be used in the sense of the isotopic niche~~,~~ as described above. ~~The isotopic niche can serve as a proxy for the realized ecological niche in relation to the use of resources and habitat~~ (32)~~.~~

~~The realised niches are linked to land use and agricultural stress factors, as these change the availability of resources and habitat quality.~~

~~The niche concept in community ecology is fundamental to understanding how communities are organized and how ecological interactions shape their strategies and ecological shifts~~ (26)~~. The niche of a species describes the range of conditions necessary for the species to exist and reproduce, including its abiotic and biotic interactions with the environment~~ (27)~~. Niches can overlap when species are co-occurring, which leads to interspecific competition. Higher species richness can lead to greater niche overlaps, as for example in carabid communities~~ (28)~~. For a detailed review of niche concepts~~

Field Code Changed

Field Code Changed

~~and developments during the 20th century see~~ (26) ~~and for examples on the further development of the `old` niche concepts (in the early 2000s) see~~ (32) ~~and~~ (27)~~.~~

~~In this study we will use niche in the sense of the isotopic niche, which can serve as a proxy for the realized ecological niche in relation to the use of resources and habitat~~ (32)~~.~~ ~~Therefore, w~~WWe ~~will~~ compared niche widths of ~~groups~~ FFGs and the pond and ditch communities based on a geometric approach originally developed by (25) to examine the isotopic niches of benthic ~~macro~~invertebrates in ponds and ditches.~~, as well as~~ The aim is to assess the influence of agricultural stressors (~~e.g.~~ toxicity of water samples due to pesticide residues and eutrophication due to nutrient inputs) on the isotopic niche metrics.

We assume that ponds and ditches are affected differently by agricultural stressors, affecting habitat availability and quality and thus influencing benthic invertebrate food webs and niche size (33,34). Ditches are expected to be more strongly influenced by agricultural stressors than ponds due to their characteristics, which are a rather homogeneous habitat in and around the water body, a directly farmed environment with the location in the field (grassland) without a riparian buffer. Ditches, for example, are probably more contaminated, but as they are part of a flow system, pollutants can also be transported downstream. Ponds, on the other hand, are better protected from pesticide inputs, but can act as a source and sink for pesticide residues in the sediment (35). The water body type's ponds and ditches are used as gradients of agricultural effects, including nutrient enrichment, pesticides, and the proportion of surrounding crops to test our hypotheses. We asked the following questions, using ponds and ditches~~ as an example.~~: Can the isotopic niches depict the effects of agriculture on benthic invertebrate communities in the selected water bodies in the study region? Are the observed patterns of isotopic niches of FFGs comparable in ponds and ditches for the FFGs?

~~In pursuit of~~To answers ~~to~~ these questions, we hypothesize that agricultural activity and low habitat variability in ditches have led to simplified trophic structures, which will be shown by wider isotopic niches, reduced niche differentiation, and ~~lower~~ larger niche overlap due to reduced ~~specialisation~~ differentiation of the FFGs and more generalists present.

Field Code Changed

Field Code Changed

Field Code Changed

2. Methods and data

2.1 Study area

Sampling was carried out in Brandenburg in the “Havelländisches Luch” region (Fig 1). The wet lowlands in the region were drained and an intensively maintained ditch system was created for industrial agricultural production d~~D~~uring ~~the time of the German Democratic Republic until~~ the 1980s~~,~~ ~~the wet lowlands of this area were drained and an intensively maintained ditch system was created for industrial agricultural production~~ (36). Traditional~~,~~ extensively managed and diverse~~-~~ grasslands were converted to seeded grasslands, often with only one type of grass, and in some cases also plowed into arable land (36). Additionally, the landscape is characterized by a large number of small standing water bodies (kettle holes, consistently named “ponds” within the context of this paper), which were formed during the last glacial period. Ponds are defined as water bodies with a maximum extent of one hectare and cover about 5 % of the arable land area over a total area of 38.000 km^2 in northeast Germany (37).

Fig 1: Map of sampling locations, ditches in blue and ponds in red. ~~-~~Map data: ~~Google satellite~~Open street map , QGIS Version 3.34.3

Samples were taken in ~~four ditches~~three ponds ~~-~~(Fig 2) and ~~three ponds~~four ditches (Fig 3) in October and November 2020. Pond 1 is entirely encompassed with trees, shrubs, and reed stands (*Phragmites* sp.). Ponds 2 and 3 are characterized by a mix of trees, shrubs, grasses (Poaceae), and reed~~s~~ (*Phragmites* sp.) in the riparian zone. Ditch 1 was dominated by reed~~s~~ (*Phragmites* sp.), while ditches 2 to 3~~.~~~~Three of the four ditches~~ were dominated by grasses (Poaceae) in the riparian zone. ~~Ditch 1 was dominated by reeds. Ponds 2 and 3 are characterized by a mix of trees, shrubs, grasses, and reeds in the riparian zone, while pond 1 is entirely encompassed with trees, shrubs, and reeds.~~ See also Table 1 for descriptions of the water bodies and their surroundings.

*Table 1: Description of the sampling sites. **Habitats were described for the water bodies~~,~~ when present in the wadeable area of the shore length ~~more than~~with a share < 5 %~~-~~. The riparian zone is defined as the area from the edge of the field ~~including the edge strip~~ to the waterline~~, on this area the growth of grasses etc.~~ where vegetation was estimated as a percentage.** CPOM = coarse particulate organic matter; FPOM = fine particulate organic matter**. Dominant types of riparian vegetation are written in bold.***

Formatted: Font: Bold

Field Code Changed

Formatted: Normal, Justified, Space After: 6 pt, Line spacing: Double, Don't keep with next

Formatted: Font: Italic

Formatted: Font: Italic

Formatted: Normal, Don't keep with next

Formatted: Font: 9 pt, Italic, Font color: Text 2

			Riparian zone [in %]				
**Site**	**Habitats**	**Agricultural cultivation**	**Grasses**	**Herbaceous perennials**	**Trees**	**Shrubs**	**Reeds**
**Pond 1**	Reeds, CPOM, macrophytes	Grassland	0	0	25	25	**50**
**Pond 2**	Reeds, CPOM, macrophytes	Grassland	30	0	5	5	**60**
**Pond 3**	Reeds, CPOM, FPOM	Maize, cereal	0	20	10	10	**60**
**Ditch 1**	Reeds, FPOM	Grassland	20	0	0	0	**80**
**Ditch 2**	CPOM, macrophytes	Grassland	**100**	0	0	0	0
**Ditch 3**	Reeds, CPOM, FPOM	Grassland	**80**	0	0	0	20
**Ditch 4**	Reeds, FPOM, macrophytes	Grassland	**100**	0	0	0	0

Formatted: Font: Bold (×7)

Fig 2: Sampled ~~ditches~~ponds, pictures: Fee Nanett Trau, JKI

Fig 3: Sampled ditches~~ponds~~, pictures: Fee Nanett Trau, JKI

2.2 Sampling and sample processing ~~–~~– invertebrates

A species protection exemption permit in accordance with Section 45 (7) No. 3 of the Federal Nature Conservation Act (BNatSchG) for the capture and killing of invertebrates with non-specific traps for agro ecological studies in the Havelland district was issued by the Brandenburg State Office for the Environment (LfU).

Benthic invertebrates were sampled using hand netting (mesh size 500 µm) from the ~~different FFGs present~~ habitats (FPOM, CPOM, submerse macrophytes, reed) present in the selected water bodies. ~~were sampled via hand netting (mesh size 500 µm) from present habitats, such as FPOM, CPOM, submerse macrophytes, and reeds~~Individuals from ~~-~~all FFGs present were sampled and o~~O~~rganisms were sorted on-site by ~~taxonomy~~ morphology (to the level of order or family)~~ and feeding groups~~. Sampling was carried out until 10 specimen of the present orders/families were collected as recommended by (31), whenever possible. All animals were kept alive for about 24 hours so that they

Formatted: Font: Bold

Formatted: Font: Bold

Formatted: Normal, Space After: 6 pt, Line spacing: Double

Formatted: Font: 11 pt

Field Code Changed

can empty their gastrointestinal tract before analysis and then killed as quickly and humanely ~~dispatched~~ as possible with boiling water to avoid altering the isotopic signals through the use of ethanol. Afterwards the samples were frozen before further processing. In the laboratory, the benthic macroinvertebrates were further taxonomically determined to family (where the order was assigned on site) or genus level when possible and sorted into the FFGs based on the freshwater ecology database (38). Gastropods, Bivalves, and Trichoptera were removed from their shell or caddis and parasites were removed from afflicted Heteropterans before further sample processing to only analyse the isotopic signal of the body tissue of selected animals. In the next step, t~~T~~he samples were dried (48 hours at 60 °C, Memmert UFE 600). ~~The individual organisms were stored in Eppendorf tubes (1.5 ml).~~ If the animal weight was >2 mg (Sartorius LA 230 P), one~~the~~ individual organism was ground into a powder. For lower sample weights, several individuals from one family were pooled into one sample to reach the optimum sample weight of 2~~1~~ mg and then ground. Material was homogenized using a mixer mill (Retsch MM400) and stored in Eppendorf tubes (1.5 ml). For each family present, all samples were analysed with a maximum of 10 to 15 samples.

The encapsulation as well as the isotope analysis was carried out by the stable isotope laboratory at Cornell University (COIL), Department of Ecology and Evolutionary Biology, USA. Stable isotope analysis of ^{15}N and ^{13}C was performed using a Thermo Delta V isotope mass spectrometer (IRMS) interfaced to a NC2500 elemental analyser. Raw data, sample weights and q~~Q~~uality control values for presented data can be obtained here (insert reference to IsoBank repository, status in submission). Isotope corrections were performed using linear regression of all δ values using two additional in-house standards (39). Results were reported as δ values as ratios of $^{13}C{:}^{12}C$ relative to Vienna PeeDee Belemnite and $^{15}N{:}^{14}N$ relative to atmospheric air (Eq. 1). Measurements were carried out from April 2021 to July 2022 in four batches.

$$\delta X = \left[\left(R_{sample}/R_{standard}\right) - 1\right] * 10^{3} \quad (1)$$

Where X is ^{13}C or ^{15}N, R is the corresponding ratio $^{13}C/^{12}C$ or $^{15}N/^{14}N$. Increased δ values show an increase of the heavy isotope in the sample~~. Taken from~~ (40).

Field Code Changed

Formatted: Normal, Justified, Line spacing: Double

2.3 Sampling and sample processing - water

Environmental ~~Physical and~~ chemical parameters were measured to identify potential variables that could function to ~~impact~~ explain the community's food web structures or isotopic niches of groups. Water samples were taken via grab sampling. A mixed water sample of approximately 5 L was taken from four to six locations around the shoreline of the ponds or along a stretch of 50 m from the ditches.

Environmental ~~Physicochemical~~ water parameters [oxygen content, electrical conductivity (EC), pH, and temperature] were measured in-situ using a portable meter (WTW Multi 3430 IDS with OxiCal-SL FDO 925, TetraCon 925 and SenTix 940 probes, Germany). Samples for pesticide analysis were partitioned into two 500 mL glass bottles, with one bottle holding 50 mL of dichloromethane for pyrethroid analysis. The water samples were analysed using multi-methods validated for analysis of 99 active substances (S1 Table).

Except for the pyrethroids, the selected active substances in the water samples were quantified by liquid chromatography mass spectrometry (LC-ESI-MS/MS) using the internal standard method after enrichment and clean-up via solid phase extraction (Chromabond HR-P, Macherey-Nagel, Germany, 50-100 µm, 3 mL, 200 mg). For LC-MS/MS analysis a 1290 Infinity II LC system (Agilent, USA) coupled to a QTRAP 6500+ mass spectrometer (SCIEX, USA) was used.

After concentration by liquid–liquid extraction of the sample, pyrethroids were analysed by gas chromatography-mass spectrometry (GC-MS) using a Trace GC Ultra coupled to a TSQ Quantum GC XLS- mass spectrometer (Thermo Electron Corporation, USA). All pesticide analysis were done accordingly to (12).

Samples for nutrient analysis were filtered through a 0.45 µm cellulose acetate filter before analysis. The concentrations of NH_4^+ + and PO_4^{3-} were measured photometrically using a spectrophotometer (DR/2000 Hach) according to DIN 38 406–5 and EN ISO 6878:2004, respectively. NO_3^- and NO_2^-

Formatted: Justified, Space After: 8 pt

Formatted: Font: Bold

Field Code Changed

concentrations were analysed by high-performance liquid chromatography (HPLC, Agilent 1100 Series HPLC System DAD, USA).

2.3.1 Toxicity calculations

To calculate the toxicity of the analysed water samples for invertebrates~~, macrophytes~~ and algae based on measured pesticide residue concentrations, ~~in the water samples for invertebrates, macrophytes and algae t~~~~T~~he toxic pressure for each sample is calculated as the sum of toxic units; TU_{sum} (Eq. 2). Toxic units were calculated for the pesticide concentrations based on half-maximal effective concentration (EC50) or 50% lethal concentration (LC50) values for invertebrates~~, macrophytes, ~~and algae. Analyzed pesticide concentrations were divided by the concentration for the most sensitive test organism and based on 48 h EC50 values for *Daphnia magna* or 96 h LC50 values for *Chironomus* sp. for the invertebrate toxicity. Toxicity calculations for~~ macrophytes are based on 7-day EC50 values for *Lemna gibba* and *Lemna minor* and for~~ algae are based on 72 h EC50 values for mostly, *Raphidocelis subcapitata* (syn: *Pseudokirchneriella subcapitata*) and *Scenedesmus subspicatus*. Data on the EC50 and LC50 values was obtained from the Pesticides Properties Database (41) or Ecotox database (42). See also S1 table for details on substances measured and the test organisms.

$$TU_{sum} = \log \sum_{i=1}^{n} \left(\frac{C_i}{EC_{50_i}/LC_{50_i}} \right) \quad (2)$$

C_i is the concentration of pesticide *i* in µg/L. Concentrations below the LOQ but above 0 were replaced with half of the LOQ.

2.4 Description of the dataset

The dataset contained $\delta^{13}C$ and $\delta^{15}N$ data ~~from 13 orders ~~covering six FFGs (Table 2) from 13 orders (see S5 for details). FFGs were assigned to the individuals according to the family determination, if available, otherwise by order (33,38,43,44). ~~The dataset contained values for 22 environmental parameters measured (Table 3).~~ Classified *as collector/filterer* are Bivalvia and the family Culicidae ~~Gen. sp.~~, which filter suspended FPOM from the water column (38). The families of Ephemeroptera and some ~~D~~dipterans belong to the group *collector/gatherers*, which feed on sedimented FPOM. *Grazers/scrapers* ~~graze on surfaces where they~~ feed on algae tissue or biofilm growing on surfaces,

Formatted: Font: Bold

Formatted: Font: Not Italic

Field Code Changed

Formatted: Font: Bold

Field Code Changed

Formatted: Font: Italic

Field Code Changed

Formatted: Font: Italic

Formatted: Font: Italic

also they feed on algae or plant tissue (38). In the present dataset ~~*those*~~*grazers/scrapers* are mostly ~~G~~gastropods ~~and families belonging to the group are for example~~e.g. of the families Lymnaeidae or Planorbidae. Within the group *omnivore*, families are gathered that could not be assigned to one distinct group. In the present dataset Chironomidae~~,~~, Corixidae~~,~~, Haliplidae and Stratiomyidae were assigned ~~to the group~~as *omnivore* ~~*indifferent*~~. *Predators* hunt on prey and examples are Dytiscidae,~~,~~ Ceratopogonidae or Coenagrionidae. Typical *shredders* are crustaceans like Gammaridae~~,~~ or Asselidae ~~,~~, which feed on plant tissue, fallen leaves or CPOM (38). A detailed list of the families belonging to the FFGs can be found in the table supporting information 5.

Table 2: ***~~Sample sizes~~Number of samples available and used for analysis for each water body and the FFGs.***

FFG	Pond 1	Pond 2	Pond 3	Ditch 1	Ditch 2	Ditch 3	Ditch 4	Sum FFG pond/ditch
**Collector/ filterer**	0	0	0	3	0	0	9	**0/12**
**Collector/ gatherer**	13	5	3	11	10	10	10	**21/41**
**Grazer/ scraper**	37	8	11	16	14	18	16	**56/64**
**~~Indifferent~~ Omnivore**	15	31	22	28	23	30	25	**68/106**
**Predator**	19	19	14	38	45	26	60	**52/169**
**Shredder**	24	7	0	18	21	10	12	**31/61**
**Sum type**		**Ponds = 228**			**Ditches = 453**			

Formatted: Font: Italic

Field Code Changed

Formatted: Font: Italic

Formatted: Font: Italic

Formatted: Font: Italic

Field Code Changed

~~(Table 3).~~***~~Table 3: Overview of the measured water quality and environmental parameters for the seven water bodies. EC = electric conductivity, CPOM = coarse particulate organic matter, FPOM = fine particulate organic matter~~***

~~Sample~~	~~C °~~	~~EC [µS/cm]~~	~~pH~~	~~O_2 [mg/L]~~	~~NH_4^+ [mg/L]~~	~~NO_2^- [mg/L]~~	~~NO_3^- [mg/L]~~	~~PO_4^{3-} [mg/L]~~	~~NH_4^+ NO_2^- +NO_3^- [mg/L]~~	~~TUsum algae~~	~~TUsum plants~~	~~TUsum inverte-brates~~	~~Algae total [µg/l]~~	~~Cyano-bacteria [µg/l]~~	~~Green algae [µg/l]~~	~~Diatoms [µg/l]~~	~~Crypto-phyceae [µg/l]~~	~~Photo-synthetic activity [%]~~	~~Reeds [%]~~	~~Habitat~~	~~Number of habitats~~	~~Pesticide detections~~
~~Pond 1~~	~~7.4~~	~~1570~~	~~6.2~~	~~5.2~~	~~0.01~~	~~0~~	~~0.2~~	~~0.25~~	~~0.21~~	~~-2.686~~	~~-4.602~~	~~-5.221~~	~~28.85~~	~~0~~	~~17.07~~	~~4.39~~	~~7.39~~	~~49.34~~	~~50~~	~~Reeds, CPOM, macrophytes~~	~~3~~	~~3~~
~~Pond 2~~	~~10.5~~	~~1112~~	~~6.5~~	~~10.6~~	~~0.09~~	~~0~~	~~0.14~~	~~0.05~~	~~0.23~~	~~-4.057~~	~~-5.205~~	~~-5.074~~	~~112.3~~	~~0~~	~~63.68~~	~~48.31~~	~~0.35~~	~~52.03~~	[illegible]	[illegible] ~~CPOM~~	[illegible]	[illegible]
~~Pond 3~~	~~11.2~~	~~881~~	~~7~~	~~13.8~~	~~0.08~~	~~0~~	~~0.23~~	~~0.11~~	~~0.31~~	~~-2.650~~	~~-4.216~~	~~-4.632~~	~~468.8~~	~~241.55~~	~~0~~	~~0~~	~~227.27~~	~~0.56~~	~~60~~	~~Reeds, CPOM, FPOM~~	~~3~~	~~4~~
~~Ditch 1~~	~~9.7~~	~~588~~	~~7~~	~~11.5~~	~~0.86~~	~~0~~	~~1.1~~	~~0.44~~	~~1.96~~	~~-2.685~~	~~-4.849~~	~~-5.428~~	~~41.25~~	~~0.74~~	~~7.35~~	~~31.73~~	~~1.43~~	~~62.39~~	[illegible]	[illegible] ~~FPOM~~	[illegible]	[illegible]
~~Ditch 2~~	~~8.3~~	~~815~~	~~7.6~~	~~6.1~~	~~0.08~~	~~0~~	~~0.72~~	~~0.06~~	~~0.8~~	~~-3.253~~	~~-3.512~~	~~-5.116~~	~~40.98~~	~~0.79~~	~~6.9~~	~~29.99~~	~~3.3~~	~~60.25~~	~~0~~	~~CPOM~~	~~1~~	~~6~~
~~Ditch 3~~	~~11~~	~~928~~	~~7~~	~~8.8~~	~~0.08~~	~~0~~	~~0.36~~	~~0.06~~	~~0.44~~	~~-2.614~~	~~-3.930~~	~~-4.817~~	~~78.27~~	~~2.05~~	~~25.75~~	~~42.66~~	~~7.81~~	~~58.63~~	[illegible]	[illegible] ~~CPOM, FPOM~~	[illegible]	[illegible]
~~Ditch 4~~	~~12.7~~	~~900~~	~~8~~	~~8.8~~	~~0.08~~	~~0~~	~~1.32~~	~~0.03~~	~~1.4~~	~~-3.456~~	~~-3.641~~	~~-4.925~~	~~5.7~~	~~0~~	~~1.22~~	~~0.88~~	~~3.6~~	~~52.15~~	~~0~~	~~Reeds, FPOM~~	~~2~~	~~7~~

Formatted Table

Formatted Table

Formatted Table

2.5 Statistical methods

All data processing and statistical analyses were performed with R Studio version 4.3.1 (45) and JAGS version 4.3.1.

2.5.1 δ^{15}N correction

The influence of organic fertilizer was ~~visible~~ observed in ditches by examining the δ^{15}N values of benthic macroinvertebrates (consumers). The δ^{15}N values for ditches are higher than in ponds due to higher δ^{15}N values in resources present in these water bodies (S2 Fig.). To compare the FFGs in the two water body types this was corrected as follows: For both water body types the mean δ^{15}N value for the consumers was calculated. The means were subtracted from each other ~~-~~and the~~its~~ difference was subtracted from the δ^{15}N values of the consumers in ditches only.

2.5.2 δ^{13}C correction

The δ^{13}C values for one pond are higher due to the influence of maize cultivation (C4-plant) on the consumers (Fig. 4). For ponds 1 and 2 (no maize influence) and pond 3 (maize influence) the mean values for δ^{13}C consumers were calculated. The means were subtracted from each other and the~~its~~ difference was subtracted from δ^{13}C values from consumers in pond 3 only.

~~The dataset with corrected δ^{15}N and δ^{13}C values was used for the comparison of FFGs using the corrected Standard Ellipse Area (SEA.c). The calculation of the Bayesian Layman metrics (Layman.B) and the distance-based Redundancy Analysis (dbRDA) were performed for the data set with corrected δ^{15}N values. Both versions were calculated to visualize the benthic invertebrate data in a biplot and to compare niche overlaps for the FFGs.~~

2.5.3 SIBER (Stable Isotope Bayesian Ellipses in R)

The R package SIBER (46) was used for the analysis of isotopic niche width of the FFGs and the comparison of food web structures between different water body types. The analysis follows a Bayesian approach to overcome uncertainties when calculating and comparing metrics (46). The estimation by Bayesian inference incorporates error from the sampling process and allows robust comparisons between groups with different and small sample sizes (31).

Formatted: Font: Bold

Formatted: Font: Bold

Formatted: Font: Bold

Formatted: Font: Bold

Field Code Changed

First the raw data was plotted for the six FFGs (*collector/filterer, collector/gatherer, grazer/scraper, omnivore~~indifferent~~, shredder,* and *predator*) and the respective water body types (S4 Fig.). Then the Layman metrics and the Bayesian inference-derived Layman.B metrics were calculated for the combination of water body and FFGs as group (37 groups) and the water body types pond and ditch as community as well as for the FFG as group (six groups) and the water body types pond and ditch as community. To compare the groups the Welsch's t-test was used (confidence interval 95 %). For the dbRDA (see below), the Layman metrics for the seven water bodies as community and FFG as group (six) were calculated.

The described metrics are based on (25) and summarize information on the distribution of data points in the isotope space. The six metrics are the $\delta^{13}C$ range, the $\delta^{15}N$ range, the total area of the convex hull (TA), the mean distance to centroid (CD), the mean nearest neighbor distance (MNND) and the standard deviation of the nearest neighbor distance (SDNND) (31). The TA describes the niche width, CD ~~is giving~~provides information on niche width and species spacing, the MNND is a description of density and clustering of species, and the SDNND gives information on the evenness of the species density (31).

Field Code Changed

Field Code Changed

Field Code Changed

Additionally, the Bayesian standard ellipse areas (SEA.B), corrected standard ellipse areas (SEAc), and convex hull area (TA) based on Bayesian interference were calculated for the FFGs in ponds and ditches. The Bayesian estimates provide robust measures of probability and can reflect model uncertainty by averaging many possible models into one single model (47). The SEA.c is based on SEA.B, which describes isotopic niche width, and corrected for small sample sizes for unbiased analysis (31). A larger SEA corresponds to a more diverse isotopic signal and thus to wider niches. The dataset with corrected $\delta^{15}N$ and $\delta^{13}C$ values was used for the comparison of FFGs using the corrected Standard Ellipse Area (SEA.c). ~~The $\delta^{15}N$ and the $\delta^{13}C$ values were corrected for the influence of organic fertilization and the influence of the C4-plant maize in order to focus on the influence of the FFGs on the ellipse area.~~ The data was plotted using the function *siberDensityPlot* (48).

Field Code Changed

The probability that the posterior distribution is smaller or larger for a group can be estimated by calculation of the SEA.B niche overlap. SEA.B overlaps between ponds and ditches were calculated for the FFGs based on the maximum likelihood estimates of the means and the covariance matrices of each group (function: *maxLikOverlap* calculated for the proportion of both overlaps, p-interval of 0.95). The calculation of the Bayesian Layman metrics (Layman.B) ~~and the distance-based Redundancy Analysis (dbRDA) were~~was performed for the data set with corrected $\delta^{15}N$ values.

Bayesian models were run with JAGS and model parameters were defined according (31) to fit the ellipses.

Field Code Changed

2.5.4 Multivariate statistics

Formatted: Font: Bold

Environmental variables were standardized using the *scale* function of the *base* package (49), whereby the mean value was set to zero and scaled to unit variance. Variable selection for the dbRDA was carried out by the *bioenv* function from the *vegan* package (50). This function detects the best environmental variable subset in a way such that the environmental variables show the maximum rank correlation (method: Spearman) with the Layman metrics matrix. The dissimilarity index for the Layman metrics matrix used was “mahalanobis”, a measure that accounts for correlations across multiple variables. Selected environmental variables, excluding the variables EC and O_2 due to small sample size and variability of measures during the course of the day, were used for the dbRDA, which can show if an explanatory variable has significant impact on the response variables and how much of the variation is explained by each axis. (function *dbrda*, package *vegan*, 50). Selected variables are: nutrient concentration sum, number of pesticide detections, TU_{sum} for algae, and TU_{sum} for invertebrates. To test for the significance of the model, a permutation test on terms and axes was carried out ($p \leq 0.05$; function *anova.cca*, package *vegan*, 50). The calculation of the distance-based Redundancy Analysis (dbRDA) was performed for the data set with corrected $\delta^{15}N$ values.

3. Results

Formatted: Font: Bold

Results for the environmental parameters, nutrient concentration and toxicities of the water samples measured to describe the quality of the water bodies (Table 3).

Formatted: Normal, Justified, Space After: 6 pt, Line spacing: Double

Formatted: Font: Not Bold

Table 3: Overview of the measured water quality and environmental parameters for the seven water bodies. EC = electric conductivity

Sample	C °	EC [µS/cm]	pH	O_2 [mg/L]	NH_4^+ [mg/L]	NO_2^- [mg/L]	NO_3^- [mg/L]	PO_4^{3-} [mg/L]	NH_4^+ NO_2^- $+NO_3^-$ [mg/L]	TUsum algae	TUsum inverte-brates	No. of habi-tats	[illegible] detec-tions
Pond 1	7.4	1570	6.2	5.2	0.01	0	0.2	0.25	0.21	-2.686	-5.221	3	3
Pond 2	10.5	1112	6.5	10.6	0.09	0	0.14	0.05	0.23	-4.057	-5.074	2	1
Pond 3	11.2	881	7	13.8	0.08	0	0.23	0.11	0.31	-2.650	-4.632	3	4
Ditch 1	9.7	588	7	11.5	0.86	0	1.1	0.44	1.96	-2.685	-5.428	2	4
Ditch 2	8.3	815	7.6	6.1	0.08	0	0.72	0.06	0.8	-3.253	-5.116	1	6
Ditch 3	11	928	7	8.8	0.08	0	0.36	0.06	0.44	-2.614	-4.817	3	7
Ditch 4	12.7	900	8	8.8	0.08	0	1.32	0.03	1.4	-3.456	-4.925	2	7

Formatted Table

The raw data on the stable isotope composition ($\delta^{13}C$ and $\delta^{15}N$) of the consumers and sampled resources are available at IsoBank repository (in submission).

3.1 Isotopic niches of communities in ponds and ditches

Formatted: Font: Bold

After correction for the influence of organic fertilizers and of maize, the $\delta^{13}C$ and $\delta^{15}N$ values of consumers show a similar range in ditches and in ponds (Fig. 4 and S2 Fig.). The biplot shows that ponds have a slightly larger ellipse area but that the water body types are comparable in general (Fig. 4).

Fig 4: Biplots for the macroinvertebrate data. The left plot is corrected for $\delta^{15}N$ influence of organic fertilizers on ditches. The right plot is corrected for $\delta^{15}N$ as well as $\delta^{13}C$ values for the influence of C4-plant maize on ~~one~~ pond 3. Ellipses contain 40% of the data.

The ~~probable~~ posterior mode of the $\delta^{15}N$ range is comparable between the two water body types (after correction for organic fertilization) (Fig. 5 a). ~~The probable mode of the~~The typical range of $\delta^{13}C$ ~~is clearly~~values is statistically significant distinct in the two water body types and larger in ponds (Fig. 5 b). The ~~probable~~ posterior distribution of TA and thus total niche width and the spacing of individuals - CD is statistically significantly larger in pond communities compared to communities in ditches (Fig. 5 b, e). The ~~probable~~ posterior values for the MNND (Fig. 5 c) and thus clustering of individuals within the community is larger in ponds, where the evenness of density is similar in ponds and ditches (Fig. 5 ~~e,~~ f).

Fig 5: SIBER density plots for the Layman metrics computed via Bayesian statitics, based on the dataset corrected for $\delta^{15}N$. Black dots show the modes. Shaded boxes represent the 50 %, 75 %, and 95 % confidence intervals, from dark to

light red/blue. Grouping is based on a combination of the individual small water body (7 SWB) and the six FFGs (in total 37 groups), the communities are assigned to the SWB types (pond, ditch). δ^{15}N values of the ditches are corrected for the influence of organic fertilizers.

~~When the maize influence is corrected, the δ^{13}C values are comparable between water body types (Fig. 4), but the δ^{15}N values are slightly larger in ponds.~~ For an illustration of the Layman.B metrics calculated for both stable isotopes corrected, see also table S3.

3.2 Comparison of functional feeding groups

There are ~~clear~~ observable differences for the SEA.B modes and confidence intervals between FFGs, but there is no ~~clear~~ pattern visible when comparing FFGs in ponds and ditches. SEA.B and SEAc modes for *shredders* are higher in ditches than in ponds (Fig. 6). The groups *omnivore*~~*indifferent*~~ and *grazer/scraper* have larger SEA.B/SEAc modes in ponds (Fig. 6). Values for *predators* and *collector/gatherers* are similar in the two water body types (Fig. 6). The credible intervals for *predators* in ponds and ditches are rather small, which shows that the uncertainty is low and that the dataset provides a precise representation of the population mean for this group. ~~The FFG *p*~~P*redators* ~~was~~ were in general more abundant and diverse in ditches.~~,~~ ~~where they form six~~Six taxa groups and 13 families and were recorded ~~with a mean abundance of 42.25 individuals~~there, ~~whereas in ponds they form~~while five taxa groups and eight families ~~and~~ were recorded ~~with a mean abundance of 17.3 individuals~~in ponds (S5 Table). ~~The groups *indifferent* and *grazer/scraper* have larger SEA.B/SEAc modes in ponds (Fig. 6).~~

Fig 6: SIBER density plots computed via Bayesian analysis using corrected δ^{15}N and δ^{13}C values. Plots are shown for the FFGs in ponds (red) and ditches (blue). Black dots are the modes of SEA.B. The grey X indicates the size-corrected standard ellipse area (SEAc) mode. Shaded boxes represent the 50 %, 75 %, and 95 % confidence intervals.

SEA.B niche overlaps for the FFGs between ponds and ditches vary between 15 and 22.9 % (corrected only for δ^{15}N) and between 22.9 to 44.7 % when the data is corrected for δ^{15}N and δ^{13}C (Table 4). For *shredders* the overlap is the same for both correction approaches, as there were no *shredders* found in the maize pond and thus no δ^{13}C correction was needed. For the corrected for δ^{15}N and δ^{13}C values, niche overlaps for *predators* (44.7 %) are largest in ponds and ditches. *Shredders* show least similarity in niches between ponds and ditches (22.9 %).

Formatted: Font: Bold

Table 4: Niche overlaps of ponds and ditches for the FFGs calculated for two datasets. The overlap for collector/filterer cannot be calculated due to their absence in pond samples.

Functional Feeding Group	Niche overlaps of ponds and ditches in %, corrected for N values	Niche overlaps of ponds and ditches in %, corrected for C and N values
Collector/gatherer	15	28.4
Grazer/scraper	16.9	29.8
~~Indifferent~~Omnivore	18.6	38.6
Predator	20	44.7
Shredder	22.9	22.9

3.3 Effects of agricultural stressors on the observed results

Formatted: Font: Bold

Water quality parameters measured in the water samples indicate a low number~~s~~ of pesticides in ponds (M = 2.7 µg/L, SD = 1.5 µg/L) and high numbers of pesticides in ditches (M = 6 µg/L, SD = 1.4 µg/L). ~~Mean value equals 2.7 ±1.5 µg/L pesticides detected in ponds and 6 ± 1.4 µg/L pesticides detected in ditches.~~ 81 % of the total pesticides detected ~~overall~~ (ponds and ditches) are herbicides and 19 % are fungicides. Furthermore, nutrient concentration sum is larger in ditches (~~mean value of~~M = 1.15, SD = ~~±~~0.7 mg/L) compared to ponds (~~mean value of~~M = 0.25 ~~±~~, SD = 0.05 mg/L). Dissolved oxygen concentrations vary from 5.2 mg/L to 13.8 mg/L in ponds and from 6.1 mg/L to 11.5 mg/L in ditches. EC varies from 881 to 1570 µS/cm in ponds and from 588 to 928 µS/cm in ditches.

The dbRDA plot does not show a clear pattern for ponds and ditches (Fig. 7).~~; the water bodies are influenced individually by different parameters. However,~~The isotopic composition of benthic invertebrates and the resulting Layman metrics ~~seem to be either influenced~~of pond 3 and 2 are influenced by the toxicity of water samples for invertebrates (TUsum_invert). Pond 1 and the ditches, except ditch 2, are influenced ~~or~~ by ~~a combination of~~ the toxicity of water samples for algae (TUsum_algae), the concentration of nutrients (nutrients_sum), and the number of pesticides found (N_pesticides). Constrained variables at the first axis explain 75 % of the variance. However, the permutation test resulted in no statistical difference (p ≤ 0.05) for the tested model, model parameters, or axes.

Fig 7: dbRDA plot, distance measure = Euclidean. Water bodies are plotted according to the Layman metrics calculated for the stable isotope results of the communities. Environmental parameters with significant correlations ~~with the water bodies~~ are plotted in blue. The length of the arrows shows how much the variable correlates with the water bodies.

4. Discussion

The two water body types were in general comparable after the correction of $\delta^{15}N$ for the influence of organic fertilizer, which was clearly visible in consumers in ditches. Such relative enrichment was also found by (51) in urban and agricultural streams compared to woodland streams. ~~However, the effects of agriculture on benthic invertebrate communities could not be depicted by the isotopic niches.~~ However, within the agricultural affected sites studied here, isotopic niches could not depict benthic invertebrate community changes except for general impacts on basal C and N resources (e.g. by fertilization or maize/C4 cropping). Also, the comparison of the isotope niches of the two water body types, pond and ditch, did not reveal any clear patterns when analysing the FFGs.

The hypothesis that we expect simplified trophic structures in ditches due to agricultural activity and low habitat variability can only be partially confirmed. The finding that the likely distribution of total niche width and distances between the individuals is greater in pond communities than in ditch communities indicates that more resources/habitats are available in ponds, which lead to more extreme isotope ratios (32). Furthermore, consumers are more divergent in ponds in terms of the isotopic niche MNND, while the evenness of density SDNND is similar in ponds and ditches. This indicates less clustering in pond communities at the same evenness level, which in turn indicates more available resources and/or habitats in ponds that were evenly utilized and colonised (52).

Even though there were no clear patterns visible when comparing FFGs, some differences were pronounced. Striking was the finding that c~~C~~ontrary to our expectations, the FFG *predator* was more ~~abundant and~~ diverse in ditches. ~~Therefore, functional redundancy for this feeding type can be assumed.~~ The high ~~abundance and~~ diversity of *predators* in ditches could be explained by the availability of a high number of primary producers facilitated by high nutrients inputs and thus high primary production, which leads to more available prey (53,54)~~.~~. The influence of changes on the base of the food web propagated to higher trophic levels was also observed by (53), where nutrient pollution promoted biofilm production and lead to an increase in herbivory and carnivory by omnivores. This positive influence of fertilization might be explained by intermediate but not high

Formatted: Font: Bold

Field Code Changed

Field Code Changed

Field Code Changed

Field Code Changed

nutrient enrichment, as in the case of (55), which found highest densities of invertebrates at intermediate levels of nutrient enrichment (primarily of phosphate). The high species number is probably stimulated by bottom-up effects of nutrient enrichment (51,56). Although we can assume that the ditches have been enriched for several years, the effects of long-term enrichment predicted by (56), namely the reduction in species numbers and the shift in community structure, were not observed in the ditch system studied when we compare it to the ponds.

However in contrast to results from (53), the nutrient pollution in ditches did not lead to more homogenous communities for the FFG *predator* in ditches compared to ponds, instead the opposite is the case. In ditches, the number of recorded families belonging to the group of predators is nearly twice as high compared to ponds. ~~The high secondary production is probably stimulated by bottom-up effects of nutrient enrichment~~ (51,56)~~. Although we can assume that the ditches have been enriched for several years, the effects of long-term enrichment predicted by~~ (56)~~, namely the reduction in secondary production and the shift in community structure, were not observed in the ditch system studied when we compare it to the ponds.~~ Therefore, functional redundancy for this feeding type can be assumed (57) which is necessary to ensure resilient systems (58). ~~This positive influence of fertilization might be explained by intermediate but not high nutrient enrichment, as in the case of~~ (55)~~, which found highest densities of invertebrates at intermediate levels of nutrient enrichment (primarily of phosphate).~~

The ~~*indifferent*~~ *omnivore* FFG expressed a variety of feeding strategies. In pond 2 they were clearly mostly predatory with two clusters formed. One cluster formed by~~some~~ individuals ~~forming a cluster~~ with the highest $\delta^{15}N$ values in that water body and a second cluster with the $\delta^{15}N$ values on the same level as the FFG *predators* in that pond (S 4 Table). In the ditches 2, 3 and 4 they were predatory as well but $\delta^{15}N$ values were on the same level as the values of the predators. Some individuals classified as ~~indifferent~~ *omnivore* showed very low $\delta^{15}N$ values which indicates an herbivorous ~~lifestyle~~feeding preference. Clusters on the upper and lower level of $\delta^{15}N$ values are visible in four water bodies (two ditches and two ponds) which shows the shift of omnivores towards either herbivorous or predatory

Field Code Changed

Field Code Changed

Field Code Changed

Field Code Changed

Field Code Changed

Field Code Changed

Field Code Changed

Formatted: Font: Italic

Formatted: Font: Italic

lifestyles as stated by (53). However, according to (53), this shift occurs in polluted water bodies, thus also the ponds ~~probably~~ show signs of communities under the influence of agricultural stressors.

The FFG *collector/gatherer* was dominated by ephemeropterans (*Cloeon* sp.) in both water body types which explains the similarity of SEA.c modes in the water body types and shows that this FFG is similar in its resource use in both water body types.

The larger SEA.c mode for FFG *grazer/scraper* in ponds could be explained by a ~~wider~~ larger number of microhabitats and/or more resources ~~base~~ available in ponds, which had a more diverse habitat in and around the water body compared to the ditches~~, which were dominated by grasses~~ in this study. For lake ecosystems t~~T~~he lower diversity of littoral habitats was also found to be associated with lower numbers of trophic links and less resource and consumer diversity ~~by~~ (59) ~~for lake ecosystems~~. This would mean that the nutrient-rich and thus primary producer-rich ditches are not diverse in resources but that some resources are very abundant. ~~.~~ Additionally, the ~~wider~~ larger number of microhabitats and/or more resources used can also ~~be~~ explain~~ed by higher taxa richness~~ commonly observed patterns of higher taxa richness and the associated possible different preferences in ponds (8–10). ~~.~~ Two *grazer/scraper* taxa groups (Diptera, Gastropoda) and five families were found in ponds, but only one *grazer/scraper* taxa group (Gastropoda) and three families in ditches. In eutrophic waters cyanobacteria blooms can occur which are toxic for other primary producers and can lower the number of *grazers*, but the concentration of cyanobacteria was high for one pond only (data not shown). This could be a measurement of a peak value that had already been exceeded in the other waters, as cyanobacterial blooms can occur very quickly.

The absence of a clear pattern is also underlined by the dbRDA. The configuration of the water bodies shows that they are influenced ~~on a case-by-case basis by the measured~~individually by different parameters, and other factors which were probably not detected. In general, the composition of benthic invertebrates and the resulting Layman metrics appear to be influenced either at the production level (indicated by the variables nutrients sum, toxicity for algae, and number of pesticides detected (mainly herbicides)) or at the consumer level (indicated by the toxicity for invertebrates).

Field Code Changed

Field Code Changed

Formatted: Font: Italic

Formatted: Font: Not Italic

Field Code Changed

Ponds 2 and 3 are similar to each other and are largely influenced on the consumer level. The four ditches and pond 1 are more strongly influenced at the primary producer level, so bottom-up effects are also probably stronger here.

Small and uneven sample sizes are principally of disadvantage, but the Bayesian statistic approach with the SEA.c should enable an unbiased, robust analysis (31). Nonetheless, the FFGs ~~*indifferent*~~ *omnivore* in ponds and *collector/filterer* in ditches show wide credible intervals that indicate a high degree of opportunism and variability in their feeding strategies which is consistent for the FFG *omnivore*. For the FFG *collector/filterer* ~~and~~ further sampling would be needed to predict modes with less uncertainty.

The FFG *collector/filterer* was sampled with below three individuals in ponds thus the data was not used in the analysis. Also in ditches their occurrence was low (10 Bivalvia, 2 Diptera, see S5 Table). It is only possible to speculate about the reasons, but potential factors could be: i) time of sampling, low occurrence of collector/filterer in November as their food base is low, ii) hydrological conditions and sedimentation in ponds which are not favourable for e.g. Bivalvia. As *collector/filterer* were present in low numbers in both ponds and ditches (only in the ponds excluded from the analysis), we did not discuss this further.

Additionally, the time of sampling (autumn) has to be taken into account and has most probably influenced the detection of taxa and pesticides. Information on community composition may have been skewed toward a less diverse composition, with lower numbers of especially Ephemeroptera, Trichoptera, and Diptera~~ns~~.

Although higher numbers of pesticides were found in ditches compared to ponds, this impact does not transport into the expected toxicity gradient for algae and invertebrates, presumably due to the application of low-toxicity pesticide products by the farmers. Additionally, (60) recently found patterns of synchronous toxicity levels towards algae and aquatic plants between water bodies with a

Field Code Changed

Formatted: Font: Italic

Formatted: Font: Italic

Formatted: Font: Italic

Formatted: Font: Italic

Formatted: Left

Field Code Changed

comparable distance to each other like in our study. This should be accounted for in further studies, and future sampling between the months of April and June is generally recommended (60,61). ~~Although higher numbers of pesticides were found in ditches compared to ponds, the lack of a strong toxicity gradient for algae, invertebrates, and water plants made the analysis challenging. This should be adjusted in further studies, and sampling between the months of April and June is recommended.~~

Field Code Changed

~~Constrained variables at the first axis of the dbRDA explained 75 % of the variance. However, the permutation test resulted in no statistical difference (p > 0.05) for the tested model, model parameters, or axes.~~ Furthermore, t~~T~~he high explanatory power of 75 % of the constrained variables on the first axis most likely resulted from running the model on a small dataset and with a high number of explanatory variables.

5. Conclusion

Formatted: Font: Bold

With the obtained dataset, we were unable to recognize clear patterns of the effects of agriculture on isotopic niches of the benthic invertebrates. Agricultural activity and a homogenous environment did not lead to simplified structures in the ditches compared to ponds~~, but it must be assumed that the pond communities are also already influenced by agricultural activity and lack the image of a "realistic" control community~~. Ecological systems respond in complex ways to the presence of abiotic and biotic stressors. Particularly small systems are unstable and exposures are often unclear, leading to ambiguous responses and uncertain patterns. The individual water bodies are influenced by different parameters, and the impacts of individual structures within each water body are more pronounced than influences based on the type of water body, at least for the dataset present. Larger dataset are promising options to overcome this issues, which to our knowledge are not available for small water bodies in the agricultural landscapes in Europe. Additionally, selected water bodies should be monitored more closely and analysed on a broader temporal scale to detect potential effects of agriculture on benthic invertebrate communities over time.

Acknowledgments

The authors thank Marlen Heinz, Manuel König, Karin Meinikmann, Maren Stockmann, and Elke Zeidler for their assistance during the field sampling campaign. Furthermore, we thank Dominique Conrad, Michael Glitschka, Christine Reichmann, Ina Stachewicz, and Gabrielle Smykalla for the sample preparation and pesticide and nutrient analysis. In addition, we thank Dominique Conrad and Jörg Stauch for their help in preparing the biological samples. Thanks also to Matthias Stähler for discussions on analytical methods and Mario Brauns, Kristin Scharnweber, René Sahm, and Matthias Pilecky for fruitful discussions on data analysis. Furthermore, we thank Holly Blevins for proofreading.

Formatted: Font: Bold

References

1. Foley JA, Ramankutty N, Brauman KA, Cassidy ES, Gerber JS, Johnston M, et al. Solutions for a cultivated planet. Nature 2011;478(7369):337–42. PubMed PMID: 21993620.
2. Tscharntke T, Klein AM, Kruess A, Steffan-Dewenter I, Thies C. Landscape perspectives on agricultural intensification and biodiversity – ecosystem service management. Ecol Lett 2005;8(8):857–74.
3. Matson PA, Parton WJ, Power AG, Swift MJ. Agricultural intensification and ecosystem properties. Science 1997;277(5325):504–9. PubMed PMID: 20662149.
4. Stoate C, Báldi A, Beja P, Boatman ND, Herzon I, van Doorn A, et al. Ecological impacts of early 21st century agricultural change in Europe--a review. J Environ Manage 2009;91(1):22–46. PubMed PMID: 19717221.
5. Hill MJ, Chadd RP, Morris N, Swaine JD, Wood PJ. Aquatic macroinvertebrate biodiversity associated with artificial agricultural drainage ditches. Hydrobiologia 2016;776(1):249–60.

Formatted: German (Germany)

6. Tews J, Brose U, Grimm V, Tielbörger K, Wichmann MC, Schwager M, et al. Animal species diversity driven by habitat heterogeneity/diversity: the importance of keystone structures. Journal of Biogeography 2004;31(1):79–92.
7. Rolke D, Jaenicke B, Pfaender J, Rothe U. Drainage ditches as important habitat for species diversity and rare species of aquatic beetles in agricultural landscapes (Insecta: Coleoptera). J Limnol 2018;77(3).
8. Biggs J, Fumetti S von, Kelly-Quinn M. The importance of small waterbodies for biodiversity and ecosystem services: implications for policy makers. Hydrobiologia 2017;793(1):3–39.
9. Williams P, Whitfield M, Biggs J, Bray S, Fox G, Nicolet P, et al. Comparative biodiversity of rivers, streams, ditches and ponds in an agricultural landscape in Southern England. Biological Conservation 2004;115(2):329–41.
10. Davies BR, Biggs J, Williams PJ, Lee JT, Thompson S. A comparison of the catchment sizes of rivers, streams, ponds, ditches and lakes: implications for protecting aquatic biodiversity in an agricultural landscape. Hydrobiologia 2008;597(1):7–17.
11. Darwall W, Bremerich V, Wever A de, Dell AI, Freyhof J, Gessner MO, et al. The Alliance for Freshwater Life : A global call to unite efforts for freshwater biodiversity science and conservation. Aquatic Conserv: Mar Freshw Ecosyst 2018;28(4):1015–22.
12. Trau FN, Fisch K, Lorenz S. Habitat type strongly influences the structural benthic invertebrate community composition in a landscape characterized by ubiquitous, long-term agricultural stress. Inland Waters 2023:1–13.
13. Lynch AJ, Cooke SJ, Arthington AH, Baigun C, Bossenbroek L, Dickens C, et al. People need freshwater biodiversity. WIREs Water 2023.
14. Fehlinger L, Misteli B, Morant D, Juvigny-Khenafou N, Cunillera-Montcusí D, Chaguaceda F, et al. The ecological role of permanent ponds in Europe: a review of dietary linkages to terrestrial ecosystems via emerging insects. Inland Waters 2023;13(1):30–46.
15. Krynak EM, Yates AG. Benthic invertebrate taxonomic and trait associations with land use in an intensively managed watershed: Implications for indicator identification. Ecological Indicators 2018;93:1050–9.
16. Dolédec S, Phillips N, Scarsbrook M, Riley RH, Townsend CR. Comparison of structural and functional approaches to determining landuse effects on grassland stream invertebrate communities. Journal of the North American Benthological Society 2006(25 (1)):44–60.
17. Villéger S, Ramos Miranda J, Flores Hernández D, Mouillot D. Contrasting changes in taxonomic vs. functional diversity of tropical fish communities after habitat degradation. Ecol Appl 2010;20(6):1512–22. PubMed PMID: 20945756.

18. Kemp VA, Grey J, Hemprich-Bennett D, Rossiter SJ, Lewis OT, Wilkinson CL, et al. Changes in trophic ecology of mobile predators in response to rainforest degradation. Journal of Applied Ecology 2023;60(6):1139–48.
19. Múrria C, Iturrarte G, Gutiérrez-Cánovas C. A trait space at an overarching scale yields more conclusive macroecological patterns of functional diversity. Global Ecol. Biogeogr. 2020;29(10):1729–42.
20. Ao S, Chiu M-C, Lin X, Cai Q. Trait selection strategy for functional diversity in freshwater systems: A case framework of macroinvertebrates. Ecological Indicators 2023;153:110450.
21. Zinkann A-C, Wooller MJ, O'Brien D, Iken K. Does feeding type matter? Contribution of organic matter sources to benthic invertebrates on the Arctic Chukchi Sea shelf. Food Webs 2021;29:e00205.
22. Bearhop S, Adams CE, Waldron S, Fuller RA, Macleod H. Determining trophic niche width a novel approach using stable isotope analysis. Journal of Animal Ecology 2004(73):1007–12.
23. Bonnaffé W, Danet A, Leclerc C, Frossard V, Edeline E, Sentis A. The interaction between warming and enrichment accelerates food-web simplification in freshwater systems. Ecol Lett 2024;27(8):e14480. PubMed PMID: 39096032.
24. Guzman I de, Elosegi A, Schiller D von, González JM, Paz LE, Gauzens B, et al. Treated and highly diluted, but wastewater still impacts diversity and energy fluxes of freshwater food webs. J Environ Manage 2023;345:118510. PubMed PMID: 37390732.
25. Layman CA, Quattrochi JP, Peyer CM, Allgeier JE. Niche width collapse in a resilient top predator following ecosystem fragmentation. Ecol Lett 2007;10(10):937–44. PubMed PMID: 17845294; PubMed Central PMCID: PMC2040226.
26. Leibold MA. The Niche Concept Revisited: Mechanistic Models and Community Context. Ecology 1995(76(5)):1371–82.
27. Polechová J, Storch D. Ecological Niche. In: Encyclopedia of Ecology: Elsevier; 2019. p. 72–80.
28. Giménez Gómez VC, Verdú JR, Gómez-Cifuentes A, Vaz-de-Mello FZ, Zurita GA. Influence of land use on the trophic niche overlap of dung beetles in the semideciduous Atlantic forest of Argentina. Insect Conserv Diversity 2018;11(6):554–64.
29. DeNiro MJ, Epstein S, editors. You are what you eat (plus a few‰): the carbon isotope cycle in food chains. Geological Society of America - Abstracts with Programs; 1976.
30. Post DM. Using stable isotopes to estimate trophic position: Models, methods and Assumptions. Ecology 2002;83(3):703–18.
31. Jackson AL, Inger R, Parnell AC, Bearhop S. Comparing isotopic niche widths among and within communities: SIBER - Stable Isotope Bayesian Ellipses in R. J Anim Ecol 2011;80(3):595–602. PubMed PMID: 21401589.
32. Newsome SD, Del Martinez Rio C, Bearhop S, Phillips DL. A niche for isotopic ecology. Frontiers in Ecology and the Environment 2007;5(8):429–36.
33. Parreira de Castro DM, Reis de Carvalho D, Pompeu PdS, Moreira MZ, Nardoto GB, Callisto M. Land Use Influences Niche Size and the Assimilation of Resources by Benthic Macroinvertebrates in Tropical Headwater Streams. PLoS One 2016;11(3):e0150527. PubMed PMID: 26934113; PubMed Central PMCID: PMC4774910.
34. Brauns M, Kneis D, Brabender M, Weitere M. Habitat availability determines food chain length and interaction strength in food webs of a large lowland river. River Research & Apps 2022;38(2):323–33.
35. Chaumet B, Probst J-L, Eon P, Camboulive T, Riboul D, Payré-Suc V, et al. Role of Pond Sediments for Trapping Pesticides in an Agricultural Catchment (Auradé, SW France): Distribution and Controlling Factors. Water 2021;13(13):1734.

Formatted: German (Germany)

36. Hartong H, Schmid C, Kornack I, Wolter A. Landschaftsrahmenplan Landkreis Havelland: Band 2 Bestand und Bewertug. Landkreis Havelland; 2014.
37. Kalettka T, Rudat C. Hydrogeomorphic types of glacially created kettle holes in North-East Germany. Limnologica 2006;36(1):54–64.
38. Schmidt-Kloiber A, Hering D. www.freshwaterecology.info – An online tool that unifies, standardises and codifies more than 20,000 European freshwater organisms and their ecological preferences. Ecological Indicators 2015;53:271–82.
39. COIL. Data Interpretation Guide for Isotopic Analysis; 2021. Available from: www.cobsil.com.
40. Peterson BJ, Fry B. Stable Isotopes in Ecosystem Studies. Annual Review of Ecology and Systematics 1987:293–320.
41. Lewis KA, Tzilivakis J, Warner DJ, Green A. An international database for pesticide risk assessments and management. Human and Ecological Risk Assessment: An International Journal 2016;22(4):1050–64.
42. Olker JH, Elonen CM, Pilli A, Anderson A, Kinziger B, Erickson S, et al. The ECOTOXicology Knowledgebase: A Curated Database of Ecologically Relevant Toxicity Tests to Support Environmental Research and Risk Assessment. Environ Toxicol Chem 2022;41(6):1520–39. PubMed PMID: 35262228; PubMed Central PMCID: PMC9408435.
43. Gholizadeh M, Heydarzadeh m. Functional feeding groups of macroinvertebrates and their relationship with environmental parameters, case study: in Zarin-Gol River. Iranian Journal of Fisheries Sciences 2017:2532–43.
44. Ramírez A, Gutiérrez-Fonseca PE. Functional feeding groups of aquatic insect families in Latin America: a critical analysis and review of existing literature. Rev Biol Trop 2014;62 Suppl 2:155–67. PubMed PMID: 25189076.
45. R core Team. R: A language and environment for statistical computing.; 2023.
46. Jackson A, Parnell A. SIBER: Stable Isotope Bayesian Ellipses in R 2023.
47. Ellison AM. Bayesian inference in ecology. Ecol Letters 2004;7(6):509–20.
48. Jackson A, Parnell A. _SIBER: Stable Isotope Bayesian Ellipses in R_; 2023.
49. R core Team. R: A Language and Environment for Statistical Computing; 2024.
50. Oksanen J, Simpson GL, Blanchet GF, Kindt R, Legendre P, Minchin PR, et al. vegan: Community Ecology Package; 2022.
51. Price EL, Sertić Perić M, Romero GQ, Kratina P. Land use alters trophic redundancy and resource flow through stream food webs. J Anim Ecol 2019;88(5):677–89. PubMed PMID: 30712255.
52. Tatenda Dalu, Ryan J. Wasserman, P. William Froneman, Olaf L. F. Weyl. Trophic isotopic carbon variation increases with pond's hydroperiod: Evidence from an Austral ephemeral ecosystem. Sci Rep 2017(7, 7572).
53. Guzman I de, Montoya JM, Elosegi A, Pérez-Calpe AV, Schiller D von, González JM, et al. Food-web energy fluxes, energy transfer efficiency, and diversity respond distinctively to pollution and water diversion in rivers. Freshwater Biology 2024.
54. Hobbie SE, Villóeger S. Interactive effects of plants, decomposers, herbivores, and predators on nutrient cycling. In: Hanley TC, La Pierre KJ, editors. Trophic Ecology: Cambridge University Press; 2015. p. 233–59.
55. García L, Pardo I, Cross WF, Richardson JS. Moderate nutrient enrichment affects algal and detritus pathways differently in a temperate rainforest stream. Aquat Sci 2017;79(4):941–52.
56. Cross WF, Wallace JB, Rosemond AD, Eggert SL. Whole-system nutrient enrichment increases secondary production in a detritus based ecosystem. Ecology 2006;87(6):1556–65.
57. Petchey OL, Gaston KJ. Functional diversity (FD), species richness and community composition. Ecol Lett 2002;5(3):402–11.
58. Rosenfeld JS. Functional redundancy in ecology and conservation. Oikos 2002(98, 1):156–62.

Formatted: German (Germany)

59. Brauns M, Gücker B, Wagner C, Garcia X-F, Walz N, Pusch MT. Human lakeshore development alters the structure and trophic basis of littoral food webs. Journal of Applied Ecology 2011;48(4):916–25.
60. Lorenz S, Trau FN, Ruf LC, Meinikmann K, Fisch K, Stähler M, et al. Pesticide contamination of small standing water bodies in the agricultural landscape of northeast Germany. Sci Total Environ 2025;975:179250. PubMed PMID: 40184998.
61. Liess M, Liebmann L, Vormeier P, Weisner O, Altenburger R, Borchardt D, et al. Pesticides are the dominant stressors for vulnerable insects in lowland streams. Water Res 2021;201:117262. PubMed PMID: 34118650.

Supporting information

Formatted: Font: Bold

Supporting information 1: Active substances ~~measured~~ (99), with their limits of quantification and detection, measured, as well as the endpoints and test organism used for the toxicity calculations.

Supporting information 2: Biplot for benthic macroinvertebrate before correction for δ15N values from and the influence of organic fertilization. Ellipses incorporate 40% of the data.

Supporting information 3: SIBER density plots for the Layman metrics, computed via Bayesian analysis. Black dots are the modes. Shaded boxes represent the 50 %, 75 % and 95 % confidence intervals, from dark to light red/blue. Grouping is based on the FFG (six groups), the communities are assigned for the water body types (pond, ditch).

Supporting information 4: Representation of the FFGs as coloured dots and ellipses, 40% of the data points are represented by the ellipses.

Supporting information 5: Overview of the sampled FFGs and determination for the ponds and ditches.

Supporting information 6: Layman metrics for ponds and ditches, calculated for the FFGs and the different water bodies as grouping.

Supporting information 7: Biplots for benthic macroinvertebrates in ponds (top) and ditches (bottom). Raw data was corrected with δ15N means of resources.

Supporting information 8: Biplots for benthic macroinvertebrates in ponds (top) and ditches (bottom). Raw data was corrected with δ15N means of resources and δ13C values were corrected for the influence of C4-plant maize.

Rebuttal letter of PONE-D-25-03786, Trau et al., Assessing agricultural effects on benthic invertebrate communities in ponds and ditches using $\delta^{15}N$ and $\delta^{13}C$ isotope niches

Author's response:

We thank the Academic Editor for handling our manuscript and for the careful selection of qualified Reviewers. We are glad to receive such a constructive, detailed, and fair review report. We revised our manuscript according to the comments of the Reviewers.

Comments to the Author

1. Is the manuscript technically sound, and do the data support the conclusions?

The manuscript must describe a technically sound piece of scientific research with data that supports the conclusions. Experiments must have been conducted rigorously, with appropriate controls, replication, and sample sizes. The conclusions must be drawn appropriately based on the data presented.

Reviewer #1: Partly

Reviewer #2: Partly

____

2. Has the statistical analysis been performed appropriately and rigorously?

Reviewer #1: I Don't Know

Reviewer #2: Yes

____

3. Have the authors made all data underlying the findings in their manuscript fully available?

The PLOS Data policy requires authors to make all data underlying the findings described in their manuscript fully available without restriction, with rare exception (please refer to the Data Availability Statement in the manuscript PDF file). The data should be provided as part of the manuscript or its supporting information, or deposited to a public repository. For example, in addition to summary statistics, the data points behind means, medians and variance measures should be available. If there are restrictions on publicly sharing data—e.g. participant privacy or use of data from a third party—those must be specified.

Reviewer #1: Yes

Reviewer #2: Yes

____

4. Is the manuscript presented in an intelligible fashion and written in standard English?

PLOS ONE does not copyedit accepted manuscripts, so the language in submitted articles must be clear, correct, and unambiguous. Any typographical or grammatical errors should be corrected at revision, so please note any specific errors here.

Reviewer #1: Yes

Reviewer #2: Yes

____

Rebuttal letter of PONE-D-25-03786, Trau et al., Assessing agricultural effects on benthic invertebrate communities in ponds and ditches using $\delta^{15}N$ and $\delta^{13}C$ isotope niches

5. Review Comments to the Author

Please use the space provided to explain your answers to the questions above. You may also include additional comments for the author, including concerns about dual publication, research ethics, or publication ethics. (Please upload your review as an attachment if it exceeds 20,000 characters)

---

6. PLOS authors have the option to publish the peer review history of their article (what does this mean?). If published, this will include your full peer review and any attached files.

If you choose "no", your identity will remain anonymous but your review may still be made public.

Do you want your identity to be public for this peer review? For information about this choice, including consent withdrawal, please see our Privacy Policy.

Reviewer #1: Yes: Daniela Cortes Guzman

Reviewer #2: No

Reviewer #1:

I finalized revising the manuscript "Assessing agricultural effects on benthic invertebrate communities in ponds and ditches using $\delta^{15}N$ and $\delta^{13}C$ isotope niches". The topic is interesting and attractive to a broad audience. However, major improvements need to be done in the manuscript. The questions and hypotheses, and how the methods were applied to solve these questions, were rather cryptic for me. It was generally hard to understand from the introduction which is the knowledge gap the authors are trying to fulfill. Moreover, without clear questions, assessing the science behind the methods and results was difficult. My major misunderstanding is why the authors expect differences between the two types of ecosystems assessed, and therefore, why this is an interesting question in the framework of agricultural stressors. I think the authors have an interesting and valuable dataset with the potential of answering interesting ecological questions. However, significant work needs to be done. I left detailed comments for each of the sections.

Author's response:

We thank the reviewer for the time that she invested in evaluating our manuscript and we hope that we have addressed all the concerns raised by the reviewer accordingly.

Comments reviewer 1 (black) with authors responses (green):

Abstract

14: Why do ponds and ditches harbor particularly endangered or scarce taxa? Moreover, why do you mean by scarce, is it low abundant taxa or taxa with low occupancy? I'd recommend removing this last part of the sentence.

Explained in more detail why these water bodies are valuable habitats and replaced scarce with rare.

16 I disagree. Numerous studies have shown the adverse effects of agricultural land use in biodiversity. Perhaps what you mean is that these habitats are likely to face multiple stressors, all derived from agriculture, which results in a problematic complex to address. More importantly, the

reader does not know so far which are these previous approaches and why they were not successful. For example, what do you mean by "classic structural biodiversity metrics"?

Added the word "indirect or multiple" to stressors and examples for the classical metrics mentioned. Problems with previous studies will be elaborated on in the introduction section. The sentence in the abstract should only function as a teaser for the reader.

19: We analyzed the carbon and nitrogen stable isotope composition of the invertebrates and the pesticides and nutrient residues of water.

Addressed.

21 readers do not know what are Standard ellipse and Layman B community metrics. I recommend leaving technical terms out of the abstract.

Addressed. Terms left out and circumscribed as "Estimates of community metrics…"

22 FFG should be introduced earlier.

FFGs are introduced earlier in the abstract when the general approach is explained. Line 18 ff. "Benthic invertebrates, from six different functional feeding groups (FFGs), and water samples were collected from three ponds and four ditches in an agricultural landscape in Brandenburg, Germany."

28 Which differences? You're leaving the reader hanging on the unexpected differences but don't tell them (us) which.

Explanation for predator example added. "…, which was more abundant and diverse in ditches."

28-32 See my previous comment. These sentences are uninformative for readers. We don't know why case-by-case patterns emerged, or where the differences lay. What do you mean by the production or consumption level? How was production measured or estimated? We also don't know which strong fluctuations on parameters did you observed; therefore, we don't know how did you reach this conclusion.

Addressed. Deleted the sentence from the abstract as it is confusing here and will be elaborated in the results and discussion.

30 Remove In the end

Addressed.

Introduction

40 is it specialization, and use of fertilizer and pesticides homogenizing the landscape? I'd say that the expansion of agricultural land is what homogenizes the landscape. Ref 3

I would still argue that it is specialisation and the increased use of inputs in intensive or industrial agriculture that makes a landscape more homogenous as intensive agriculture usually goes hand in hand with larger plots being used for one crop, which can be sprayed and harvested by large machines. The expansion of agriculture is another point that can also lead to homogenization when land is converted to intensive agricultural land. Thus, I kept the sentence but added expansion to the explanations in the sentence.

43 What do you mean by semi-natural habitats? Under less intensive agriculture?

Rebuttal letter of PONE-D-25-03786, Trau et al., Assessing agricultural effects on benthic invertebrate communities in ponds and ditches using δ^{15}N and δ^{13}C isotope niches

I mean habitats such as ponds, ditches, field margins or hedges. In general, habitats that were altered by humans but still have a high biodiversity value. Examples added in text.

45 Starting a paragraph with an example is quite counterintuitive. You haven't introduced yet non-productive areas, so why are you giving us examples?

I connected the paragraph with the paragraph before, as this is the explanation with examples for the semi-natural areas. See also comment above.

49 Same as in the abstract: would you define scarce more precisely.

Replaced scarce with rare.

50 a high richness compared to what? Also, these groups are highly abundant in many aquatic ecosystems; therefore, why are they particularly important in ditches?

I changed the sentence to make my intention with that sentence clear. "Also ditches, which are in close proximity to agricultural practices, have the potential to host diverse communities as well as rare or endangered aquatic species (9) and show a high richness for the groups Coleoptera, Gastropoda, Trichoptera and Heteroptera (9).

51 I think this is your topic sentence for this paragraph; therefore, it should be the first one -or maybe the second one, but at the beginning!

Paragraph reordered:

"Ponds and ditches are biodiversity hotspots and form aquatic corridors in a landscape dominated by agriculture in need of conservation action (5,6). Unfortunately, these small water are usually not included in monitoring programs (5,7), but the importance of particularly ponds, but also ditches, for benthic macroinvertebrate biodiversity and ecosystem services is increasingly gaining recognition in research activities (5,9–14). Ponds are known to harbour high species numbers (9), however also ditches, which are usually in close proximity to agricultural practices, have the potential to host diverse communities as well as rare or endangered aquatic species (5) and show a high richness for the groups Coleoptera, Gastropoda, Trichoptera and Heteroptera (5)."

53 but this contradicts your previous sentence in line 48

Yes true, deleted the contradicting part. The main point here was that those small water bodies are not included in monitoring programs.

57 in which sense is challenging? Actually, invertebrates are one of the most used groups for biodiversity assessment because sampling is comparatively easy and cheap to other taxa, and they are very good indicators of ecosystem status and functions.

Added explanation in the sentence. "…the assessment of water or habitat quality by benthic invertebrate biodiversity is challenging…"

59 But there is extensive literature showing the effects of agriculture on biodiversity. I think you need to be more specific as to why functional approaches outperform, or complement, taxonomic approaches. For example, why would functional approaches be more sensible to persistent stressors or short gradients? Ref 8

Rebuttal letter of PONE-D-25-03786, Trau et al., Assessing agricultural effects on benthic invertebrate communities in ponds and ditches using $\delta^{15}N$ and $\delta^{13}C$ isotope niches

Added: "Additionally species occurrences may be subject to some degree of stochasticity (15) and functional responses can reflect the ecological conditions and habitat change better than taxonomic approaches (16,17)."

64 why were these other traits not included in your analysis? One important effect of agricultural land use is the change in inputs of organic matter to the water bodies; this justifies the use of feeding groups. However, other effects are channelization or modification of the physical structure, which might produce a response in locomotion; changes in productivity (eutrophication), with effects on body size and respiration; changes in temperature, with effects on thermal tolerance and respiration. In other words, you need either to include more traits, or to justify why feeding groups are the best approach to address the effects of agriculture, including persistent stressors and short gradients.

Channelization and physical modification are important factors for (natural) streams (or lakes for physical modification) suffering from anthropogenic pressure. Ditches are per se longitudinal channels, all of which are constantly modified in their physical structure by bank cleaning and mowing. Therefore, we assume communities to be adapted to this environment.

Added in the manuscript: "We selected FFG to address the effects of agriculture as further stressors such as channelization and modification of physical structure do not vary in their intensity in the selected water bodies"

80 this paragraph is disconnected with the rest of the introduction. While important concepts need to be defined, the reader wants to know how the niche concept(s) is related to your own work and very importantly, to your question and knowledge gap. It is your task, as writers, to tell the reader how this concept can help understanding effects of stressors in biodiversity -not give the reader homework . For example, I'd recommend combining this paragraph with the trait paragraph, since the niche is going to be defined based on those traits. This will provide more coherence between your paragraphs and shorten your introduction.

Addressed. Combined with the trait paragraph as suggested. This also makes clear that the adaptation of the niche concept in stable isotope studies is done to create isotopic niches, which are a proxy for the habitat and resource use of a community. Habitat and resources are linked to the land use and agricultural stressors. This link was stressed in the text.

88 we used. You haven't defined isotopic niche nor realized niche. Feeding resources? And which habitats? Do you mean between ponds and ditches? If so, why feeding resources and, therefore, isotopic niches would differ between them?

Described in more detail above in the manuscript.

89 we compared

Addressed

90 which groups?

Addressed. "…compared niche widths of FFGs and the pond and ditch communities based on…"

92 are you assessing more than one stressor? Or is it pesticides the only metric you are using?

No, pesticides and nutrients are examined. Changed in the text as follows: "…influence of agricultural stressors (toxicity of water samples due to pesticide residues and eutrophication due to nutrient inputs)…"

Rebuttal letter of PONE-D-25-03786, Trau et al., Assessing agricultural effects on benthic invertebrate communities in ponds and ditches using $\delta^{15}N$ and $\delta^{13}C$ isotope niches

93 we asked. Can

Adressed

94 I don't fully understand your questions. Are you assessing isotopic niches in a gradient of agricultural stress, as measured by pesticides? Why do you expect differences, or not, between ponds and ditches?

Added in the text: "We assume that ponds and ditches are influenced differently by agricultural stressors. Ditches are expected to be more strongly influenced by agricultural stressors than ponds due to their characteristics, which are a rather homogeneous habitat in and around the water body, a directly farmed environment with the location in the field (grassland) without a riparian buffer. "

Questions adapted: "We asked the following questions, using ponds and ditches as an example.: Can the isotopic niches depict the effects of agriculture on benthic invertebrate communities in the selected water bodies in the study region? Are the observed patterns of isotopic niches of FFGs comparable in ponds and ditches for the FFGs?"

96 to answer these questions…

Adressed

98 what is the difference between niche differentiation and niche specialization? And why reduced niche specialization would result in less overlap? I'd expect the opposite. How about the expectations for ponds? Do ponds have different effects of agriculture?

The term differentiation and specialisation mean the same. Niche differentiation = competing species use the niche in different ways to coexist, different use of resources, portioning of space and time. The word specialisation was replaced with differentiation in the second part of the sentence.

Yes, it should be "larger overlaps". Changed in the text.

It is not clear to me which questions are being answered and how. Is it the question related to differences in niche width due to different levels of agricultural stress? Then, would these effects differ between ponds and ditches? Why? What do you mean by habitat variability? Is this variability inside each water body, or among types?

Added in the text, see above.

Added text at the beginning of the paragraph: "….affecting habitat availability and quality and thus influencing benthic invertebrate food webs and niche size (33,34)."

Added text: "Ditches, for example, are probably more contaminated, but as they are part of a flow system, pollutants can also be transported downstream. Ponds, on the other hand, are better protected from pesticide inputs, but can act as a source and sink for pesticide residues in the sediment (35)."

Methods

103 I think that saying During the 1980s, the wet lowlands… is enough. Moreover, I'd turn the sentence around: The wet lowlands in the Havelländisches region were drained … during the 1980s.

Addressed

Rebuttal letter of PONE-D-25-03786, Trau et al., Assessing agricultural effects on benthic invertebrate communities in ponds and ditches using δ^{15}N and δ^{13}C isotope niches

105: What do you mean by traditional grasslands? As in, they were already agricultural lands but transformed into monoculture crops? Or were they natural grasslands converted to agriculture?

I mean grasslands but extensively used with a number of herbs and grasses growing together, extensively managed. This was changed to grassland, which was seeded with only one type of grass. Changed in the text to: "Traditional, extensively managed and diverse grasslands were converted to seeded grasslands…"

107 What about ditches?

What exactly do you mean? I talk about the ditches in the previous sentences, when talking about the draining of land and the established ditch system in the region.

113 describe the site's characteristics in order, from Pond 1 forward, and ditch 1 on.

Addressed

Table 1: How were these habitats characterized? Do they correspond to the littoral zone, the whole water body? Which area of the water body was estimated or quantified? Same for the riparian zone: which distance from the shore was considered riparian, and how these percentages were estimated/quantified?

Adressed. Text added in the caption. "Habitats were described for the water bodies, when present in the wadeable area of the shore length with more than 5 %. The riparian zone is defined as the area from the edge of the field including the edge strip to the waterline, on this area the growth of grasses etc. was estimated as a percentage."

You could highlight the dominant type of riparian vegetation to help readers focus.

Adressed. Written in bold in table 1

124 Benthic invertebrates were sampled using hand netting…

Addressed. Text reordered. "Benthic invertebrates were sampled using hand netting (mesh size 500 µm) from the habitats (FPOM, CPOM, submerse macrophytes, reed) present in the selected water bodies. Individuals from all FFGs present were sampled and organisms were sorted on-site by taxonomy (to the level of order or family)."

126 meaning that samples were composed by combining all habitats?

Yes. The sampling strategy of every habitat and description of habitats was used to get representative samples and to describe the water bodies, but data was not analysed for the single habitats.

126 how were feeding groups distinguished in the field? Based on morphology?

Yes. Taxonomy changed to morphology in the text.

129 how is dispatch with boiling water humanely? To what end were they kept alive for 24 hours? And why did you use this boiling water method, instead of preservatives like alcohol or formol. This is related to the isotopic signal, but the reader does not know this.

It is humane in the sense that it happens very quickly and the animals hopefully notice as little as possible. They are kept alive for 24 hours so that they can empty their gastrointestinal tract. This material should not be analysed. Information added in the text.

Rebuttal letter of PONE-D-25-03786, Trau et al., Assessing agricultural effects on benthic invertebrate communities in ponds and ditches using δ^{15}N and δ^{13}C isotope niches

131 Explain why their shells were remove.

Addressed. Explanation added in the text. "…to only analyse the isotopic signal of the body tissue of selected animals"

149 I don't see how conductivity and pH can affect the food web structure or isotopic niche. Oxygen and temperature, indirectly, if they reflect productivity?

True. I wanted to say parameters that could function to explain the community's food web structures or isotopic niches of groups. Changed in the text.

175 Why was toxicity for macrophytes and algae estimate?

As we know from parallel studies currently under review there might be indirect effects from pesticides affecting the invertebrate communities. These effects, e.g. herbicide effects on macrophytes or algae can be measured by the toxicity of the samples towards macrophytes and algae. Therefore, it was estimated here and used as variables in the dbRDA. However, in the end only the value for algae was used in the analysis thus I deleted the information on toxicity on macrophytes in the manuscript.

187 So far, FFGs have not being defined or described. What are they, what do they represent, how do you assign an organism to a FFG, or which categories exist. This table should be probably moved to the Results.

Paragraph added to describe the FFGs and what they represent: "Classified as collector/filterer are Bivalvia and the family Culicidae Gen. sp., which filter suspended FPOM from the water column (32). The families of Ephemeroptera and some Dipterans belong to the group collector/gatherers, which feed on sedimented FPOM. Grazers/scrapers graze on surfaces where they feed on algae tissue or biofilm, also they feed on algae or plant tissue (32). In the present dataset, those are mostly Gastropods and families belonging to the group are for example Lymnaeidae or Planorbidae. Within the group indifferent families are gathered that could not be assigned to one distinct group. In the present dataset Chironomidae, Corixidae, Haliplidae and Stratiomyidae were assigned to the group. Predators hunt on prey and examples are Dytiscidae, Ceratopogonidae or Coenagrionidae. Typical shredders are Gammaridae or Asselidae, which feed on plant tissue, fallen leaves or CPOM (32). A detailed list of the families belonging to the FFGs can be found in the table supporting Information 5."

The authors decided to not move the table to the results section as it is describing the database for the following methods and thus should be kept in the material and methods section.

188 family determination based on which literature? FFGs can vary in different regions because organisms are flexible in their diets, even given certain morphology. Moreover, they can usually be assigned to more than one category. Even in the book of Merrit, Cummins, and Berg (the "inventors" of FFGs), taxa are usually categorized in more than one group.

Table 3. CPOM and FPOM are in the legend but not the table. Columns after the TU are not explained. How were all these metrics quantified and to what aim? Move to Results.

The table was very wide and some columns were probably not visible for you. However, I can delete them here as the information on habitats and riparian zone is already given in table 1. Column reed in % deleted, as the information is already given in table 1. Columns after TU with information on algae concentrations deleted, as they are not used in the further analysis of the data.

Rebuttal letter of PONE-D-25-03786, Trau et al., Assessing agricultural effects on benthic invertebrate communities in ponds and ditches using δ^{15}N and δ^{13}C isotope niches

Additionally a sentence before table three was added as explanation and the table was moved to results as suggested.

200 was observed

Addressed

201 of all invertebrates or for primary consumers? This does not seem to be an appropriate justification, from my point of view, precisely because you are looking for potential differences between water body types. To my knowledge, there are two ways of correcting dN: first, by using a basal resource dN value as a baseline; this might have the problem of having different values among sites, if they differ in the amount of fertilizer. Second, using the dN value of the primary consumer with the lowest dN to obtain comparable trophic positions across sites.

The difference of d15N values was subtracted from all consumers in the ditches. We followed the presented approach as the differences between all FFGs between ponds and ditches in their d15N values were visible and most likely originating from fertilization in the ditches (farmers data not shown). See also supporting information 2. Thus, we only correct for the effect of fertilization to compare FFGs between ditches and ponds to avoid biases by the fertilization regime. Therefore, we calculated the correction value for d15N from all invertebrates in ponds and ditches and used it to correct all invertebrates in ditches. As you already stated the commonly used correction with basal resources does not correct the fertilizer effect. The second method you propose, using the d15N value of the lowest primary consumer to correct will also not solve the strong effect of fertilization that might override possible patterns. Therefore, we used the most appropriate method for this special issue in our data.

204 difference between what and what?

The difference between the d15N mean in ditches and d15N mean in ponds. More explanation added in the text.

209 what difference?

The difference between the mean d13C values from pond 1/ 2 and the mean d13C values from pond 3. More explanation added in the text.

210 an alternative is centering or scaling the deltas of all sites to obtain comparable values, but I don't understand why to alter the variability of the sites showing effects of agriculture, if that's your question? Moreover, it seems odd and a bit arbitrary to transform values of one site.

Yes, we do observe the effect of agriculture, in this case maize cultivation. This is a special effect as maize is a C4 plant and has different isotope composition than other plants such as grasses, wheat and so on. Our intention was not to measure the effect of a single crop (which in case of C4 plant cropping is just a random effect) when comparing general responses of ponds and ditches. If we would keep original C values the C4 maize effect will override everything else we might be able to observe in the data. Thus we only corrected the d13C values in pond 3 which is the only pond influenced by C4 cropping..

212 Explain what these analyses are and why were they performed

Sentence deleted here and moved to the chapters 2.5.3 and 2.5.4 where the statistical analysis are explained.

Rebuttal letter of PONE-D-25-03786, Trau et al., Assessing agricultural effects on benthic invertebrate communities in ponds and ditches using $\delta^{15}N$ and $\delta^{13}C$ isotope niches

233 what information

CD provides information on niche width and species spacing

235 to what end? What is the information provided by these metrics?

Added in the manuscript: "The Bayesian estimates provide robust measures of probability and can reflect model uncertainty by averaging many possible models into one single model (45). The SEA.c is based on SEA.B, which describes isotopic niche with, and corrected for small sample sizes for unbiased analysis (31). "

237 I don't understand why do you want to correct for the effects of agriculture? Also, is this another correction or the same one mentioned in the previous subsections?

No, it is the correction from chapters 2.5.1 and 2.5.2. As explained in more detail above we are not correcting for the effect of agriculture itself but for the random effect of C4 cropping at this specific field compared to the rest of the field where C3 plants were cultivated. We are correcting for these effects, as they would override further patterns behind the C3/C4 crop difference due to the dimension of d13C scaling between C3 and C4for further analysis I used the corrected data to compare the FFGs in the two water body types.

Results

265 it hasn't been explained or justified why these two types of habitats would differ in the isotopic composition

Text was changed and further explanation on this issue has been added in detail at the end of the introduction as follows:

" We compared niche widths of FFGs and the pond and ditch communities based on a geometric approach originally developed by (25) to examine the isotopic niches of benthic invertebrates in ponds and ditches. The aim is to assess the influence of agricultural stressors (toxicity of water samples due to pesticide residues and eutrophication due to nutrient inputs) on the isotopic niche metrics.

We assume that ponds and ditches are affected differently by agricultural stressors, affecting habitat availability and quality and thus influencing benthic invertebrate food webs and niche size (33,34). Ditches are expected to be more strongly influenced by agricultural stressors than ponds due to their characteristics, which are a rather homogeneous habitat in and around the water body, a directly farmed environment with the location in the field (grassland) without a riparian buffer. Ditches, for example, are probably more contaminated, but as they are part of a flow system, pollutants can also be transported downstream. Ponds, on the other hand, are better protected from pesticide inputs, but can act as a source and sink for pesticide residues in the sediment (35). The water body type's ponds and ditches are used as gradients of agricultural effects, including nutrient enrichment, pesticides, and the proportion of surrounding crops to test our hypotheses. We asked the following questions, using ponds and ditches. Can the isotopic niches depict the effects of agriculture on benthic invertebrate communities in the selected water bodies in the study region? Are the observed patterns of isotopic niches of FFGs comparable in ponds and ditches for the FFGs? To answers these questions, we hypothesize that agricultural activity and low habitat variability in ditches have led to simplified trophic structures, which will be shown by wider isotopic niches, reduced niche differentiation, and larger niche overlap due to reduced differentiation of the FFGs and more generalists present."

Figure 4 the resolution of the figure is very poor, please improve it.

I don't understand why resolution was poor for you. Maybe the journal send lower resolutions to the reviewers. Quality is at 300 dpi.

Rebuttal letter of PONE-D-25-03786, Trau et al., Assessing agricultural effects on benthic invertebrate communities in ponds and ditches using $\delta^{15}N$ and $\delta^{13}C$ isotope niches

271 replace clearly by a more appropriate term, like statistically

Addressed. The typical range of δ13C values is distinct in the two water body types and larger in ponds (Fig. 5 b).

274 … for the MNND (Fig 5c)…

Addressed

275 evenness is Fig 5f, not e

Addressed

284 Which clear differences, between what and what? Please, try to avoid using words like "clear" or "clearly" since patterns might not be as clear or obvious for the readers.

Addressed.

Figure 6: the boxes correspond to SEA.B or SEAc?

To both. It is explained in the description for the figure as follows: "Black dots are the modes of SEA.B. The grey X indicates the size-corrected standard ellipse area (SEAc) mode."

284 this paragraph needs to be more organized. It is mixing the isotope values with abundances and diversity for one FFG.

Addressed. Paragraph reordered and simplified.

306-309 these sentences are quite hard to read; please, revise the grammar.

Addressed. Text revised

321 were the explanatory variables scaled before running the analysis?

Yes, they were scaled with *scale* function of the *base* package. This was explained in chapter 2.5.4 multivariate statistics in the methods section.

Discussion

327 but how was this tested?

Addressed. Changed in the text as follows: " However, within the agricultural affected sites studied here, isotopic niches could not depict benthic invertebrate community changes except for general impacts on basal C and N resources (e.g. by fertilization or C4 cropping)."

339 why did you expect more abundance and diversity in ponds?

Ponds in general are more diverse as stated by various authors (Biggs J, Fumetti S von, Kelly-Quinn M. The importance of small waterbodies for biodiversity and ecosystem services: implications for policy makers. Hydrobiologia 2017;793(1):3–39 and Williams P, Whitfield M, Biggs J, Bray S, Fox G, Nicolet P, et al. Comparative biodiversity of rivers, streams, ditches and ponds in an agricultural landscape in Southern England. Biological Conservation 2004;115(2):329–41 and Davies BR, Biggs J, Williams PJ, Lee JT, Thompson S. A comparison of the catchment sizes of rivers, streams, ponds, ditches and lakes: implications for protecting aquatic biodiversity in an agricultural landscape. Hydrobiologia 2008;597(1):7–17. This is also stated in the introduction from line 51 ff.

Rebuttal letter of PONE-D-25-03786, Trau et al., Assessing agricultural effects on benthic invertebrate communities in ponds and ditches using $\delta^{15}N$ and $\delta^{13}C$ isotope niches

341 was any of this measured? Is the relative abundance of herbivores significatively higher in ditches? This all seems very speculative

Term abundance deleted in the sentences.

Reference added: Hobbie, Sarah E.; Villóeger, Sóebastien (2015): Interactive effects of plants, decomposers, herbivores, and predators on nutrient cycling. In: Torrance C. Hanley und Kimberly J. La Pierre (Hg.): Trophic Ecology: Cambridge University Press, S. 233–259.

346 homogenous in which sense? How was homogeneity measured?

Homogenous is meant here as low in number of families. However as stated in the results (section: 3.2) the FFG predator is a diverse/heterogenous group in ditches, compared to ponds.

347 then, why citing 46?

The reference is cited as it explains my statement before.

349 I don't think secondary production is an appropriate term here, since this was not measured. Secondary production is a complex metric requiring, at the very least, biomass estimation.

Yes, true. Secondary production was substituted by species number in the text.

351 again, secondary production was not measured.

Yes, see comment above.

355 this paragraph is generally confusing. I'd recommend you explain your results at the beginning, as a topic sentence, and then develop the ideas that support your findings. In the current version, sentences supporting and contradicting your findings are mixed making it hard for readers to get the main message you want to transmit.

Agreed. The paragraph was slightly changed and rearranged as follows:

"Even though there were no clear patterns visible when comparing FFGs, some differences were pronounced. Striking was the finding that contrary to our expectations, the FFG predator was more diverse in ditches. The high diversity of predators in ditches could be explained by the availability of a high number of primary producers facilitated by high nutrients inputs and thus high primary production, which leads to more available prey (52,53). The influence of changes on the base of the food web propagated to higher trophic levels was also observed by (53), where nutrient pollution promoted biofilm production and lead to an increase in herbivory and carnivory by omnivores. This positive influence of fertilization might be explained by intermediate but not high nutrient enrichment, as in the case of (55), which found highest densities of invertebrates at intermediate levels of nutrient enrichment (primarily of phosphate). The high species number is probably stimulated by bottom-up effects of nutrient enrichment (51,54). Although we can assume that the ditches have been enriched for several years, the effects of long-term enrichment predicted by (54), namely the reduction in species numbers and the shift in community structure, were not observed in the ditch system studied when we compare it to the ponds.

However in contrast to results from (53), the nutrient pollution in ditches did not lead to more homogenous communities for the FFG predator in ditches compared to ponds, instead the opposite is the case. In ditches, the number of recorded families belonging to the group of predators is nearly twice as high compared to ponds. Therefore, functional redundancy for this feeding type can be assumed."

Rebuttal letter of PONE-D-25-03786, Trau et al., Assessing agricultural effects on benthic invertebrate communities in ponds and ditches using $\delta^{15}N$ and $\delta^{13}C$ isotope niches

356 is there a better term than indifferent? I think generalist could be more appropriate

I changed indifferent to the term omnivore.

357 meaning enriched values either due to high levels of omnivory or secondary predators.

The omnivore-feeding group acts mostly as predators in the pond 2. They formed two clusters. One was on the same level as the predators and the second cluster even higher. I changed the text to make that clear. "In pond 2 they were clearly mostly predatory with two clusters formed. One cluster formed by individuals with the highest $\delta^{15}N$ values in that water body and a second cluster with the $\delta^{15}N$ values on the same level as the FFG *predators* in that pond (S 4 Table)."

360 herbivorous feeding preference

Addressed

364 but you measured pollution. This is something you should be able to test.

True. I deleted the word probably.

368 why is this related to wider habitats?

Because a more diverse habitat leads to more diverse resources which leads to a more diverse diet and a more diverse isotopic signal, resulting in wider niches = larger SEA.c mode.

There was a sentence added in the methods section to make this connection clear for the reader.

"A larger SEA corresponds to a more diverse isotopic signal and thus to wider niches."

370 lower diversity of littoral microhabitats in ditches? You started your paragraph talking about ponds.

I made clear in the text that I mean more diverse habitat around the ponds than the ditches in this study. I changed the order of the next sentence to make clear that I am talking about lake ecosystems. This was the closest I could get to ponds in the body of scientific literature on that issue.

373 a well-documented effect of eutrophication is the development of cyanobacteria, that outcompete other primary producers, but are commonly toxic for animals, explaining why eutrophication could actually reduce grazers diversity.

Added in the text as follows: ". In eutrophic waters cyanobacteria blooms can occur which are toxic for other primary producers and can lower the number of *grazers*, but the concentration of cyanobacteria was high for one pond only (data not shown). This could be a measurement of a peak value that had already been exceeded in the other waters, as cyanobacterial blooms can occur very quickly."

374 it is not clear what do you mean by wider habitat? Is the width of the water body, or the number of microhabitats available?

Yes, the number of microhabitats is meant. Changed in the text.

374 higher taxa richness where? These results were not presented before

The sentence was changed to: "Additionally, the larger number of microhabitats and/or more resources used can explain commonly observed patterns of higher taxa richness and the associated possible different preferences in ponds (8-10)"

375 what is a taxa group?

The larger groups, such as Coleoptera, Diptera, Ephemeroptera etc. Taxa groups named in the text for clarity.

381 could pesticides have important direct effects on the invertebrates, leading to higher mortality rates, in addition to the indirect effects through primary production?

They could have direct effects and these direct effects can be measured with the TUsum for invertebrates. This value indicates the toxicity of the sample towards invertebrates, see also chapter 2.3.1. However the TU values for invertebrates were not above the critical threshold value of 3.27 (see Liess et al. 2021) in our data thus we do not expect high mortality rates from direct pesticide exposure. Nevertheless, the data is based on one measurement and peak concentrations can be missed.

386 it makes sense for me to have higher variability in these groups related to their diets; they are after all generalists

Yes, true for the omnivores. Text was changed as follows: "Nonetheless, the FFGs indifferent omnivore in ponds and collector/filterer in ditches show wide credible intervals that indicate a high degree of opportunism and variability in their feeding strategies which is consistent for the FFG omnivore. For the FFG collector/filterer and further sampling would be needed to predict modes with less uncertainty.

393 Why?

We reformulated this sentence as follows: "Although higher numbers of pesticides were found in ditches compared to ponds, this impact does not transport into the expected toxicity gradient for algae, invertebrates, and aquatic plants, presumably due to the application of low-toxicity pesticide products by the farmers. Additionally, (54) recently found patterns of synchronous toxicity levels towards algae and aquatic plants between water bodies with a comparable distance to each other like in our study. This should be accounted for in further studies, and future sampling between the months of April and June is generally recommended (54,55).

405 I am surprised to read here that ponds were supposed to be a reference community. This kind of statement should be mentioned very early in the Methods or Introduction. Moreover, I don't think ponds embedded in an agricultural landscape could be used as a reference, less impacted community by agriculture. I'd suggest using the water bodies as a gradient of agricultural effects, including nutrient enrichment, pesticides, and the proportion of surrounding crops to test your hypotheses.

Yes, the ponds are no control community in the real sense of a control. The part of the sentence was deleted. In the manuscript we are concluding that: "Agricultural activity and a homogenous environment did not lead to simplified structures in the ditches compared to ponds."

Your suggestion was added at the end of the introduction leading to the research questions.

Reviewer #2:

Manuscript:

Assessing agricultural effects on benthic invertebrate communities in ponds and ditches

using δ^{15}N and δ^{13}C isotope niches

Authors: Fee Nanett Trau, Kathrin Fisch, Stefan Lorenz

Rebuttal letter of PONE-D-25-03786, Trau et al., Assessing agricultural effects on benthic invertebrate communities in ponds and ditches using $\delta^{15}N$ and $\delta^{13}C$ isotope niches

Overall, the manuscript presents interesting and valuable research, but several key areas require further clarification to improve its structure, transparency and readability. The study's aim is currently too vague and should be made more specific, particularly in regard to which aspects of benthic invertebrates are being investigated. The introduction would benefit from a clearer distinction between ponds and ditches, specifying their purpose, shape, and typical formation or usage.

In the methods section, there is a lack of clarity surrounding the toxicity calculations mentioned. It is important to explicitly connect these methods to the study's objectives so the reader can follow the logic of your approach. At the moment, the description of toxicity tests, seem disconnected from the rest of the paper and need to be more clearly integrated into the study's goals. If toxicity testing was conducted, please describe the specific methods used, including whether test organisms or particular pesticides were involved. Further, the process for sorting organisms into taxonomic and functional feeding groups (FFGs) should be described more clearly. The use of boiling water as a method needs more explanation- if this method is employed instead of freeze-drying, please justify why it was chosen and explain how it might affect the isotopic analysis.

It would be valuable to emphasize whether the observed differences between ponds and ditches are statistically significant. Discussing the statistical significance of these differences would provide a clearer understanding of their reliability and help contextualize the results within the broader field. Additionally, providing information about the statistical tests used, the p-values, and any relevant confidence intervals could strengthen the interpretation of the findings and give readers more confidence in the conclusions drawn.

Some aspects of the figures and tables require additional detail. For example, Figure 1 should have a more informative caption, specifying what the red labels represent and distinguishing between the different symbols. Some Tables also need clearer explanations.

In addition, there are a number of minor issues that need addressing. It would be useful to include more details on the taxonomic composition of riparian vegetation, ideally at the family level. The citation format should be checked according to the authors' guidelines to ensure consistency. Also, please specify the number of replicates used at each location and for each taxonomic or functional feeding group, as well as the amount of material weighed for each replicate sample.

More specific comments and suggestions are provided in the attached PDF. Addressing these points will strengthen the manuscript, making it clearer, more precise, and easier for readers to follow.

Author's response:

We thank the Reviewer for the time that he/she invested in evaluating our manuscript and we hope that we have addressed all the concerns raised by the reviewer accordingly.

Comments reviewer 2 (black) with authors responses (green):

Abstract

17/18 The aim should be more detailed and clear. It is not clear which aspect of benthic invertebrates are you investigating?

Aspect added in the text. "… isotopic compositions of benthic invertebrates in ditch and pond communities.

Rebuttal letter of PONE-D-25-03786, Trau et al., Assessing agricultural effects on benthic invertebrate communities in ponds and ditches using $\delta^{15}N$ and $\delta^{13}C$ isotope niches

20 carbon and nitrogen stable isotope ratios

Addressed, changed to ratios.

28 what exactly regarding predators?

The large diversity in ditches compared to ponds, added in text.

30-32 this is very general conclusion - try to be more specific following your findings

More specific information added in the text as follows: "The impacts of individual structures within each water body are more pronounced than influences based on the type of water body. "

Introduction

46 I would recommend adding a sentence or two that highlights the key differences between a pond and a ditch, specifically regarding their purpose, shape, and typical formation or usage. This would help clarify the distinction between the two and provide a more comprehensive understanding for the reader..

The differences of ponds and ditches were highlighted with regards to the influence of agriculture they are prone to and the importance for benthic invertebrates in the introduction. Information on the formation and usage can be found in chapter 2.1.

64 Maybe it would be more precise to say "respiration mode" here.

Addressed

69-72 I suggest revising the sentence for clarity. The current wording implies that the principle of "you are what you eat (plus a few ‰)" makes stable isotope analysis possible, but it's actually the use of stable isotopes that has led to the conclusion that this principle applies.

A more accurate phrasing could be: "The principle of "you are what you eat (plus a few ‰)" (23) has led to the use of stable isotopes to analyze isotopic niches, food web structures, and species interactions in ecosystems (24)."

This revision would better reflect the relationship between stable isotopes and ecological analysis.

Addressed. Changed as suggested.

73 Consider to shape this part as follows: "...progressively increases at each trophic level."

Addressed. Changed as suggested.

76 , while

Addressed. Changed as suggested.

77 which exactly? (information on the carbon and nitrogen stable isotope ratios?)

Yes, info added for clarity.

78 Which community metrics? It would be helpful to clarify what metrics are being referred to (such as species diversity, trophic structure, or niche overlap?)

Metrics explained in the text as follows " …can be quantified and metrics that for example provide insights on the isotopic niche width or proxies for resources use can be derived,…"

Rebuttal letter of PONE-D-25-03786, Trau et al., Assessing agricultural effects on benthic invertebrate communities in ponds and ditches using $\delta^{15}N$ and $\delta^{13}C$ isotope niches

85 can you maybe provide an example more relevant for your (freshwater) study ?

We searched relevant literature to the topic of ecological disturbance and changes of niche overlaps and to the best of our knowledge, no such literature exists. We also made it more clear in the sentence that this is an example from a disturbed ecosystem. If you have literature suggestions we are very open to those.

85-87 Since this is not a review paper, I recommend removing this sentence. If relevant, you could summarize key points from these sources, but as it stands, it doesn't contribute significantly to the understanding of your study topic.

Removed as suggested.

88 I recommend avoiding the use of "we will" when stating the study aim. Instead, consider rephrasing it as "The aim of this study is/was to..." which maintains a more formal and objective tone

Addressed, as follows: "In this study the word niche will be used in the sense of the isotopic niche,…"

89 The goal stated like this lacks clarity regarding which specific ponds and ditches are being studied, as well as the geographical regions involved. It would be beneficial to specify the study locations or the regions of interest to provide context. Consider revising the sentence to include this information, such as: "...compare the isotopic niche widths of benthic macroinvertebrate groups and communities in ponds and ditches from [specific regions or habitats], using a geometric approach (22), to assess the impact of agricultural stressors, such as pesticide residues, on these niche metrics.

Throughout the review process, it was stated more clearly in the introduction what are the differences between ponds and ditches and why we are investigating, those water body types. For example the paragraph was changed in the text as follows: "We compared niche widths of FFGs and the pond and ditch communities based on a geometric approach originally developed by (25) to examine the isotopic niches of benthic invertebrates in ponds and ditches. The aim is to assess the influence of agricultural stressors (toxicity of water samples due to pesticide residues and eutrophication due to nutrient inputs) on the isotopic niche metrics.
We assume that ponds and ditches are affected differently by agricultural stressors, affecting habitat availability and quality and thus influencing benthic invertebrate food webs and niche size (33,34). Ditches are expected to be more strongly influenced by agricultural stressors than ponds due to their characteristics, which are a rather homogeneous habitat in and around the water body, a directly farmed environment with the location in the field (grassland) without a riparian buffer. Ditches, for example, are probably more contaminated, but as they are part of a flow system, pollutants can also be transported downstream. Ponds, on the other hand, are better protected from pesticide inputs, but can act as a source and sink for pesticide residues in the sediment (35). The water body type's ponds and ditches are used as gradients of agricultural effects, including nutrient enrichment, pesticides, and the proportion of surrounding crops to test our hypotheses."

90 please specify (taxonomic groups? or some other groups? communities of which organisms?)

…added in text. Meant are the functional feeding groups and the pond and ditch communities.

90 I think you should name authors here and then put (22) after their names (please refer to instructions for authors)

I checked the required reference style from the journal, again and they want the following: "References are listed at the end of the manuscript and numbered in the order that they appear in the text. In the text, cite the reference number in square brackets (e.g., "We used the techniques

developed by our colleagues [19] to analyze the data").”
https://journals.plos.org/plosone/s/submission-guidelines#loc-references

Therefore, I kept the style used in the manuscript.

92 In the methods section, you mention toxicity calculations, but it’s unclear whether this refers to the part you’re describing. Please ensure there is a clear connection between the stated goals and the methods, so that the reader can easily follow the logic of your approach.

Yes, added explanation in the brackets to make the clear connection as suggested “…(e.g. toxicity of water samples due to pesticide residues)…”

92 You also measure other physico-chemical parameters. Are you investigating their influence as well?

Yes, they are used as variables for the dbRDA analysis. They were referred to as environmental or physicochemical parameters in the manuscript. Changed it to environmental parameters throughout the script.

94 in these specific habitats or region (where the study was conducted)?

Addressed. Added for clarity as suggested.

94 patterns of what exactly?

“…patterns of isotopic niches of FFGs…”, added for clarity.

Methods and data

Fig. 1 I was unable to find figure captions that provide sufficient context to help me understand the figures. In the caption and legend of Fig. 1, it would be helpful to specify what the red label represents for the regions and what it represents for the ditches. Additionally, I recommend splitting Fig. 1 into Fig. 1A and Fig. 1B for clarity. Also, could you clarify the difference between the pink circles (representing ponds) and the blue symbols (indicating pond locations or selection)?

Addressed. Figure 1 revised and simplified. As well as additional information in caption added.

104 Is the irrigation main purpose of these ditches?

No, it was mainly drainage of the area to utilize the wetlands for agricultural production. The function of ditches is mentioned in the sentence before. It is drainage.

114 It would be helpful to include more details on the taxonomic composition of the riparian vegetation, at least at the family level, to provide a clearer understanding of its structure.

Information added for reed and grasses. For the trees and shrubs the information was not protocoled. However for the influence of agricultural stressors the vertical composition of the riparian zone is important that’s why we only protocol if there were shrubs and trees present or not.

Table 1 Please improve the caption for Table 1 by providing clear explanations of the terms 'habitat' and 'agricultural cultivation,' as the meaning of 'agricultural cultivation' is unclear. Additionally, it is not described in the text or the table caption how the percentage of the listed riparian vegetation types was determined. Is this percentage based on the surrounding surface area or a specific zone around the sites?

Rebuttal letter of PONE-D-25-03786, Trau et al., Assessing agricultural effects on benthic invertebrate communities in ponds and ditches using δ^{15}N and δ^{13}C isotope niches

Addressed. Caption Text added: "Habitats were described for the water bodies, when present in the wadeable area of the shore length with more than 5 %. The riparian zone is defined as the area from the edge of the field including the edge strip to the waterline, on this area the growth of grasses etc. was estimated as a percentage."

Table 1, table header, column 4-8 How did you assess the % of individual riparian vegetation type?

The percentages are estimated in the field. Information added in caption.

Table 1, table header, column 2 This refers to sampled aquatic (micro)habitats or something else?

Yes, exactly. See above. Information added in caption.

123 I know that now it is too late, but it would be good to also have analysis of the primary food sources (plant material, periphyton, etc.).

Initially it was planned to sample primary food source in parallel to the consumers. However, we were not able to discriminate isotopic signals from the food sources from each other and thus assume incorrect sampling of these sources to bias our analysis. Therefore, we were not able to incorporate correct source data in the analysis to create e.g. mixing models. Additionally, we should have sampled the food sources a few weeks earlier and also repeatedly before sampling the consumers to account for metabolisation of sources into consumer tissue. However, I am open for further discussions on the dataset and opportunities for further analysis. Please feel free to contact me.

126/127 Was the sorting of organisms done by order/family or by functional feeding groups? This is unclear. Did you first sort them into taxonomic groups and then assign them to functional feeding groups afterward? Also, which keys or resources did you use for sorting the organisms into taxonomic and functional feeding groups?

Organisms were sampled in the field based on orders/families. Then determined to family or genus where possible and then assigned into functional feeding groups based on the freshwater ecology database (Schmidt-Kloiber, Astrid; Hering, Daniel (2015): www.freshwaterecology.info – An online tool that unifies, standardises and codifies more than 20,000 European freshwater organisms and their ecological preferences. In: Ecological Indicators 53, S. 271–282. DOI: 10.1016/j.ecolind.2015.02.007.) Information added in the paragraph.

129 Could you provide a reference for this step? It's unclear why this procedure was used. Did you freeze-dry the organisms instead of pouring boiling water over them? Please clarify and explain the rationale behind this choice.

The method was used to kill the individuals as quickly and humanely as possible. Boiling water was used to avoid altering the isotopic signals using ethanol. Afterwards individuals were dried in a drying chamber. There is no literature available that compares freeze drying and boiling but accordingly to the following literature we do not expect any significant effects.

- Ricardo Fernandes, John Meadows, Alexander Dreves, Marie-Josée Nadeau, Pieter Grootes, A preliminary study on the influence of cooking on the C and N isotopic composition of multiple organic fractions of fish (mackerel and haddock), Journal of Archaeological Science, Volume 50, 2014, Pages 153-159, ISSN 0305-4403, https://doi.org/10.1016/j.jas.2014.07.006.
- Zhou J, Guo B, Wei Y, Zhang G, Wei S, Ma Y. The effect of different cooking processes on stable C, N, and H isotopic compositions of beef. Food Chem. 2015 Sep 1;182:23-6. doi: 10.1016/j.foodchem.2015.02.116

Rebuttal letter of PONE-D-25-03786, Trau et al., Assessing agricultural effects on benthic invertebrate communities in ponds and ditches using $\delta^{15}N$ and $\delta^{13}C$ isotope niches

129 If boiling water is used, it would be important to explain why in the context of the study, as it may not be the most optimal method for preserving isotopic integrity.

Please see comment above.

Samples were afterwards preserved in the freezer until further processing. Information added in the text.

129/130 In the second sentence above, you mention that this step was carried out on-site. Please verify this and provide a detailed description of the procedure you followed.

Individuals were further determined in the lab. To family where only the order was assigned out on-site and to genus where possible. Added in the text for clarity.

133 Which groups of organisms were treated as individuals in your analysis?

This information follows in the sentence afterwards. It depends on the weight of one organism. If the sample weight was above 2 mg, one organism was analysed. If the sample weight was below 2mg, several organisms had to be pooled for analysis. Sentence clarified in the text.

137 How many replicates did you have for each location/site and for each taxonomic or functional feeding group? It is still unclear whether you pooled all individuals from the same taxonomic group or all individuals belonging to the same functional feeding group (FFG). Additionally, how much material was weighed for each replicate sample, and what was the weight of the individual samples (i.e., how much ground material did you use per replicate)?

See above. Individuals were only pooled if the individual was below the optimum sample weight of 2mg. They were pooled only to one sample when they are from the same family. The raw data, sample weights and quality control values can be found in the isobank repository. Added in the text for clarity.

140/141 insert reference to IsoBank repository,

Yes, I will insert it in the process. Submission is still in progress. Please let me know if you want to receive the table with the raw data before. I can provide it through the editors.

143 relative to?

Addressed.

166 Please check the authors' instructions to clarify the citation format. Should you list the authors and year of publication followed by the reference number in parentheses, or should it be formatted with only the reference number in parentheses?

I checked the required reference style from the journal, again and they want the following: "References are listed at the end of the manuscript and numbered in the order that they appear in the text. In the text, cite the reference number in square brackets (e.g., "We used the techniques developed by our colleagues [19] to analyze the data")."
https://journals.plos.org/plosone/s/submission-guidelines#loc-references

Therefore I kept the style used in the manuscript.

147 deleted "Taken from"

Addressed

Rebuttal letter of PONE-D-25-03786, Trau et al., Assessing agricultural effects on benthic invertebrate communities in ponds and ditches using $\delta^{15}N$ and $\delta^{13}C$ isotope niches

172 It is unclear to me which study goal this part corresponds to. Additionally, the method description does not provide enough detail on what was actually done. Did you test the toxicity of the sampled water using test organisms, or did you specifically test certain pesticides? This section seems disconnected from the main context of the paper.

This point was also mentioned earlier in the introduction. To clearly connect the parts it was stated in the introduction as follows: "…(e.g. toxicity of water samples due to pesticide residues)…". Also see your comment above. L. 92. Furthermore I added the following sentence at the beginning of the paragraph: "To calculate the toxicity of the analysed water samples based on measured pesticide residue concentrations in the water samples for invertebrates, macrophytes and algae the toxic pressure…"

Additionally I clarified in the text that the EC50 and LC50 values were taken from the databases mentioned.

177 sp. should not be italicized

Addressed.

187 I don't see taxonomic orders in Table 2 - there are FFGs only. Please provide missing information or change descriptions of the procedures and corresponding datasets.

Yes true, description changed. The information on the orders can only be found in the supporting material 5. Stated in the text now.

Table 2 The Table caption should be more informative.

Addressed. Change as follows: "Number of samples available and used for analysis for each water body and the FFGs."

Table 2 Can you somewhere explain which organisms belong to this FFG? What is the feeding strategy of this group?

Yes, the following paragraph was added:

"Classified as collector/filterer are Bivalvia and the family Culicidae Gen. sp., which filter suspended FPOM from the water column (32). The families of Ephemeroptera and some Dipterans belong to the group collector/gatherers, which feed on sedimented FPOM. Grazers/scrapers graze on surfaces where they feed on algae tissue or biofilm, also they feed on algae or plant tissue (32). In the present dataset those are mostly Gastropods and families belonging to the group are for example Lymnaeidae Gen. sp. or Planorbidae Gen. sp.. Within the group indifferent orders/families are gathered that could not be assigned to one distinct group. In the dataset Chironomidae Gen. sp., Corixidae Gen. sp., Haliplidae Gen. sp. and Stratiomyidae Gen. sp. were assigned to the group. Predators hunt on prey and examples are Dytiscidae Gen. sp., Ceratopogonidae Gen. sp. or Coenagrionidae Gen. sp.. Typical shredders are Gammaridae Gen. sp. or Asselidae Gen. sp., which feed on plant tissue, fallen leaves or CPOM (32). A detailed list of the families belonging to the FFGs can be found in the table supporting Information 5."

Table 3 These are not listed in the table? If you have measured them, please add a description in the Methods part and the values in this table.

The table was very wide and the columns were probably not visible for you. But I can delete them here as the information on habitats and riparian zone is already given in table 1.

Rebuttal letter of PONE-D-25-03786, Trau et al., Assessing agricultural effects on benthic invertebrate communities in ponds and ditches using $\delta^{15}N$ and $\delta^{13}C$ isotope niches

Table 3 There is no description of the methods used to determine these listed parameters. Please provide this information in the Methods section.

Column reed in % deleted, as the information is already given in table 1. Columns with information on algae concentrations deleted as they are not used in the further analysis of the data.

200 – 202 This belongs to the Results in my opinion.

The section is part of the methods chapter as it is the basis for further data processing.

207/208 This is more appropriate to shift to the Results section.

The section is part of the methods chapter as it is the basis for further data processing.

223 what does this mean?

Functional feeding group indifferent is explained above. The new paragraph in your comment regarding table 2.

237/238 This was already explained above, so you can remove this.

Deleted in the chapters above. 2.5.1. and kept here where the analysis are explained.

Results

Fig. 4, caption Which one (Pond 3)?

Yes, stated in the text.

270 What do you mean by this term (probable mode)?

Changed to posterior.

271 I guess that in this context the expression "probable mode" refers to the most likely or typical value or range within a distribution of δ13C values. It seems to imply the central or most likely range of δ13C values for the ponds.

Yes, exactly. The mode is the value most often appearing in the data. Probable might be confusing. I changed it to posterior, as the mode is calculated via Bayesian statistics.

So, the range of δ13C values in ponds is likely broader (or more variable) compared to ditches? A clearer phrasing might be:

"The typical range of δ13C values appears to be larger in ponds."

Addressed. Text changed as follows: The typical range of $\delta^{13}C$ values is distinct in the two water body types and larger in ponds (Fig. 5 b).

271 have you tested for significant differences? Are these differences statistically significant?

Yes, added the p-values for the T-test in Figure 5 and added “statistically significant” in the text.

273 Are these differences significantly larger?

Yes, added the p-values for the T-test in Figure 5 and added “statistically significant” in the text.

Fig. 5, caption FFG or taxonomic groups?

Rebuttal letter of PONE-D-25-03786, Trau et al., Assessing agricultural effects on benthic invertebrate communities in ponds and ditches using $\delta^{15}N$ and $\delta^{13}C$ isotope niches

The grouping is based on the combination of the water body and the functional feeding group, resulting in 37 groups. Made more clearly in caption.

280/281 This should be somehow merged with the first two sentences after the Fig. 4 caption above.

To be honest I already said the same just before the Fig. caption 4. So I completely deleted the sentence in Lines 280/281.

290 Does this mean that you have also analyzed benthic density at each location? If yes, please describe it in the Methods part.

No, we did not. This part was confusing. I deleted the abundances.

307 space needed (check throughout the text)

Changed the structure of stating mean and standard deviation as suggested by another reviewer to this format: “(M = 2.7 µg/L, SD = 1.5 µg/L)”

313/314 this is more appropriate for the Discussion

Incorporated in discussion as suggested.

314 – 318 This is more appropriate for the Discussion.

Addressed. I changed the sentences in the results part to be not interpretative, as follows: “The isotopic composition of benthic invertebrates and the resulting Layman metrics of pond 3 and 2 are influenced by the toxicity of water samples for invertebrates (TUsum_invert). Pond 1 and the ditches, except ditch 2, are influenced by the toxicity of water samples for algae (TUsum_algae), the concentration of nutrients (nutrients_sum), and the number of pesticides found (N_pesticides).

The result will be discussed later in the discussion.

Fig. 7, caption The caption needs to provide more information. Specifically, what do the arrows represent? The figure and its caption should be self-explanatory, allowing the reader to understand the content without needing to refer to the Results section.

Addressed. Information added in caption.

Discussion

323 Can you also discuss the influence of your used methodology on the obtained results (using the boiling water instead of dry-freezing the organisms).

As stated in our comment above there is no literature existent comparing the methods. Experiments where isotopic composition was measured after cooking process resulted in no significant change thus we do not expect any difference of the isotopic signal by using boiling water.

Therefore, no discussion on this aspect of the methodology could be provided.

334 reference for this statement is needed

Addressed. Reference added.

337 reference needed for this conclusion

Addressed. Reference added.

340 – 343 references needed to support these claims

Rebuttal letter of PONE-D-25-03786, Trau et al., Assessing agricultural effects on benthic invertebrate communities in ponds and ditches using δ¹⁵N and δ¹³C isotope niches

References added in the manuscript.

344 space missing

Addressed.

365 Cloeon italicized, sp. not italicized

Addressed.

374/375 reference needed

References added.

383/384 How did you get to this conclusion?

The paragraph is referring to the configuration of the dbRDA, which is showing the influence of the environmental parameters based on the Layman metrics for the water bodies. An influence on the primary producer level and thus bottom-up effects are expected for the ditches and pond one as the variables explaining those best are all connected to primary production.

387 can you elaborate why there are no collector/filterers in ponds, but they are present in ditches? (probably due to hydrological conditions, but would be worth of discussing)

The FFG collector/filterer was sampled with below three individuals in ponds thus the data was not used in the analysis. Also in ditches their occurrence was low (10 Bivalvia, 2 Dipterans, see S5 Table). It is only possible to speculate about the reasons, but potential factors could be: i) time of sampling, low occurrence of collector/filterer in November as their food base is low, ii) hydrological conditions and sedimentation in ponds which are not favourable for e.g. Bivalvia. As collector/filterers were present in low numbers in both ponds and ditches (only in the ponds excluded from the analysis), we did not discuss this further.

397 – 399 This text part is more apropriate for the Results section

Addressed.

Supporting information

545 this should go after "Active substances! and measured should go after parentheses

Addressed.

555 Genus names should be written in italic

Addressed.

Figures

Fig. 1 I suggest to add labels for Ditch 1, Ditch 2, Ditch 3, Ditch 4 as well as for Pond 1, Pond 2 and Pond 3, so it would be easier to follow Figs 2 and 3 and Table 1.

Due to contractual agreements, the locations may not be assigned by name.

Fig 2 It would be helpful to link these photos with the labels in Figure 1. Please add labels for 'Ponds' and 'Ditches' in Fig. 1, corresponding to the labels in Table 1 and those used in Figs. 2 and 3, for better clarity and consistency.

Due to contractual agreements, the locations may not be assigned by name.

Rebuttal letter of PONE-D-25-03786, Trau et al., Assessing agricultural effects on benthic invertebrate communities in ponds and ditches using $\delta^{15}N$ and $\delta^{13}C$ isotope niches

Fig 6 I recommend using the same order of FFGs along the x-axis for both the red and blue categories (e.g., predator, shredder, collector/gatherer, collector/filterer, grazer/scraper, indifferent). If certain FFGs are absent, such as collector/filterers in the red category, it would be helpful to leave blanks. This way, it will be clear that these FFGs are not present in ponds.

Addressed. Figure changed for the same order of FFGs for the water body types and a NA for FFG collector/filterer in the case of ponds.
