# Supplementary material for: Assessing agricultural effects on benthic invertebrate communities in ponds and ditches using δ¹⁵N and δ¹³C isotope niches
Source: PLoS One. 2025 Nov 24;20(11):e0336486. doi: 10.1371/journal.pone.0336486 (PMC12643296; doi:10.1371/journal.pone.0336486)
Supplement: Attachment — Submitted filename: Response to Reviewers_v2.docx [file pone.0336486.s012.docx]

**Author’s response:**

We thank the Academic Editor for handling our manuscript and for the careful selection of qualified Reviewers. We are glad to receive such a constructive, detailed, and fair review report. We revised our manuscript again according to the comments of the Reviewers. We hope to address all raised questions and issues made by the reviewers. Please go through the following comments and authors response.

**Comments to the Author**

1. If the authors have adequately addressed your comments raised in a previous round of review and you feel that this manuscript is now acceptable for publication, you may indicate that here to bypass the “Comments to the Author” section, enter your conflict of interest statement in the “Confidential to Editor” section, and submit your "Accept" recommendation.

Reviewer #1: (No Response)

Reviewer #2: (No Response)

2. Is the manuscript technically sound, and do the data support the conclusions?

The manuscript must describe a technically sound piece of scientific research with data that supports the conclusions. Experiments must have been conducted rigorously, with appropriate controls, replication, and sample sizes. The conclusions must be drawn appropriately based on the data presented.

Reviewer #1: Partly

Reviewer #2: Yes

3. Has the statistical analysis been performed appropriately and rigorously?

Reviewer #1: No

Reviewer #2: Yes

4. Have the authors made all data underlying the findings in their manuscript fully available?

The [PLOS Data policy](http://track.editorialmanager.com/CL0/http:%2F%2Fwww.plosone.org%2Fstatic%2Fpolicies.action%23sharing/1/010f019719162fd9-2e4e4ad1-3503-4d79-b68d-01b53593cfe1-000000/lCTWwONEFpnbOIrLp-aM794nJlK1F1QVjQWLyDI9fJA=213) requires authors to make all data underlying the findings described in their manuscript fully available without restriction, with rare exception (please refer to the Data Availability Statement in the manuscript PDF file). The data should be provided as part of the manuscript or its supporting information, or deposited to a public repository. For example, in addition to summary statistics, the data points behind means, medians and variance measures should be available. If there are restrictions on publicly sharing data—e.g. participant privacy or use of data from a third party—those must be specified.

Reviewer #1: Yes

Reviewer #2: Yes

5. Is the manuscript presented in an intelligible fashion and written in standard English?

PLOS ONE does not copyedit accepted manuscripts, so the language in submitted articles must be clear, correct, and unambiguous. Any typographical or grammatical errors should be corrected at revision, so please note any specific errors here.

Reviewer #1: No

Reviewer #2: Yes

7. PLOS authors have the option to publish the peer review history of their article ([what does this mean?](https://track.editorialmanager.com/CL0/https:%2F%2Fjournals.plos.org%2Fplosone%2Fs%2Feditorial-and-peer-review-process%23loc-peer-review-history/1/010f019719162fd9-2e4e4ad1-3503-4d79-b68d-01b53593cfe1-000000/3Nk51pLtW-dSzHwCvMmHcuc2HxdasmB0smgFhv5QH84=213)). If published, this will include your full peer review and any attached files.


If you choose “no”, your identity will remain anonymous but your review may still be made public.


**Do you want your identity to be public for this peer review?** For information about this choice, including consent withdrawal, please see our [Privacy Policy](https://track.editorialmanager.com/CL0/https:%2F%2Fwww.plos.org%2Fprivacy-policy/1/010f019719162fd9-2e4e4ad1-3503-4d79-b68d-01b53593cfe1-000000/NrRFWkltFSVKti-Qzti2peVK9NYRLXkH08HlISYR0ZI=213).

Reviewer #1: **Yes:** Daniela Cortes

Reviewer #2: No

# Reviewer #1

Review Comments to the Author

The manuscript has improved. The authors addressed some of the concerns; however, considerable work is still needed in order to have a manuscript ready for publication. It seems to me that some changes in the manuscript were made to please the reviewers, but without giving deeper thought about why these requests were made. In that sense, changes lack attention to detail. For example, Table 3 was moved to results, as suggested, but it is not described, and Figure 4 is missing. Furthermore, I disagree with the interpretation of results the authors made. They found statistical differences in several metrics yet concluded that there are no clear patterns.
I have several major and minor comments across all sections that I detailed here:

**Author’s response:**

We thank the reviewer for the time that she invested in evaluating our manuscript again and we hope that we have addressed all the concerns raised by the reviewer now.

**Comments reviewer 1 (black) with authors responses (green):**

Abstract
15: the last part of the sentence, after landscape is a bit redundant and unnecessary; it could be removed.

Addressed, part removed.

15-20 I still find the wording here difficult to justify the use of functional approaches (or not use of taxonomic ones). Might I suggest a phrasing like: Biodiversity metrics based on taxonomy, such as richness or evenness indices, might fail to describe changes in ecosystem functions that result from the effects of agriculture. In contrast, functional approaches, such as the use of functional feeding groups and isotopic composition, better describe the use of resources by the invertebrates, improving our understanding of the influence of agricultural stressors on aquatic communities (just a suggestion, please use your own words)

Addressed as follows: “Due to a lack of sensitivity biodiversity metrics based on taxonomy only, such as overall taxa number, Shannon or evenness index, might fail to detect changes in communities, which lead to changes in ecosystem functions resulting from agricultural practices. In contrast, functional approaches, such as the use of functional feeding groups and isotopic composition, better describe the utilisation of resources by invertebrates and improve our understanding of the impact of agricultural stressors on benthic invertebrate communities in ponds and ditches.”

29 we still don’t know why these differences are unexpected. I suggest removing that word.

Addressed. “unexpected” was deleted

33-34 this seems to be part of your results, rather than a general statement. I would write it in past tense. More importantly, this might be, in effect, a consequence of your small number of water bodies. Therefore, I don’t think your sentence at line 31 is justified: communities respond in complex ways to stressors… since this is, again, an effect of the sample size. I suggest re-ordering this last section of the abstract to provide more accurate conclusions. For example: The strong fluctuations in biotic and abiotic parameters resulted in pronounced differences among water bodies, but no clear patterns between water body types. However, larger datasets… However, after reading your main text, I disagree with this conclusion.
Statement written in past tense and text changed as follows: „The strong fluctuations in biotic and abiotic parameters led to pronounced differences between the water bodies. Our study showed that benthic invertebrate communities in small unstable systems respond in complex ways to stressors.

## Introduction

51 water bodies. Suggested phrasing: Although these small water bodies are usually not included in monitoring programs (5,7), their importance for benthic macroinvertebrate biodiversity and ecosystem services is increasingly gaining recognition in research activities (5,8–13).

Changed as suggested.

53-56 These last sentences are a bit repetitive. It was already stated that these ecosystems are valuable habitats for invertebrates. Moreover, the sentence structure is a bit confusing. I think mentioning the orders at the end of the sentence is unnecessary since, as I have suggested before, these are abundant and common groups in many aquatic ecosystems. My previous comment was not meant to have an aesthetic change in the wording, but to justify the inclusion of such a sentence in your introduction. I recommend closing the paragraph with a statement highlighting the knowledge gap that the manuscript is addressing.
Addresses, repetitive sentences deleted. Paragraph connected with the first sentences of the following paragraph and statement highlighting knowledge gap added as follows: “…This study will contribute to gain knowledge on measuring changes in communities, which is indispensable in order to conserve and promote these aquatic habitats and their biodiversity. Thorough knowledge on the status and indicators for measuring biodiversity is needed.”

58-59 I still disagree on that it is challenging being one of the most common bioindicators. I think you could instead jump directly into the previous findings and avoid this sentence.
Addressed. Deleted as suggested.

62-63 long-term persistent stressors would be likely reflected in taxonomic diversity or EPT, whereas short stressor gradients might result in stochasticity, for which functional approaches consistently reflecting habitat types and resources might be a solution
I partly agree. Long-term persistent stressors can be reflected in taxonomic diversity and EPT only when taxonomic reference data exists. Usually there is no historical data available from communities that were not exposed to pesticides etc.

69 the acronym FFG has not been defined in the introduction. Please do so
Addressed.

69-71 this sentence does not belong here since you haven’t told us yet anything about the water bodies under study. The justification for the use of FFG, from an ecological point of view, is your next sentence.
Agreed. Sentence deleted. The sentence that follows in the manuscript is the ecological explanation.

73 explain why they reflect the effects of agriculture. Is it because of the change in quality, quantity, type of feeding resources?

All the changes you mentioned are important and can be reflected. Made two sentences out of the part and revised the second sentence as follows: “Such analyses also provide valuable insights into the impacts of agricultural activity, including habitat fragmentation, degradation, and pollution, which alter resource quality, quantity, and type, ultimately influencing community structure (17,22–24).

74-78 this definition of niche could be better described in terms of the feeding/trophic niche, given that it is the topic of the manuscript and to better connect with the previous section of the paragraph.

Sentence to better connect the two paragraphs added: “Understanding how species interact with their environment and with each other is therefore essential for interpreting changes in community structure. In this context, the niche concept provides a framework for linking species traits and resource use with ecosystems.”

Further explanation and descriptions of the isotopic niche follow in the paragraph later. I started with the general niche concept and then went into stable isotopes and their meaning and then to the term isotopic niche. Otherwise the reader does not know what I mean with isotopic I think.

91 please define realized ecological niche
Addressed as follows: “The isotopic niche can thus serve as a proxy for the realized ecological niche of a species (portion of the ecological niche that a species occupies in nature, after accounting for competition and predation (31)) reflecting their use of resources and habitat (32). The realised niches are linked to land use and agricultural stressors, which alter resource availability and habitat quality.”

92 previous sentence was spelled realized... please, confirm with the journal British or American spelling
Under the term language they just state “Manuscripts must be submitted in English.” (<https://journals.plos.org/plosone/s/submission-guidelines>). I changed it to the American English spelling.

93-94 not necessary if the niche is explained in that context in the previous sentences
I would rather keep it to make it clear which niche term I am using to the reader. Especially as I am talking about the realized niche and the isotopic niche in the sentence before.

95-98 this paragraph could be written more concisely. Suggestion: We aimed to assess the influence of agricultural stressors, measured as water toxicity from pesticide residues and eutrophication, on the isotopic niche of FFGs in pond and ditch communities.
I followed your suggestion and kept the last part of my sentence to mention also the method: “We aimed to assess the influence of agricultural stressors, measured as water toxicity from pesticide residues and eutrophication, on the isotopic niche of FFGs in pond and ditch communities, using a geometric approach originally developed by (24).”

106-108 this phrasing is confusing. In my understanding, is not the body type, pond or ditch, what is used as a gradient (since this a categorical factor with only two levels; if so, which is higher and which lower?). It is the continuous values of nutrient enrichment, pesticides, and the proportion of surrounding crops.
I rephrased the sentence as follows: “Ponds and ditches representing water body types with varying exposure to agricultural effects (nutrient enrichment, pesticides, and the proportion of surrounding crops) were used to test our hypotheses.”

111 I am not sure I understand your second question. Do you mean that, on one side, you expect differing effect of agriculture between ditches and ponds on the food web structure (Q1 and H1), but similarities in the isotopic niches of specific FFGs (Q2 and H?)?

For your explanation. Question one is asking if we can depict the effects of agriculture on benthic invertebrate communities with the isotopic niches, can we see a gradient for example. In the text: “Can the isotopic niches depict the effects of agriculture on benthic invertebrate communities in the selected water bodies in the study region?”

Question two is asking if we do see the same image/pattern in ponds and ditches. In the text: “Are the observed patterns of isotopic niches of FFGs comparable in ponds and ditches for the FFGs?”

114 ... and presence of generalists compared to ponds. To keep parallelism and include the ponds in your hypothesis.

Added as suggested: “On the other hand, we hypothesize that trophic structures in ponds are more differentiated and that we observe more specialized groups with less niche overlap.”

## Methods

131 and ditch 4?
Addressed. It should be ditches 2 to 4.

149 specimens
Addressed.

150 otherwise? You collected as many as possible or discarded samples that did not reach 10 individuals?
Some more explanation was added in the text as follows: “Sampling was carried out until 10 specimens of the present orders/families were collected or as enough specimens were collected to create 10 pooled samples with the necessary sample weight as recommended by (30), whenever possible. If 10 specimen were not found a minimum of three samples was used for the analysis.”

159, 161 grounded
Addressed.

163 10-15 samples, even if they belonged to different genera?

No, samples were analysed for identified taxa separated. Identification was on family or genus level, if more than one genus was identified they were not mixed. Text changed as follows: “For each taxon identified, a maximum of 10 to 15 samples was analyzed.”

164 isotopic... were carried
Addressed.

177 that can explain... I still don’t understand this. Why do you expect that conductivity and pH explain the food web structure? Your answer was unfortunately not sufficient. I know these are standard variables typically measured during sampling, but that does not justify their inclusion in your statistical analysis. I could understand that you included them to control for intrinsic differences among water bodies; therefore, they are not explanatory but control variables.
The variables can explain differences between the water body types. Thus I would not name them control variables. Rephrased: “Environmental chemical parameters were measured to identify potential variables that could function to explain differences between the water body types.”

253 explain why these metrics were calculated, or what information did you get from them.
The explanation follows in the next paragraph. To make this clearer I added at the beginning of the paragraph “layman” before metrics in the text and deleted the word “described”.

269 check parallelism of the sentence
Rephrased to avoid parallelism and make sentences easier to folllow: “The SEA.c, based on SEA.B and adjusted for small sample sizes, provides and unbiased measure of isotopic niche width. (30). Larger SEA values indicate more diverse isotopic signals and broader niches. Corrected δ15N and δ13C values were used to compare FFGs via SEA.c.”

289 why do you have small samples sizes in EC and oxygen if they were measured with a probe?
We only measured one water sample parallel to the biological sampling. There was no probe installed taking multiple samples. I added “…once during sampling..” in the methods section to make this clear.

## Results

293 You have to describe the table, not just reference it. For example, what common patterns did you observe across ponds, and in comparison to ditches? Which type of water body tended to have higher nutrient concentrations, toxicity, or pesticides?
Table 3 moved to chapter 3.3. Here the table values are described.

Table 3. For the sake of completeness, describe all acronyms. Sample was used before to describe the individuals analyzed for isotopic composition. I recommend using here Site or water body. C° are the units of temperature. To keep it consistent with your other columns, use T [°C] or something similar.

Column heading for temperature changed. Column heading changed from sample to site in the table.

Fig 4 is missing. Is it maybe Fig S2? If so, could you show the biplot after correction? Maybe this will distinguish

Unfortunately, the figure got lost in the revision uploading process. This is the missing figure:

Fig 4: Biplots for the macroinvertebrate data. The left plot is corrected for δ^15^N influence of organic fertilizers on ditches. The right plot is corrected for δ^15^N as well as δ^13^C values for the influence of C4-plant maize on pond 3. Ellipses contain 40% of the data.

313 dC13 range is 5d not b
Addressed.

316 whereas
Addressed.

324 is this Figure S3? What different information is provided between Fig 5 and S3? It seems that many of the differences shown in Fig 5 disappeared in S3. This should be important to explain. In a more general comment, all tables and figures in the text are meant to be described and explained, not just mentioned.
In the figure in the supplements (S3) additionally the δ13C values are corrected for the influence of maize in pond 3. See figure description and text “For an illustration of the Layman.B metrics calculated **for both stable isotopes corrected**, see also figure S3.” therefore, the differences in δ13C values disappear and the TA and mean distance to centroid are not pronounced any more. Appendix 3 is only intended as an additional visualisation of what the values would look like without the influence of maize. S3 was included for the sake of completeness, as the correction is mentioned in the manuscript.

326 since Fig. 6 is comparing FFG among wb types, I suggest placing each FFG in pond and ditches next to each other, and distinguish them with colors, as you have done. This way is easier for the reader to notice the differences between, e.g., predators between water body types. You also need less labels in the x axis this way.
Changes to the plot were also requested by reviewer 2. These comments were used as a guide to enhance the figure.

327 I disagree with your initial statement. I think there are differences between ponds and ditches, as you mentioned in the following sentences.
Yes, addressed as follows: “ There are observable differences for the SEA.B modes and confidence intervals between FFGs when comparing ponds and ditches. “

332 what about the diversity of the other groups? Also, you didn’t really quantify abundance, since you stopped gathering individuals when you collected 10.

Quantifying the abundance was not the scope of the study. Text changed as follows: “Predators were in general observed in larger numbers and more diverse in ditches. Six taxa groups and 13 families and were recorded there, while five taxa groups and eight families were recorded in ponds (S5 Table). “

Predators are named here as an example as there were most families compared to other groups recorded, as stated in the following sentence.

Population mean changed to sample mean in line 332.

342 for all FFG, overlap was greater when corrected for both C and N. Moreover, why this comparison between corrections is important? Is one correction providing different information than the other? Why not simply correct by both and compare overlaps?
Both correction approaches are shown as the correction for δ15N only, removes the influence of the fertilization in ditches. The aim was to compare the FFGs for differences or similarities resulting from stressors nutrients and/or toxicity without overriding these effects by the strong fertilization effect (allow for comparability).

The correction for both effects (fertilization and maize in one pond) is shown for the sake of completeness in the table. It shows how the overlaps would look like if there was no fertilization effect and no maize effect. Also to see differences or similarities resulting from stressors nutrients and/or toxicity.

We added a sentence in chapter 2.5.3 to make clear why not both corrections are used for layman calculations: “Maize cultivation was not a general phenomenon like fertilization in the study area, the original δ13C values were kept for the calculations. In other words, the real C-source should not be artificially changed because of the niche width, but the N-source had to be scaled to the same level for comparability.”

348 a lower ... in ponds compared to ditches, and higher... in ditches...
Addressed.

351 higher
Addressed.

354 this is the description of table 3 that should be with the table, not in a different section.

Agreed. Table 3 moved to chapter 3.3.

## Discussion

366 what exactly do you mean by comparable? The isotopic niche width? However, you found marked differences in Fig 5

We meant that the two water body types can be compared between each other. Changed in the text as follows: “After the correction of δ^15^N for the influence of organic fertilizer, which was clearly visible in consumers in ditches, we were able to comparatively assess differences in the two water body types.”

369 I don’t agree with this interpretation. Again, you found statistical differences in some niche metrics and in some FFGs.
Addressed. Text slightly changed as follows: “However, within the agricultural affected sites studied here, isotopic niches could not depict benthic invertebrate community changes due to the agricultural stressors of nutrient input and pesticides. Exceptions are the general impacts on basal C and N resources (e.g. by fertilization or maize/C4 cropping), which were observed in the isotope niches.”

376 since the isotopic niche is indirectly measuring feeding resources, I’d recommend sticking to that word and not use it together with habitats.
Changed in the text as follows: “. The finding that the likely distribution of total niche width and distances between the individuals is greater in pond communities than in ditch communities indicates that resource diversity (that may be driven by the amount or presence of different habitats) is present in ponds, which lead to more extreme isotope ratios (32).”

381 what patterns did you expect to find? Since your main message seems to be that there were no differences among habitats, despite several metrics in your results showing differences, I wonder which were the patterns you expected to observe?

To make the message clear and avoid confusion, the first part of the Paragraph was deleted and we start the paragraph with “Contrary to our expectations…”

382 however, in your introduction you stated that ditches could also harbour high diversity. Therefore, why is this contrary to your expectations?
Ditches are also known to harbour high diversities but still we would expect ponds to be more diverse compared to ditches. This is why it was contrary to our expectations. The introduction was adapted based on a reviewer comment. The point is not as prominent anymore for the ditches as it was repeating the previous sentence and the groups mentioned are common found groups of invertebrates.

384 but is this true? Did you observe more plants or algae in ditches? Did you observe higher diversity of primary consumers? Moreover, this is assuming that the food web is based on primary production, but disregarding the role of detritus to sustain aquatic food webs, this is, all allochthonous inputs.
We did not measure the primary production, therefore we wrote “could be explained by …” as an educated guess to explain our findings. This statement does not differentiate between the allochthonous and autochthounous inputs as they are not measured. But the ditches are characterized by grassland surroundings so not so much inputs are expected from the outside. Only the number of sampled primary consumers led to that interpretation. See also Table 2 for the numbers.

Additionally added in text in chapter 3.2: “ In ditches, a mean value of 44.5 N was recorded for primary consumers and a mean value 26.5 N of omnivores and a mean value of 42.25 N of predators was recorder (Table 2). In comparison, a mean value of 36 N for primary consumers, 22.6 N for omnivores and 17.3 N for predators was recorded in ponds (Table 2).”

393 but you didn’t compare species richness
Added some information in the beginning of the results section: “An average of 76 N was analysed in ponds and 113.3 N in ditches (Table 2). In S5 an overview of the sampled FFGs and determination individuals analysed in pond and ditches is given. In ponds 22 families and in ditches 29 families were found during the sampling campaign.”

According to (58), the effects of long-term nutrient enrichment should lead to a reduction in species numbers and shift community structures. Although we did not directly measure species numbers, we did not observe this phenomenon in the ditches compared to the ponds, as the number of families and individuals found is greater in ditches.

396 what do you mean by more homogeneous communities for predators? This term could be confusing, and it is not to be compared to having lower diversity.
Changed the word homogenous to less diverse

400 this paragraph is describing the results from the figure, not discussing the further implications of such results. It should be moved to the Results section and discussed in the Discussion. Moreover, I failed to see why the ample preference of omnivorous is restricted to polluted waters, since they are, by definition, feeding on multiple types of resources.
The results are presented in the supplement. To not unnecessarily enhance length of the paper by extending the results section when transferring Table S4 to the main manuscript body, we prefer to stick on this kind of presentation, as it provides the basis for the discussion part at the end of this paragraph.

Omnivorous preference is not restricted to polluted waters, this is not written in the text. But it is stated in the text, that omnivorous invertebrates tend to shift to either predatory or herbivorous lifestyle in polluted waters, which is observable in S4 Fig.

410 same, these are Results. Please reference the figure or table that they refer to.
Moved to chapter 3.2 Comparison of functional feeding groups. Reference to figure 6 added.

414 as mentioned before, I recommend you keep your interpretation in terms of the feeding resources, not the habitats or microhabitats
Deleted the point with the microhabitats, but kept habitats later in the sentence, as a larger number of resources available can be explained by the more diverse habitats protocolled in the field in and around the ponds.

415 but you found higher diversity in ponds. Where is the link with the lake’s reference?

Addressed. We changed the sentence to “A low diversity of littoral habitats was shown to be associated with low numbers of trophic links and less resource and consumer diversity (61), which might be the case in the ditches in our study”.

418 what do you mean by primary producer-rich? As in having higher primary production? Measured how? Or as in having higher diversity of primary producers? Measured how?
We deleted primary producer-rich, as we have not analyzed primary producers.

In ditches we seem to have a low diversity of resources, but the present resources are abundant. This is taken from the argument before lines 422 – 424 and it is shown by the lower ellipse area of collector/gatherer and grazer/scraper in ditches compared to ponds.

427 see my previous comments on this conclusion
Text changed as follows: The absence of a distinct difference, which is attributable to the stressors nutrient concentrations and pesticide residues, is also underlined by the dbRDA.

436-439 these sentences are disconnected to the starting sentence of the paragraph.
Yes, true. Sentence deleted, as this fact is already stated in chapter 2.5.3 SIBER (Stable Isotope Bayesian Ellipses in R)

443 explain why their food resources would be low in November
explanation added: “…food base is low due to the reduced availability of suspended particles as a result of less algae growth and less agricultural activity and therefore less erosion, …”

444 in deed, organisms need current to filter; therefore, it is expected to have low densities of filterers in water bodies without current.

Yes.

448 explain why sampling in autumn affects the detectability of invertebrates and pesticides
Explanations added:

“…as the larvae have already emerged from the water bodies to reproduce and lay eggs.”

“Pesticide residues in winter are lower, as pesticides are mainly applied in spring and early summer.”

456 explain why is this recommended

Explanation added: “…recommended to sample larvae stages of merolimnic invertebrates and to match the period of pesticide applications (62,63).”

Conclusions
If the other sections are revised, this section would also require modifications accordingly.

Addressed. Modifications accordingly to changes in discussion part made.

# Reviewer #2:

Dear Authors,

Thank you for your thorough revision of the manuscript. It is evident that you have made significant efforts to address the reviewers' comments, and many aspects of the manuscript have improved as a result. That said, there are still several points that require further attention to enhance the clarity, coherence, and scientific rigor of the work.
Most notably, improvements to the language are recommended to increase readability and avoid unnecessary or unclear repetitions. In particular:
_Some sentences are redundant, as the information they convey has already been clearly stated in preceding text.
_Paragraph structure can be improved by shifting certain sentences to new paragraphs for better flow and thematic clarity.
_Several sentences would benefit from rephrasing to improve clarity and avoid grammatical issues such as the repetitive use of conjunctions (e.g., multiple occurrences of “but” in the same sentence).
_In a few instances, sentences are overly long and difficult to follow, and should be restructured into more concise and digestible forms.
_All abbreviations should be defined at their first mention in the text to ensure clarity for all readers.
_Certain terminology (e.g., "realized niche") may be unclear in this context. More precise phrasing is suggested to better convey the intended meaning.
_Some key methodological choices would benefit from the inclusion of appropriate references to justify their use.
_As a general comment, enhancing the narrative structure and clarity of expression will improve the overall impact of the manuscript and make it more accessible to a broader readership. A careful final language edit is strongly recommended.
For more detailed suggestions and specific examples, please refer to the attached PDF version of the comments.

**Author’s response:**

We thank the Reviewer for the time that he/she invested in evaluating our manuscript again and we hope that we have addressed all the concerns raised by the reviewer now.

**Comments reviewer 2 (black) with author’s responses (green):**

## Introduction

40 delete “had”

Addressed.

44 write loss instead of losses

Addressed.

47-49 This sentence is redundant (as examples are already mentioned in the previous sentence)

Sentence deleted.

49/50 this sentence (and the text that follows) can be shifted to new paragraph. Also, consider rephrasing into: "Ponds and ditches serve as biodiversity hotspots and act as aquatic corridors within agricultural landscapes, highlighting their importance for conservation efforts." (to increase clarity)

Addressed as suggested.

50-53 rephrase to avoid two "but" in the same sentence. Also, rephrase the following sentences to improve the narrative and avoid unnecessary repetition.

Changed as follows: “Although these small water bodies are usually not included in monitoring programs (5,7) their importance for benthic macroinvertebrate biodiversity and ecosystem services is increasingly gaining recognition in research activities (5,8–13).”

51 "water bodies"?

Addressed.

67 I suggest to shift this sentence into new paragraph

Addressed.

69 you should provide the explanation of the abbreviation by the FIRST mention in text (you provide explanation in the sentence below, but it should be here)

Addressed, based on suggestions from the other reviewer the sentence was deleted and the explanation for the abbreviation follows in the sentence.

74 I suggest to shift this sentence into new paragraph

Addressed.

86-91 rephrase this sentence - it is too long and hard to follow

Addressed and rephrased as follows: “Stable isotope ratios of carbon and nitrogen allow the quantification of the isotopic niche of a species or taxa group (e.g. Coleoptera, Odonata etc.). From these data, metrics can be derived that reflect isotopic niche width or proxies for resource, enabling statistical comparisons across broad spatial scales and among communities composed of different species (18,30).”

91-93 I suggest to avoid using the phrase "realized niche" (it is not clear). Rather replace with the following: "The isotopic niche can thus serve as a proxy for the actual ecological role of a species, reflecting its use of available resources and habitats."

"These ecological roles are influenced by land use and agricultural stressors, which alter resource availability and habitat quality."

The term is a term used by Hutchinson (1957), I added a definition and changed the sentences partly based on your suggestions as follows: “The isotopic niche can thus serve as a proxy for the realized ecological niche of a species (portion of the ecological niche that a species occupies in nature, after accounting for competition and predation (31)) reflecting their use of resources and habitat (32). The realised niches are linked to land use and agricultural stressors, which alter resource availability and habitat quality.”

95-97 I suggest rephrasing to: "We compared the niche widths of functional feeding groups (FFGs) and entire pond and ditch communities using a geometric method originally developed to analyze the isotopic niches of benthic invertebrates (24)."

I changed the paragraph also according to another reviewer. In the manuscript it is written as follows: “We aimed to assess the influence of agricultural stressors, measured as water toxicity from pesticide residues and eutrophication, on the isotopic niche of FFGs in pond and ditch communities, using a geometric approach originally developed by (24).”

106-108 I don't understand this sentence

I rephrased the sentence as follows: “Ponds and ditches representing water body types with varying exposure to agricultural effects (nutrient enrichment, pesticides, and the proportion of surrounding crops) were used to test our hypotheses.”

108/109 This sentence should also be reshaped for clarity

Sentence shortened: “We asked the following questions:”

111 delete “To answer these questions,”

Addressed

## Methods

128 Genus should be in italic format

Addressed

129 Genus should be in italic format

Addressed

130 Genus should be in italic format

Addressed

152 I still miss the reference that would justify the usage of this method

References given in response to the reviewers added in the manuscript.

168 Insert Isobank reference

Unfortunately Isobank has funding problems and cannot provide support for data upload at the moment. As an alternative, we have submitted our data to PANGEA. They are currently being processed there. As soon as a DOI has been assigned, it will be added.

## Results

235/236 This is for the results

The section is part of the methods chapter as it is the basis for further data processing, therefore it was not moved to the results section.

241/242 This is for the results

The section is part of the methods chapter as it is the basis for further data processing, therefore it was not moved to the results section.

## Discussion

433/434 How did you get to this conclusion? Include the arguments stated in the authors' responses to reviewer comments

Explanation added: “…here, as the variables explaining those best are all connected to primary production.”

444 reference needed for these statements

References added.
