# Supplementary material for: Assessing agricultural effects on benthic invertebrate communities in ponds and ditches using δ¹⁵N and δ¹³C isotope niches
Source: PLoS One. 2025 Nov 24;20(11):e0336486. doi: 10.1371/journal.pone.0336486 (PMC12643296; doi:10.1371/journal.pone.0336486)
Supplement: Attachment — Submitted filename: Response to Reviewers_v3.docx [file pone.0336486.s013.docx]

**Author’s response:**

We thank the Academic Editor for handling our manuscript and for selecting qualified reviewers. We greatly appreciate receiving such a constructive and detailed review. In response, we have revised the manuscript accordingly again and believe we have addressed all questions and concerns raised. Below, we provide the reviewer’s comments along with our corresponding responses.

**Comments to the Author**

1. If the authors have adequately addressed your comments raised in a previous round of review and you feel that this manuscript is now acceptable for publication, you may indicate that here to bypass the “Comments to the Author” section, enter your conflict of interest statement in the “Confidential to Editor” section, and submit your "Accept" recommendation.

Reviewer #1: (No Response)

____________________________________

2. Is the manuscript technically sound, and do the data support the conclusions?

The manuscript must describe a technically sound piece of scientific research with data that supports the conclusions. Experiments must have been conducted rigorously, with appropriate controls, replication, and sample sizes. The conclusions must be drawn appropriately based on the data presented.

Reviewer #1: Partly

________________________________________

3. Has the statistical analysis been performed appropriately and rigorously?

Reviewer #1: No

________________________________________

4. Have the authors made all data underlying the findings in their manuscript fully available?

The PLOS Data policy requires authors to make all data underlying the findings described in their manuscript fully available without restriction, with rare exception (please refer to the Data Availability Statement in the manuscript PDF file). The data should be provided as part of the manuscript or its supporting information, or deposited to a public repository. For example, in addition to summary statistics, the data points behind means, medians and variance measures should be available. If there are restrictions on publicly sharing data—e.g. participant privacy or use of data from a third party—those must be specified.

Reviewer #1: Yes

________________________________________

5. Is the manuscript presented in an intelligible fashion and written in standard English?

PLOS ONE does not copyedit accepted manuscripts, so the language in submitted articles must be clear, correct, and unambiguous. Any typographical or grammatical errors should be corrected at revision, so please note any specific errors here.

Reviewer #1: Yes

________________________________________

# 6. Review Comments to the Author

Please use the space provided to explain your answers to the questions above. You may also include additional comments for the author, including concerns about dual publication, research ethics, or publication ethics. (Please upload your review as an attachment if it exceeds 20,000 characters)

Reviewer #1: I have finished reviewing the manuscript for the third time. The manuscript has improved compared to the previous versions. However, I still have significant comments that, in my opinion, must be addressed before publication. I regret to say, once more, that the authors’ responses are occasionally not satisfactory. Moreover, while I appreciate their responses and explanations directed to me, my request and questions are finally made to improve understanding from the readers. In that sense, replying to me, but not fully addressing the issues in the text is not helpful for future readers of their work. Some explanations and changes made from the previous version to this one turned out to be more confusing to me. Specifically, the maize corrections for C. Some analyses and figures are presented with the correction, which leads to the conclusion that there are not differences between water body types, whereas others are presented without the correction, in which case significant differences emerged. In my previous comments, I asked about the different results when correcting versus not correcting, and the answer was that it was only intended as an additional visualization. However, the conclusions are dramatically different between the two methods.

Additional comments are detailed below.

**Author’s response:**

We thank the reviewer for the time that she invested in evaluating our manuscript again and we hope that we have addressed all the concerns raised by the reviewer now.

Regarding the mentioned corrections for maize I would like to stress that we did the correction for only one pond to be able to compare ponds and ditches and the influences on them of the agricultural stressors (nutrients and pesticides) in general without looking at the extreme effects occurring only in a single pond. Those extreme effects would override the general effects of the stressors we aimed at. The effects of manure fertilization in ditches were corrected as well to check if we can see differences for the stressor pesticides between the two distinct water body types. I made this explanation also more clear in the text. See lines 298 – 304.

Furthermore for some questions the values were corrected for C and N. This was done to see whether differences among FFGs exist beyond these obvious factors. This explanation was also added in the method section, lines 292 – 295.

**Comments reviewer 1 (black) with authors responses (green):**

## Abstract

15 delete only, as they are not only based on taxonomy, they also account for abundance.

Addressed

30 again, you didn’t measure abundance because you stopped counting when reaching 10 individuals. They were more diverse because you identified more taxa.

Addressed. “No clear pattern emerged when comparing the FFGs in the two water body types, but some differences were found, for example in FFG *predator*, which were found in the ditches with several different taxa and were thus more diverse.”

## Introduction

54 for measuring biodiversity in ponds and ditches affected by agricultural activities… or something similar that provides context for the sentence.

Addressed as suggested: “Thorough knowledge on the status and indicators for measuring biodiversity in ponds and ditches affected by agricultural activities is needed.”

86 taxonomic group since taxa is plural

Addressed.

87 for resource use or consumption

Addressed: “….proxies for resource use, …”

91 the habitat use is not reflected in isotopic compositions because it is not incorporated in the tissue. This applies for the rest of your ms, when you mention habitat use or habitat diversity. For this reason, I recommended in my previous comments to keep the terminology as feeding resources and remove the habitat/microhabitats.

I removed the term microhabitats to avoid confusion, but I still disagree about the term habitat.

Isotopic compositions give information on the habitat occupied by organisms. See also: Newsome, Seth D.; Del Martinez Rio, Carlos; Bearhop, Stuart; Phillips, Donald L. (2007): A niche for isotopic ecology. In: Frontiers in Ecology and the Environment 5 (8), S. 429–436. DOI: 10.1890/060150.1. “Stable isotope analysis provides quantitative information on both resource (bionomic) and habitat (scenopoetic) use commonly utilized to define ecological niche space”

See also as an example: <https://www.sciencedirect.com/science/article/pii/S2053716622000032>

96 connect this sentence with the following paragraph

Addressed as follows: “We aimed to assess the influence of agricultural stressors, measured as water toxicity from pesticide residues and eutrophication, on the isotopic niche of FFGs in pond and ditch communities, using a geometric approach originally developed by (24). Given that ponds and ditches are likely affected differently by these stressors, with consequent impacts on habitat availability and quality, we hypothesize that such variations ultimately shape benthic invertebrate food webs and niche size (33,34).

100 morphological characteristics

Addressed.

101 in addition, ditches are probably more contaminated…

Addressed.

103 protected by a riparian buffer?

Yes, addressed.

104 act as a source of pesticides? How?

Explanation and literature added in the text as follows: “Ponds, on the other hand, are better protected by a riparian buffer from pesticide inputs, but can act as a sink (e.g. sediment deposition and retention due to long retention times and closed systems, biological uptake in plants) and source (e.g. remobilisation from sediment due to disturbances, contaminated groundwater connection) for pesticide residues in the sediment (35,36).”

109 I personally find the word comparable vague because I don’t know if you mean they can be compared with one being larger than the other (which, of course, they can, using isotopic compositions!) or they are similar.

I changed the question as follows, not using the word comparable: “Do the observed patterns of isotopic niches of FFGs comparable differ between ponds and ditches?

## Methods

150 necessary for the isotopic analyses. Moreover, how did you estimated the weight in the field?

Explanation added in the text: “Necessary sample weights were estimated by visual inspection in the field. The exact weighting in the tin capsules for analysis was carried out by the analysing lab, the Cornell Stabel Isotope Facility.”

148-149 however, in your response to my previous comment, you said that taxa were actually sorted more than at the family level (i.e., you did not gather individuals from different genera in the same group).

The lines 148/149 referred to the sorting on site. Here we only sorted organisms into order or family level. Later in the lab we further determined the organisms either to family level or to genus level. See lines 155-157: “In the laboratory, the benthic macroinvertebrates were further taxonomically determined to family (where the order was assigned on site) or genus level when possible and sorted into the FFGs based on the freshwater ecology database (40).”

179 that could explain

Addressed.

225 by lack of information or because the family includes species from different feeding groups?

Could be for both reasons you mentioned here. Additional some taxa are defined as omnivores. Added in the text: “…for a number of reasons, such as lack of information in the database, species with different feeding groups in the family or due to the assignment of the family to the group omnivore by the database.”

262 move this paragraph up to line 258

Changed as suggested.

269 what is the difference from TA from the previous paragraph? Were the metrics in the previous paragraph not calculated as well for FFG in ponds and ditches?

As stated in the text lines 269: “Additionally, the Bayesian standard ellipse areas (SEA.B), corrected standard ellipse areas (SEAc), and convex hull area (TA) **based on Bayesian interference were calculated.**”

Thus the TA here was calculated based on Bayesian statistics and not based on the frequentist statiscical approach as used by Layman. Which is described above.

272 meaning that in practical terms, TA, SEA.B and SEA.c provide the same information: niche width. Any reason for including the three of them?

Differences explained in more detail in the text: “TA is the total convex hull area enclosing all data points and thus showing the full data range. SEA.B is the Bayesian version of the standard ellipse area, usually compromising 40 % of data points, providing a robust estimate with credible intervals that account for uncertainty (30). The SEA.c based on SEA.B but is adjusted for small sample sizes, a frequentist measure of the core niche that gives a single point estimate (30).

281 I do not follow this new justification. Both C- and N-corrections were made because one water body showed enrichment of either C or N. Then, why now is only one being considered? Moreover, if the C-correction was not used in the analysis, remove the section where it is explained. However, in your Fig. 4 there is a clear difference in the isotopic space with and without correction, so it does have an effect.

We made the use of corrections more clear in the text: “The calculation of the Bayesian Layman metrics (Layman.B) was performed for the data set with corrected δ15N values, as this was a general phenomenon observed for all ditches and changes throphic position in comparision to ponds and thus could override effects of for example pesticides Maize cultivation was not a general phenomenon like fertilization in the study area, thus the original δ13C values were kept for the following calculations. In other words, the real C-source (resource use) should not be artificially changed because of the niche width, but the N-source had to be scaled to the same level for the two water body types for comparability of trophic position without possible overriding effects of fertilization.”

Yes, of course. The corrections do have an effect this is what figure 4 illustrates. Corrected for N and C values makes the two water body types overlay. The explanation above and added in the text should make clear why δ^14^N correction is used in the following analysis and C correction is only illustrated in figure 4.

294 my previous question was not answered. Why EC and O2 have small sample size but temperature and pH don’t? they were all measured with a probe. Additionally, in the text and in your answer, it was stated they were measured once, then why there is variability during the day?

Yes, true. I meant the natural variability during the day, not measured variability. And the small sample size is meant as the small sample size of seven water bodies in general, thus I cannot use a large number of explanatory variables in the dbRDA.

Changed in the text as follows: “Selected environmental variables by the bioenv function, excluding the variables EC and O2, due to small sample size of seven water bodies and the natural expected variability of the variables during the course of the day,…”

Also pH and temperature vary naturally during the day and we only have a small sample size as you said. But those variables were already excluded by the use of the bioenv function, explanined in the lines above. This is the reason they are not mentioned here.

305 this are the number of samples, correct? Then, I recommend not using N since it might be confusing with nitrogen.

Yes, it is the number of samples. Addressed.

## Results

310 then again, you said that the maize correction was not necessary.

Yes, it was not used for the further analysis as reasons are also clarified above now, but it is still a result worth showing in my opinion. So it is mentioned here as I also show the figure 4.

317 significantly distinct

Addressed.

319 closing dash is missing (and they should be m-dashes)… are significantly larger…

Changed as follows: “). The posterior distribution of TA and thus total niche width and the spacing of individuals (mean distance to centroid, CD) are significantly larger in pond communities compared to communities in ditches (Fig. 5 b, e).”

Why is Fig. 5 again only corrected by N? It is very confusing how the authors are mixing results regarding the corrections. This makes very hard to assess the outcomes of these analyses.

I hope the clarifications provided now above in the method section make this clear. Correction for the fertilization in ditches is kept for the analysis as it is a general phenomenon in all ditches and could override other effects. Corrections for the one maize pond is not incorporated in the analysis as it is not a general phenomenon. This correction is only shown in the figure four to illustrate the effect of maize on one pond. This is explained in the manuscript in the methods section in detail.

331 …for primary consumers, a mean value 26.5 N for omnivores, and a mean value of 42.25 N for predators… See my previous comment about the use of N for sample size

Addressed. N, changed to “isotope samples”

Moreover, I assume primary consumers refer to grazers, collectors, and shredders? Please, stick to the same terms throughout the manuscript, in this case, terms of table 2. Otherwise, explain why this grouping was made.

Addressed. Term defined in brackets in the text. “…(*collector/filterer, collector/gatherer, grazer/scraper and shredder*)”.

337 groups

Addressed.

344 I see how my previous comment was not addressed. Your method does not estimate abundance, because you stopped counting until reaching a certain number of individuals. Therefore, you cannot argue that they were observed in larger numbers. Could taxa group be replaced simply by order?

Addressed. Sentence changed in the text: “Predators were in general observed with more taxa groups in ditches. Six orders (Coleoptera, Diptera, Heteroptera, Hirudinea, Megaloptera, Odonata) and 13 families and were recorded there, while five orders (Coeloptera, Diptera, Heteroptera, Odonata, Trichoptera) and eight families were recorded in ponds (S5 Table).”

365 is M the mean? This is not an standard acronym. Do these values correspond to the column Pesticide detections in Table 4? The column does not have units.

Addressed. Written “mean”, instead of M in the text.

No. for number added in the column pesticide detections in table 3 and the units deleted for the numbers. There were not supposed to be there.

## Discussion

383 but you did find differences in the C range, TA, distance to centroid, MNND (Fig. 5). However, this is because this figure was not corrected by C. The fact that some figures/analyses are corrected and some others are not makes the interpretation difficult. Which results are to be used, with or without correction? And based on that, are or are there not differences?

Added some more explanation on the use of corrections in the method section in chapter 2.5.3 as follows: “Both corrections were applied to eradicate the strong influence of fertilization in ditches and the maize effect in one pond, allowing us to set the focus on the FFGs and to investigate whether differences among FFGs beyond these obvious factors.”

This applies to the whole discussion since some paragraphs seem to be based on corrected values (e.g., paragraph 2) and others not (paragraph 1).

Yes, paragraph one gives general information on the meaning and results of the corrections as well as related literature.

Paragraph 2 focuses on the use of resources and the Layman metrics calculated and thus on the δ^15^N correction only.

The reasons for the corrections for the different questions should be made clear in the methods section, now.

391 expected

Addressed.

395 is higher in ponds

Addressed.

395 led to enriched values

Lead changed to led, but I would not call it enriched values. The ratios of the isotopes are more extreme due to different resources present. It is not per se enrichment.

401 these are not diversity values. These are the number of samples that you used, which should not be used as a diversity metric. A diversity metric is, for example, the number of families.

As also mentioned in your comment in the abstract, the predators are more diverse because we identified more taxa in our sample. So it is a diversity metric = number of families. There were more families of predators in the samples. Furthermore I changed the word diversity in the following sentence to number.

Changed in the text as follows: “Contrary to our expectations, the FFG predator was more diverse in ditches, we identified more families in ditches. The high number of predators in samples in ditches (mean 42.25 in ditches and mean of 17.3 in ponds)…”

402 but again, this is highly speculative since you didn’t measure primary production. More prey could also come from filterers, gatherers, or shredders consuming FPOM or CPOM.

Added the following sentence in the text to make this point also clear in the discussion: “However we have to be careful with this conclusions as we did not measure primary production.”

407 ok but highest densities are not to be compared with higher diversity. They could have found higher densities of a few (or even one) taxa, while you found higher diversity (more families) or predators.

They were both found for predators. High mean values in isotope samples and high taxa number identified. Added in the sentence in the discussion: “The high taxa number and number of mean isotope samples is probably stimulated by bottom-up effects of nutrient enrichment (54,59).”

408-411 but these two sentences are completely contradicting each other!

Yes, I made this clearer in the text: “The high taxa number and number of mean isotope samples is probably stimulated by bottom-up effects of nutrient enrichment (54,59). In contrast to that, according to (58), the effects of long-term nutrient enrichment should lead to a reduction in species numbers and shift community structures. Although we did not …”

I wanted to discuss different results from the literature.

412 but nutrient concentration was higher in ditches.

Yes, therefore I did write: “we did not observe this phenomenon…” made this more clear with the end of the sentence as well: …” we did not observe this phenomenon in the ditches compared to the ponds, as the number of families and individuals found is greater in ditches even though the nutrient concentrations were higher in ditches.

In general, this paragraph is very confusing and contradictory.

I wanted to discuss the results in comparison with different findings from the literature. I think the additions above should eradicate the confusion in the paragraph.

414 it seems to be a typo: more less

Yes, typo. The word more was deleted.

427 I partly disagree. The shift could occur in polluted water bodies, or in any water body for multiple reasons. Mainly, resource availability, which changes seasonally, according to the longitudinal position, riparian structure, etc… that is why they are omnivores.

Agreed. The point is added with literature in the manuscript as follows: “Nevertheless responses of omnivores are dynamic and shifts could also occur due to other perturbations, e.g. temporal shifts in resources across seasons.”

434 how do you conclude they are highly abundant?

I deleted that part of the sentence. It was more an observation than based on data.

434 see my comment in the intro about why isotopic compositions are not meant to be used for habitat use, but for feeding resources, which are not the same. Also, keep consistency between habitat and microhabitat-unless you mean something different-, although my suggestion is to remove the habitat part.

I deleted the term microhabitats to avoid confusion, but as explained above isotopic compositions are applied to get information for the habitat use by organisms. See my comment regarding your comment on the introduction, line 91. And the explanation provided.

I also added some explanation in the manuscript for the habitats in brackets: “Additionally, the larger number of microhabitats (e.g. macrophytes, reeds, CPOM) and/or more resources used can also explain commonly observed patterns of higher taxa richness and the associated possible different preferences in ponds (7–9).”

466 do you mean autumn?

Yes, added autumn in the manuscript.

________________________________________

7. PLOS authors have the option to publish the peer review history of their article (what does this mean?). If published, this will include your full peer review and any attached files.

If you choose “no”, your identity will remain anonymous but your review may still be made public.

Do you want your identity to be public for this peer review? For information about this choice, including consent withdrawal, please see our Privacy Policy.

Reviewer #1: Yes: Daniela Cortes

**________________________________________**
