# Supplementary material for: Assessing agricultural effects on benthic invertebrate communities in ponds and ditches using δ¹⁵N and δ¹³C isotope niches
Source: PLoS One. 2025 Nov 24;20(11):e0336486. doi: 10.1371/journal.pone.0336486 (PMC12643296; doi:10.1371/journal.pone.0336486)
Supplement: Attachment — Submitted filename: Response to Reviewers_v4.docx [file pone.0336486.s014.docx]

**Author’s response:**

We thank the Academic Editor for handling our manuscript and for selecting qualified reviewers. We greatly appreciate receiving such a constructive and detailed review. In response, we have revised the manuscript accordingly again and believe we have addressed all questions and concerns raised. Below, we provide the reviewer’s comments along with our corresponding responses.

**Comments to the Author**

1. If the authors have adequately addressed your comments raised in a previous round of review and you feel that this manuscript is now acceptable for publication, you may indicate that here to bypass the “Comments to the Author” section, enter your conflict of interest statement in the “Confidential to Editor” section, and submit your "Accept" recommendation.

Reviewer #1: (No Response)

________________________________________

2. Is the manuscript technically sound, and do the data support the conclusions?

The manuscript must describe a technically sound piece of scientific research with data that supports the conclusions. Experiments must have been conducted rigorously, with appropriate controls, replication, and sample sizes. The conclusions must be drawn appropriately based on the data presented.

Reviewer #1: Yes

________________________________________

3. Has the statistical analysis been performed appropriately and rigorously?

Reviewer #1: Yes

________________________________________

4. Have the authors made all data underlying the findings in their manuscript fully available?

The PLOS Data policy requires authors to make all data underlying the findings described in their manuscript fully available without restriction, with rare exception (please refer to the Data Availability Statement in the manuscript PDF file). The data should be provided as part of the manuscript or its supporting information, or deposited to a public repository. For example, in addition to summary statistics, the data points behind means, medians and variance measures should be available. If there are restrictions on publicly sharing data—e.g. participant privacy or use of data from a third party—those must be specified.

Reviewer #1: Yes

________________________________________

5. Is the manuscript presented in an intelligible fashion and written in standard English?

PLOS ONE does not copyedit accepted manuscripts, so the language in submitted articles must be clear, correct, and unambiguous. Any typographical or grammatical errors should be corrected at revision, so please note any specific errors here.

Reviewer #1: Yes

________________________________________

6. Review Comments to the Author

Please use the space provided to explain your answers to the questions above. You may also include additional comments for the author, including concerns about dual publication, research ethics, or publication ethics. (Please upload your review as an attachment if it exceeds 20,000 characters)

Reviewer #1: I have finalized revising the manuscript again. I acknowledge the effort of the authors to address all previous comments, which is reflected in the improvement of the manuscript. I still have one general comment and two major issues. My general comment is about the effects of the N and C corrections. I think that the authors could emphasize the reasoning of the corrections in the discussion, to clarify and remind readers of why some figures and results are based on one/both corrections and others are not.

**Author’s response:**

We thank the reviewer for the time that she invested in evaluating our manuscript again all concerns should be addressed now.

Regarding the general comment on the N and C corrections we added a paragraph in the beginning of the discussion chapter and think that this clarifies the use of the corrections for our readers. The paraghraph was added as follows: “The corrections for the influences of fertilizers (δ^15^N) and maize (δ^13^C) were applied to eradicate the overlaying effects, allowing us to set the focus on the differences between either the two water body types or the FFGs in the two water body types and their niche overlaps. In order to investigate whether differences among FFGs niches beyond the obvious factors (fertilization, maize) can be detected we corrected for both isotopes. For comparisions of the two water body types only corrections of δ15N (fertilizer) were applied.”

**Comments reviewer 1 (black) with authors responses (green):**

My major issues are:

1. The assignment of taxonomic groups without FFG information to omnivores. This is not trivial, as you are arbitrary assigning NAs to one functional group, which could have an important effect on your results. For example, this could explain the clusters you observed in your analysis. Dealing with NA is an extensively discussed topic in Ecology, and among the multiple approaches to address it, arbitrary assigning values is not a recommended one. My suggestions are as follow: you can remove these taxa from your FFG analysis, and justify this because of the lack of information. You can extend your literature sources to find the appropriate FFG category for such taxa; given the generally coarse taxonomic level you managed (order, family, or genus), I am convinced that this information is available for European taxa. Finally, you can repeat your analysis excluding these taxa and compare to your current results to discard (or not) the influence of your approach.

The table below is a subset from the appendix with all individuals defined as omnivores throughout the study. The added notes indicate the information on feeding preferences we based the decision to define the taxa as omnivores. Notes show that the decision to define the taxa as omnivores is based on information from the literature.

**Tables:** Taxa assigned to the FFG omnivore with the given information assigned from the freshwater ecology database (Schmidt-Kloiber, Astrid; Hering, Daniel (2015). Ten categories, numbers in brackets in notes) are defined according to Moog (1995).

| **FFG** | **ponds** | | | | |
| --- | --- | --- | --- | --- | --- |
|  | **taxagroup** | **family_**  **subfamily** | **genus** | **N** | **notes** |
| omnivore | Coleoptera | Haliplidae | *Haliplus* sp. | 4 | assigned to grazers/scrapers (2), miners (4), shredder (1) and predator (3) by freshwater ecology database, ten point system 🡪 therefore listed as omnivore |
| omnivore | Diptera | Chironomidae | NA | 33 | assigned to grazers/scrapers (2), miners (1), gatherers/collectors (3), active filter feeder (2), predator (3) and parasite (1) by freshwater ecology database🡪 therefore listed as omnivore |
| omnivore | Heteroptera | Corixidae | *Sigara* sp. | 9 | knowledge is contradictory, assigned to gatherers/collectors (10) by freshwater ecology database, feeding habitas according to Tachet et al. (Schmidt-Kloiber and Hering 2015) are more diverse as well as according to Hädicke et al. 2017, so we also assigned them as omnivores |
| omnivore | Heteroptera | Corixidae | NA | 22 | knowledge is contradictory, assigned to grazers/scrapers (2), miners (3), gatherers/collectors (5) by freshwater ecology database, but from our data and literature (<http://publication.nhmus.hu/pdf/folentom/FoliaEntHung_1978_Vol_31_2_19.pdf> and Hädicke et al. 2017) they are known to be also predatory or omnivore 🡪 therefore listed as omnivores |

| **FFG** | **ditches** | | | | |
| --- | --- | --- | --- | --- | --- |
|  | **taxagroup** | **family_**  **subfamily** | **genus** | **N** | **notes** |
| omnivore | Coleop-tera | Haliplidae | *Haliplus* sp. | 34 | assigned to grazers/scrapers (2), miners (4), shredder (1) and predator (3) by freshwater ecology database 🡪 therefore listed as omnivore |
| omnivore | Diptera | Chironomidae | NA | 36 | assigned to grazers/scrapers (2), shredder (3), gatherers/collectors (3) and active filter feeder (1) by freshwater ecology database 🡪 therefore listed as omnivore |
| omnivore | Diptera | Stratiomyidae | NA | 2 | assigned to grazers/scrapers (3), miners (1), gatherers/collectors (3), active filter feeder (2), predator (3) and parasite (1) by freshwater ecology database 🡪 therefore listed as omnivore |
| omnivore | Heterop-tera | Corixidae | NA | 34 | see Corixidae above |

Based on the information given in the tables we did not follow the recommendation to repeat the analysis excluding taxa without information, as we do have information on the taxa. The FFG omnivore is not assigned based on a lack of knowledge. This was written misleading in our previous comment for the reviewer. We apologize for the confusion.

2. Your discussion about the predator diversity explained by increased primary production. Once more, I consider that adding “However we have to be careful with this conclusions as we did not measure primary production.” is not enough to address the comment. As I explained before: first, this is especulative as you didn't measure PP; however, you did measure FFG, and you can quantify the number of potential preys available and easily check/discuss if they are more preys in ditches than in ponds. Second, more importantly, one would expect that increasing PP—although only hypothetical—leads to a higher number of primary consumers, particularly grazers. But this is not what you observed. An alternative, which is not discussed in your text, is that predators are not being sustained by a green food web, but by a brown one. This is, instead of PP, there are more detritus sustaining preys (but this also needs to be supported by your own results) and, therefore, sustaining predators. As I mentioned in my previous comment, more available prey could come from filterers, gatherers, or shredders consuming FPOM or CPOM.

Yes, we did observe larger numbers of primary consumers in ditches. Also, the numbers of grazers are higher in ditches. This is all listed in table 2. As suggested, we added the potential numbers of available prey in the discussion chapter and added a discussion and literature on the raised point of the alternative of a brown food web sustaining the predators as follows:

“This was underlined by the results, which showed that the potential number of prey was higher in ditches (mean primary consumers = 44.5 N and mean omnivores = 26.5 N), compared to ponds (mean primary consumers = 36 N and mean omnivores = 22.6 N). However, we have to be careful with this conclusion as we did not measure primary production. Alternatively, predators could be sustained by the bown food web to a higher extent in ditches (mean shredder = 61 N) than in ponds (mean shredder = 31 N). This would also be in line with an earlier modelling study indicating that predators may rely on energy derived from the brown food web but also underlines that brown and green food webs are interacting and cascading effects are theoretically in place (60). This could be especially true for non-natural sites, as a study by (61) found that predators rely on detrivores more in restored sites than in reference sites.”

Regarding your second point raised in the comments. We do have higher numbers of primary consumers in ditches, which are most probable characterized by a higher primary production. See text part added above and table two in the manuscript.

**Literature:**

Hadicke, Christian W.; Redei, David; Kment, Petr (2017): The diversity of feeding habits recorded for water boatmen (Heteroptera: Corixoidea) world-wide with implications for evaluating information on the diet of aquatic insects. In Eur. J. Entomol. 114, pp. 147–159. DOI: 10.14411/eje.2017.020.

Moog, O. (ed.) (1995): Fauna Aquatica Austriaca - A Comprehensive Species Inventory of Austrian Aquatic Organisms with Ecological Notes. Federal Ministry for Agriculture and Forestry, Wasserwirtschaftskataster Vienna: loose-leaf binder.

Schmidt-Kloiber, Astrid; Hering, Daniel (2015): www.freshwaterecology.info – An online tool that unifies, standardises and codifies more than 20,000 European freshwater organisms and their ecological preferences. In Ecological Indicators 53, pp. 271–282. DOI: 10.1016/j.ecolind.2015.02.007.

________________________________________

7. PLOS authors have the option to publish the peer review history of their article (what does this mean?). If published, this will include your full peer review and any attached files.

If you choose “no”, your identity will remain anonymous but your review may still be made public.

Do you want your identity to be public for this peer review? For information about this choice, including consent withdrawal, please see our Privacy Policy.

Reviewer #1: Yes: Daniela Cortes
