# Supplementary material for: Assessing agricultural effects on benthic invertebrate communities in ponds and ditches using δ¹⁵N and δ¹³C isotope niches
Source: PLoS One. 2025 Nov 24;20(11):e0336486. doi: 10.1371/journal.pone.0336486 (PMC12643296; doi:10.1371/journal.pone.0336486)
Supplement: Attachment — Submitted filename: Response to reviewers_v5.docx [file pone.0336486.s016.docx]

**Author’s response:**

We thank the Academic Editor for handling our manuscript and for selecting qualified reviewers. We greatly appreciate that all the reviewers comments were handled successfully and there are no additional request. We have handleded the comments made by the editor in the manuscript. Below, we provide the editors’s comments along with our corresponding responses.

**Comments to the Author**

1. If the authors have adequately addressed your comments raised in a previous round of review and you feel that this manuscript is now acceptable for publication, you may indicate that here to bypass the “Comments to the Author” section, enter your conflict of interest statement in the “Confidential to Editor” section, and submit your "Accept" recommendation.

Reviewer #1: All comments have been addressed

________________________________________

2. Is the manuscript technically sound, and do the data support the conclusions?

The manuscript must describe a technically sound piece of scientific research with data that supports the conclusions. Experiments must have been conducted rigorously, with appropriate controls, replication, and sample sizes. The conclusions must be drawn appropriately based on the data presented.

Reviewer #1: Yes

________________________________________

3. Has the statistical analysis been performed appropriately and rigorously?

Reviewer #1: Yes

________________________________________

4. Have the authors made all data underlying the findings in their manuscript fully available?

The PLOS Data policy requires authors to make all data underlying the findings described in their manuscript fully available without restriction, with rare exception (please refer to the Data Availability Statement in the manuscript PDF file). The data should be provided as part of the manuscript or its supporting information, or deposited to a public repository. For example, in addition to summary statistics, the data points behind means, medians and variance measures should be available. If there are restrictions on publicly sharing data—e.g. participant privacy or use of data from a third party—those must be specified.

Reviewer #1: Yes_______________________________________

5. Is the manuscript presented in an intelligible fashion and written in standard English?PLOS ONE does not copyedit accepted manuscripts, so the language in submitted articles must be clear, correct, and unambiguous. Any typographical or grammatical errors should be corrected at revision, so please note any specific errors here.

Reviewer #1: Yes

________________________________________

6. Review Comments to the Author

Please use the space provided to explain your answers to the questions above. You may also include additional comments for the author, including concerns about dual publication, research ethics, or publication ethics. (Please upload your review as an attachment if it exceeds 20,000 characters)

Reviewer #1: (No Response)

________________________________________

7. PLOS authors have the option to publish the peer review history of their article (what does this mean?). If published, this will include your full peer review and any attached files.

If you choose “no”, your identity will remain anonymous but your review may still be made public.

Do you want your identity to be public for this peer review? For information about this choice, including consent withdrawal, please see our Privacy Policy.

Reviewer #1: Yes: Daniela Cortes Guzman

**Comments by the editor:** Dear authors, many thanks for thorough revision, the Reviewer has no additional requests. However, I have made several minor comments and suggestions in the word document of your manuscript that need to be considered before your manuscript can be accepted for publication.

**Author’s response:**

We thank the editor for the time that she invested in evaluating our manuscript all concerns should be addressed now. See below for detailed explanations. Comments by the editor or lines given in black and authors responses in green.

Line 10: Addressed as suggested.

Line 39: Changed to: “Agricultural land covers…”

Line 59: Addressed as suggested.

Line 95: Addressed as suggested.

Line 130: Why didnt you choose the same number of sites per habitat type?

Unfortunately, there were only three ponds in the project study region that had enough water for sampling.

Line 136: Sometimes your sites are refer to as sampling locations, sometimes sampling sites, sometimes waterbodies - please choose only one term and use it in the whole manuscript.

Adressed. Changed to sites throughout the manuscript.

Line 149: Microhabitats/different substrate types

The term microhabitat was deleted throughout the review process. In the methods sampled areas within the water bodies are also described as habitats. Also, the sampled habitats include reed or macrophytes so we are not just sampling different substrate types. Thus, I would like to keep the term habitat.

Lines 152-157: How many samples (individuals) you had per habitat (ponds/lakes) in total?

This information was not recorded. Samples from the habitats were pooled. Sampling was carried out in each habitat seperately first to make sure that all present invertebrate groups were sampled. There is an overview of the samples for pond/ditch in table 2.

Line 156: Addressed as suggested.

Line 163: Addressed as suggested.

Line 188: Addressed as suggested.

Line 247: Addressed as suggested.

Line 252: Addressed as suggested.

Line 292: Addressed as suggested.

Line 293: Addressed as suggested.

Line 312: Addressed as suggested.

Lines 321 – 323: Addressed as suggested.

Line 355: Functional groups are sometimes italic, sometimes not.

Functional groups are written in italics throughout the manuscript.

Line 373: It is better to use the full terms in the legends of the figures and tables.

Addressed as suggested.

Line 379: Water parameters were only measured once, and in one spot?

Yes, water parameters were measured once per sampling site. They are measured in-situ in the mixed water sample taken. I made this more clear in the methods lines 187 - 189 as follows: „Environmental water parameters [oxygen content, electrical conductivity (EC), pH, and temperature] were measured *in-situ* once during sampling in the mixed water sample using a portable meter (WTW Multi 3430 IDS with OxiCal-SL FDO 925, TetraCon 925 and SenTix 940 probes, Germany).

Table 3: Add in the legend with water temperature explanation.

Abbrevation “Temp.” added in table description.

Table 3: Please use the same number of decimal places within a particular parameter, e.g. in pH, all need to have one decimal place, NH4 two etc.

Addressed as suggested.

Line 385: It should be avoided to start a sentence with a number

Addresses as follows: ”The majority of the total pesticides detected (ponds and ditches) are herbicides, accounting for 81%, while 19% are fungicides.”

Line 386: SD shall have the same number of decimal places as the mean.

Addressed as suggested.

Line 392: Addressed as suggested.

Line 406: Addressed as suggested.

Line 426/427: Please use the same number of decimal places for the FFG means, one or two, does not matter, but should be consistent - please correct where necessary

Addressed as suggested.

Line 433: Addressed as suggested.

Line 438: Addressed as suggested.

Line 451: Addressed as suggested.

Line 452: Addressed as suggested.

Line 472: Not changed to microhabitats as suggested. See explanation for keeping the term habitat above.

Line 476: Addressed as suggested.

Line 490: Addressed as suggested.

Line 496: Addressed as suggested.

Line 503: Addressed as suggested.

Line 504/505: Reference?

Application follows the regulations for the pesticide product and the time of crop development. Changed in the text as follws and references added: “Pesticide residues in autumn and winter are lower, as pesticides are mainly applied in spring and early summer due to the general agricultural practice to establish crops such as oilseed rape, maize or cereals during the main growing season (69,70).”

Line 525: Addressed as suggested.
